# Rh(I) Complexes with Hemilabile Thioether-Functionalized NHC Ligands as Catalysts for [2 + 2 + 2] Cycloaddition of 1,5-Bisallenes and Alkynes

**DOI:** 10.1021/acscatal.2c05790

**Published:** 2023-02-17

**Authors:** Jordi Vila, Miquel Solà, Thierry Achard, Stéphane Bellemin-Laponnaz, Anna Pla-Quintana, Anna Roglans

**Affiliations:** † Institut de Química Computacional i Catàlisi (IQCC) and Departament de Química, Facultat de Ciències, Universitat de Girona (UdG), C/Maria Aurèlia Capmany, 69, Girona, 17003 Catalunya, Spain; ‡ Institut de Physique et Chimie des Matériaux de Strasbourg, CNRS-Université de Strasbourg, UMR7504, 23 Rue du Loess BP 43, 67034 Strasbourg, France

**Keywords:** [2 + 2 + 2] cycloaddition, allene, rhodium, N-heterocyclic carbene, DFT calculation

## Abstract

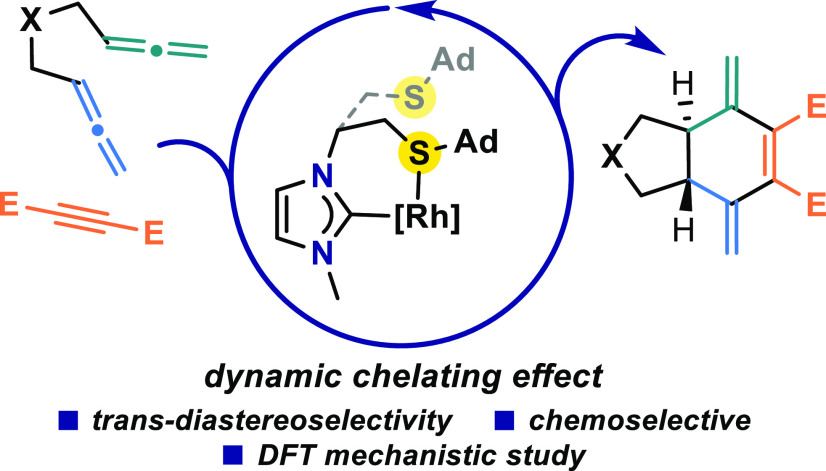

The [2 + 2 + 2] cycloaddition of 1,5-bisallenes and alkynes under the catalysis of Rh(I) with hemilabile thioether-functionalized N-heterocyclic carbene ligands is described. This protocol effectively provides an entry to different *trans*-5,6-fused bicyclic systems with two exocyclic double bonds in the cyclohexene ring. The process is totally chemoselective with the two internal double bonds of the 1,5-bisallenes being involved in the cycloaddition. The complete mechanism of this transformation as well as the preference for the *trans*-fusion over the *cis*-fusion has been rationalized by density functional theory calculations. The reaction follows a typical [2 + 2 + 2] cycloaddition mechanism. The oxidative addition takes place between the alkyne and one of the allenes and it is when the second allene is inserted into the rhodacyclopentene that the *trans*-fusion is generated. Remarkably, the hemilabile character of the sulfur atom in the N-heterocyclic carbene ligand modulates the electron density in key intermediates, facilitating the overall transformation.

## Introduction

**Scheme 1:**
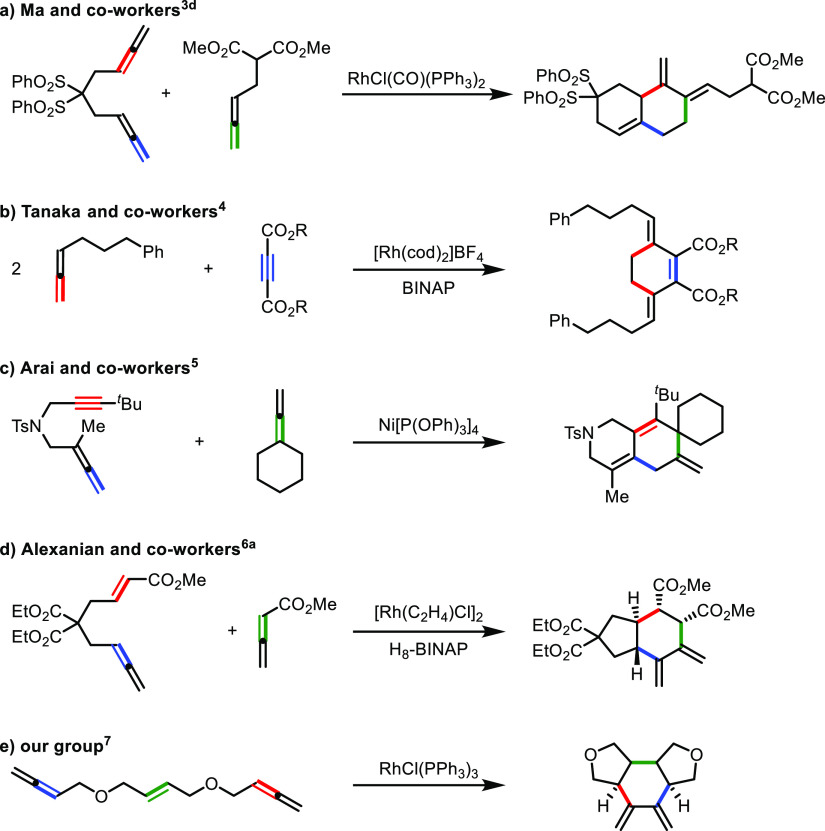
Metal-Catalyzed [2 + 2 + 2] Cycloaddition Reactions of Allenes

Transition-metal-catalyzed [2 + 2 + 2] cycloaddition reactions are a useful tool for the synthesis of six-membered carbo- and heterocyclic compounds in a one-step highly atom economic process.^1^ The different types of unsaturation that can be involved in these processes open the door to a wide range of cyclic derivatives with different functionalities. Among the unsaturations that can be involved in the [2 + 2 + 2] cycloaddition reaction, allenes are particularly versatile as their two cumulated double bonds can individually participate in the cycloaddition. However, with this increased diversity comes greater difficulty in controlling chemoselectivity.^2^ As the number of allenes involved in the [2 + 2 + 2] cycloaddition increases, the number of possible regioisomers also increases. After the pioneering studies by Benson and Lindsey^3a^ in 1958 based on the cyclotrimerization of allene by a Ni(0) catalyst, only a couple of studies by Ma and co-workers involve three allenes in a [2 + 2 + 2] cycloaddition to obtain steroid-like scaffolds.^3b−3d^ Initially, the group described a bimolecular [2 + 2 + 2] cycloaddition of bisallenes under rhodium catalysis giving a diene, which then underwent a Diels–Alder reaction with the remaining allene, giving precursors of steroidal structures.^3b,3c^ A complementary approach to this process was the cycloaddition between a 1,5-bisallene and monoallenes (Scheme 1a).^3d^ The reaction took place chemoselectively between an internal double bond and a terminal double bond of the bisallene and the terminal double bond of the monoallene under rhodium catalysis, giving a bicyclic derivative with two exocyclic double bonds. On the other hand, if we consider the participation of two allenes with an alkyne, we can find different processes based on the nature of the allenes and the nature of the transition metal used as the catalyst. Tanaka and co-workers^4^ described the cross-cyclotrimerization of two monosubstituted allenes with one alkyne to afford 3,6-dimethylenecyclohex-1-ene derivatives resulting from the cycloaddition of the terminal double bond of the allene (Scheme 1b). In contrast, when starting with di- or trisubstituted allenes and using the same catalytic system, a β-hydrogen elimination on the rhodacyclopentene intermediate took place instead of the insertion of the second allene, affording dendralene derivatives. Arai and co-workers^5^ reported the cycloaddition between two different allenes and an alkyne under nickel catalysis and demonstrated by density functional theory (DFT) calculations that the selectivity of the reaction was affected by steric effects around the π-bonds (Scheme 1c). There are also examples involving an alkene with two allenes that have been reported by both Alexanian and co-workers^6^ and our own group.^7^ Alexanian described the stereoselective and enantioselective rhodium-catalyzed [2 + 2 + 2] cycloaddition of ene-allenes and allenoates, affording *trans*-fused carbocycles with four stereogenic centers (Scheme 1d). The authors postulated an initial oxidative coupling of the two allenes involving the two internal double bonds of both allenes. The alkene was then inserted into the rhodacyclopentane establishing the *trans* ring fusion in this step. We also studied the stereoselective cycloaddition of linear allene–ene–allene substrates to afford tricyclic systems.^7^ The Wilkinson complex promoted cycloaddition involving the internal double bonds of both terminal allenes, affording the corresponding exocyclic dienes (Scheme 1e). DFT calculations showed that initial oxidative coupling took place between the internal double bond of one of the allenes and the alkene, affording a *cis* ring fusion. The internal double bond of the second allene was then inserted delivering this time a *trans* ring fusion to afford the exocyclic hexadiene after reductive elimination. In the same study, we also reported an analogous reaction on an allene–yne–allene substrate.

**Scheme 2:**
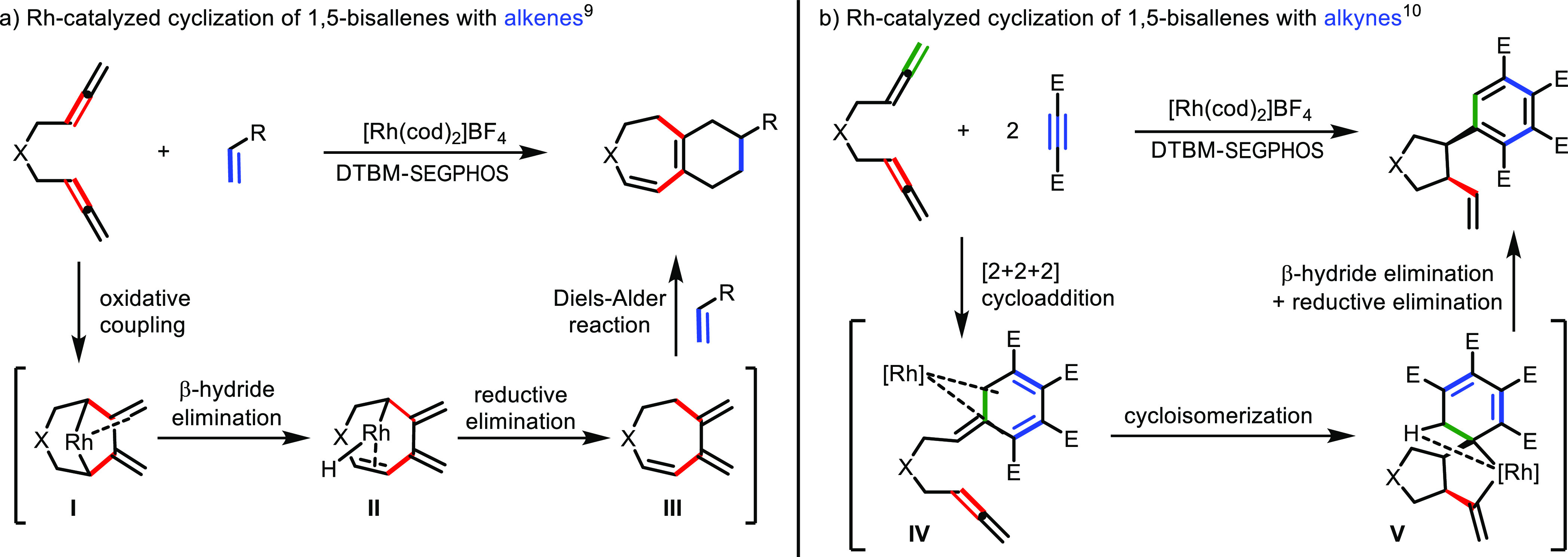
Rh(I)-Catalyzed Cyclization Reactions of 1,5-Bisallenes with Alkenes and Alkynes Performed in Our Group

Following on from our interest in allenes in cycloaddition reactions, we became interested in involving 1,5-bisallenes in the [2 + 2 + 2] cycloaddition reactions. This special class of allenes has shown a wide range of reactivities under transition-metal catalysis.^8^ However, in our initial studies, both by reaction with alkenes and alkynes, we were unable to trigger a [2 + 2 + 2] cycloaddition reaction. Instead, very interesting reactivities were observed (Scheme 2). In the reaction of 1,5-bisallenes with alkenes, dihydroazepine- and dihydrooxepine-fused ring systems were obtained in good yields.^9^ Further mechanistic study by DFT calculations showed that the reaction took place through a rhodium-catalyzed cycloisomerization/Diels–Alder cascade encompassing oxidative coupling of the rhodium to the central carbon atoms of both allenes (intermediate **I**) followed by a β-hydride elimination (intermediate **II**) and reductive elimination of the rhodium to afford a non-isolable cycloheptatriene derivative **III**, which gave a further Diels–Alder reaction with the alkene (Scheme 2a). In contrast, when an alkyne was used as a third component using the same catalytic system as before, 1,5-bisallenes reacted with two molecules of the alkyne to afford *cis*-3,4-arylvinyl pyrrolidines and cyclopentanes in a totally diastereoselective manner.^10^ Here again, DFT calculations allowed us to unveil the course of the reaction, which involves a [2 + 2 + 2] cycloaddition between two molecules of the alkyne and the terminal double bond of one of the two allenes of the bisallene (intermediate **IV**) followed by a cycloisomerization reaction involving the internal double bond of the second allene unit (intermediate **V**). Finally, a β-hydride elimination step followed by reductive elimination afforded the final product (Scheme 2b).

Still with the idea of developing a [2 + 2 + 2] cycloaddition reaction of 1,5-bisallenes, we came across the study by the Alexanian group^11^ on the cycloaddition of ene-allenes and alkenes under nickel catalysis. The study nicely showed that the selectivity can be controlled by fine-tuning the catalytic system, as the use of P(OTol)_3_ triggered a [2 + 2 + 2] cycloaddition, an alkenylative cyclization occurred when PBu_3_ was used, and finally, a [2 + 2] cycloaddition of ene-allene occurred when Xantphos was the ligand of choice. In addition, 1,5-bisallenes also follow different reaction pathways under rhodium catalysis depending on the nature of the ligands. Whereas Ma and co-workers^3d^ described a bimolecular [2 + 2 + 2] cycloaddition of the bisallene to afford steroid-type scaffolds when the Wilkinson catalyst was used (Scheme 1a), our group, using DTBM-SEGPHOS as the ligand, described a cascade process encompassing a cycloisomerization of the 1,5-bisallene followed by a selective Diels–Alder homodimerization affording spirocyclic compounds.^12^ Inspired by the results of Alexanian and our own experience, we thought of testing ligands other than bisphosphines to influence the course of the process. An alternative class of ligands are the N-heterocyclic carbenes (NHCs) that often give reactivities that are complementary to those of phosphines. Several examples of transition-metal-catalyzed [2 + 2 + 2] cycloadditions with NHC ligands are described in the literature. Among these, complexes of cobalt^13^ and, especially, nickel^14^ are the most used. Louie’s research group has conducted several studies of [2 + 2 + 2] cycloadditions involving mainly hetero-unsaturations, such as isocyanates, ketenes, nitriles, aldehydes, and ketones, using Ni–NHCs ligands.^15^ To the best of our knowledge, in the case of rhodium, there are only two previous studies reported by our group in which Rh–NHC complexes, both in homogeneous^16^ and heterogeneous versions,^17^ are tested for [2 + 2 + 2] cycloaddition reactions of alkynes.

NHCs are strong σ-donor ligands and hence form strong metal–ligand interactions that can prevent catalyst decomposition resulting in loss of ligands under reaction conditions. Moreover, their simple synthetic procedures and the ability to tune their steric and electronic properties by modifying the nitrogen or backbone substituents make them very attractive for rapid ligand screening. Rhodium(I) bearing monodentate NHCs have been successfully applied to several metal-catalyzed transformations such as hydrothiolation, hydrophosphination, cross-coupling reaction, and dimerization reactions.^18,19^ Although most NHCs are monodentate ligands, a strong interest has been shown for the past 10 years and still continues today for the chemistry of functionalized NHC carbenes in which a Lewis base moiety (often rooted on: N, P, or O atom) is attached to the strongly bonded imidazolyl ring.^20^ However, although the S-functionalized N-heterocyclic carbenes are less represented,^21^ they are also an interesting class of ligands because they can potentially provide an “on and off” dynamic chelating effect for the metal complex during a catalytic cycle. This hemilability has been particularly demonstrated for the thioether function with different metals.^22^ NHC-SR metal complexes have demonstrated their catalytic activity in several transformations such as hydrosilylation of aldehydes^22e^ and ketones,^23^ the Suzuki–Miyaura cross-coupling reaction,^24^ hydrogenation of double bonds,^25^ the click reaction,^26^ dehydrogenation of amine,^27^ and the reaction of amidation.^28^ Only two examples were reported for NHC-SR rhodium(I) complexes by the group of Poli^22^ and the group of Lassaletta,^29^ and none of them were applied to cycloaddition reactions.

In this work, a variety of NHC-imidazole ligand precursors (monodentate and bidentate) were evaluated in the Rh(I)-catalyzed [2 + 2 + 2] cycloaddition of 1,5-bisallene and alkynes. Through extensive screening of various conditions, a catalytic system with an S-functionalized NHC–Rh complex, with a catalyst loading of 5 mol %, was developed, which for the first time promoted the efficient [2 + 2 + 2] cycloaddition of 1,5-bisallene and alkynes.

## Results and Discussion

### Synthesis of S-Functionalized Imidazolium Salts

**Figure 1:**
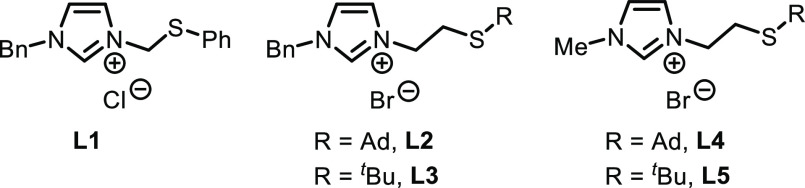
S-functionalized imidazolium salt precursors used in the cycloaddition of bisallene **1a** and alkyne **2a**.

All S-functionalized imidazolium salts **L2-5** (Figure 1) were synthesized according to our previously reported procedure by direct reaction of the 2-bromoethyl-imidazolium derivative with the corresponding sodium thiolate.^22d,27^ Considering that the size of the R thioether group can influence its ability to coordinate the rhodium center and therefore its hemilability, groups with different steric hindrances were attached to the sulfur atom in order to evaluate their influence in catalysis.

The stability of a complex formed with a bidentate ligand depends also on the size of the chelate rings. Our NHC-SR ligands **L2-5** possess a flexible ethylene organic backbone, which can generate six-membered chelate rings. To evaluate this effect, the ligand **L1** with a shorter N-arm was also prepared according to a literature procedure (Figure 1).^22b^

### Cycloaddition Catalysis

**Figure 2:**
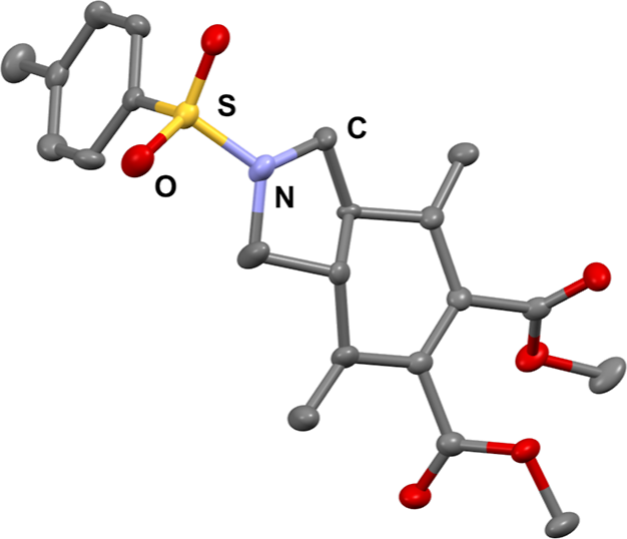
ORTEP representation of **3a-*trans*** at 50% of the probability level (CCDC 2209948).

We started our study with the reaction of *N*-tosyl-tethered bisallene **1a** and dimethylacetylenedicarboxylate (DMAD) **2a** (Table 1). S-functionalized imidazolium salts **L1-L5** (Figure 1) were tested as ligands. In the initial tests, the rhodium complexes were generated in situ by treatment of the corresponding imidazolium salt with ^*t*^BuOK as a base and the dimeric rhodium complex [Rh(cod)Cl]_2_. The reaction with **L1** afforded three products, which were identified by NMR spectroscopy. In striking contrast to our previous studies with the same substrates (Scheme 2b),^10^ a mixture of diastereoisomers *trans* and *cis***3a** in 35% yield with a ratio of about 5:1 was formed by a [2 + 2 + 2] cycloaddition reaction between the two internal double bonds of the two allenes of **1** and the alkyne (entry 1), also in contrast to the case of Ma (Scheme 1a) in which both an internal and an terminal double bond were involved in the reaction.^3d^ The molecular structure and stereochemistry of the major diastereoisomer **3a**, which was found to be *trans*, was confirmed by X-ray crystallographic analysis (Figure 2).^30^ Of note, **3a** bears structural similarities to the product obtained through the intermolecular [2 + 2 + 2] reaction of two allenes and one alkyne described by Tanaka and co-workers (Scheme 1b).^4^ A second product **4** was also obtained, although with a very low yield. This was formed by our previously described process^9^ based on a cycloisomerization/Diels–Alder cascade reaction (see for instance Scheme 2a), in which in this case the dienophile is an alkyne (entry 1, Table 1). Interestingly, the same reaction with the monodentate 1,3-bis(2,6-diisopropylphenyl)imidazolium chloride (IPr·HCl) provided lower selectivity and reactivity (entry 2, Table 1). In addition, the monodentate IMes and a bidentate OH-functionalized NHC or a bis-NHC have been less successful (see Scheme S2 in Supporting Information for details).

**Table 1:**
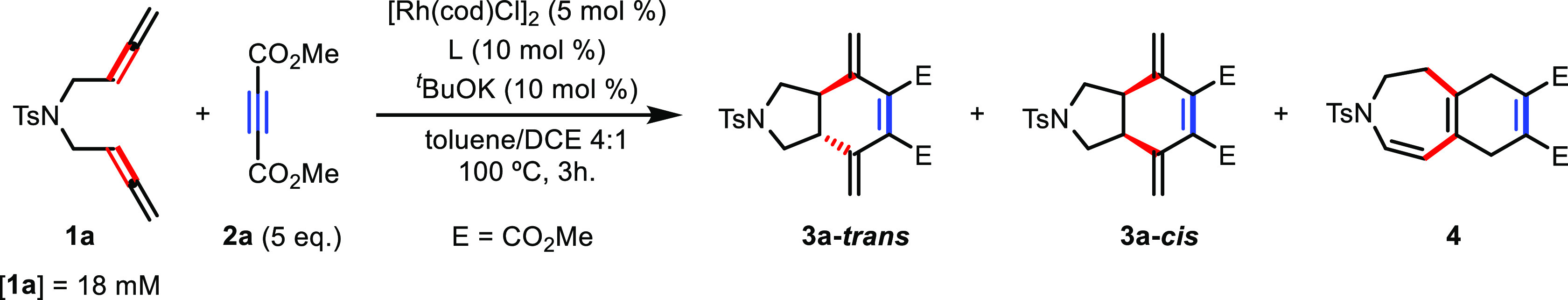
Optimization of the Rhodium(I)–NHC-Catalyzed [2 + 2 + 2] Cycloaddition of **1a** and **2a**

entry	ligand	variation of reaction conditions	yield (%) 3 (*trans*/*cis*)/4^a^
1	L1		35 (83:17)/4
2	IPr·HCl		23 (78:22)/13
3	L2		51 (86:14)/10
4	L2	80 °C, 2 h	66 (86:14)/13
5	L3	80 °C, 2 h	61 (87:13)/10
6	L4	80 °C, 2 h	74 (89:11)/8
7	L5	80 °C, 2 h	61 (90:10)/7
8	L4	80 °C, 2 h, toluene	73 (93:7)/4
9	L4	80 °C, 2 h, toluene, [**1a**] = 36 mM	76 (93:7)/0
10	L4	80 °C, 2 h, toluene, **2a** (2 equiv)	66 (91:9)/8
11	L4	80 °C, 2 h, toluene, **2a** (10 equiv)	77 (92:8)/0
12	RhL4	80 °C, 2 h, toluene, RhL4 (1 mol %) [**1a**] = 36 mM	31 (91:9)/0
13	RhL4	80 °C, 2 h, toluene, RhL4 (2.5 mol %) [**1a**] = 36 mM	55 (91:9)/0
**14**	**RhL4**	**80 °C, 2 h, toluene, RhL4 (5 mol %) [1a]** = **36 mM**	82 (92:8)/0
15	RhL4	80 °C, 2 h, toluene, RhL4 (10 mol %), [**1a**] = 36 mM	81 (92:8)/0

a Yields and ratios calculated by ^1^H NMR from the reaction crude.

Encouraged by the selective formation of [2 + 2 + 2] cycloadducts **3** using this new catalytic system, we explored the other NHC-SR ligands and tested different reaction conditions to improve the efficiency and selectivity of the cycloaddition. Using imidazolium salt **L2** with longer N-wing chain the yield of **3** improved to 51%, but the selectivity decreased (entry 3, Table 1). As a series of unidentified compounds were observed in the crude mixture in these experiments, the reaction was performed again by decreasing the temperature to 80 °C and the yield of **3** improved to 66% (entry 4, Table 1). Under these conditions, we proceeded to test the less sterically demanding NHC-SR ligands **L3-5** (entries 5–7, Table 1). The use of ligand **L4** provided the best yield and selectivity (entry 6, Table 1) and was thus selected for further tests. Performing the reaction in the absence of DCE led to an increase in the *trans*/*cis* ratio and a decrease in the formation of **4** (entry 8, Table 1). To our delight, formation of byproduct **4** could be fully suppressed by increasing the concentration of **1a** from 18 to 36 mM (entry 9, Table 1). Increasing and decreasing the amount of **2a** (2 equiv and 10 equiv, respectively) did not improve the results (entries 10–11, Table 1). Once the ligand was optimized, we proceeded to prepare a preformed and well-defined Rh complex **RhL4**, which we used as a catalyst.

**Scheme 3:**
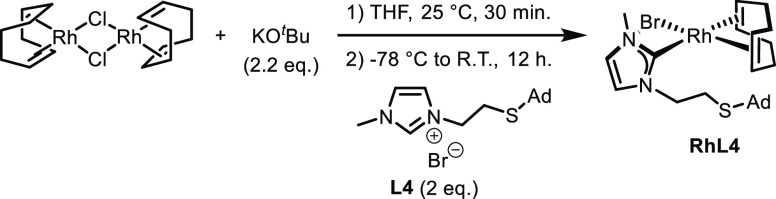
Synthesis of Rhodium Complex **RhL4**

The NHC–rhodium(I) complex **RhL4** was prepared in a one-step process by the reaction of 2 equiv of imidazolium salt **L4** with 1 equiv of [Rh(cod)Cl]_2_ in the presence of 2.2 equiv of ^*t*^BuOK in THF (Scheme 3) following a procedure previously described by us.^31^ The corresponding N-heterocyclic carbene complex **RhL4** was obtained in a high yield (above 95%) as a yellow shiny solid, which was fully characterized. In the presence of the halogen onto the metal center, no direct evidence of the coordination of the sulfur atom to the metal center was noticed by ^1^H NMR.^32^

**Figure 3:**
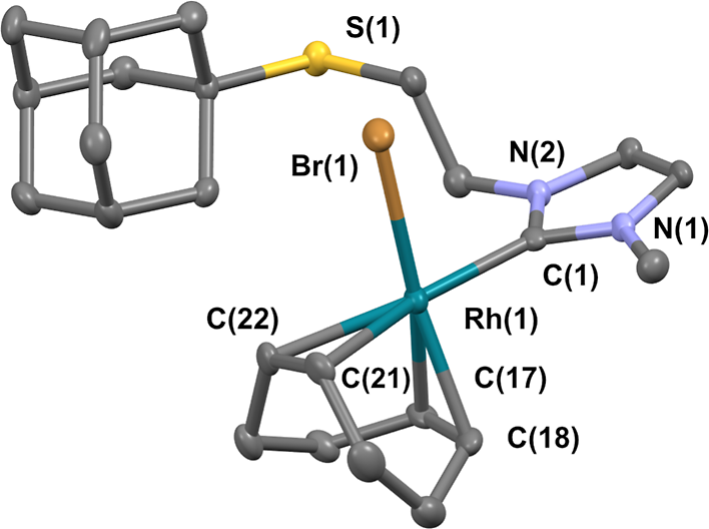
ORTEP representation of **RhL4** at 50% of the probability level (CCDC: 2213801). Selected bond lengths [Å] and angles [deg]: C(1)-Rh(1), 2.033(4); Rh(1)-Br(1), 2.5026(6); Rh(1)-C(17), 2.102(4); Rh(1)-C(18), 2.109(4); Rh(1)-C(21), 2.207(4); Rh(1)-C(22), 2.227(5); C(1)-Rh(1)-Br(1), 86.5(1); C(21)-Rh(1)-Br(1), 91.8(1); C(22)-Rh(1)-Br(1), 95.4(1); C(18)-Rh(1)-C(1), 94.2(2); C(17)-Rh(1)-C(1), 90.6(2).

The molecular structure of **RhL4** was confirmed by X-ray diffraction studies (Figure 3).^30^ The rhodium–carbene bond distance, 2.033(4) Å, is within the range reported for [RhCl(cod)(imidazol-2-ylidene)] complexes.^32,33^ The Rh–C bonds located in a relative *trans* position to the carbene are significantly elongated (mean Rh–C 2.217 Å) compared to those in *trans* to the bromide ligand (mean Rh–C 2.1055 Å) due to the strong *trans* influence of the NHC ligand. The imidazole-2-ylidene ring is almost perpendicular to the coordination plane of the rhodium center (dihedral angle 88.64°), and this geometry is consistent with the ^1^H NMR spectra.

Different catalytic amounts of **RhL4** were tested, from 1 to 10 mol % (entries 12–15, Table 1). Quantities below 5 mol % reduced the yield of **3a** (entries 12–13, Table 1) and the use of 10 mol % of **RhL4** (entry 15, Table 1) did not improve the results compared to the use of 5 mol % (entry 14, Table 1). It should be noted that in no case the formation of byproduct **4** was observed. Therefore, the optimal reaction conditions for further studies were defined as **1a** ([**1a**] = 36 mM), **2a** (5 equiv), **RhL4** (5 mol %) in toluene at 80 °C for 2 h (entry 14, Table 1). In addition, two blank tests were performed. The reaction was first carried out in the presence of the rhodium dimer [Rh(cod)Cl]_2_ excluding the NHC ligand and second in the absence of both the transition metal and the ligand. The reaction did not work in either case, and only the two starting products **1a** and **2a** were recovered.

It should be noted that bisphosphine ligands such as BINAP, Tol-BINAP, BIPHEP, DPEphos, Xantphos, and XPhos were not able to provide a defined product upon reaction of **1a** and **2a** as investigated in a previous study of our group.^10^

**Scheme 4:**
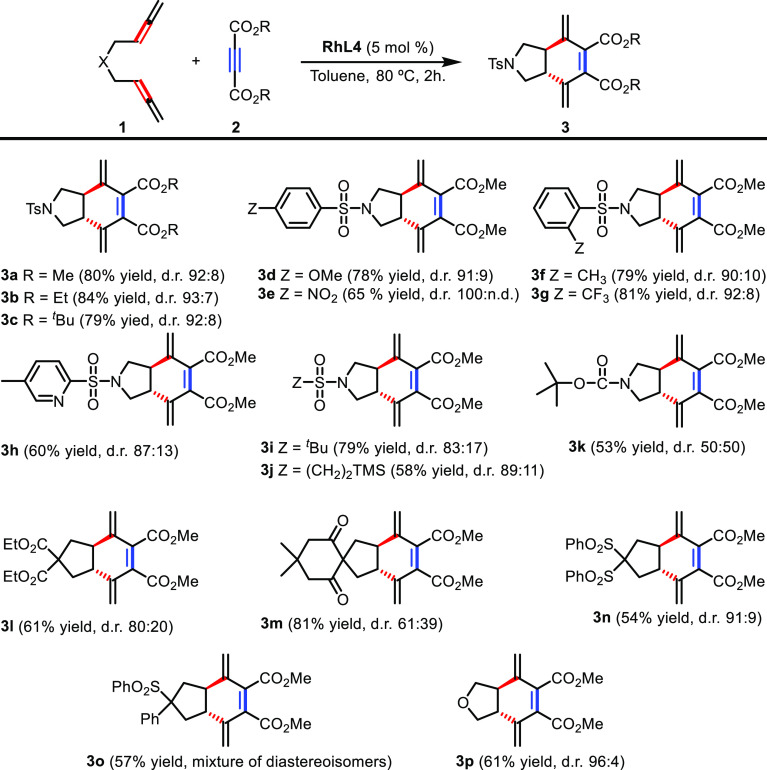
Scope of the [2 + 2 + 2] Cycloaddition Reaction of Bisallenes **1** and Alkynes **2**

The scope of the reaction was then evaluated (Scheme 4). Methyl, ethyl, and *tert*-butyl acetylenedicarboxylates **2a**, **2b**, and **2c** were tested in the cycloaddition affording excellent yields and high *trans*/*cis* ratios^34^ of the corresponding cycloadducts **3a–3c**. However, terminal alkynes, such as monoacetylenecarboxylates and phenylacetylene analogues, did not react under these conditions. The nature of the substituents at the phenyl ring of the sulfonamide tether in bisallene **1** was then explored. The reaction proceeded efficiently with both electron-donating (**3d**) and electron-withdrawing groups (**3e**), as well as with substituents at the ortho position of the phenyl ring (**3f**, **3g**). A bisallene bearing the 5-methyl-2-pyridinesulfonyl group provided **3h** in a 60% yield, indicating that the presence of a potentially coordinating nitrogen atom did not poison the catalyst. Sulfonamide tethers with aliphatic substitution (*tert*-butyl and trimethylsilylethyl) were also efficient, delivering cycloadducts **3i** and **3j** in 79 and 58% yields, respectively. Changing the sulfonamide protecting group of the nitrogen tether to a carbamate (*N*-Boc bisallene), the reaction took place, although cycloadduct **3k** was obtained in a moderate yield and with low diastereoselectivity. Bisallenes with a carbonyl group attached to the quaternary carbon atom of the tether also participated in the cycloaddition, affording **3l** and **3m** with 61 and 81% yields, respectively. When the carbonyl group was substituted for arylsulfonyl groups, lower yields of the cycloadducts **3n** and **3o** were obtained, but the diastereoisomeric ratios were better. In the case of **3o**, an inseparable mixture of diastereoisomers was obtained due to the chiral center in the tether making it difficult to determine the diastereoisomeric ratio. Finally, oxygen-tethered bisallene participated in the process affording cycloadduct **3p** with a 61% yield and an excellent diastereoisomeric ratio.

### Computational Analysis

**Figure 4:**
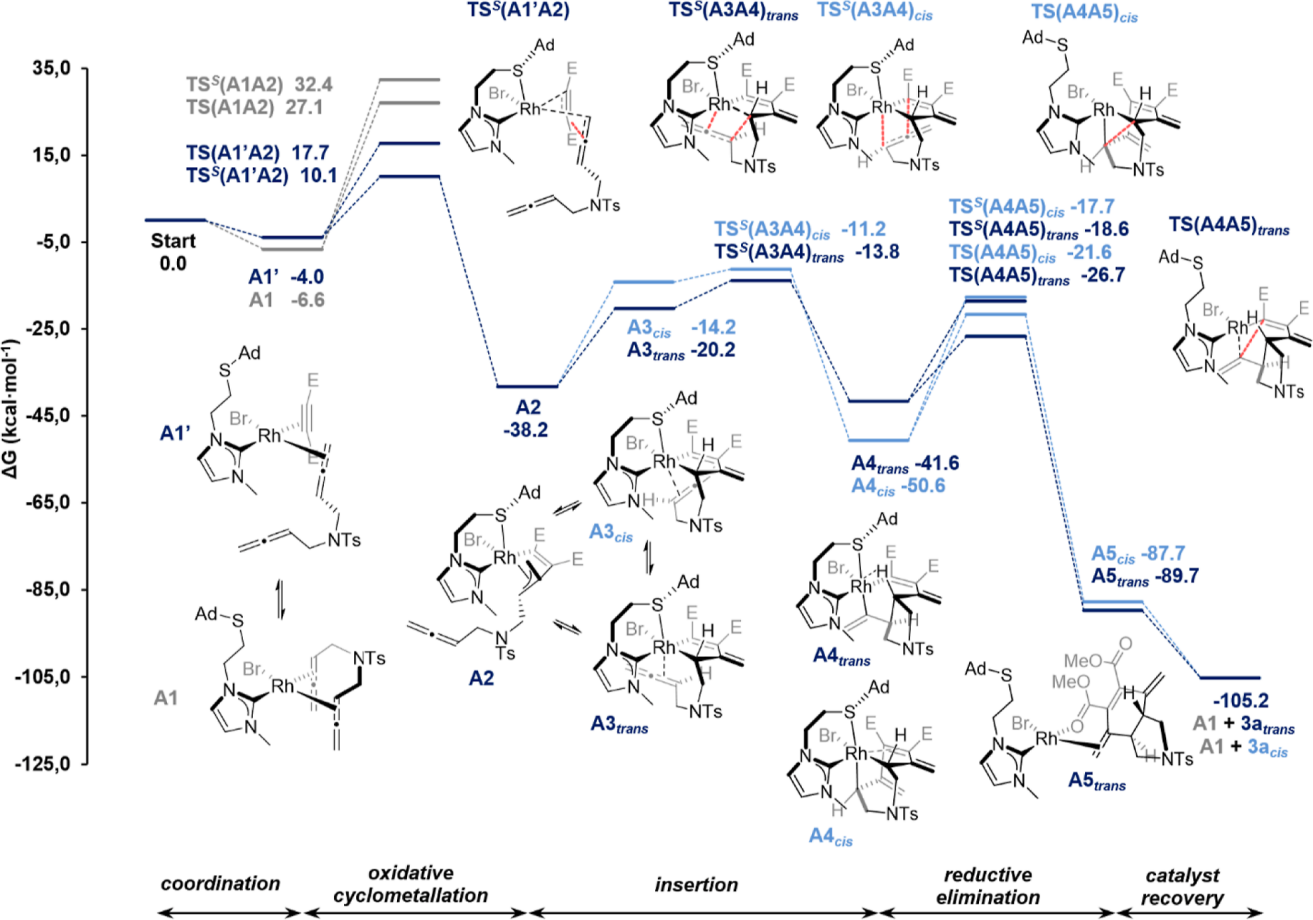
Gibbs energy profile (in kcal·mol^–1^) for the cycloaddition of 1,5-bisallene **1a** and dimethylacetylenedicarboxylate **2a** leading to **3a** (E = CO_2_Me).

Intrigued by the role of the hemilabile NHC-SR ligand in the chemoselectivity of the rhodium-catalyzed cycloaddition of 1,5-bisallenes and alkynes, we performed DFT calculations on the entire reaction. The Gibbs energy profile computed at 353.15 K and 1 atm with the ωB97X-D/cc-pVTZ-PP/SMD(toluene)//B3LYP-D3/cc-pVDZ-PP method is depicted in Figure 4, and the molecular structures of all intermediates and transition states (TSs) are available in the Supporting Information (see Supporting Information for a complete description of the computational methods and Table S1 for a justification of the density functional employed).

The reaction starts with the coordination equilibrium of 1,5-bisallene and DMAD with the rhodium catalyst to give Rh(I) 16 e^–^ square planar complexes **A1** and **A1′** (Figure 4) (see Figure S1 for the whole set of coordination complexes). The coordination of the two internal double bonds of the 1,5-bisallene (**A1**) is the most exergonic process, releasing 6.6 kcal·mol^–1^. For the upcoming oxidative cyclometallation, all possible orientations have been evaluated along with the dynamic chelating effect of the sulfur (see Figures S2 and S3), resulting in two possible TSs for each orientation: one with the sulfur chelating the rhodium (TSs superindexed with *S* in Figure 4) and the other without such a chelation.^35^ Since **A1** and **A1′** (and the rest of the possible coordination complexes) are in equilibrium, according to the Curtin–Hammett principle,^36^ the major rhodacyclopentene or rhodacyclopentane intermediate formed is the one generated through the lowest in energy TS, in this case **TS**^***S***^**(A1′A2)**. This TS involves an oxidative cyclometallation of the central carbon of one of the allenes of the 1,5-bisallene **1a** with DMAD **2a** with the sulfur chelating the rhodium. Formation of rhodacyclopentene intermediate **A2** takes place with an affordable barrier of 16.7 kcal·mol^–1^ [**A1** to **TS**^***S***^**(A1′A2)**, being 7.6 kcal·mol^–1^ lower than its non-chelating counterpart (**TS(A1′A2)**, Δ*G*^‡^ = 24.3 kcal·mol^–1^]. This step **A1 ⇌ A1′ → A2** is exergonic by 31.6 kcal·mol^–1^. Alternative ways to generate **A2** through other coordination complexes including **A1** have higher energy barriers (see Figures 4 and S2 and S3 in Supporting Information). For this path, the sulfur-assisted TS [**TS**^***S***^**(A1′A2)**] is lower in energy than its analogue in which the sulfur is not coordinated to the rhodium [**TS(A1′A2)**] due to the *S* electron-donating character, which adds electronic density to the rhodium and facilitates its oxidation [NPA charges on Rh are −0.101 e in **TS(A1′A2)** and −0.443 e in **TS**^***S***^**(A1′A2)**]. However, in the oxidative cyclometallation from **A1**, coordination of *S* leads to a pyramidalization of the rest of the ligands coordinated to Rh. Pyramidalization brings the ligands closer to one another and, consequently, the two coordinated double bonds in the 1,5-bisallene become perpendicularly arranged to one another. This perpendicular arrangement is destabilizing^37^ and the trend is inverted. As a result, the non-chelated **TS(A1A2)** is lower in energy than **TS**^***S***^**(A1A2)** by 5.3 kcal·mol^–1^. **A2** is a Rh(III) 18 e^–^ complex and exhibits an octahedral geometry in which the three carbons from the reacted allene are η^3^-coordinated to the rhodium (*d*_Rh–C_ = 2.217, 2.129, and 2.218 Å). This type of π-allyl metallacycle intermediates have previously been postulated in cycloaddition reactions.^38^ From this point, **A2** needs to rearrange to set a coordination position free for the second allene unit, giving either **A3**_***trans***_ or **A3**_***cis***_ at the cost of 18.0 and 24.0 kcal·mol^–1^, respectively. For the formation of the *trans*-fused rhodabicyclo intermediate **A4**_***trans***_ through **A3**_***trans***_, the insertion (via the Schore mechanism^39^) of the internal double bond of the second allene takes place in the Rh–C sp^3^ bond through **TS**^***S***^**(A3A4)**_***trans***_. This process **A2 → A4**_***trans***_ has a total Gibbs energy barrier of 24.4 kcal·mol^–1^ and is exergonic by 3.4 kcal·mol^–1^. In contrast, for the formation of **A4**_***cis***_, the insertion is found to occur in the Rh–C sp^2^ bond, surpassing a Gibbs energy barrier of 27.0 kcal·mol^–1^ [**TS**^***S***^**(A3A4)**_***cis***_] and releasing 12.4 kcal·mol^–1^. The coordination of a second DMAD unit to **A2**, which would lead to our previously reported *cis*-3,4-arylvinyl pyrrolidine derivative (Scheme 2b),^10^ was also considered. However, it was found to be disfavored at the experimental concentration of DMAD (see Figure S6 in Supporting Information).

For the final reductive elimination step, the *S*-adamantyl functionality is dissociated from the rhodium center to remove electronic density, drastically reducing the Gibbs energy barriers by 8.1 kcal·mol^–1^ [**TS(A4A5)**_***trans***_ vs **TS**^***S***^**(A4A5)**_***trans***_] and 3.9 kcal·mol^–1^ [**TS(A4A5)**_***cis***_ vs **TS**^***S***^**(A4A5)**_***cis***_]. Finally, ligand exchange from **A5**_***trans***_ and **A5**_***cis***_ releases **3a-*trans*** and **3a-*cis*** and gives **A1** to restart the catalytic cycle. The formation of byproduct **4** (Table 1) was also evaluated computationally, and the results account for its formation in minor quantities at low concentrations of **2a** (see Scheme S6 and Figures S4 and S5 in Supporting Information for the complete discussion).

In summary, the reaction follows the typical [2 + 2 + 2] cycloaddition mechanism^40^ and has an overall reaction energy of −98.6 kcal·mol^–1^ (Δ*G* = *G*_**3a**_ – [*G*_**1a**_ + *G*_**2a**_]), almost identical for both diastereoisomers. For **3a-*trans***, the energetic span between the turnover-frequency-determining intermediate (TDI, **A2**) and the turnover-frequency-determining TS (TDTS, **TS(A3A4)**_***trans***_) is 24.4 kcal·mol^–1^, and for **3a-*cis***, the energetic span between TDI (**A4**_***cis***_) and the TDTS [**TS(A4A5)**_***cis***_] is 29.0 kcal·mol^–1^.^41^ The allene–allene oxidative cyclometallation is found to be much higher in energy than the allene–alkyne oxidative cyclometallation (ΔΔ*G*^‡^ = 16.0 kcal·mol^–1^), indicating that the ring fusion stereochemistry comes from the latter insertion^6b^ of the second allene, in which the insertion leading to the *trans* isomer [**TS(A3A4)**_***trans***_] is preferred over the *cis* [**TS(A3A4)**_***cis***_] by 2.6 kcal·mol^–1^. This difference in energy is translated into a 98:2 *trans*/*cis* ratio using the Eyring equation, in perfect agreement with the experimental data.

In conclusion, the hemilability of NHC-SR ligands enabled an efficient rhodium-catalyzed [2 + 2 + 2] cycloaddition of 1,5-bisallenes and alkynes. A number of N-, C-, and O-tethered 1,5-bisallenes as well as variously substituted alkynes were successfully used in the reaction. The methodology developed gives access to bicyclic 3,6-dimethylenecyclohex-1-ene derivatives. Importantly, the exocyclic alkenes in the product scaffold provide ample opportunities of synthetic manipulation to build more complex molecules. A mechanistic investigation by means of DFT calculations has been carried out to unravel that a canonical [2 + 2 + 2] reaction manifold accounts for the observed transformation and that the hemilabile character of the sulfur in the ligand precisely modulates the electron density in key intermediates and facilitates the overall transformation. This hemilability in the catalytic system is expected to be useful for further development of demanding cycloaddition reactions.

## Supporting Information Available

Experimental procedures, compound characterization data including NMR spectra for all new compounds, and computational data (PDF)
Crystallographic data of 3a (CIF)
Crystallographic data of RhL4 (CIF)
Crystallographic data of [RhL4][PF_6_] (CIF)

## Supplementary Material

cs2c05790_si_001.pdf

cs2c05790_si_002.cif

cs2c05790_si_003.cif

cs2c05790_si_004.cif
