# Supplementary material for: Rh(I) Complexes with Hemilabile Thioether-Functionalized NHC Ligands as Catalysts for [2 + 2 + 2] Cycloaddition of 1,5-Bisallenes and Alkynes
Source: ACS Catal. 2023 Feb 17;13(5):3201–10. doi: 10.1021/acscatal.2c05790 (PMC9990073; doi:10.1021/acscatal.2c05790)
Supplement: Supplementary file 1 — cs2c05790_si_001.pdf [file cs2c05790_si_001.pdf]

SUPPORTING INFORMATION

Rh(I) complexes with hemilabile thioether-functionalized NHC ligands as catalysts for [2+2+2] cycloaddition of 1,5-bisallenes and alkynes

Jordi Vila,[a] Miquel Solà,[a] Thierry Achard,[b] Stephane Bellemin-Laponnaz,[b,] Anna Pla-Quintana,[a] Anna Roglans[a,*]*

[a] Institut de Química Computacional i Catàlisi (IQCC) and Departament de Química, Universitat de Girona (UdG), Facultat de Ciències, C/ Maria Aurèlia Capmany, 69, 17003-Girona, Catalunya, Spain.

[b] Institut de Physique et Chimie des Matériaux de Strasbourg, CNRS-Université de, Strasbourg, UMR7504 23 rue du Loess BP 43, 67034 Strasbourg, France.

Corresponding authors e-mail address: anna.roglans@udg.edu; bellemin@unistra.fr;

TABLE OF CONTENTS

- General materials and methods S4
- Scheme S1. Synthesis of bisallene 1d S6
- Scheme S2. Cycloaddition reaction using NHC ligands L6, L7, L8, and L9. .... S7
- Synthesis of imidazolium salts L1-L5 .... S7

  Scheme S3. General procedure for the synthesis of L1-L5 .... S7

  General procedure for the synthesis of L2-L5 .... S8
- Synthesis of Rhodium complex RhL4 .... S8

  Scheme S4. Procedure for the synthesis of complex RhL4 .... S8
- Synthesis of compounds 3a-p S9

  Scheme S5. General procedure GP1 for the synthesis of products 3a-p S9
- Computational methods S17

  Figure S1. Gibbs energy formation of the complexes evaluated, resulting from the coordination of 1,5-bisallene 1a and DMAD 2a into RhL4. Carboxylates of DMAD are omitted for clarity. S18

  Figure S2. Gibbs energy barriers of the paths evaluated for the oxidative cyclometallation in the formation of 3a without the sulfur chelating the rhodium. S19

  Figure S3. Gibbs energy barriers of the paths evaluated for the oxidative cyclometallation in the formation of 3a with the sulfur chelating the rhodium. S20

  Scheme S6. Alternative reaction paths evaluated leading to 4 and to our previously reported *cis*-3,4-arylvinyl pyrrolidine derivative P1 through IV. S21

  Figure S4. Energy profile of the reaction including the alternative paths leading to 4 and to our previously reported *cis*-3,4-arylvinyl pyrrolidine derivative P1. Calculated at 353.15 K and 1 atm at the ωB97X-D/cc-pVTZ-PP(SMD,Solvent=Toluene) // B3LYP-D3/cc-pVDZ-PP theory level. S22

  Figure S5. Graphical representation of the I:A2 production ratio versus the concentration of DMAD 2a. S23

  Figure S6. Graphical representation of the $A4_{cis}$:$A4_{yne}$ production ratio versus the concentration of DMAD 2a. S24

  Table S1. Results obtained with different density functionals of the Gibbs energy difference (ΔΔG) between the reaction barriers leading from A2 to $A4_{trans}$ and to $A4_{yne}$ and the ratio of reaction rates ($v_{A4trans}/v_{A4yne}$) obtained from the ΔΔG values using the transition state theory. S25

  Computational data S27
- ^{1}H and ^{13}C NMR spectra S28

  Imidazolium salt L4 .... S28

  Rhodium-NHC complex RhL4 .... S31

  Product 3a S37

  Product 3b S44

  Product 3c S47

  Product 3d S50

  Product 3e S53

  Product 3f S56

  Product 3g S59

  Product 3h S63

Product 3i S66
Product 3j S69
Product 3k S72
Product 3l S77
Product 3m S82
Product 3n S87
Product 3o S90
Product 3p S96
- Crystal structure of compound 3a-*trans* S99
  Figure S7. ORTEP representation of 3a-*trans* with probability level of 50% S99
  Table S2. Sample and crystal data for 3a-*trans*..S100
  Table S3. Data collection and structure refinement for 3a-*trans*. S100
  Table S4. Atomic coordinates and equivalent isotropic atomic displacement parameters (Å2) for 3a-*trans*..S101
- Crystal structure of compound RhL4 ....S102
  Figure S8. ORTEP representation of RhL4 with probability level of 50%, Hydrogen are omitted for clarity. S103
  Table S5. Sample and crystal data for RhL4. ....S103
  Table S6. Data collection and structure refinement for RhL4...S103
  Table S7. Atomic coordinates (x 10^4) and equivalent isotropic atomic displacement parameters (Å2 x 10^3) for RhL4. ....S104
- Crystal structure of cationic compound [RhL4][PF_6]..S105
  Figure S9. ORTEP representation of [RhL4][PF_6] with probability level of 50%, Hydrogen are omitted for clarity...S106
  Table S8. Sample and crystal data for [RhL4][PF_6]. S107
  Table S9. Data collection and structure refinement for [RhL4][PF_6]. S107
  Table S10. Atomic coordinates (x 10^4) and equivalent isotropic atomic displacement parameters (Å2 x 10^3) for [RhL4][PF_6]. S108
- References S110

General materials and methods

Unless otherwise noted, materials were obtained from commercial suppliers and used without further purification. Bisallenes **1** were prepared from the corresponding bisalkynes using Crabbé homologation reaction. Experimental procedures and full characterization have been described previously by us: bisallenes **1a**[1] (X = NTs), **1b**[1] (X = *p*-MeOPhSO_2N), **1c**[1] (X = *p*-NO_2PhSO_2N), **1e**[1] (X = *o*-CF_3PhSO_2N), **1f**[1] (X = 5-methyl-2-pyridinesulfonamidil), **1g**[1] (X = ^{t}BuSO_2N), **1h**[1] (X = TMS$CH_2CH_2SO_2$N), **1i**[2] (X = BocN), **1j**[1] (X = C(CO_2Et)$_2$), 1**k**[2] (X = 5,5-dimethylcyclohexane-1,3-dione), **1l**[2] (X = C(SO_2Ph)$_2$), **1m**[1] (X = C(Ph)(SO_2Ph)), 1**n**[3] (X = O). Bisallene **1d** (X = *o*-CH_3PhSO_2N) was prepared for the first time from the corresponding bisalkyne using a Crabbé homologation reaction (Scheme S1). **SL1**[4], **SL2**[5] and **L1**[6] (Scheme S3) were prepared according to the procedures reported in the literature.

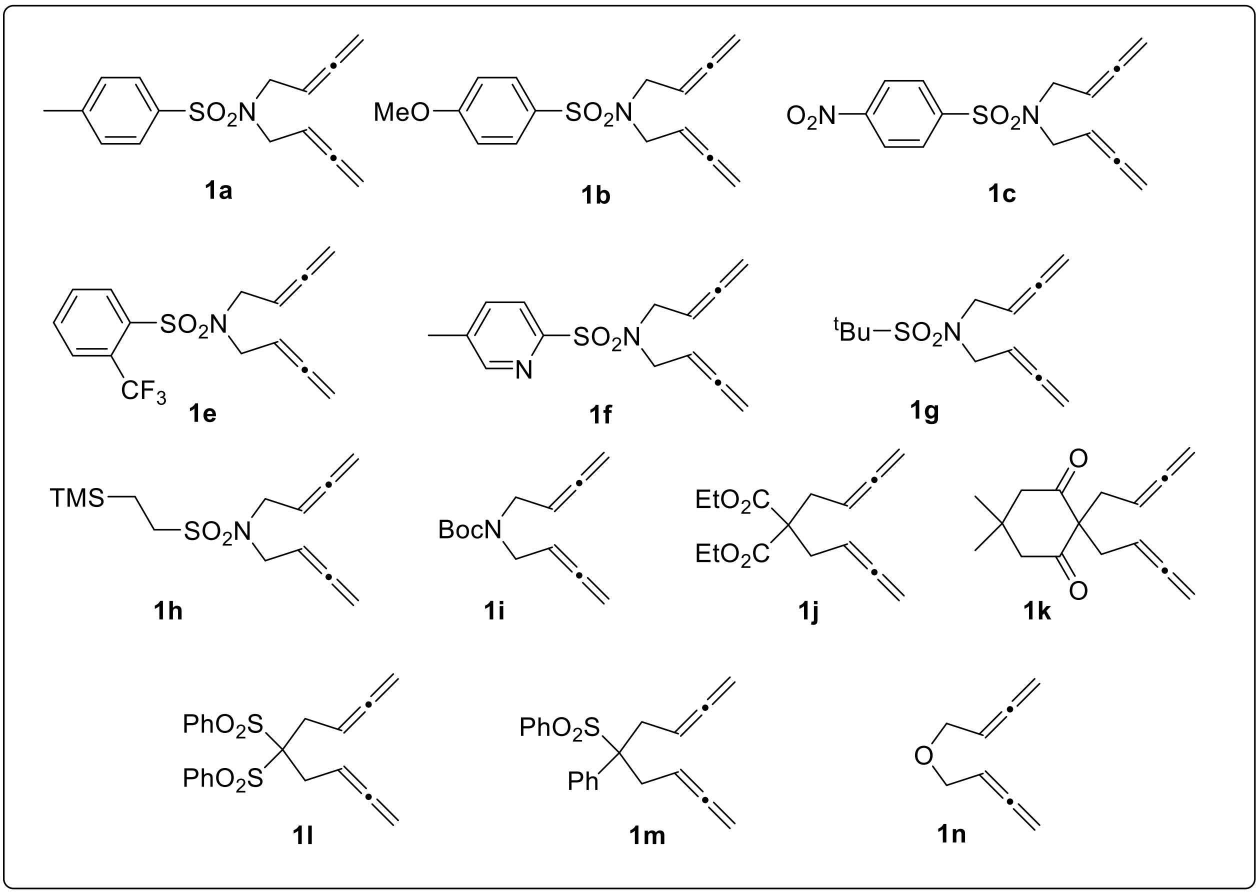

Anhydrous solvents were dried under nitrogen by passing through solvent purification columns (MBraun, SPS-800) or obtained from commercial suppliers. Reaction progress during the preparation of all compounds was monitored using thin layer chromatography on Macherey-Nagel Xtra SIL G/UV254 silica gel plates. Solvents were removed under reduced pressure with a rotary evaporator. Reaction mixtures were chromatographed on silica gel using an automated purification instrument Interchim PuriFlash XS 520 Plus equipped with a quaternary gradient pump (up to 300 ml/min, 20 bar) and an UV-Vis 200-800 nm díode array detector. All ^{1}H and ^{13}C NMR spectra were recorded on a Bruker ASCEND 400 spectrometer equipped with a 5 mm BBFO probe using $CDCl_3$ as a deuterated solvent. Chemical shifts for ^{1}H and ^{13}C NMR are reported in ppm (δ) relative to

residual solvent signals (7.26 ppm for ^{1}H, 77,16 ppm for ^{13}C). Coupling constants are given in hertz (Hz). ^{1}H and ^{13}C NMR signals were assigned based on 2D-NMR experiments (HSQC, HMBC, COSY and NOESY). Electrospray ionisation high-resolution mass spectrometry was performed using a Bruker microTOF-Q II instrument and was operated in the positive ESI(+) ion mode. IR spectra were recorded on an Agilent Cary 630 FT-IR spectrometer equipped with an ATR sampling accessory. The X-ray intensity data were measured on a 'Bruker D8 QUEST ECO' three-circle diffractometer system equipped with a Ceramic X-ray tube (Mo Kα, λ = 0.71076 Å) and a doubly curved silicon crystal Bruker Triumph monochromator. Melting points were measured in a SMP10 apparatus from Stuart without any correction.

Scheme S1. Synthesis of bisallene 1d

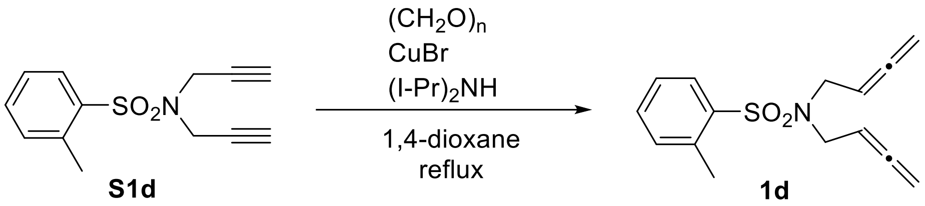

In a round-bottom flask equipped with a reflux condenser and a magnetic stirrer, a suspension of the diyne **S1d** (1.51 g, 6.11 mmol, 1 equiv.), paraformaldehyde (0.91 g, 30.6 mmol, 5 equiv.) and CuBr (0.88 g, 6.13 mmol, 1 equiv.) in 1,4-dioxane (34 mL) was stirred and heated to reflux. Diisopropylamine (3.4 mL, 0.24 mmol, 4 equiv.) was then added and the resulting mixture was stirred at reflux for 2 h until completion (TLC monitoring). The reaction mixture was allowed to cool down to room temperature, filtered through a Celite pad and concentrated under reduced pressure. The resulting brown oil was mixed with Et_2O (70 mL) and water (30 mL) and the mixture was acidified to pH = 2 with HCl 6M. The Et_2O/water layers were decanted from solid residues, the Et_2O layer was separated, and the water layer was extracted with Et_2O. The combined organic extracts were washed with water until neutral pH, washed then with brine, dried over anhydrous Na_2SO_4 and concentrated under reduced pressure. The resulting crude was purified by column chromatography (SiO2, 40–60 µm) using mixtures of hexane/EtOAc as the eluent (100:0 to 80:20 v/v) to afford bisallene **1d** (0.42 g, 25 % yield) as a colorless oil.

MW ($C_{15}H_{17}NO_2S$): 275.37 g/mol. **Rf**: 0.56 (Hexane/EtOAc 8:2). **IR (ATR)** ν **(cm^{-1})**: 1951, 1319, 1154. **^{1}H NMR ($CDCl_3$, 400 MHz): δ_H** 7.95 (dd, 1H, $^3J_{ortho}$ = 8.2 Hz, $^4J_{meta}$ = 1.4 Hz), 7.44 (td, 1H $^3J_{ortho}$ = 7.5 Hz, $^4J_{meta}$ = 1.4 Hz), 7.33 – 7.28 (m, 2H), 5.00 (p, 2H, ^3J = ^4J = 6.9 Hz), 4.73 (dt, 4H, ^4J = 6.9 Hz, ^5J = 2.4 Hz), 3.92 (dt, 4H, ^3J = 6.9 Hz, ^5J = 2.4 Hz), 2.60 (s, 3H). **^{13}C{1H} NMR ($CDCl_3$, 101 MHz): δ_C** 210.0, 138.3, 137.8, 132.9, 132.8, 130.0, 126.2, 85.8, 76.3, 45.2, 20.6. **HRMS (ESI) m/z:** $[M+Na]^+$ Calcd. For $C_{15}H_{17}NO_2SNa$ 298.0872; Found 298.0878.

Scheme S2. Cycloaddition reaction using NHC ligands L6, L7, L8, and L9.

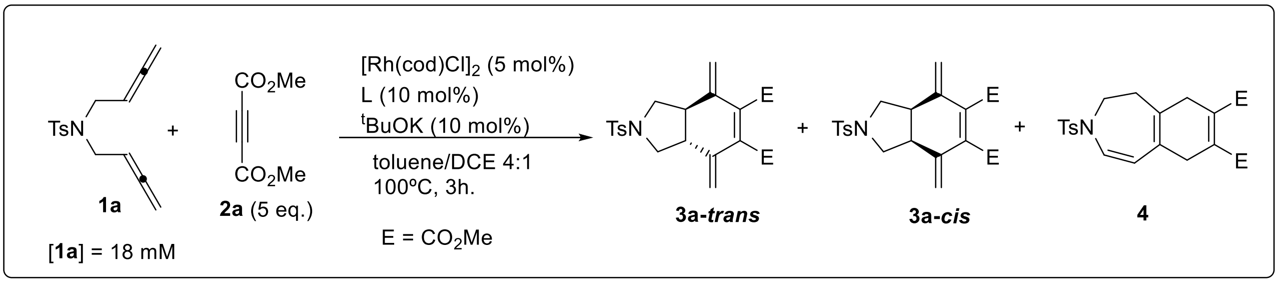

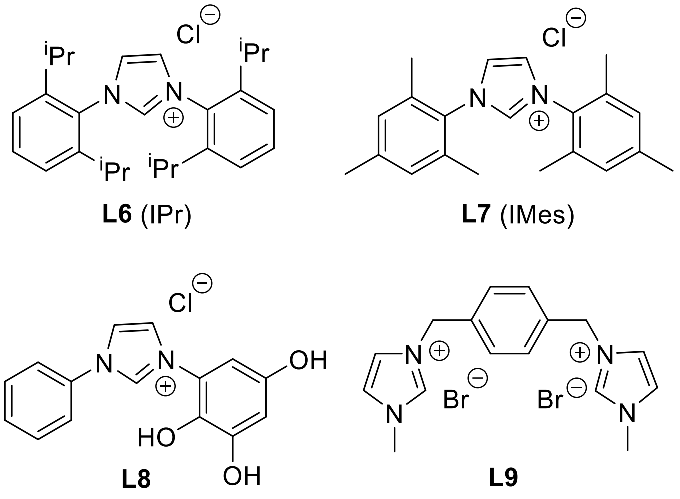

Entry	NHC precursor	Yield (%) **3a** (*trans*:*cis*) / **4**
1	**L6** (IPr)	23 (78:22) / 13
2	**L7** (IMes)	24 (75:25) / 5
3	**L8**	18 (55:45) / 9
4	**L9**	19 (47:53) / 9

Synthesis of imidazolium salts L1-L5

Scheme S3. General procedure for the synthesis of L1-L5

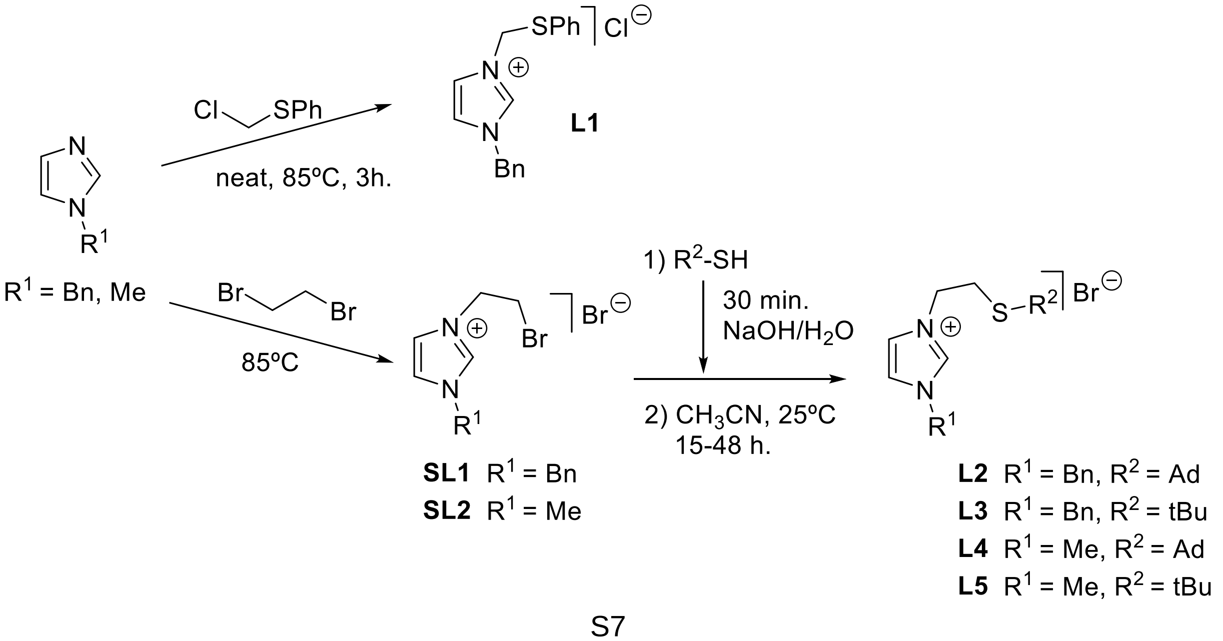

General procedure for the synthesis of L2-L5

The corresponding thiol R^2-SH (1.5 equiv.) was dissolved in dry acetonitrile. Sodium hydroxide (1.5 equiv.) in water was added, and the resulting mixture was stirred for 30 min. at room temperature. The bromine precursor **SL1** or **SL2** (1 equiv.) was then added, and the resulting mixture was stirred at room temperature for 15-48 h. After this time, the solvent was removed under reduced pressure. The solid residue was suspended in dichloromethane and filtered over Celite. The resulting crude was purified by column chromatography (SiO2, 40–60 µm) using mixtures of DCM/MeOH as the eluent (100:0 to 90:10 v/v) to afford **L2-L5**. See reference 7 for spectroscopic data of imidazolium salts **L2**, **L3**, and **L5**.

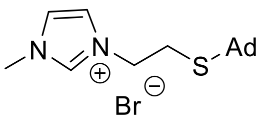

L4

Imidazolium salt **L4** (91% yield) was obtained from **SL2** (1 g, 3.7 mmol, 1 equiv.), adamantane-1-thiol (0.935 g, 5.56 mmol, 1.5 equiv.) and NaOH (0.223 g, 5.56 mmol, 1.5 equiv.) following the general procedure.

MW ($C_{16}H_{25}BrN_2S$): 357.35 g/mol. **IR (ATR) ν (cm^{-1}):** 2902, 2847, 1572, 1562, 1449, 1341, 1298, 1169, 1100, 1041, 810, 748. **^{1}H NMR (CDCl$_3$, 400 MHz): δ_H** 10.17 (s, 1H, C**H**$_{(imid)}$), 7.53 (t, ^3J = 1.8 Hz, 1H, C**H**$_{(imid)}$), 7.45 (t, ^3J = 1.8 Hz, 1H, C**H**$_{(imid)}$), 4.53 (m, 2H, NC**H**$_2$), 4.08 (s, 3H, NC**H**$_3$), 3.07 – 2.99 (m, 2H, SC**H**$_2$), 2.05 – 1.98 (br s, 3H, C**H**$_{(Ad)}$), 1.79 (d, ^3J = 2.9 Hz, 6H, C**H**$_{2(Ad)}$), 1.64 (br s, 6H, C**H**$_{2(Ad)}$). **^{13}C{1H} NMR (CDCl$_3$, 101 MHz): δ_C** 137.7 (**C**H$_{(imid)}$), 123.1 (**C**H$_{(imid)}$), 122.8 (**C**H$_{(imid)}$), 50.8 (N**C**H$_2$), 45.8 (S**C**$_{(Ad)}$), 43.5 (**C**H$_{2(Ad)}$), 36.9 (N**C**H$_3$), 36.1 (**C**H$_{2(Ad)}$), 29.7 (**C**H$_{(Ad)}$), 26.4 (S**C**H$_2$). **HRMS (ESI) m/z:** [M+Na]$^+$ calcd. for $C_{16}H_{25}N_2S$ 277.1738; Found 277.1735.

Synthesis of Rhodium complex RhL4

Scheme S4. Procedure for the synthesis of complex RhL4

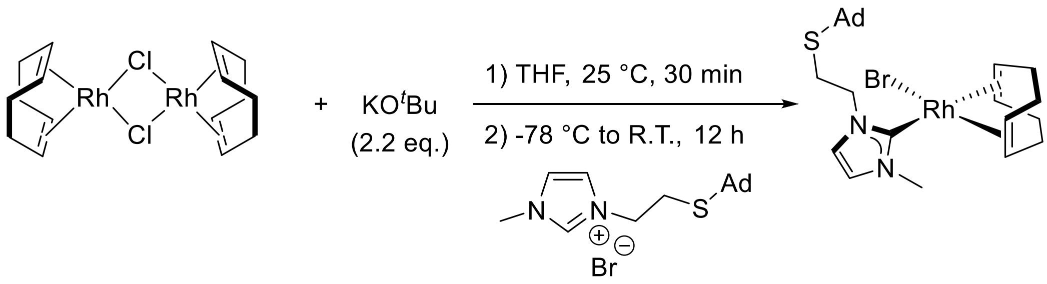

L4 (2eq.)

In a Schlenck equipped with a magnetic stirrer, a suspension of imidazolium **L4** (145 mg, 2 equiv.) in anhydrous THF was stirred at -78ºC under inert atmosphere. In another Schlenck, a solution of [Rh(cod)Cl]$_2$ (100 mg, 1 equiv.) and pure tBuOK (50 mg, 2.2 equiv.) in anhydrous THF was stirred under inert atmosphere at room temperature for 30 min. The rhodium orange solution was then added slowly to the previous suspension of **L4** at -78ºC. The resulting mixture was allowed to warm to room temperature and stirred overnight. The dark orange solution was centrifuged, the

supernatant solution was separated and the volatiles were removed in vacuum. The crude solid was washed with pentane (1 mL) and dried under vacuum to afford complex **RhL4** (218 mg, 95 % yield) as a shiny yellow solid.

Suitable crystals for X-ray analysis were obtained by slow diffusion of pentane into a solution of **RhL4** in dichloromethane.

MW ($C_{24}H_{36}BrN_2RhS$): 567.43 g/mol. **IR (ATR) ν (cm^{-1}):** 2902, 2884, 1454,1399, 1225, 1153, 1080, 1044, 953, 863, 739, 722, 697, 684. **^{1}H NMR ($CDCl_3$, 400 MHz): δ_H** 6.87 (d, 1H, ^3J = 1.9 Hz, C**H**$_{(imid)}$), 6.80 (d, 1H, ^3J = 1.9 Hz, C**H**$_{(imid)}$), 5.13 (br s, 1H, C**H**$_{(cod)}$), 5.07 – 4.94 (m, 2H, C**H**$_{(cod)}$, NC**H**$_2$), 4.11 (m, 1H, NC**H**$_2$), 4.04 (s, 3H, NC**H**$_3$), 3.44 (ddd, ^2J = 13.7 Hz, ^3J = 10.9 Hz, ^3J = 5.5 Hz, 1H, SC**H**$_2$), 3.38 – 3.31 (m, 1H, C**H**$_{(cod)}$), 3.31 – 3.24 (m, 1H, C**H**$_{(cod)}$), 2.69 (ddd, ^2J = 13.7 Hz, 11.2 Hz, ^3J = 5.4 Hz, 1H, SC**H**$_2$), 2.45 – 2.22 (m, 4H, C**H**$_{2(cod)}$), 2.11 – 2.04 (m, 6H, C**H**$_{(Ad)}$, C**H**$_{2(Ad)}$), 2.03 – 1.83 (m, 7H, C**H**$_{2(cod)}$, C**H**$_{2(Ad)}$), 1.77 (d, 3H, ^3J = 12.7 Hz, C**H**$_{2(Ad)}$), 1.69 (d, 3H, ^3J = 12.3 Hz, C**H**$_{2(Ad)}$). **^{13}C{1H} NMR ($CDCl_3$, 101 MHz): δ_C** 182.7 (d, $^1J_{Rh-C}$ = 49.9 Hz, **C**-Rh), 122.3 (**C**H$_{(imid)}$), 120.8 (**C**H$_{(imid)}$), 98.5 (bs, **C**H$_{(cod)}$), 97.9 (bs, **C**H$_{(cod)}$), 69.3 (bs, **C**H$_{(cod)}$), 69.1 (bs, **C**H$_{(cod)}$), 52.4 (N**C**H$_2$), 46.0 (S**C**$_{(Ad)}$), 43.7 (**C**H$_{2(Ad)}$), 37.9 (N**C**H$_3$), 36.3 (**C**H$_{2(Ad)}$), 32.9 (2x**C**H$_{2(cod)}$), 30.0 (**C**H$_{(Ad)}$), 29.2 (2x**C**H$_{2(cod)}$), 27.0 (S**C**H$_2$). **HRMS (ESI) m/z:** [M+Na]$^+$ calcd. for $C_{24}H_{36}N_2RhS$ 487.1649; Found 487.1651. **Elementary Analysis**: Anal. Calcd for $C_{24}H_{36}BrN_2RhS$: C, 50.80; H, 6.4; N, 4.94; found: C, 50.52; H, 6.46; N, 4.94.

Synthesis of compounds 3a-p

Scheme S5. General procedure GP1 for the synthesis of products 3a-p

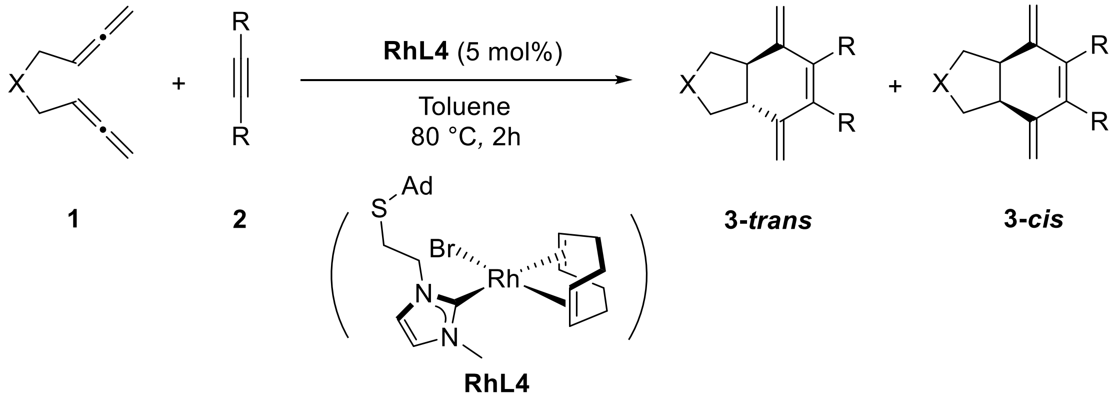

In a 10 mL capped vial, rhodium complex **RhL4** (5 mol%) was dissolved in anhydrous toluene (1 mL) and transferred via syringe into a solution of the corresponding bisallene **1** (1 equiv.) and the alkyne **2** (5 equiv.) in anhydrous toluene (1.5 mL) under inert atmosphere. The resulting mixture was heated and stirred for 2 h. at 80 °C. The solvent was then removed under reduced pressure and the resulting reaction crude was purified by flash chromatography on silica gel (silica gel, 20 µm) using mixtures of hexane/EtOAc as the eluent (90:10 to 40:60 v/v). The *trans:cis* ratio was calculated by NMR spectroscopy on the crude reaction mixture.

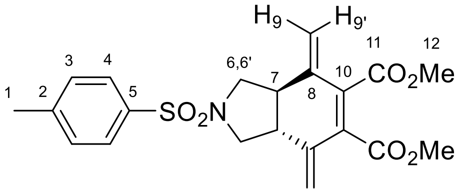

3a

Compound **3a** (d.r. 92:8) was obtained from bisallene **1a** (24.9 mg, 0.09 mmol) following the general procedure **GP1**. Purification by flash chromatography (silica gel, 20 µm, Hexanes/EtOAc 90:10 to 60:40 v/v) provided **3a** (30.3 mg, 80% yield) as a colourless solid.

MW ($C_{21}H_{23}NO_6S$): 417.48 g/mol. **Rf**: 0.32 (Hexane/EtOAc 6:4). **MP (°C):** 168 – 170 (dec.). **IR (ATR) ν (cm^{-1})**: 2922, 1720, 1333, 1156. **^{1}H NMR ($CDCl_3$, 400 MHz): δ_H** 7.73 (d, 2H, $^3J_{ortho}$ = 8.2 Hz, **H4**), 7.32 (d, 2H, $^3J_{ortho}$ = 8.2 Hz, **H3**), 5.38 (s, 2H, **H9'**), 5.06 (s, 2H, **H9**), 3.81 (dd, 2H, ^2J = 9.6 Hz, ^3J = 6.5 Hz, **H6/H6'**), 3.77 (s, 6H, **H12**), 3.28 (t, 2H, ^2J = ^3J = 9.6 Hz, **H6/H6'**), 2.50 – 2.39 (m, 2H, **H7**), 2.43 (s, 3H, **H1**). **^{13}C{1H} NMR ($CDCl_3$, 101 MHz): δ_C** 166.9 (**C11**), 143.9 (**C2**), 137.9 (**C8**), 134.5 (**C10**), 134.1 (**C5**), 130.1 (**C3**), 127.4 (**C4**), 116.6 (**C9**), 52.6 (**C12**), 50.4 (**C6**), 45.1 (**C7**), 21.7 (**C1**). **HRMS (ESI) m/z:** $[M+Na]^+$ calcd. for $C_{21}H_{23}NO_6SNa$ 440.1138; Found 440.1136.

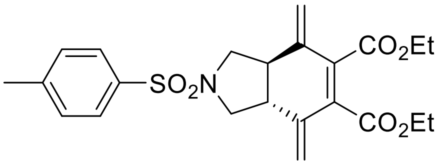

3b

Compound **3b** (d.r. 93:7) was obtained from bisallene **1a** (24.8 mg, 0.09 mmol) following the general procedure **GP1**. Purification by flash chromatography (silica gel, 20 µm, Hexanes/EtOAc 90:10 to 60:40 v/v) provided a non-separable mixture of **3b** and the cyclotrimerization of the alkyne. Yield of **3b** was obtained through the NMR of the crude using mesitylene as internal standard (84 % yield).

MW ($C_{23}H_{27}NO_6S$): 445.53 g/mol. **Rf**: 0.41 (Hexane/EtOAc 6:4). **IR (ATR) ν (cm^{-1})**: 2919, 1720, 1338, 1158. **^{1}H NMR ($CDCl_3$, 400 MHz): δ_H** 7.73 (d, 2H, $^3J_{ortho}$ = 8.2 Hz), 7.33 (d, 2H, $^3J_{ortho}$ = 8.2 Hz), 5.38 (s, 2H), 5.05 (s, 2H), 4.28 – 4.19 (m, 4H), 3.82 (dd, 2H, ^2J = 9.7 Hz, ^3J = 6.5 Hz), 3.28 (t, 2H, ^2J = ^3J = 9.7 Hz), 2.52 – 2.40 (m, 2H), 2.44 (s, 3H), 1.29 (t, 6H, ^3J = 7.1 Hz). **^{13}C{1H} NMR ($CDCl_3$, 101MHz): δ_C** 166.5, 144.0, 138.1, 134.5, 134.2, 130.1, 127.4, 116.3, 61.8, 50.5, 45.1, 21.7, 14.1. **HRMS (ESI) m/z:** $[M+Na]^+$ calcd. for $C_{23}H_{27}NO_6SNa$ 468.1451; Found 468.1448.

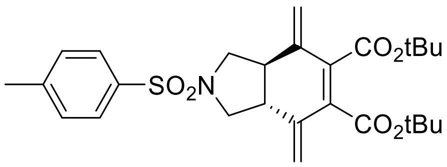

3c

Compound **3c** (d.r. 92:8) was obtained from bisallene **1a** (24.8 mg, 0.09 mmol) following the general procedure **GP1**. Purification by flash chromatography (silica gel, 20 µm, Hexanes/EtOAc 90:10 to 60:40 v/v) provided **3c** (35.8 mg, 79 % yield) as a colourless oil.

MW ($C_{27}H_{35}NO_6S$): 501.64 g/mol. **Rf**: 0.56 (Hexane/EtOAc 6:4). **IR (ATR) ν (cm^{-1})**: 2929, 1715, 1336, 1151. **^{1}H NMR (CDCl$_3$, 400 MHz): δ_H** 7.73 (d, 2H, $^3J_{ortho}$ = 8.2 Hz), 7.32 (d, 2H, $^3J_{ortho}$ = 8.2 Hz), 5.31 (s, 2H), 4.97 (s, 2H), 3.79 (dd, 2H, ^2J = 9.7 Hz, ^3J = 6.4 Hz), 3.26 (t, 2H, ^2J = ^3J = 9.7 Hz), 2.49 – 2.38 (m, 2H), 2.44 (s, 3H), 1.51 (s, 18H). **^{13}C{1H} NMR (CDCl$_3$, 101 MHz): δ_C** 166.1, 143.9, 138.6, 134.8, 134.1, 130.0, 127.4, 115.1, 83.0, 50.5, 45.1, 28.1, 21.7. **HRMS (ESI) m/z:** [M+Na]$^+$ calcd. for $C_{27}H_{35}NO_6SNa$ 524.2077; Found 524.2079.

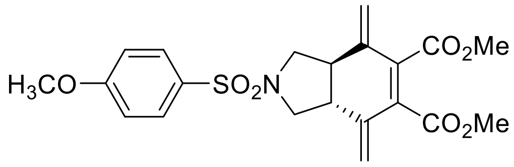

3d

Compound **3d** (d.r. 91:9) was obtained from bisallene **1b** (26.2 mg, 0.09 mmol) following the general procedure **GP1**. Purification by flash chromatography (silica gel, 20 µm, Hexanes/EtOAc 90:10 to 60:40 v/v) provided **3d** (30.4 mg, 78% yield) as a colourless solid.

MW ($C_{21}H_{23}NO_7S$): 433.47 g/mol. **Rf**: 0.28 (Hexane/EtOAc 6:4). **MP (°C):** 138 – 140 (dec.). **IR (ATR) ν (cm^{-1})**: 2950, 1721, 1338, 1155. **^{1}H NMR (400 MHz, CDCl$_3$): δ_H** 7.78 (d, 2H, $^3J_{ortho}$ = 8.8 Hz), 6.99 (d, 2H, $^3J_{ortho}$ = 8.8 Hz), 5.38 (s, 2H), 5.06 (s, 2H), 3.87 (s, 3H), 3.85 – 3.74 (m, 2H), 3.77 (s, 6H), 3.27 (t, 2H, ^2J = ^3J = 9.7 Hz), 2.52 – 2.39 (m, 2H). **^{13}C{1H} NMR (101 MHz, CDCl$_3$): δ_C** 166.9, 163.2, 137.9, 134.5, 129.5, 128.8, 116.6, 114.6, 55.7, 52.6, 50.4, 45.1. **HRMS (ESI) m/z:** [M+Na]$^+$ calcd.for $C_{21}H_{23}NO_7SNa$ 456.1087; Found 456.1089.

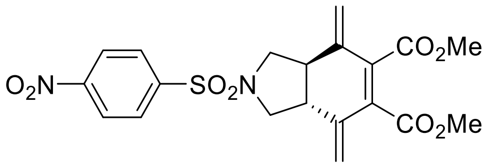

3e

Compound **3e** was obtained from bisallene **1c** (27.6 mg, 0.09 mmol) following the general procedure **GP1**. Purification by filtration of the crude provided **3e** (26.1 mg, 65 % yield, d.r. 100:0) as a colourless solid.

MW ($C_{20}H_{20}N_2O_8S$): 448.45 g/mol. **Rf**: 0.32 (Hexane/EtOAc 6:4). **MP (°C):** 258 – 259 (dec.). **IR (ATR) ν (cm^{-1})**: 2953, 1717, 1535, 1343, 1161. **^{1}H NMR (CDCl$_3$, 400MHz): δ_H** 8.40 (d, 2H, $^3J_{ortho}$ = 8.7 Hz), 8.05 (d, 2H, $^3J_{ortho}$ = 8.7 Hz), 5.43 (s, 2H), 5.08 (s, 2H), 3.87 (dd, 2H, ^2J = 9.4 Hz, ^3J = 6.2 Hz), 3.79 (s, 6H), 3.34 (t, 2H, ^2J = ^3J = 9.4 Hz), 2.61 – 2.49 (m, 2H). **^{13}C{1H} NMR (101 MHz, CDCl$_3$): δ_C** 166.8, 150.4, 143.5, 137.5, 134.5, 128.5, 124.8, 116.9, 52.7, 50.5, 45.3. **HRMS (ESI) m/z:** [M+Na]$^+$ calcd. for $C_{20}H_{20}N_2O_8SNa$ 471.0833; Found 471.0837.

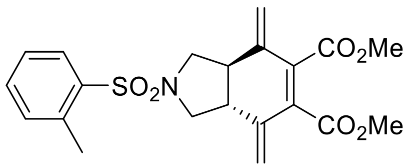

3f

Compound **3f** (d.r. 90:10) was obtained from bisallene **1d** (25.0 mg, 0.09 mmol) following the general procedure **GP1**. Purification by flash chromatography (silica gel, 20 µm, Hexanes/EtOAc 90:10 to 60:40 v/v) provided **3f** (30.1 mg, 79 % yield) as a colourless oil.

MW ($C_{21}H_{23}NO_6S$): 417.48 g/mol. **Rf**: 0.40 (Hexane/EtOAc 6:4). **IR (ATR) *ν* (cm^{-1})**: 2948, 1719, 1317, 1156. **^{1}H NMR ($CDCl_3$, 400 MHz): δ_H** 7.93 (dd, 1H, $^3J_{ortho}$ = 8.2 Hz, $^4J_{meta}$ = 1.4 Hz), 7.47 (td, 1H, $^3J_{ortho}$ = 7.5 Hz, $^4J_{meta}$ = 1.4 Hz), 7.35 – 7.31 (m, 2H), 5.42 (s, 2H), 5.08 (s, 2H), 3.88 – 3.82 (m, 2H), 3.80 (s, 6H), 3.37 (dd, 2H, ^2J = 9.1 Hz, ^3J = 10.0 Hz), 2.75 – 2.68 (m, 2H), 2.65 (s, 3H). **^{13}C{1H} NMR ($CDCl_3$, 101 MHz): δ_C** 167.0, 138.0, 137.9, 137.3, 134.6, 133.1, 133.0, 129.6, 126.4, 116.7, 52.7, 49.7, 45.5, 20.7; **HRMS (ESI) m/z:** $[M+Na]^+$ calcd. for $C_{21}H_{23}NO_6SNa$ 440.1138; Found 440.1139.

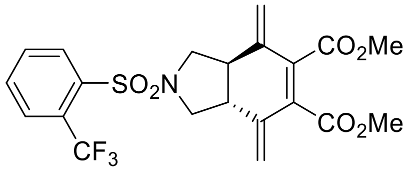

3g

Compound **3g** (d.r. 92:8) was obtained from bisallene **1e** (29.4 mg, 0.09 mmol) following the general procedure **GP1**. Purification by flash chromatography (silica gel, 20 µm, Hexanes/EtOAc 90:10 to 60:40 v/v) provided **3g** (34.0 mg, 81 % yield) as a colourless oil.

MW ($C_{21}H_{20}F_3NO_6S$): 471.45 g/mol. **Rf**: 0.30 (Hexane/EtOAc 6:4). **IR (ATR) *ν* (cm^{-1})**: 2952, 1716, 1266, 1138 **^{1}H NMR ($CDCl_3$, 400 MHz): δ_H** 8.20 – 8.13 (m, 1H), 7.94 – 7.87 (m, 1H), 7.71 (m, 2H), 5.43 (s, 2H), 5.08 (s, 2H), 3.91 (dd, 2H, ^2J = 9.4 Hz, ^3J = 6.5 Hz), 3.80 (s, 6H), 3.42 (t, 2H, ^2J = ^3J = 9.4 Hz), 2.80 – 2.66 (m, 2H). **^{13}C{1H} NMR ($CDCl_3$, 101 MHz): δ_C** 167.0, 138.6 (q, $^4J_{C-F}$ = 1.5 Hz), 137.8, 134.6, 132.9, 132.5 (q, $^4J_{C-F}$ = 1.0 Hz), 131.4, 128.8 (q, $^3J_{C-F}$ = 6.5 Hz), 128.0 (q, $^2J_{C-F}$ = 33.3 Hz), 122.7 (q, $^1J_{C-F}$ = 275.1 Hz) 116.7, 52.7, 50.1, 45.4. **HRMS (ESI) m/z:** $[M+Na]^+$ calcd. for $C_{21}H_{20}F_3NO_6SNa$ 494.0856; Found 494.0857.

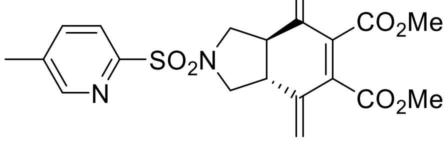

3h

Compound **3h** (d.r. 87:13) was obtained from bisallene **1f** (25.0 mg, 0.09 mmol) following the general procedure **GP1**. Purification by flash chromatography (silica gel, 20 µm, Hexanes/EtOAc 90:10 to 60:40 v/v) provided **3h** (22.7 mg, 60 % yield) as a colourless solid.

MW ($C_{20}H_{22}N_2O_6S$): 418.46 g/mol. **Rf**: 0.23 (Hexane/EtOAc 6:4). **MP (°C):** 170 – 171 (dec.). **IR (ATR) ν (cm^{-1}):** 2952, 1720, 1340, 1165. **^{1}H NMR (CDCl$_3$, 400 MHz): δ_H** 8.47 – 8.46 (m, 1H), 7.86 (d, 1H, $^3J_{ortho}$ = 8.0 Hz), 7.68 (dd, 1H, $^3J_{ortho}$ = 8.0 Hz, $^4J_{meta}$ = 1.6 Hz), 5.39 (s, 2H), 5.06 (s, 2H), 4.04 (dd, 2H, ^2J = 9.7 Hz, ^3J = 6.5 Hz), 3.79 (s, 6H), 3.46 (t, 2H, ^2J = ^3J = 9.7 Hz), 2.71 – 2.59 (m, 2H), 2.42 (s, 3H). **^{13}C{1H} NMR (CDCl$_3$, 101 MHz): δ_C** 167.0, 154.1, 150.8, 138.1, 138.0, 137.5, 134.6, 122.8, 116.6, 52.7, 51.3, 45.2, 18.7. **HRMS (ESI) m/z:** [M+Na]$^+$ calcd. for $C_{20}H_{22}N_2O_6SNa$ 441.1091; Found 441.1084.

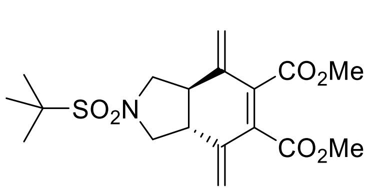

3i

Compound **3i** (d.r. 83:17) was obtained from bisallene **1g** (21.5 mg, 0.09 mmol) following the general procedure **GP1**. Purification by flash chromatography (silica gel, 20 µm, Hexanes/EtOAc 90:10 to 60:40 v/v) provided **3i** (27.0 mg, 79 % yield, dr 83:17) as a colourless solid.

MW ($C_{18}H_{25}NO_6S$): 383.46 g/mol. **Rf**: 0.39 (Hexane/EtOAc 6:4). **MP (°C):** 197 – 198 (dec.). **IR (ATR) ν (cm^{-1}):** 2952, 1728, 1305, 1113. **^{1}H NMR (CDCl$_3$, 400 MHz): δ_H** 5.42 (s, 2H), 5.09 (s, 2H), 3.97 – 3.90 (m, 2H), 3.80 (s, 6H), 3.50 (t, 2H, ^2J = ^3J = 9.4 Hz), 2.79 – 2.65 (m, 2H), 1.41 (s, 9H). **^{13}C{1H} NMR (CDCl$_3$, 101 MHz): δ_C** 167.0, 138.1, 134.6, 116.6, 61.6, 52.6, 52.1, 45.7, 24.7. **HRMS (ESI) m/z:** [M+Na]$^+$ calcd. for $C_{18}H_{25}NO_6SNa$ 406.1295; Found 406.1301.

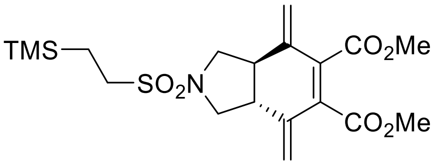

3j

Compound **3j** (d.r. 89:11) was obtained from bisallene **1h** (26.0 mg, 0.09 mmol) following the general procedure **GP1**. Purification by flash chromatography (silica gel, 20 µm, Hexanes/EtOAc 90:10 to 60:40 v/v) provided **3j** (22.7 mg, 58 % yield) as a colourless solid.

MW ($C_{19}H_{29}NO_6SSi$): 427.59 g/mol. **Rf**: 0.44 (Hexane/EtOAc 6:4). **MP (°C):** 109 – 111 (dec.). **IR (ATR) ν (cm^{-1}):** 2950, 1723, 1332, 1270, 1148. **^{1}H NMR (CDCl$_3$, 400 MHz): δ_H** 5.45 (s, 2H), 5.11 (s, 2H), 3.88 (dd, 2H, ^2J = 9.4 Hz, ^3J = 6.2 Hz), 3.81 (s, 6H), 3.42 (t, 2H, ^2J = ^3J = 9.4 Hz), 2.97 – 2.91 (m, 2H), 2.80 – 2.69 (m, 2H), 1.07 – 0.90 (m, 2H), 0.05 (s, 9H); **^{13}C{1H} NMR (CDCl$_3$, 101 MHz): δ_C** 167.0, 138.0, 134.6, 116.7, 52.7, 50.3, 47.0, 45.7, 10.3, -1.8. **HRMS (ESI) m/z:** [M+Na]$^+$ calcd. for $C_{19}H_{29}NO_6SSiNa$ 450.1377; Found 450.1374.

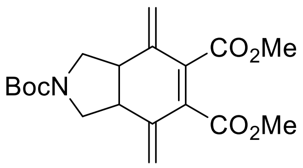

3k

Compound **3k** (d.r. 50:50) was obtained from bisallene **1i** (19.5 mg, 0.09 mmol) following the general procedure **GP1**. Purification by flash chromatography (silica gel, 20 µm, Hexanes/EtOAc 90:10 to 60:40 v/v) provided **3k** (16.8 mg, 53 % yield) as a colourless solid.

MW ($C_{19}H_{25}NO_6$): 363.41 g/mol. **Rf**: 0.42 (Hexane/EtOAc 6:4). **MP (°C):** 153 – 155 (dec.). **IR (ATR)** ***ν*** **(cm^{-1}):** 2951, 1724, 1675, 1229. **^{1}H NMR ($CDCl_3$, 400 MHz): δ_H*** 5.42 (s, 2H^a, 2H^b), 5.15 (s, 2H^a), 5.13 (s, 2H^b), 3.88 (dd, 2H^a, ^2J = 10.2 Hz, ^3J = 6.0 Hz), 3.81 (s, 6H$^{a/b}$), 3.81 (s, 6H$^{a/b}$), 3.81 – 3.76 (m, 2H^b), 3.34 (t, 2H^a, ^2J = ^3J = 10.2 Hz), 3.31 (t, 2H^b, ^2J = ^3J = 10.1 Hz), 2.72 – 2.57 (m, 2H^a, 2H^b), 1.49 (s, 9H$^{a/b}$), 1.48 (s, 9H$^{a/b}$). **^{13}C{1H} NMR ($CDCl_3$, 101 MHz): δ_C*** 167.3, 154.6, 138.8 (a/b), 138.6 (a/b), 134.8 (a/b), 134.6 (a/b), 116.4 (a), 116.1 (b), 79.9, 52.6, 48.6 (b), 48.2 (a), 45.4 (b), 44.7 (a), 28.7. **HRMS (ESI) m/z:** $[M+Na]^+$ calcd. for $C_{19}H_{25}NO_6Na$ 386.1574; Found 386.1581.

*Some NMR signals can be assigned to a particular diastereoisomer, but since we cannot discern between *trans* and *cis*, they are named as ‘a’ or ‘b’ (a/b = ‘a’ or ‘b’). In ^{13}C NMR, nonlabelled signals have the same shift for both diastereoisomers.

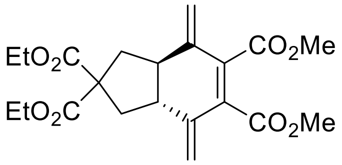

3l

Compound **3l** was obtained from bisallene **1j** (23.6 mg, 0.09 mmol) following the general procedure **GP1**. Purification by flash chromatography (silica gel, 20 µm, Hexanes/EtOAc 90:10 to 83:17 v/v) provided **3l** (22.1 mg, 61 % yield, d.r. 80:20) as a colourless oil.

MW ($C_{21}H_{26}O_8$): 406.43 g/mol. **Rf**: 0.51 (Hexane/EtOAc 6:4). **IR (ATR)** ***ν*** **(cm^{-1}):** 2950, 1719, 1245. **^{1}H NMR ($CDCl_3$, 400 MHz): δ_H** 5.38 (s, 2H), 5.22 (s, 2H), 4.25 – 4.16 (m, 4H), 3.80 (s, 6H), 2.81 (dd, 2H, ^2J = 13.0 Hz, ^3J = 6.0 Hz), 2.42 – 2.37 (m, 2H), 2.12 (dd, 2H, ^2J = 13.0 Hz, ^3J = 11.2 Hz), 1.26 (t, 6H, ^3J = 7.1 Hz). **^{13}C{1H} NMR ($CDCl_3$, 101 MHz): δ_C** 172.3, 167.6, 141.0, 134.9, 115.9, 61.9, 58.4, 52.5, 46.2, 37.0, 14.2. **HRMS (ESI) m/z:** $[M+Na]^+$ calcd for $C_{21}H_{26}O_8Na$ 429.1520; Found 429.1523.

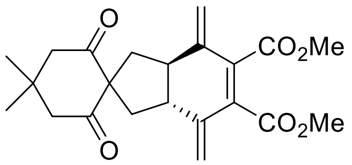

3m

Compound **3m** was obtained from bisallene **1k** (16.9 mg, 0.07 mmol) following the general procedure **GP1**. Purification by flash chromatography (silica gel, 20 µm, Hexanes/EtOAc 90:10 to 60:40 v/v) provided **3m** (21.6 mg, 81 % yield, d.r. 61:39) as a colourless oil.

Some impurities observed in NMR spectra are due to the decomposition of the product.

MW ($C_{22}H_{26}O_6$): 386.44 g/mol. **Rf**: 0.37 (Hexane/EtOAc 6:4).; **IR (ATR)** ν **(cm^{-1}):** 2951, 1719, 1690. **^{1}H NMR (CDCl$_3$, 400 MHz): δ_H (*trans)*** 5.37 (s, 2H), 5.19 (s, 2H), 3.79 (s, 6H), 2.67 – 2.63 (m, 4H), 2.45 (dd, 2H, ^2J = 11.5 Hz, ^3J = 5.8 Hz), 2.40 – 2.33 (m, 2H), 2.16 (t, 2H, ^2J = ^3J = 11.5 Hz), 1.01 (s, 6H). **^{1}H NMR (CDCl$_3$, 400 MHz): δ_H (*cis*)** 5.44 (s, 2H), 5.42 (s, 2H), 3.78 (s, 6H), 2.96 – 2.88 (m, 2H), 2.56 (s, 4H), 2.33 (d, 4H, ^3J = 6.8 Hz), 0.97 (s, 6H). **^{13}C{1H} NMR (CDCl$_3$, 101 MHz): δ_C (*trans*)** 207.3, 167.5, 140.9, 134.8, 115.9, 69.0, 52.5, 51.7, 43.1, 36.5, 34.3, 28.5. **^{13}C NMR (CDCl$_3$, 101 MHz): δ_C (*cis*)** 205.5, 167.7, 137.4, 132.6, 119.8, 70.6, 52.5, 52.0, 46.0, 35.0, 30.6, 28.5. **HRMS (ESI) m/z:** $[M+Na]^+$ calcd. for $C_{22}H_{26}O_6Na$ 409.1622; Found 409.1623.

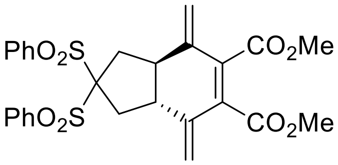

3n

Compound **3n** was obtained from bisallene **1m** (25.9 mg, 0.06 mmol) following the general procedure **GP1**. Purification by flash chromatography (silica gel, 20 µm, Hexanes/EtOAc 90:10 to 60:40 v/v) provided **3n** (18.9 mg, 54 % yield, d.r. 91:9) as a colourless oil.

MW ($C_{27}H_{26}O_8S_2$): 542.62 g/mol. **Rf**: 0.26 (Hexane/EtOAc 6:4). **IR (ATR)** ν **(cm^{-1}):** 2949, 1719, 1232, 1137. **^{1}H NMR (CDCl$_3$, 400 MHz): δ_H** 8.07 (d, 4H, ^3J = 7.6 Hz), 7.75 (t, 2H, ^3J = 7.6 Hz), 7.63 (t, 4H, ^3J = 7.6 Hz), 5.41 (s, 2H), 5.14 (s, 2H), 3.80 (s, 6H), 2.88 (dd, 2H, ^2J = 14.2, ^3J = 5.8 Hz), 2.60 – 2.52 (m, 2H), 2.48 – 2.43 (m, 2H). **^{13}C{1H} NMR (, CDCl$_3$, 101 MHz): δ_C** 167.2, 140.1, 136.1, 135.1, 134.6, 131.6, 129.1, 116.1, 91.7, 52.6, 45.7, 35.2. **HRMS (ESI) m/z:** $[M+Na]^+$ calcd. for $C_{27}H_{26}O_8S_2Na$ 565.0961; Found 565.0960.

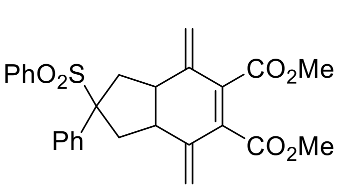

3o

Compound **3o** was obtained from bisallene **1n** (30.5 mg, 0.09 mmol) following the general procedure **GP1**. Purification by flash chromatography (silica gel, 20 µm, Hexanes/EtOAc 90:10 to 60:40 v/v) provided **3o** (24.8 mg, 57 % yield, mixture of diastereoisomers) as a colourless oil.

MW ($C_{27}H_{26}O_6S$): 478.56 g/mol. **Rf**: 0.30 (Hexane/EtOAc 6:4). **IR (ATR)** ν **(cm^{-1}):** 2949, 1719, 1232, 1137. **HRMS (ESI) m/z:** $[M+Na]^+$ calcd. for $C_{27}H_{26}O_6SNa$ 501.1342; Found 501.1351. ^{1}H and ^{13}C NMR signals not described due to the complexity of the spectra.

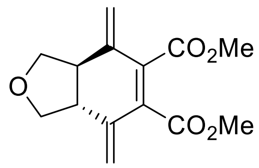

3p

Compound **3p** was obtained from bisallene **1o** (11.0 mg, 0.09 mmol) following the general procedure **GP1**. Purification by flash chromatography (silica gel, 20 µm, Hexanes/EtOAc 90:10 to 60:40 v/v) provided **3n** (14.4 mg, 61 % yield, d.r. 96:4) as a colourless solid.

MW ($C_{14}H_{16}O_5$): 264.28 g/mol. **Rf**: 0.42 (Hexane/EtOAc 6:4). **MP (°C):** 222 – 223 (dec.). **IR (ATR)** ***ν*** **(cm^{-1}):** 2951, 1718, 1245, 1134. **^{1}H NMR ($CDCl_3$, 400 MHz):** **δ_H** 5.40 (d, 2H, ^4J = 1.2 Hz), 5.03 (d, 2H, ^4J = 1.1 Hz), 4.23 (t, 2H, ^2J = ^3J = 7.2 Hz), 3.81 (s, 6H), 3.83 – 3.78 (m, 2H), 2.79 – 2.67 (m, 2H). **^{13}C{1H} NMR ($CDCl_3$, 101 MHz):** **δ_C** 167.3, 138.4, 135.2, 116.0, 69.7, 52.6, 46.7. **HRMS (ESI) m/z:** $[M+Na]^+$ calcd. for $C_{14}H_{16}O_5Na$ 287.0890; Found 287.0897.

Compounds **3l, 3n**, and **3o** are quite unstable in solution and when the 1H-NMR spectra was performed, they start to decompose in the resonance tube itself and so it is difficult to have clean spectra.

Computational methods

Geometries of all stationary points were optimized without symmetry constraint with the Gaussian 16 program[8] using the DFT B3LYP hybrid exchange-correlation functional.[9] The all-electron cc-pVDZ basis set[10] was employed for non-metal atoms and the cc-pVDZ-PP basis set[11] containing an effective core relativistic pseudopotential was used for Rh. The electronic energy was improved by performing single point energy calculations with the cc-pVTZ (cc-pVTZ-PP for Rh) basis set and the ωB97X-D functional,[12] including solvent effects corrections computed with the solvent model based on density (SMD) continuum solvation model[13] using the keyword SCRF=(SMD,Solvent=Toluene). The D3 Grimme energy corrections for dispersion[14] with the original damping function were added in all B3LYP/cc-pVDZ-PP calculations. The ωB97X-D functional has integrated a version of D2 dispersion model with a similar damping function to that used by the D3 model. Analytical Hessians were computed to determine the nature of stationary points (one and zero imaginary frequencies for transition states and minima, respectively) and to calculate unscaled zero-point energies (ZPEs) as well as thermal corrections and entropy effects using the standard statistical-mechanics relationships for an ideal gas.[15] These two latter terms were computed at 353.15 K and 1 atm to provide the reported relative Gibbs energies. As a summary, the reported Gibbs energies contain electronic energies including solvent effects calculated at the ωB97X-D/cc-pVTZ-PP(SMD,Solvent=Toluene) // B3LYP-D3/cc-pVDZ-PP level together with gas phase thermal and entropic contributions computed at 353.15 K and 1 atm with the B3LYP-D3/cc-pVDZ-PP method. All stationary points were unambiguously confirmed by IRC calculations.

Figure S1. Gibbs energy formation of the complexes evaluated, resulting from the coordination of 1,5-bisallene **1a** and DMAD **2a** into **RhL4**. Carboxylates of DMAD are omitted for clarity.

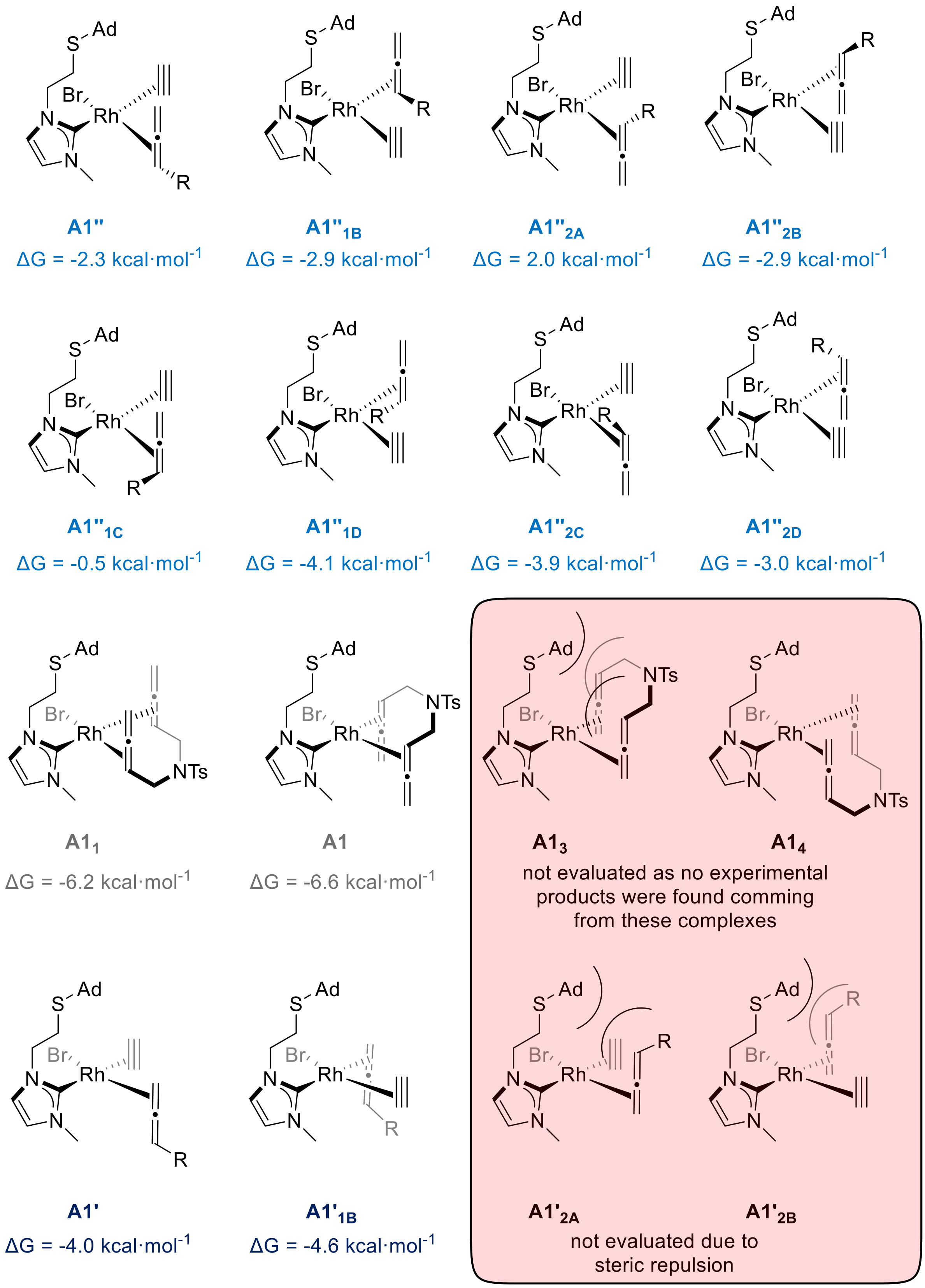

Figure S2. Gibbs energy barriers of the paths evaluated for the oxidative cyclometallation in the formation of **3a** without the sulfur chelating the rhodium.

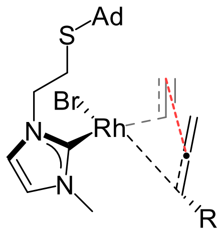

TS(A1''A2)

$\Delta G^{\ddagger}$ = 31.6 kcal·mol^{-1}

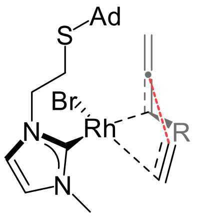

TS(A1''$_{1B}$A2)

$\Delta G^{\ddagger}$ = 29.5 kcal·mol^{-1}

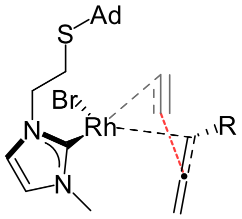

TS(A1''$_{2A}$A2)

$\Delta G^{\ddagger}$ = 29.1 kcal·mol^{-1}

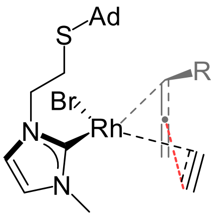

TS(A1''$_{2B}$A2)

$\Delta G^{\ddagger}$ = 35.9 kcal·mol^{-1}

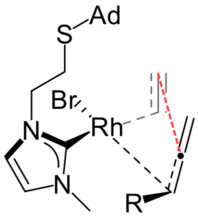

TS(A1''$_{1C}$A2)

$\Delta G^{\ddagger}$ = 26.8 kcal·mol^{-1}

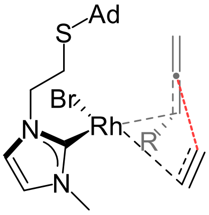

TS(A1''$_{1D}$A2)

$\Delta G^{\ddagger}$ = 34.8 kcal·mol^{-1}

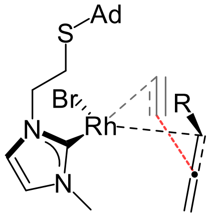

TS(A1''$_{2C}$A2)

$\Delta G^{\ddagger}$ = 25.0 kcal·mol^{-1}

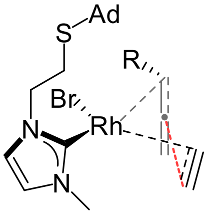

TS(A1''$_{2D}$A2)

$\Delta G^{\ddagger}$ = 25.7 kcal·mol^{-1}

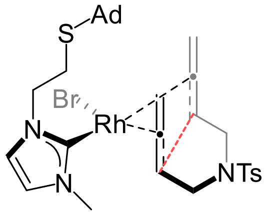

TS(A1$_1$A2)

$\Delta G^{\ddagger}$ = 35.3 kcal·mol^{-1}

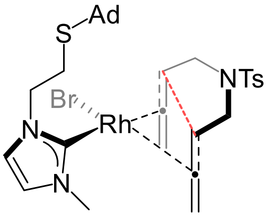

TS(A1A2)

$\Delta G^{\ddagger}$ = 33.7 kcal·mol^{-1}

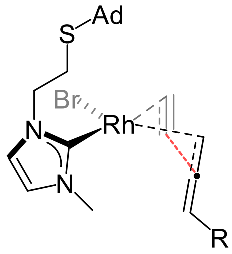

TS(A1'A2)

$\Delta G^{\ddagger}$ = 24.3 kcal·mol^{-1}

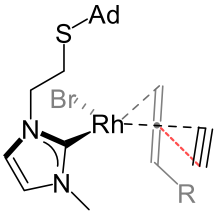

TS(A1'$_{1B}$A2)

$\Delta G^{\ddagger}$ = 23.0 kcal·mol^{-1}

Figure S3. Gibbs energy barriers of the paths evaluated for the oxidative cyclometallation in the formation of **3a** with the sulfur chelating the rhodium.

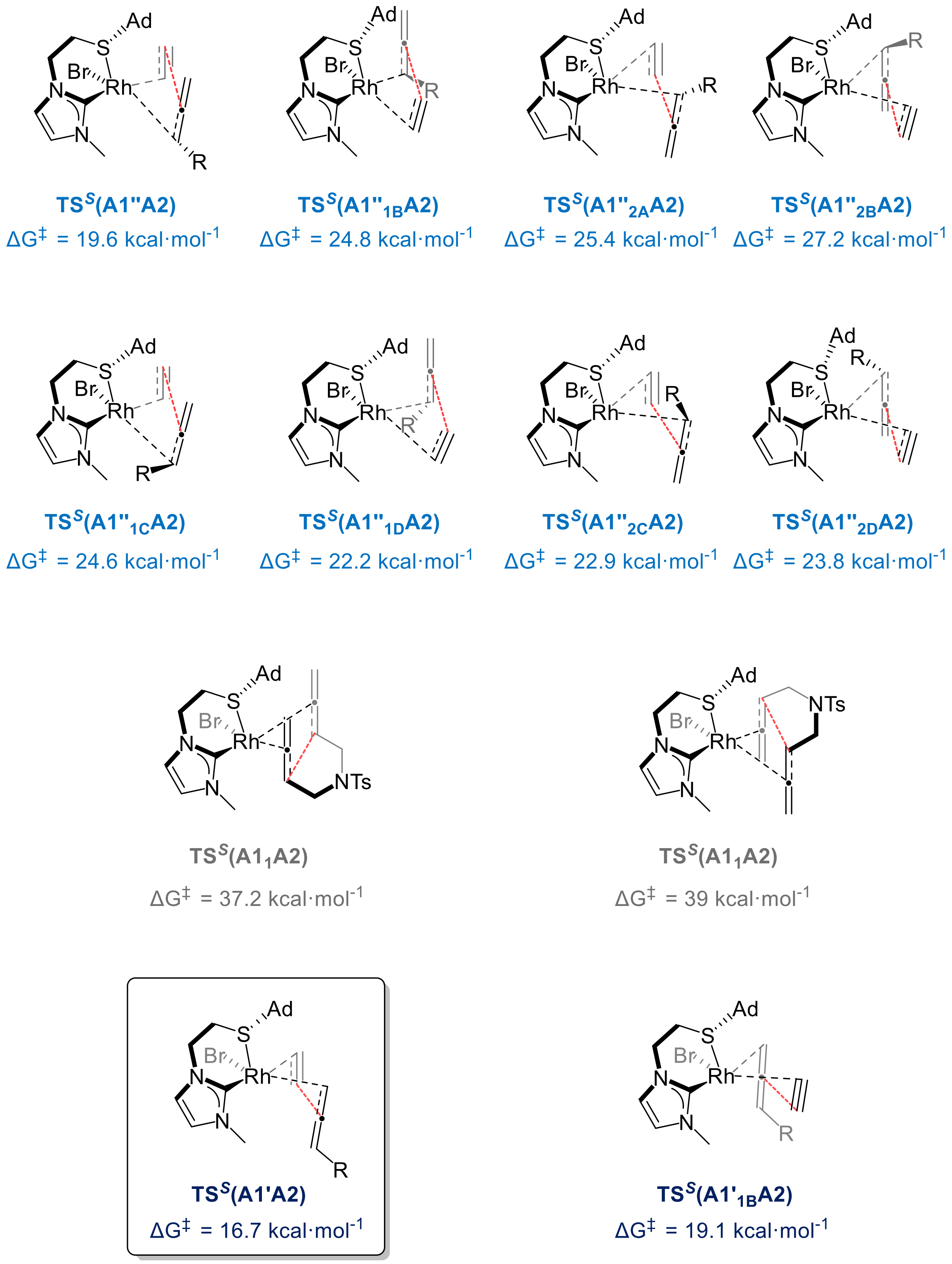

Scheme S6. Alternative reaction paths evaluated leading to **4** and to our previously reported *cis*-3,4-arylvinyl pyrrolidine derivative **P1** through **IV**.

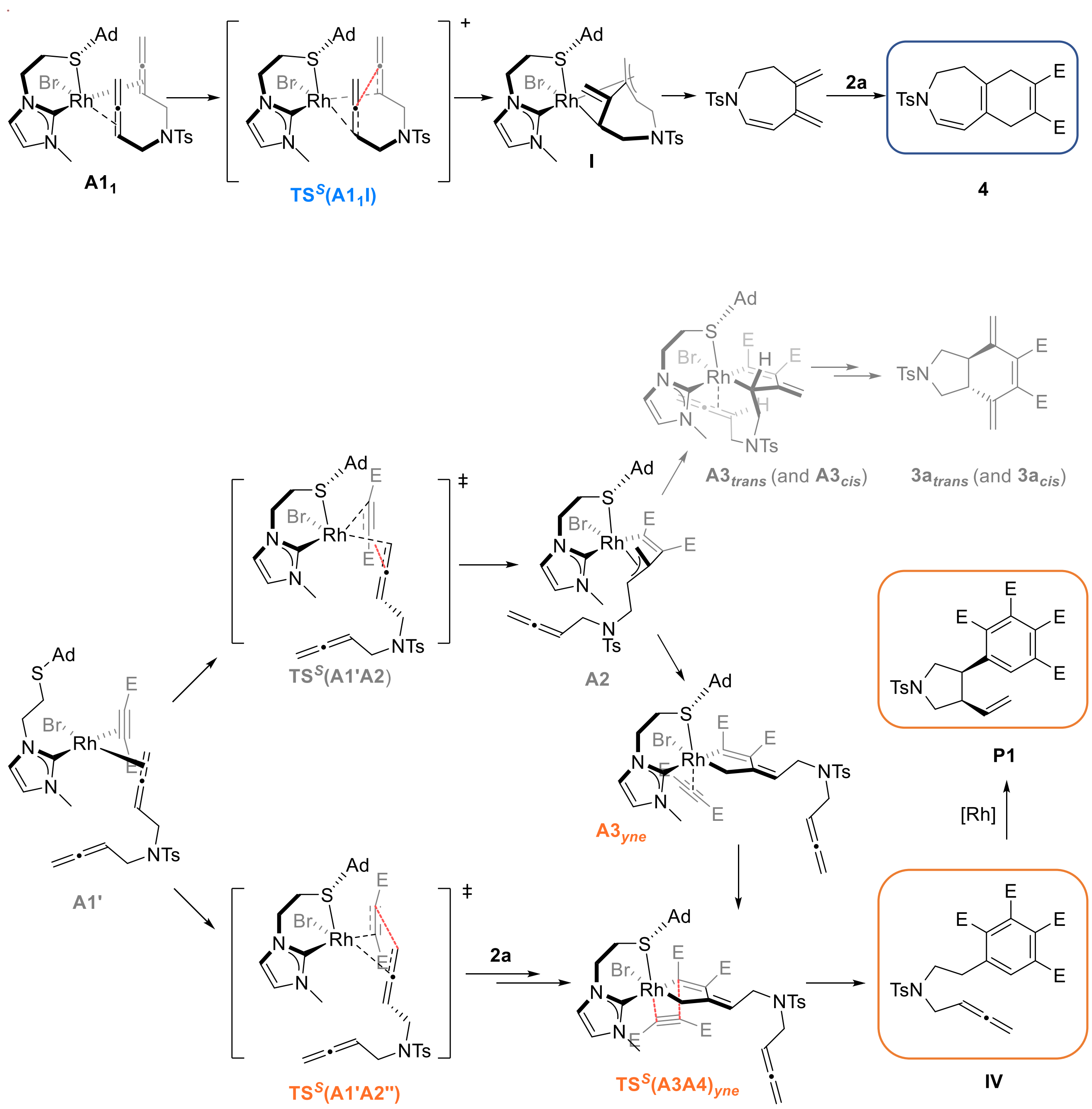

Figure S4. Energy profile of the reaction including the alternative paths leading to **4** and to our previously reported *cis*-3,4-arylvinyl pyrrolidine derivative **P1**. Calculated at 353.15 K and 1 atm at the ωB97X-D/cc-pVTZ-PP(SMD,Solvent=Toluene) // B3LYP-D3/cc-pVDZ-PP theory level.

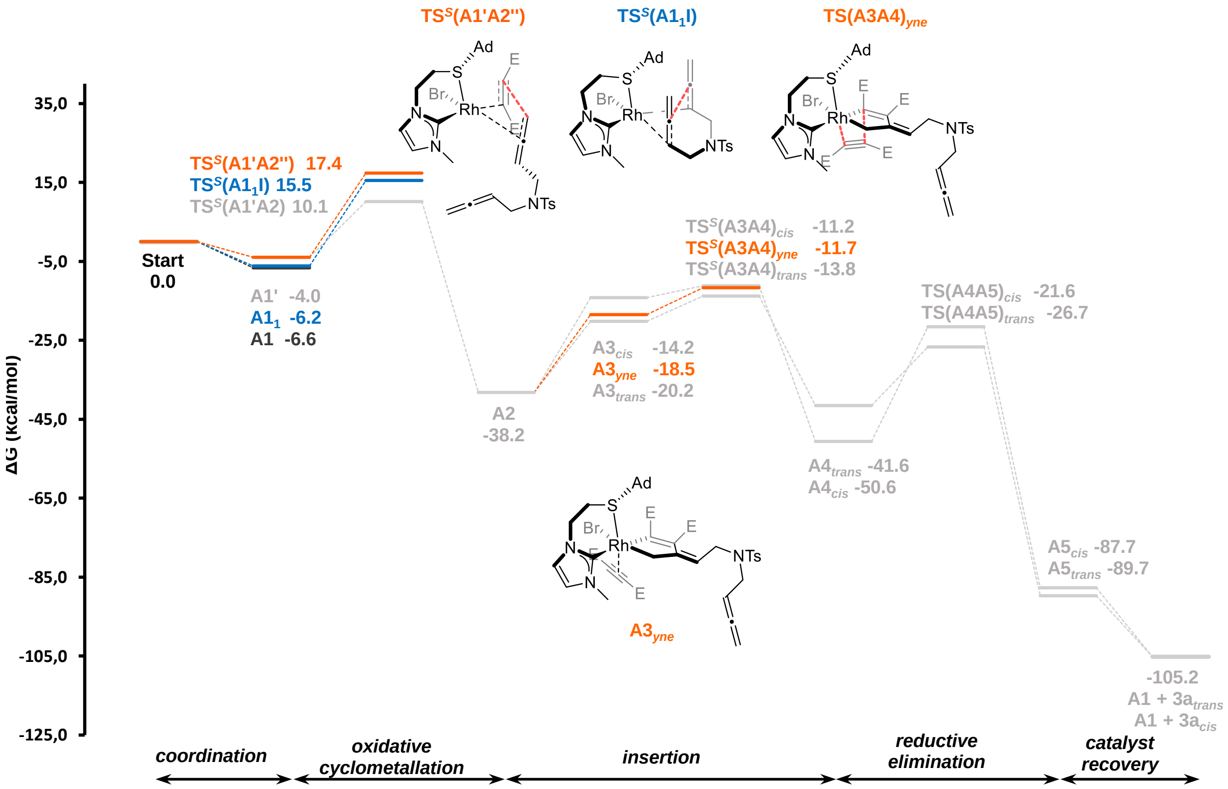

Figure S5. Graphical representation of the **I**:**A2** production ratio versus the concentration of DMAD **2a**.

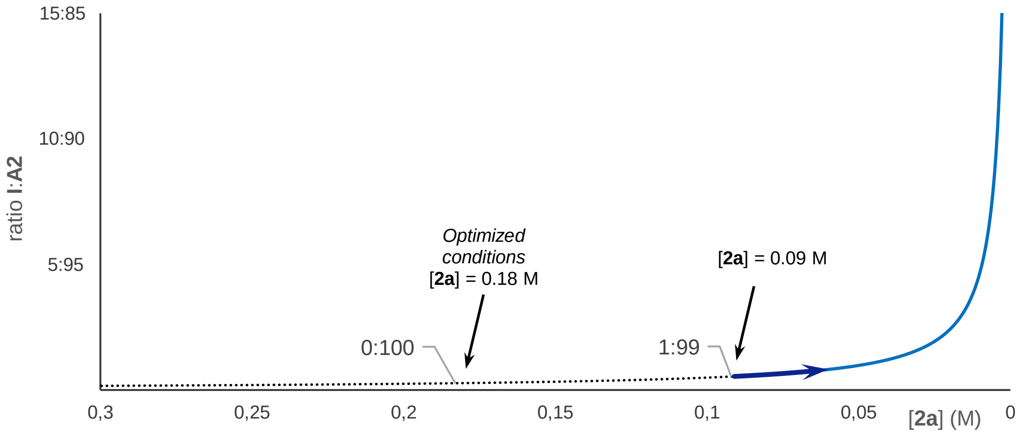

As $v_{A2} = \frac{d[A2]}{dt} = k_{A2}[A1][2a]$ and $v_I = \frac{d[I]}{dt} = k_I[A1]$, then $\frac{v_{A2}}{v_I} = \frac{k_{A2}[A1][2a]}{k_I[A1]} = \frac{k_{A2}}{k_I}[2a]$

Using the Eyring equation: $k_i = \frac{k_B T}{h} e^{\frac{-\Delta G_i^{\ddagger}}{RT}} \rightarrow \frac{v_{A2}}{v_I} = \frac{e^{\frac{-\Delta G_{A2}^{\ddagger}}{RT}}}{e^{\frac{-\Delta G_I^{\ddagger}}{RT}}}[2a] = [2a]e^{\frac{\Delta G_I^{\ddagger} - \Delta G_{A2}^{\ddagger}}{RT}}$

The $\frac{v_{A2}}{v_I}$ is normalized and plotted versus decreasing [**2a**].

The energy barrier for the oxidative cyclometallation between the two central carbons of the 1,5-bisallene, leading to **I** (**TS^S(A1$_1$I)**), is found to be 5.4 kcal·mol^{-1} higher than **TS^S(A1'A2)**, which explains the absence of **4** under the optimized reaction conditions. The production rate of **A2** depends on the concentration of DMAD, which is consumed during the reaction to produce its cyclotrimerization adduct and **3a**. At higher starting concentrations of DMAD ([**2a**] = 0.18 M, optimized conditions), the amount of DMAD is not reduced enough during the reaction to significantly reduce the production rate of **A2**. However, the amount of DMAD rapidly decreases at lower concentrations, and thus, the oxidative cyclometallation towards **I** is less disfavored. This explains the production of **4** in low amounts during the optimization of the reaction conditions at lower concentrations of **2a** (entries 5 and 9, Table 1; [**2a**] = 0.09 and 0.072 M respectively).

Figure S6. Graphical representation of the **A4**$_{cis}$:**A4**$_{yne}$ production ratio versus the concentration of DMAD **2a**.

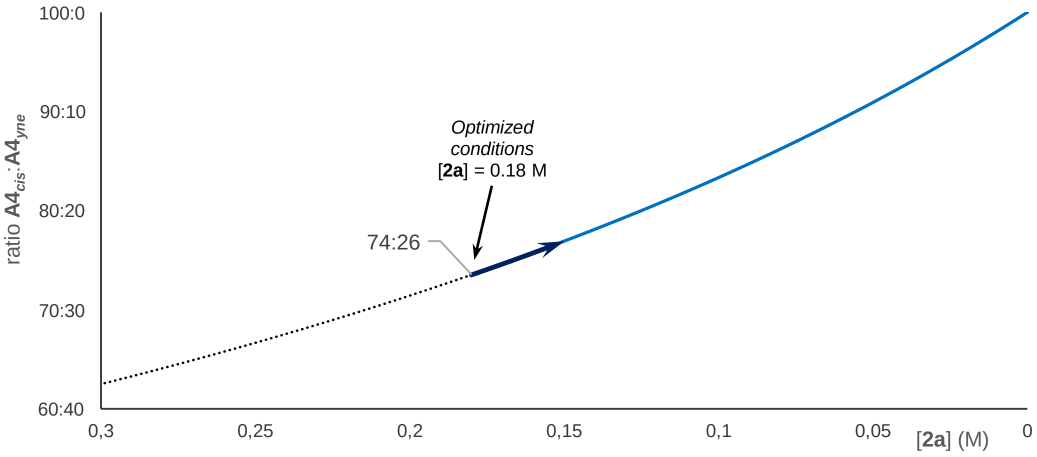

$\frac{v_{A4_{cis}}}{v_{A4_{yne}}}$ calculated as before, resulting in $\frac{v_{A4_{cis}}}{v_{A4_{yne}}} = \frac{1}{[2a]} e^{\frac{\Delta G^{\ddagger}_{yne} - \Delta G^{\ddagger}_{cis}}{RT}}$

Then, $\frac{v_{A4_{cis}}}{v_{A4_{yne}}}$ is normalized and plotted versus decreasing [**2a**].

Although **TS^S(A3A4)$_{yne}$** is slightly lower in energy than **TS^S(A3A4)$_{cis}$** ($\Delta\Delta G^{\ddagger}$ = -0.5 kcal·mol^{-1}), the formation of **A4**$_{cis}$ is favored since the production rate of **A4**$_{yne}$ depends on the concentration of DMAD. At the starting concentration of DMAD, the production rate **A4**$_{cis}$:**A4**$_{yne}$ is 74:26, which is even increased during the reaction as the DMAD is consumed. Considering that this **A4**$_{cis}$:**A4**$_{yne}$ ratio is applied over a 98:2 **A4**$_{trans}$:**A4**$_{cis}$ ratio (computationally calculated), the production of **A4**$_{yne}$ is completely disfavored, and thus, not observed experimentally.

Table S1. Results obtained with different density functionals of the Gibbs energy difference (ΔΔG) between the reaction barriers leading from **A2** to **A4**$_{trans}$ and to **A4**$_{yne}$ and the ratio of reaction rates ($v_{A4trans}/v_{A4yne}$) obtained from the ΔΔG values using the transition state theory.

Functional	Geometry	Gibbs (Hartrees)	ΔΔG (Hartrees)	ΔΔG (kcal/mol)	$v_{A4trans}/v_{A4yne}$ (Eyring)
**B3LYP**	**TSA3A4**$_{trans}$	-5534,936738	0,001776	1,1	1,1
	**TSA3A4**$_{yne}$	-6068,161188			
	**DMAD**	-533,222674	-	-	-
**CAM-B3LYP**	**TSA3A4**$_{trans}$	-5533,857861	-0,000704	-0,4	**10,4**
	**TSA3A4**$_{yne}$	-6066,850448			
	**DMAD**	-532,993291	-	-	-
**M06**	**TSA3A4**$_{trans}$	-5533,157444	0,003183	2,0	0,3
	**TSA3A4**$_{yne}$	-6066,049921			
	**DMAD**	-532,889294	-	-	-
**M06-2X**	**TSA3A4**$_{trans}$	-5533,793530	-0,0037778	-2,4	**162,9**
	**TSA3A4**$_{yne}$	-6066,794407			
	**DMAD**	-533,004654	-	-	-
**BD97-D**	**TSA3A4**$_{trans}$	-5534,912624	0,002094	1,3	0,9
	**TSA3A4**$_{yne}$	-6067,788204			
	**DMAD**	-532,873486	-	-	-
**PW6B95-D3**	**TSA3A4**$_{trans}$	-5539,171139	0,001260	0,8	1,8
	**TSA3A4**$_{yne}$	-6073,011833			
	**DMAD**	-533,839433	-	-	-
**MN15**	**TSA3A4**$_{trans}$	-5532,207342	-0,001715	-1,1	**25,7**
	**TSA3A4**$_{yne}$	-6064,831959			
	**DMAD**	-532,626332	-	-	-
**MN15L**	**TSA3A4**$_{trans}$	-5532,453095	0,009448	5,9	0,0
	**TSA3A4**$_{yne}$	-6065,188954			
	**DMAD**	-532,726411	-	-	-
**ωB97X-D**	**TSA3A4**$_{trans}$	-5534,144687	-0,003358	-2,1	**111,9**
	**TSA3A4**$_{yne}$	-6067,165015			
	**DMAD**	-533,023686	-	-	-
**PBE0**	**TSA3A4**$_{trans}$	-5531,837106	0,002863	1,8	0,4
	**TSA3A4**$_{yne}$	-6064,452781			
	**DMAD**	-532,612812	-	-	-

When choosing the method of calculation, the B3LYP–D3/cc-pVDZ-PP method for geometry optimizations was selected because it was used successfully in many of our previous studies.[1-3] In this project, it was important to obtain a good description of the reaction pathways leading from **A2** to **A4**$_{trans}$ and to **A4**$_{yne}$. The **A2** to **A4**$_{trans}$ pathway should be favoured over the **A2** to **A4**$_{yne}$ since we

did not observe the formation of **A4*yne*** experimentally. Therefore, to refine the B3LYP–D3/cc-pVDZ-PP results with a triple-zeta basis set (cc-pVTZ-PP), single point energy calculations were performed with different functionals (Table S1) looking for those functionals that would give the **A2** to **A4*trans*** pathway with a lower Gibbs energy than the **A2** to **A4yne** pathway. Of the functionals analysed, only the CAM-B3LYP, M06-2X, MN15, and ωB97X-D gave the desired result. The fact that some functionals worked and others did not in this particular example is not surprising since the ΔΔG obtained from most of the functionals analyzed is ± 2 kcal/mol. Such a small difference is within the expected error in density functional theory. M06-2X was discarded because, in general, this functional is not recommended when transition metals are involved in the reactions studied, and CAM-B3LYP was also discarded because the Gibbs energy difference between the two pathways was close to zero. In the end, ωB97X-D rather than MN15 was chosen due to the more negative ΔΔG value of the former.

Computational data

Cartesian coordinates of all optimized species can be downloaded from:

DOI: 10.19061/iochem-bd-4-46

^{1}H and ^{13}C NMR spectra

Imidazolium salt L4

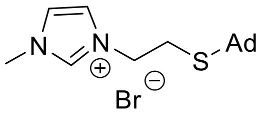

^{1}H NMR (400 MHz, $CDCl_3$)

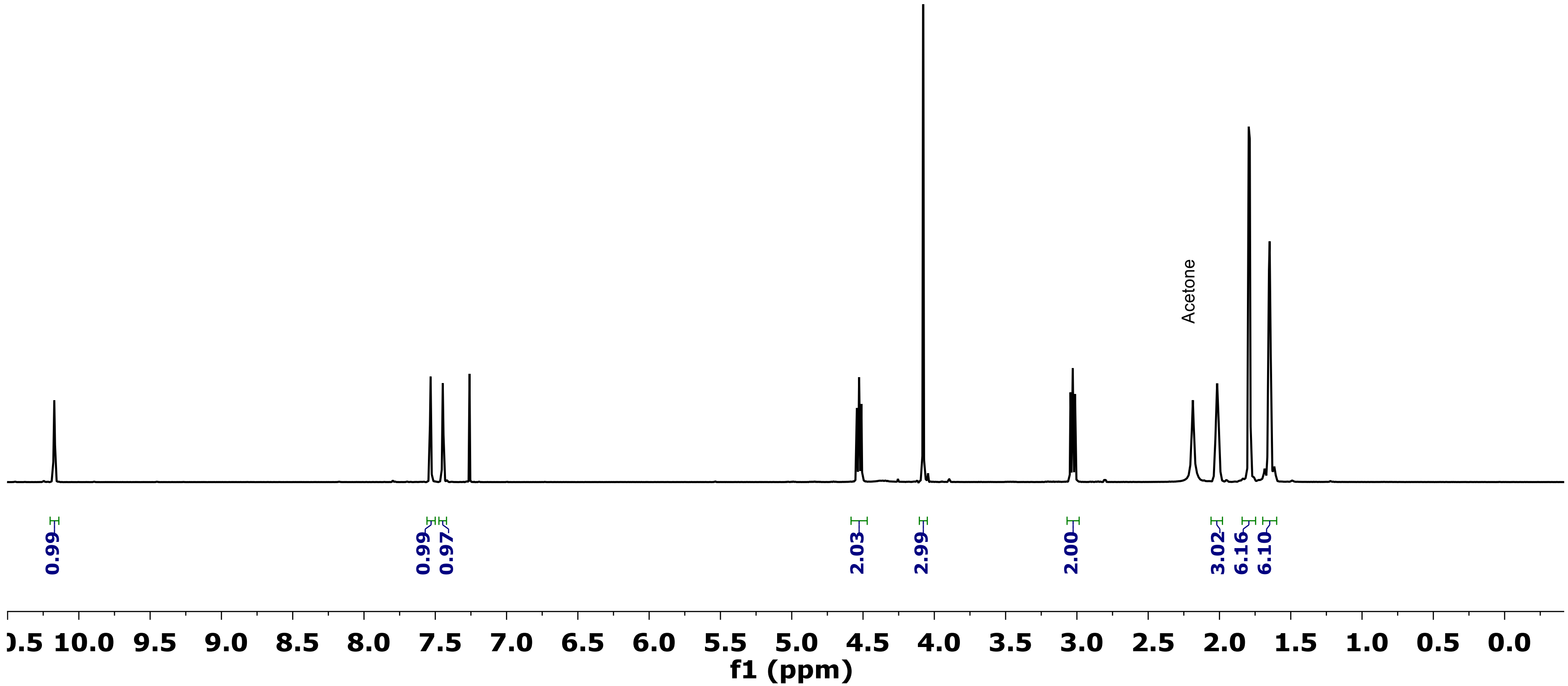

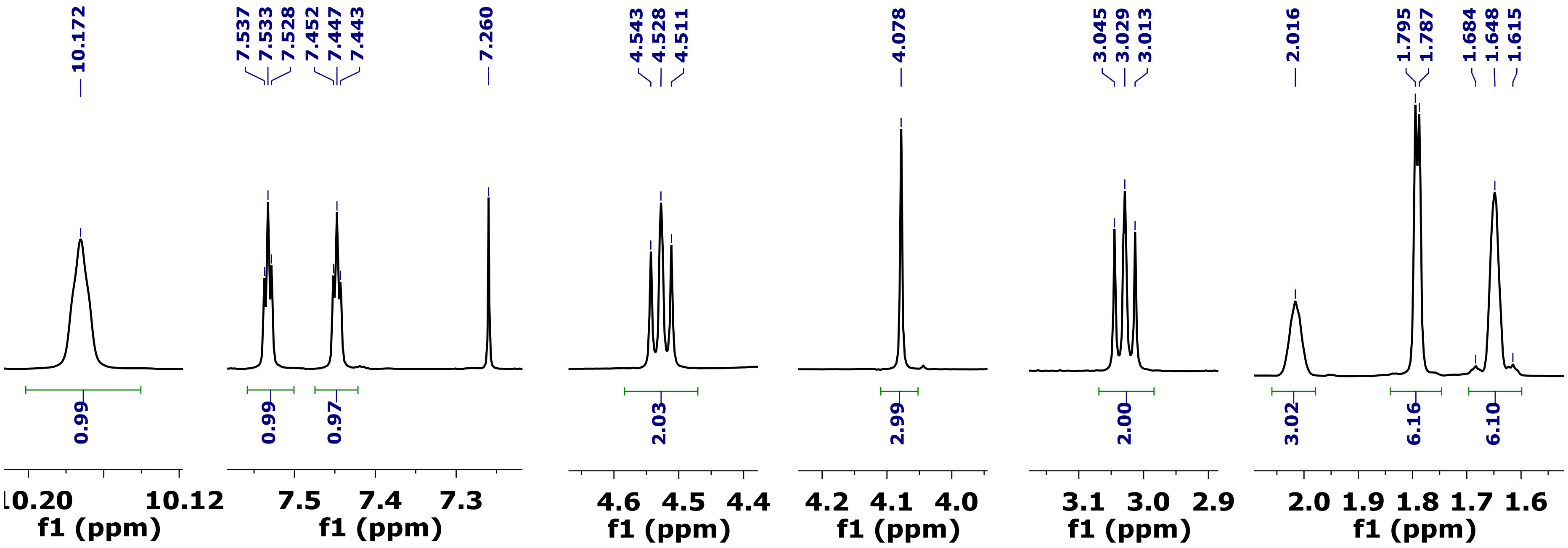
10.172
7.537
7.533
7.528
7.452
7.447
7.443
7.260
4.543
4.528
4.511
4.078
3.045
3.029
3.013
2.016
1.795
1.787
1.684
1.648
1.615
0.99
0.99
0.97
2.03
2.99
2.00
3.02
6.16
6.10
10.20
10.12
7.5
7.4
7.3
4.6
4.5
4.4
4.2
4.1
4.0
3.1
3.0
2.9
2.0
1.9
1.8
1.7
1.6
f1 (ppm)

^{13}C NMR (101 MHz, $CDCl_3$)

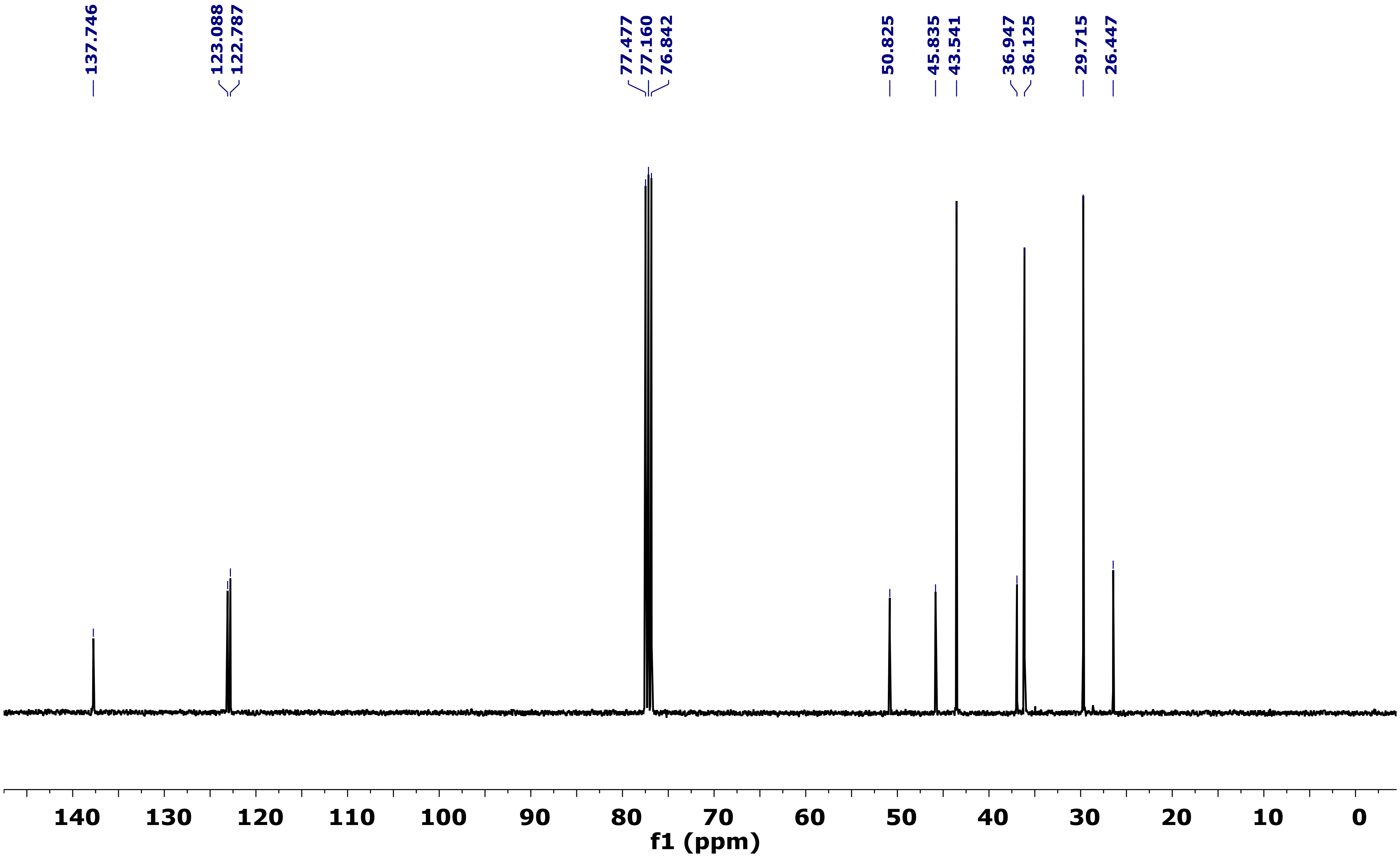

Rhodium-NHC complex RhL4

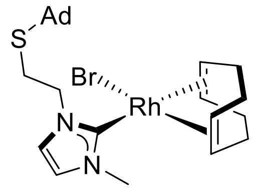

^{1}H NMR (400 MHz, $CDCl_3$)

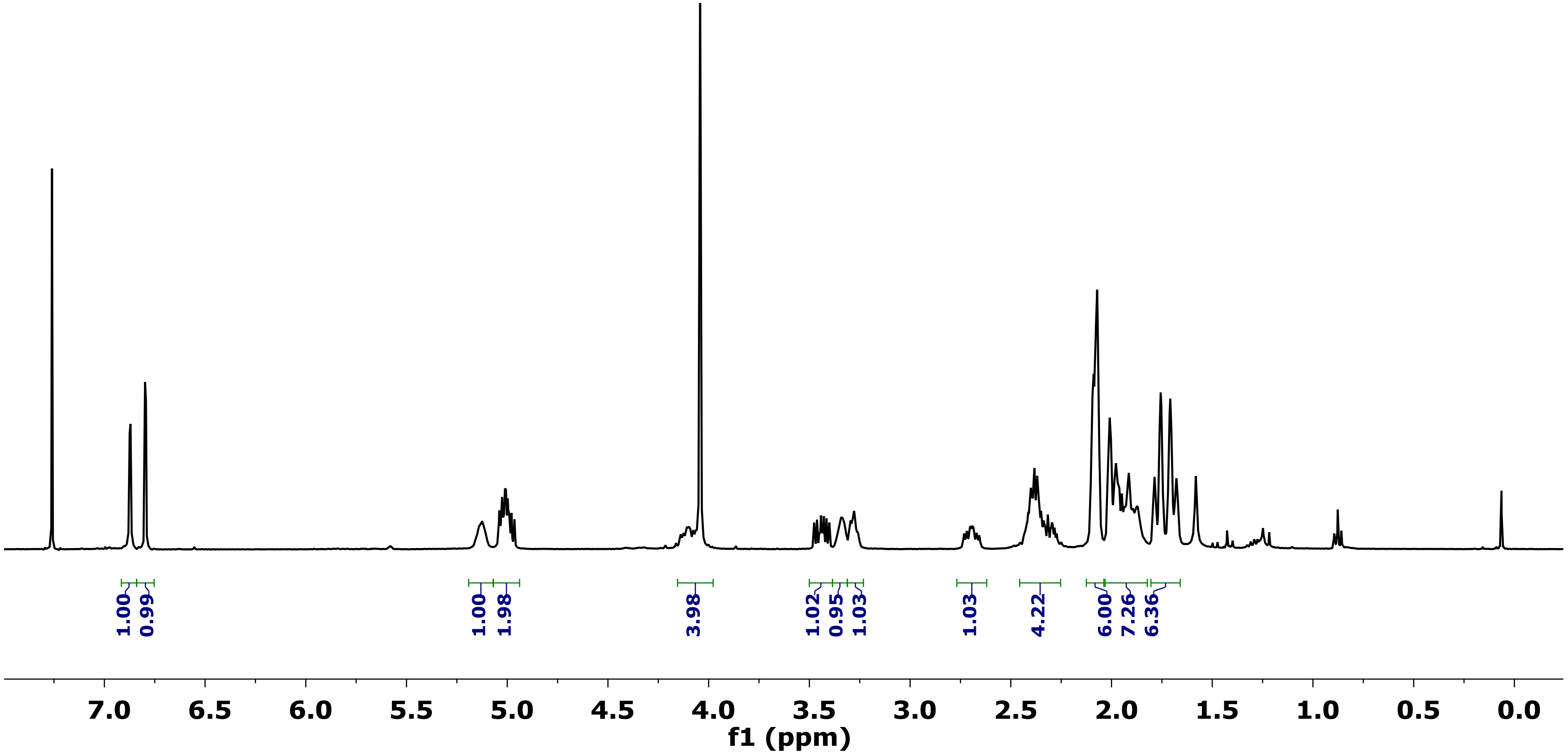

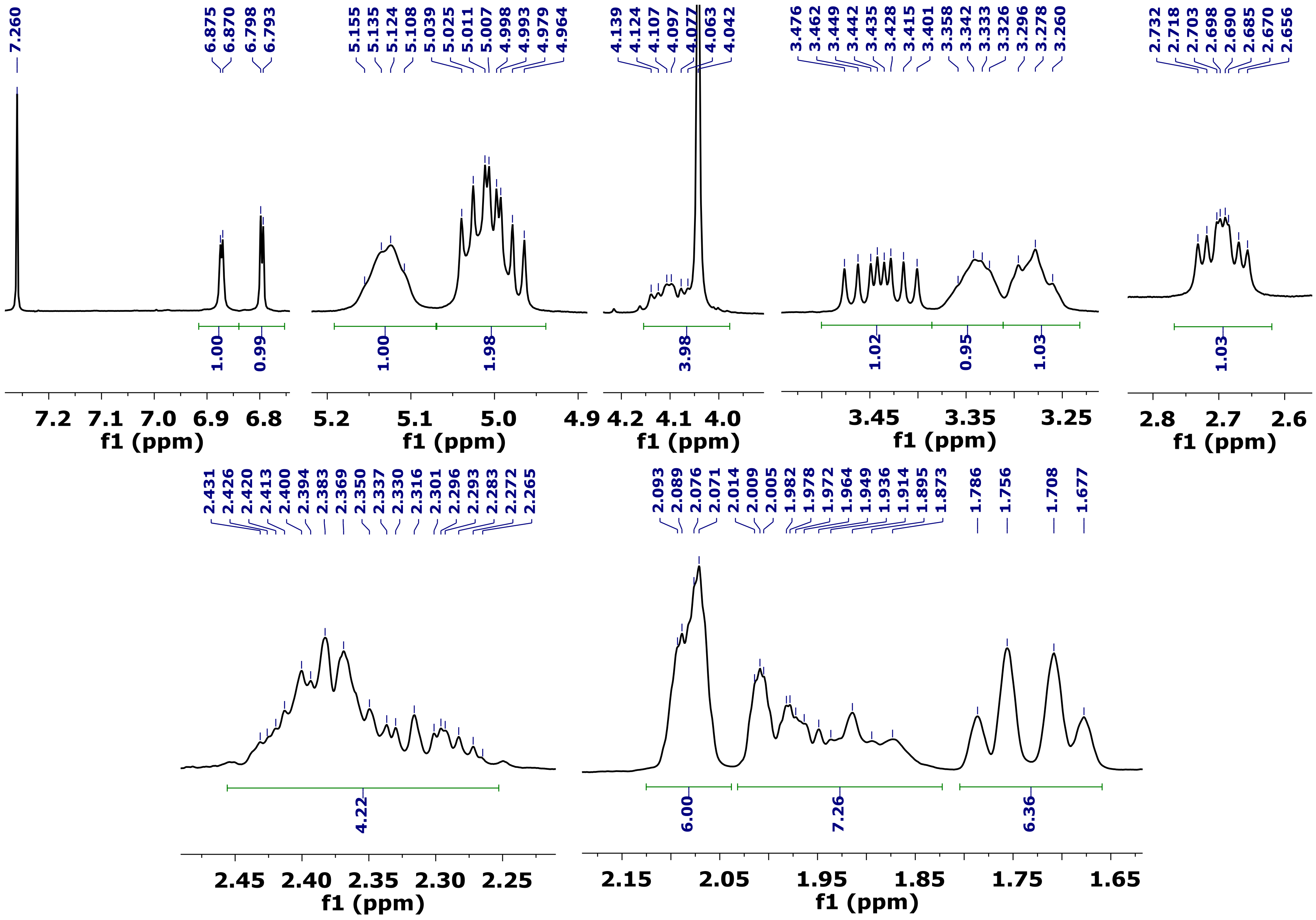
7.260
6.875
6.870
6.798
6.793
1.00
0.99
7.2 7.1 7.0 6.9 6.8
f1 (ppm)
5.155
5.135
5.124
5.108
5.039
5.025
5.011
5.007
4.998
4.993
4.979
4.964
1.00
1.98
5.2 5.1 5.0 4.9
f1 (ppm)
4.139
4.124
4.107
4.097
4.077
4.063
4.042
3.98
4.2 4.1 4.0
f1 (ppm)
3.476
3.462
3.449
3.442
3.435
3.428
3.415
3.401
3.358
3.342
3.333
3.326
3.296
3.278
3.260
1.02
0.95
1.03
3.45 3.35 3.25
f1 (ppm)
2.732
2.718
2.703
2.698
2.690
2.685
2.670
2.656
1.03
2.8 2.7 2.6
f1 (ppm)
2.431
2.426
2.420
2.413
2.400
2.394
2.383
2.369
2.350
2.337
2.330
2.316
2.301
2.296
2.293
2.283
2.272
2.265
4.22
2.45 2.40 2.35 2.30 2.25
f1 (ppm)
2.093
2.089
2.076
2.071
2.014
2.009
2.005
1.982
1.978
1.972
1.964
1.949
1.936
1.914
1.895
1.873
1.786
1.756
1.708
1.677
6.00
7.26
6.36
2.15 2.05 1.95 1.85 1.75 1.65
f1 (ppm)

^{13}C NMR (101 MHz, $CDCl_3$)

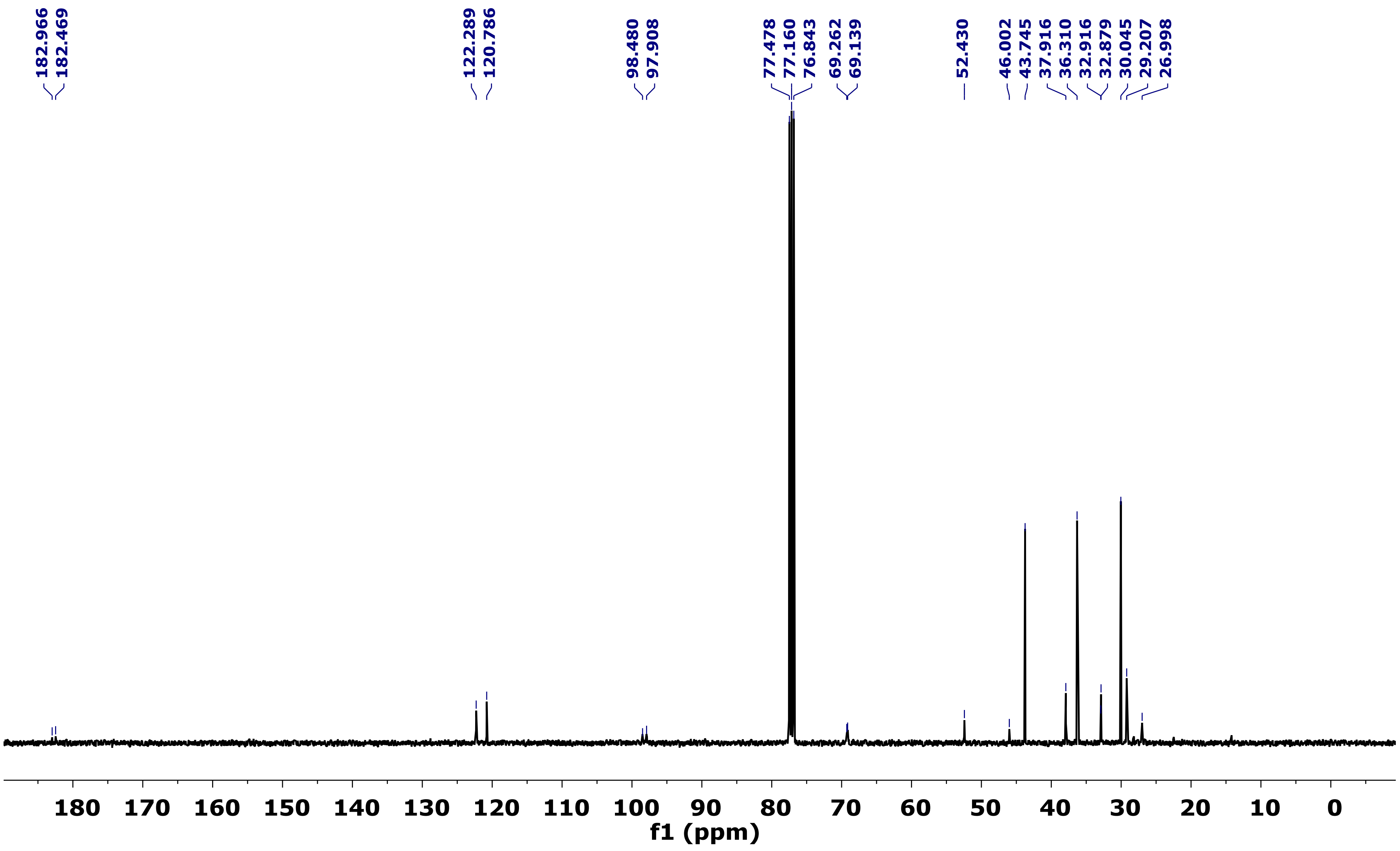

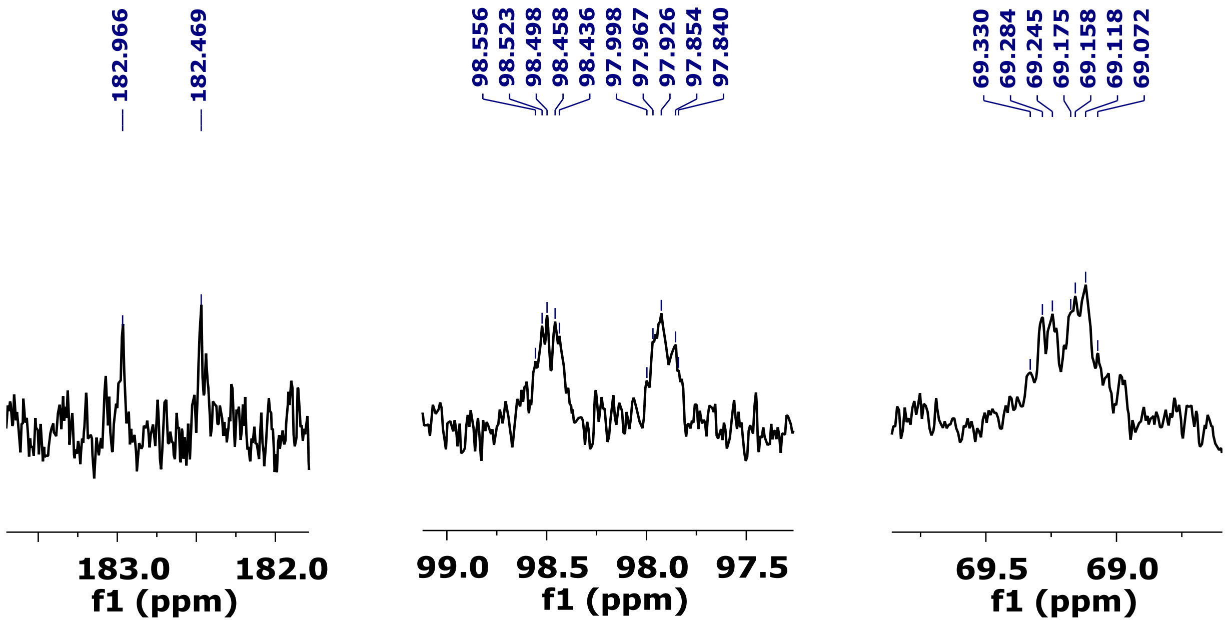
182.966
182.469
183.0
182.0
f1 (ppm)
98.556
98.523
98.498
98.458
98.436
97.998
97.967
97.926
97.854
97.840
99.0
98.5
98.0
97.5
f1 (ppm)
69.330
69.284
69.245
69.175
69.158
69.118
69.072
69.5
69.0
f1 (ppm)

2D NMR HSQC

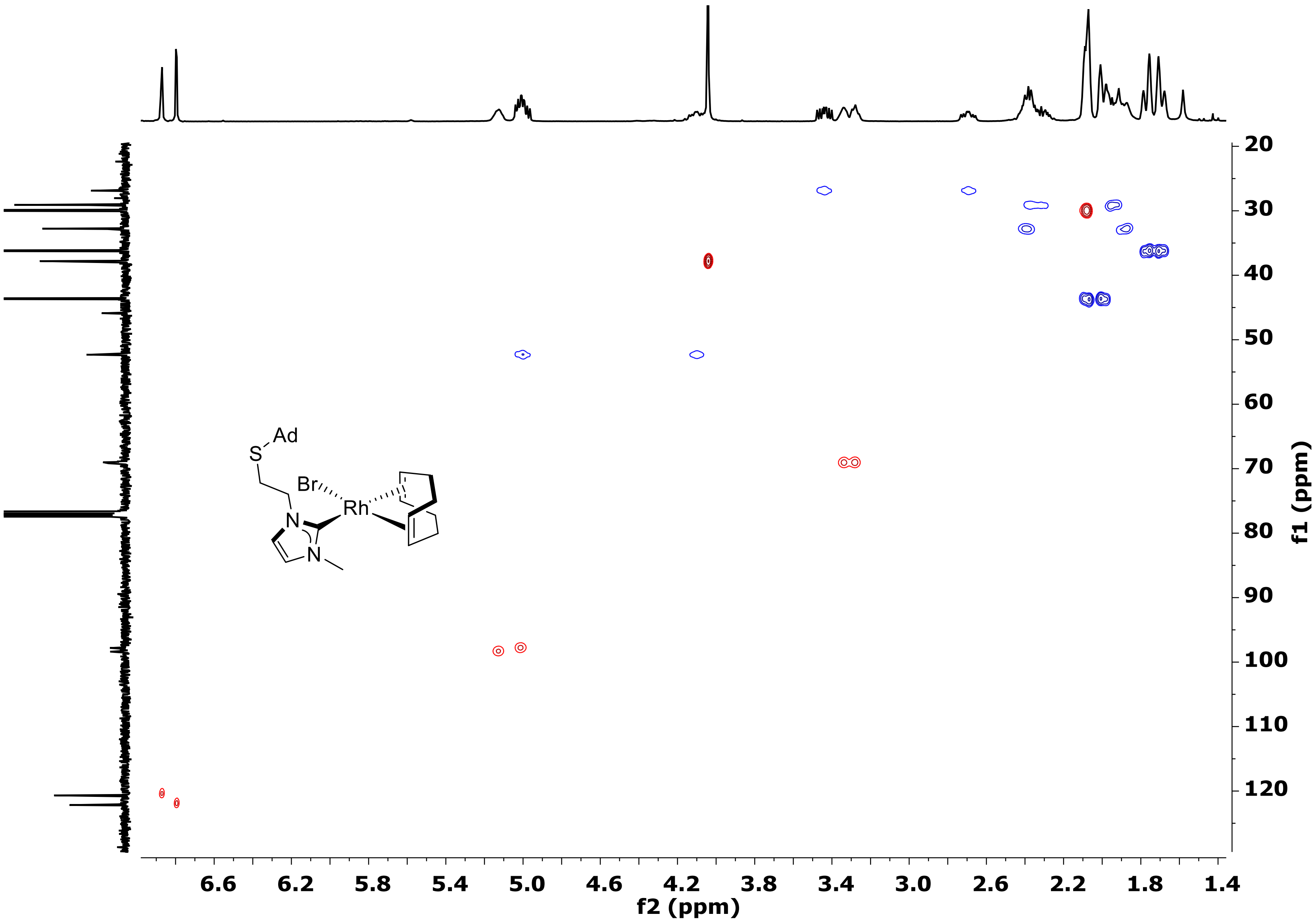
S
Ad
Br
Rh
N
N
6.6
6.2
5.8
5.4
5.0
4.6
4.2
3.8
3.4
3.0
2.6
2.2
1.8
1.4
f2 (ppm)
20
30
40
50
60
70
80
90
100
110
120
f1 (ppm)

2D NMR COSY

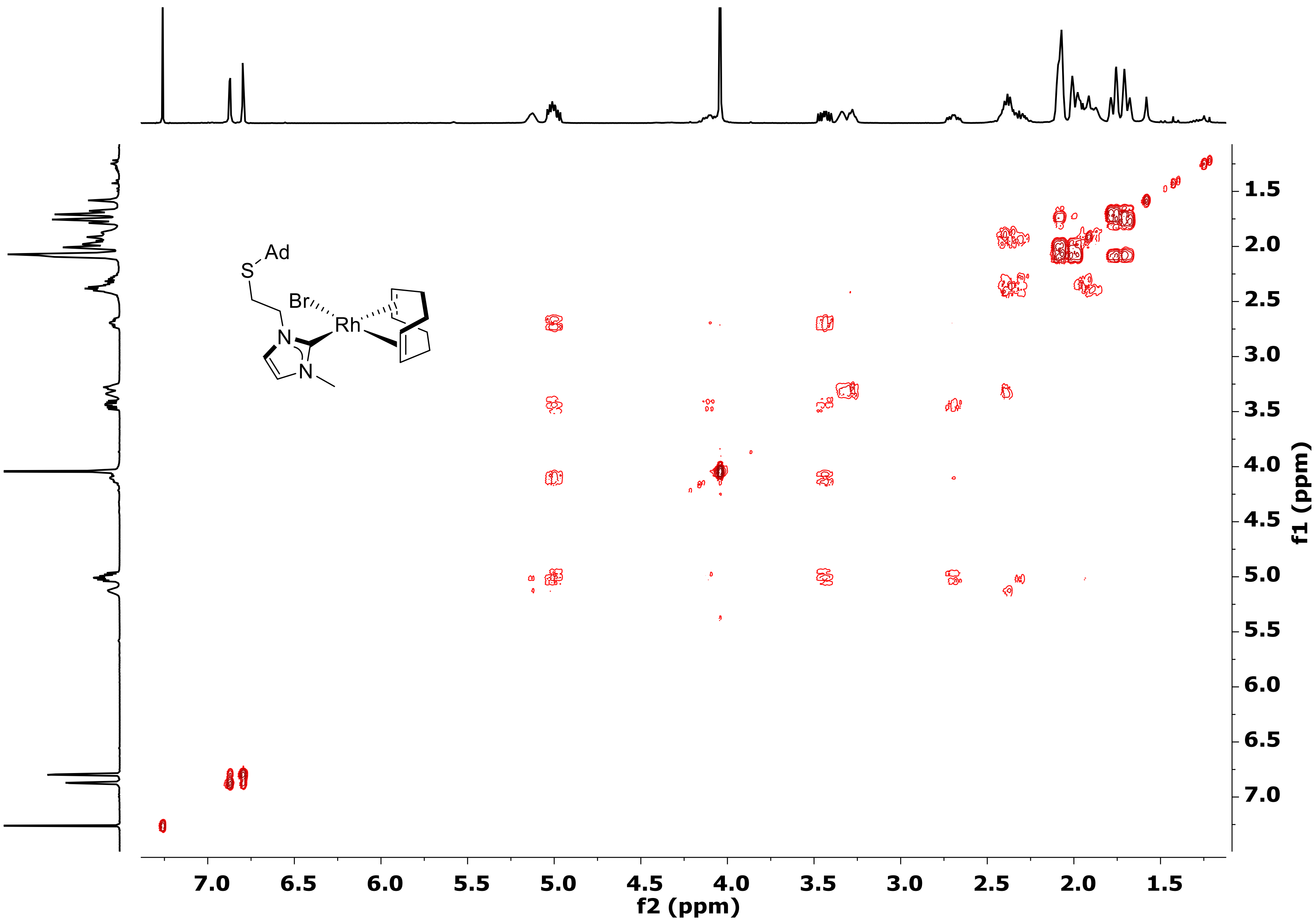

Product 3a

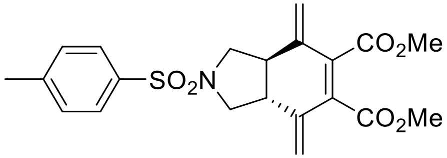

^{1}H NMR (400 MHz, $CDCl_3$)

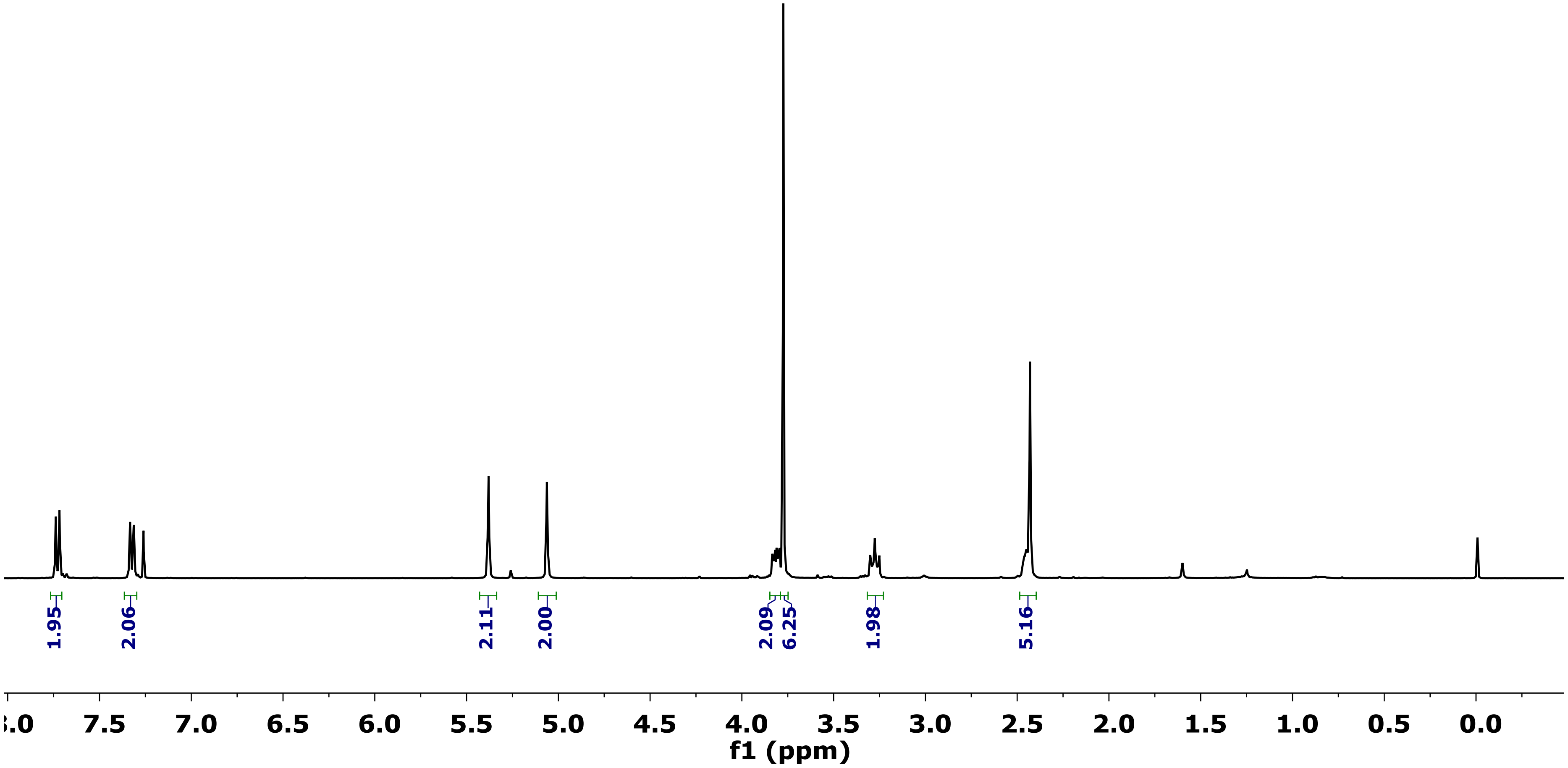

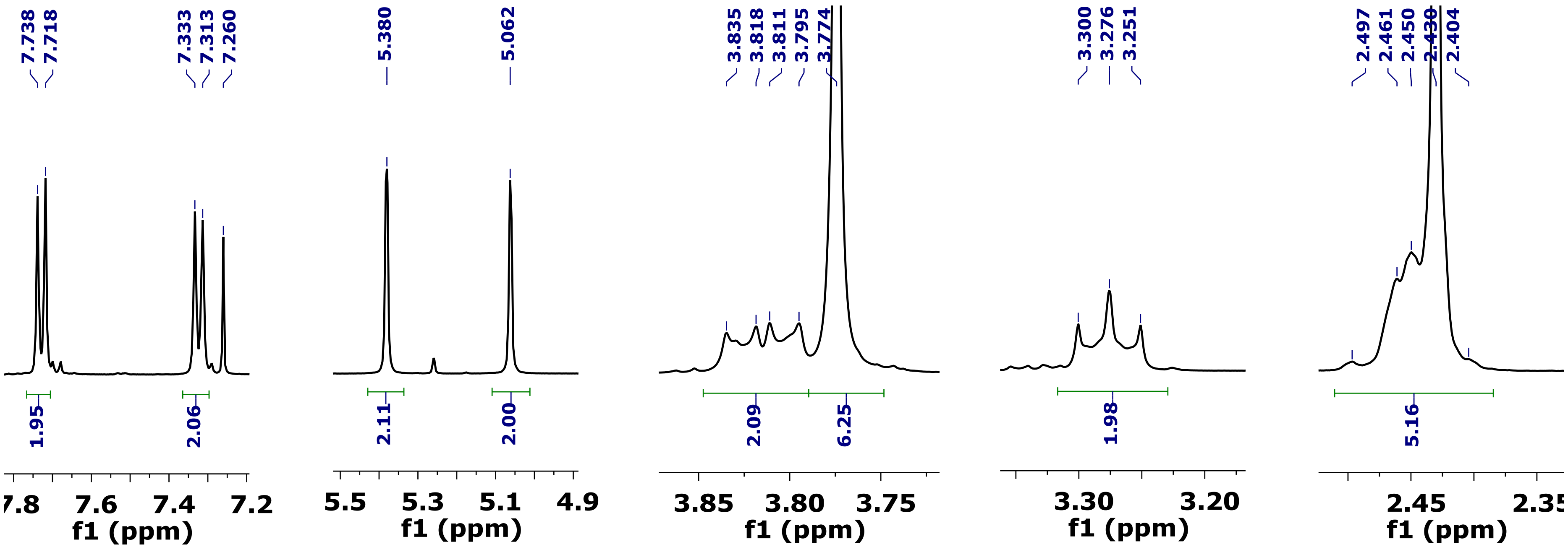
7.738
7.718
7.333
7.313
7.260
1.95
2.06
7.8 7.6 7.4 7.2
f1 (ppm)
5.380
5.062
2.11
2.00
5.5 5.3 5.1 4.9
f1 (ppm)
3.835
3.818
3.811
3.795
3.774
2.09
6.25
3.85 3.80 3.75
f1 (ppm)
3.300
3.276
3.251
1.98
3.30 3.20
f1 (ppm)
2.497
2.461
2.450
2.430
2.404
5.16
2.45
f1 (ppm)

^{13}C NMR (101 MHz, $CDCl_3$)

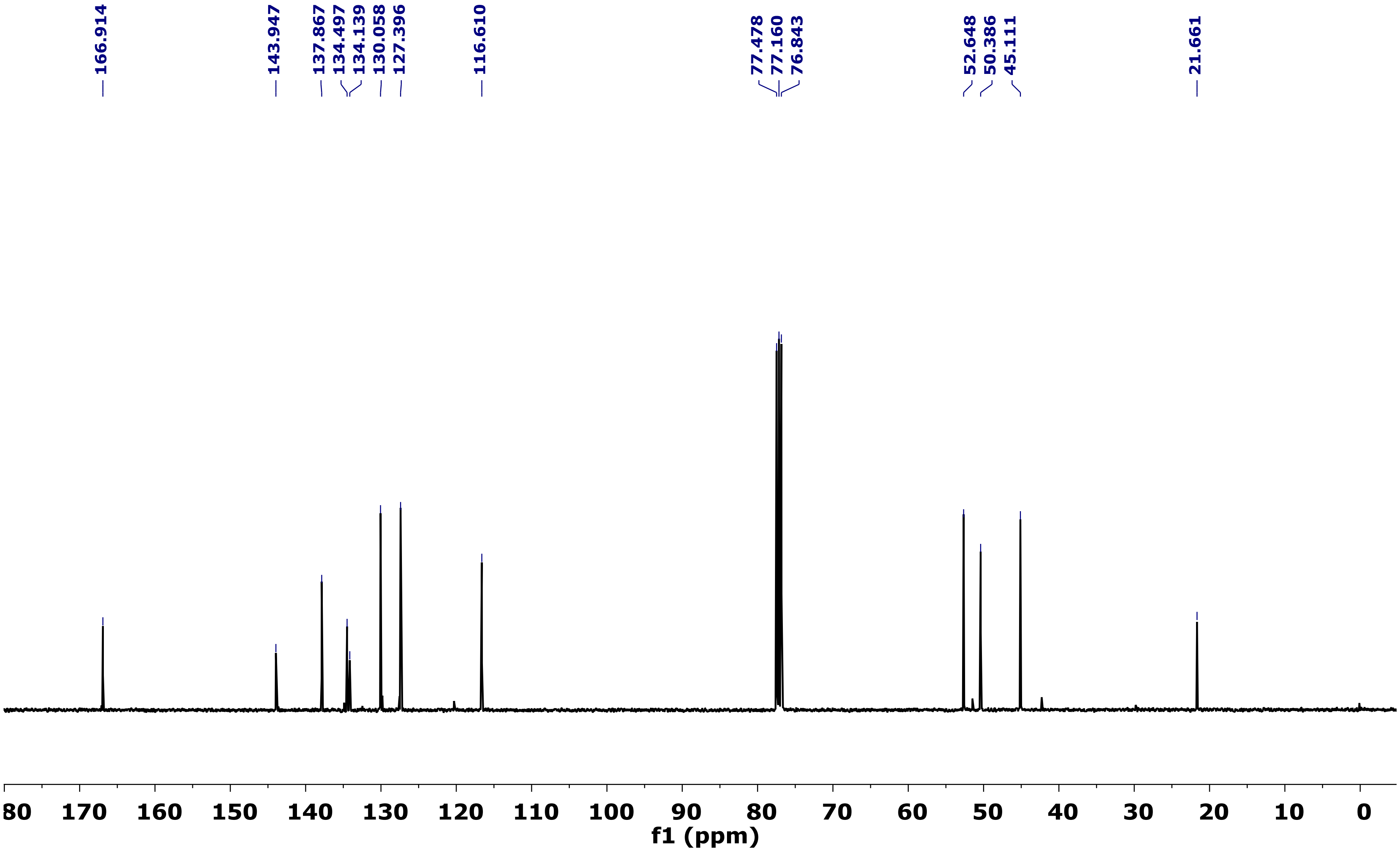

2D NMR HSQC

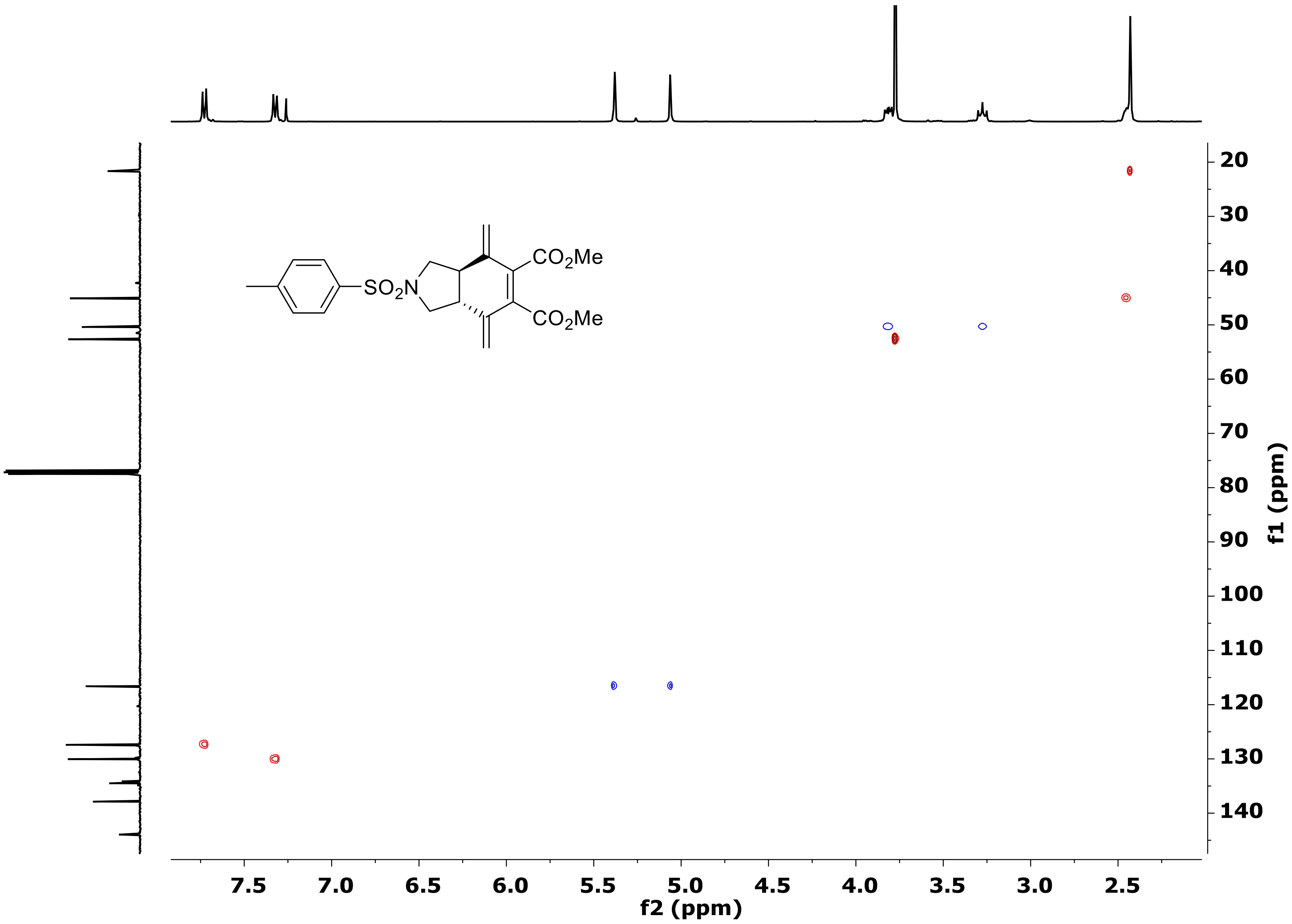

SO2N
CO2Me
CO2Me
20
30
40
50
60
70
80
90
100
110
120
130
140
f1 (ppm)
7.5
7.0
6.5
6.0
5.5
5.0
4.5
4.0
3.5
3.0
2.5
f2 (ppm)

2D NMR HMBC

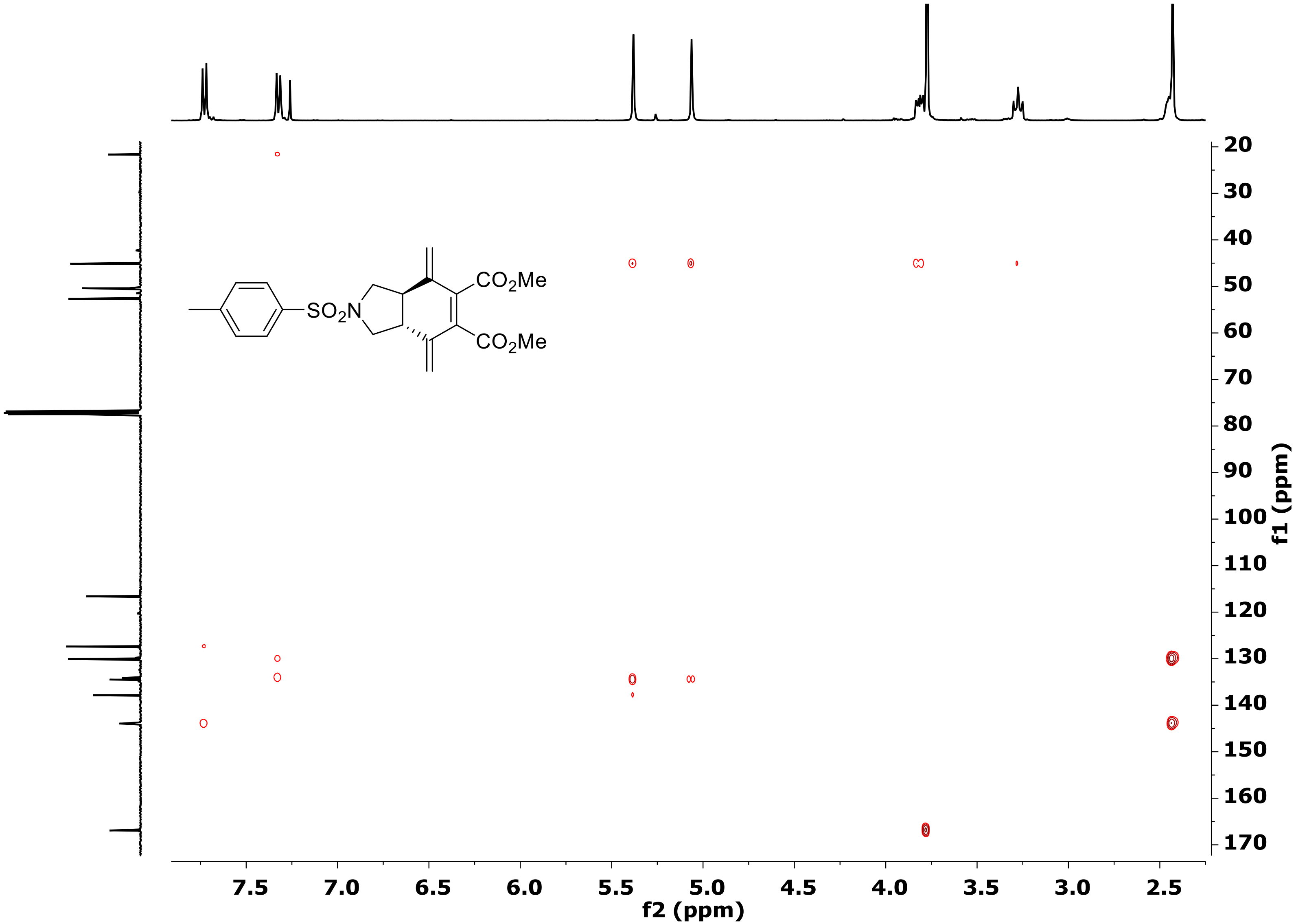
SO2N
CO2Me
CO2Me
f2 (ppm)
f1 (ppm)
7.5
7.0
6.5
6.0
5.5
5.0
4.5
4.0
3.5
3.0
2.5
20
30
40
50
60
70
80
90
100
110
120
130
140
150
160
170

2D NMR COSY

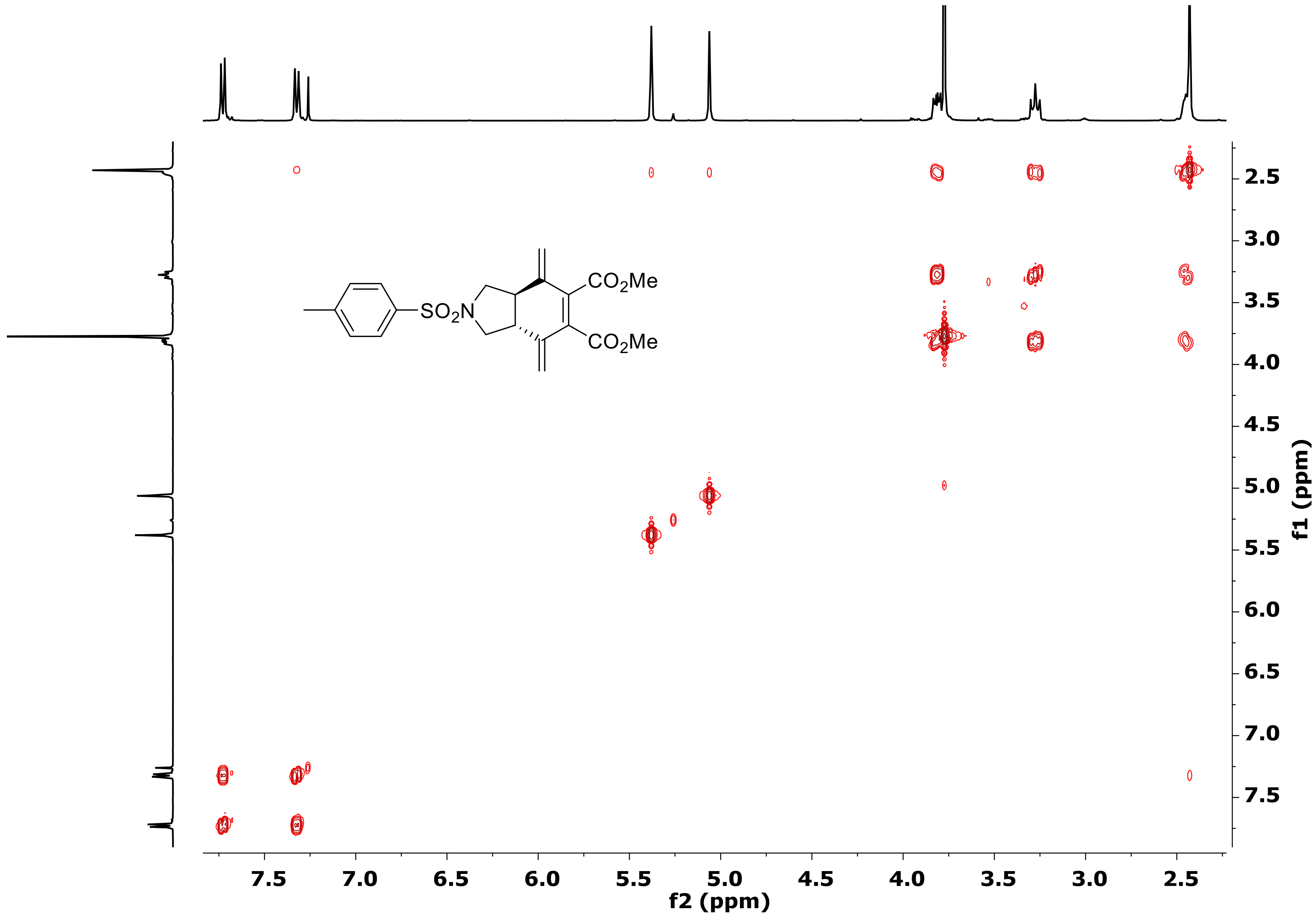

2D NMR NOESY

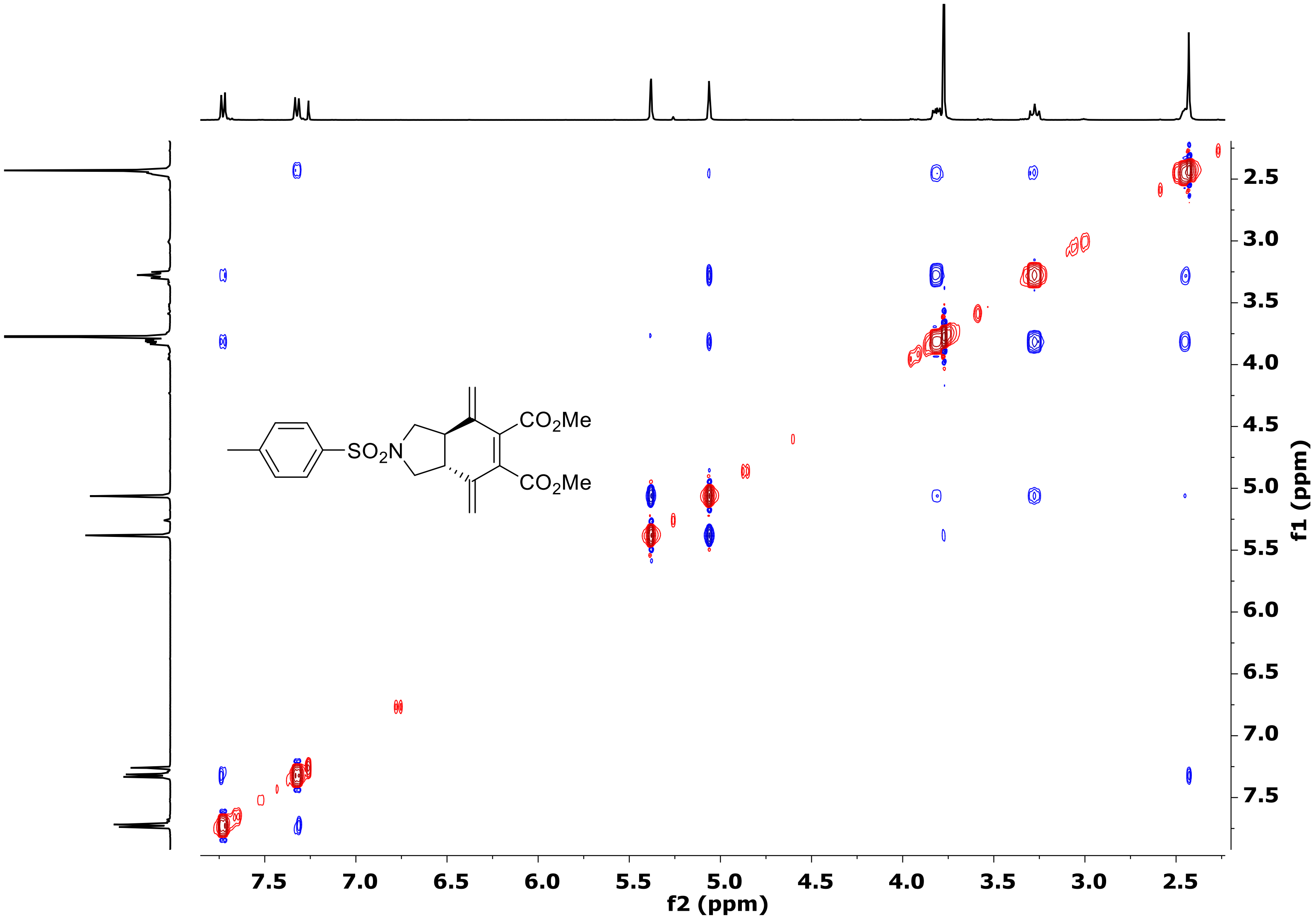

Product 3b

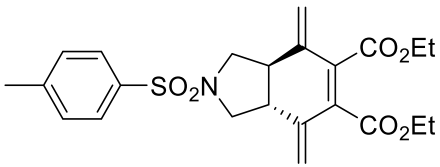

^{1}H NMR (400 MHz, $CDCl_3$)

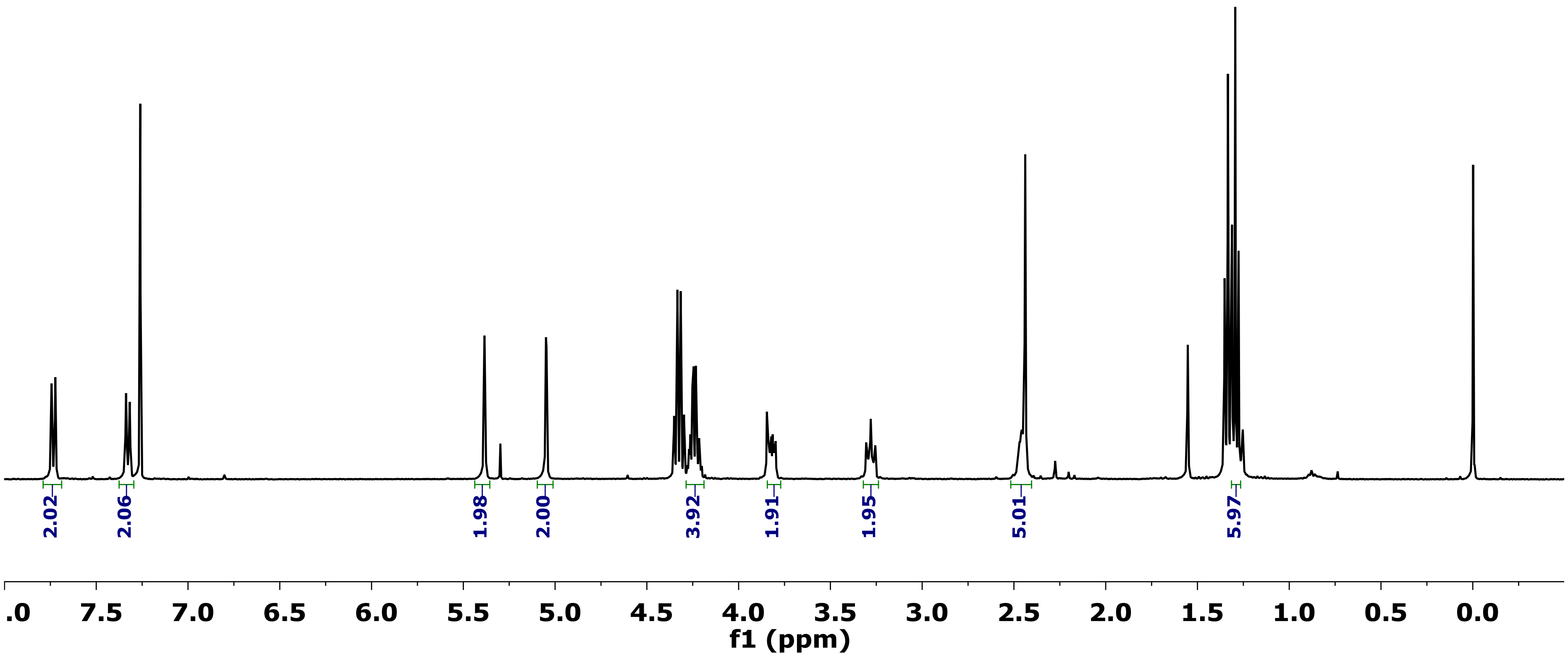

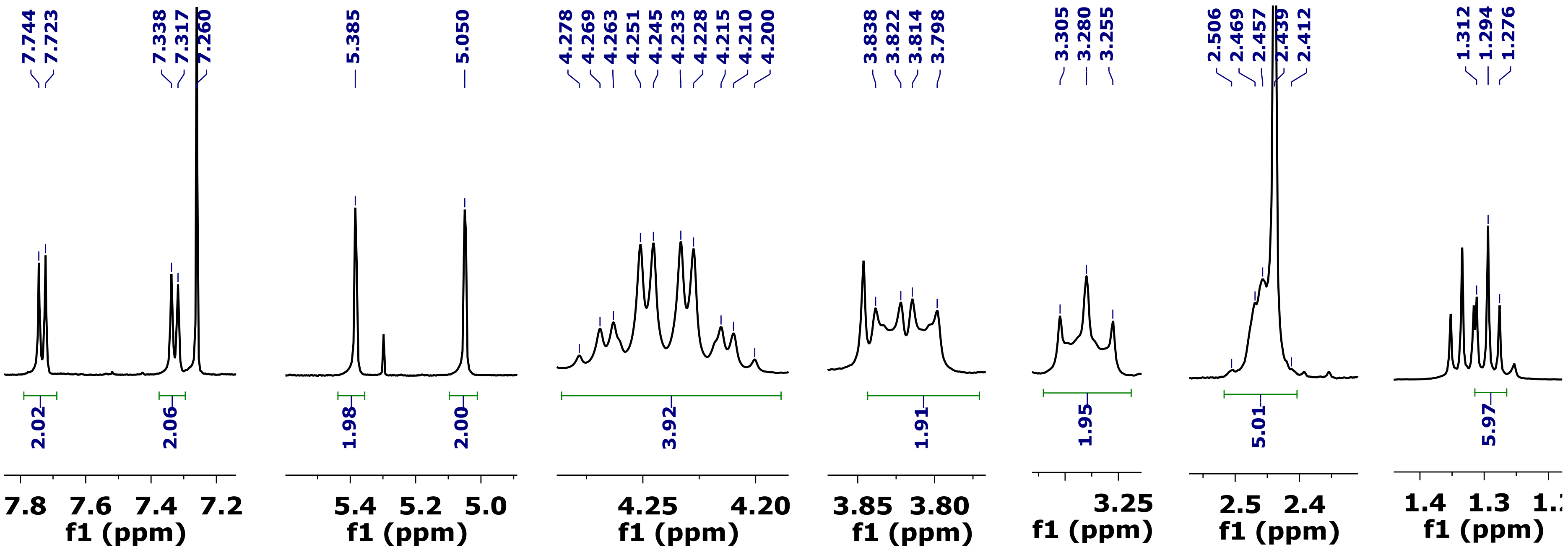
7.744
7.723
7.338
7.317
7.260
5.385
5.050
4.278
4.269
4.263
4.251
4.245
4.233
4.228
4.215
4.210
4.200
3.838
3.822
3.814
3.798
3.305
3.280
3.255
2.506
2.469
2.457
2.439
2.412
1.312
1.294
1.276
2.02
2.06
1.98
2.00
3.92
1.91
1.95
5.01
5.97
7.8 7.6 7.4 7.2
5.4 5.2 5.0
4.25 4.20
3.85 3.80
3.25
2.5 2.4
1.4 1.3
f1 (ppm)

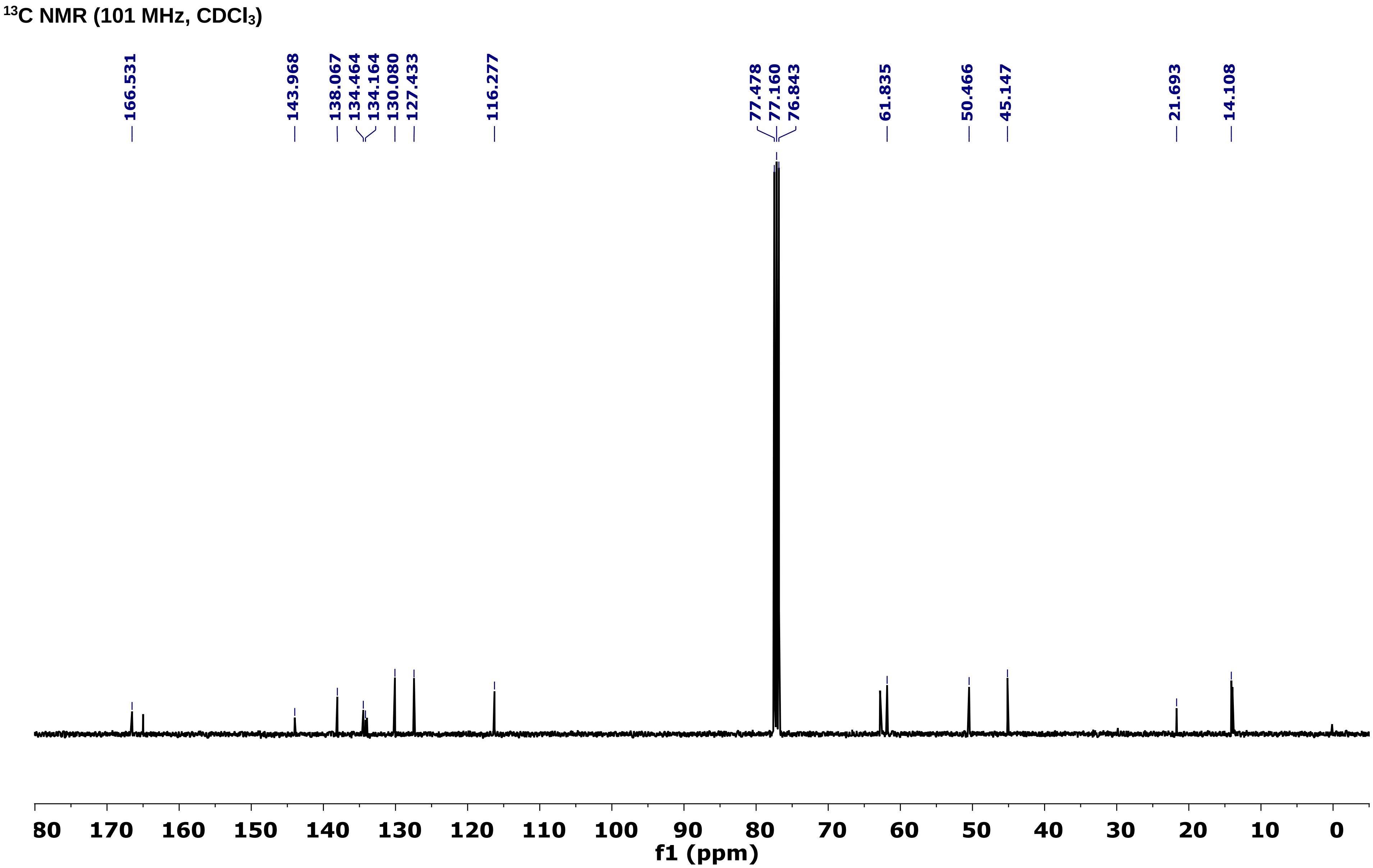

^{13}C NMR (101 MHz, CDCl$_3$)
166.531
143.968
138.067
134.464
134.164
130.080
127.433
116.277
77.478
77.160
76.843
61.835
50.466
45.147
21.693
14.108
80 170 160 150 140 130 120 110 100 90 80 70 60 50 40 30 20 10 0
f1 (ppm)

Product 3c

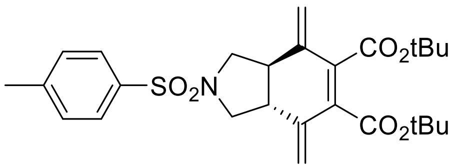

^{1}H NMR (400 MHz, CDCl$_3$)

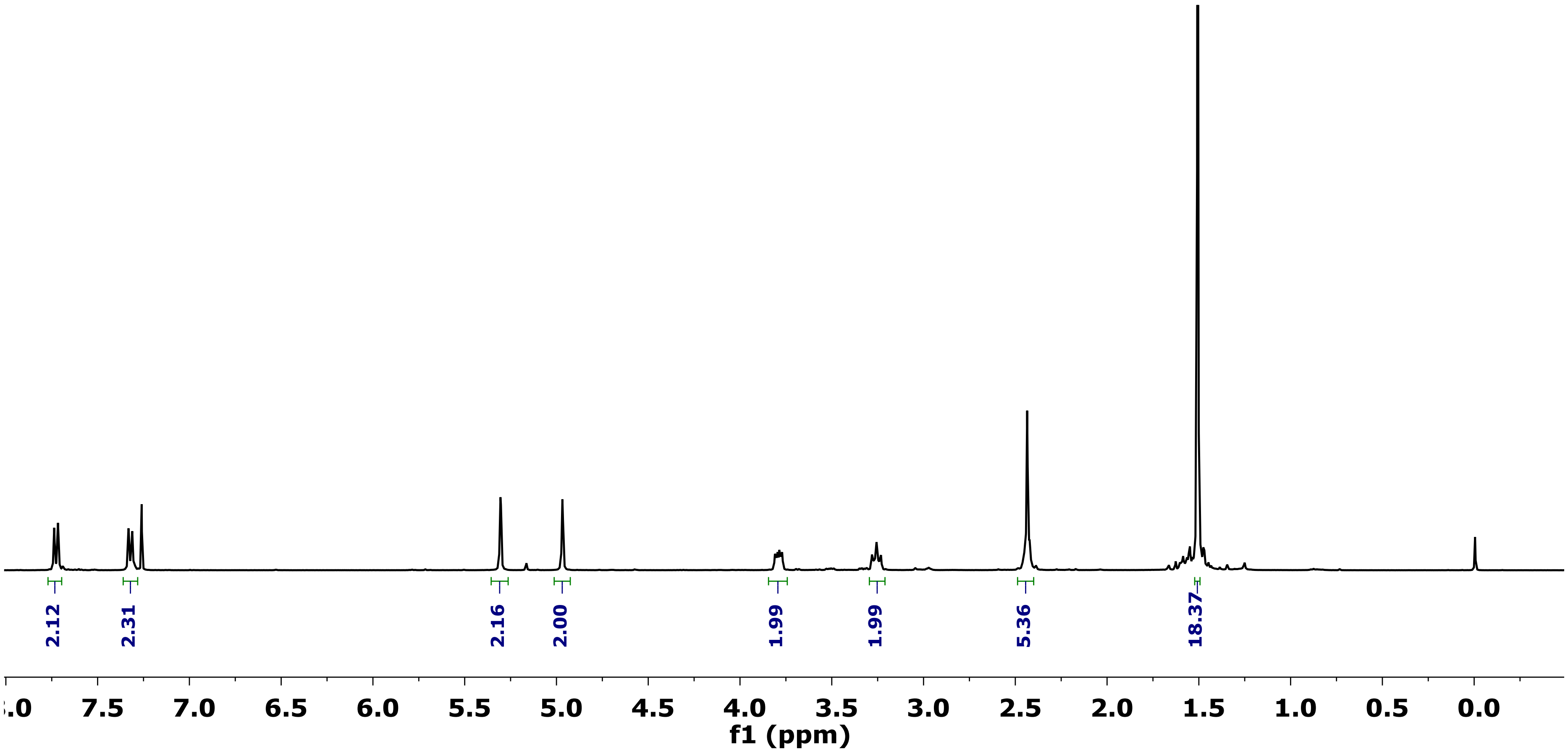

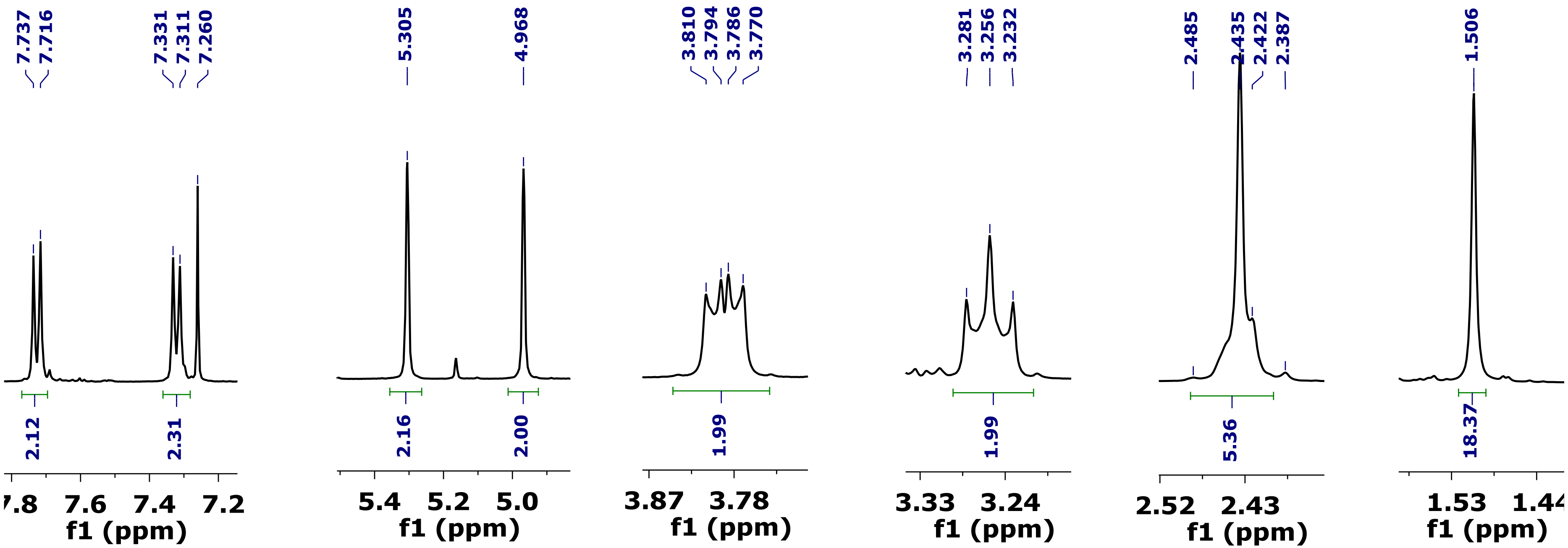
7.737
7.716
7.331
7.311
7.260
2.12
2.31
7.8 7.6 7.4 7.2
f1 (ppm)
5.305
4.968
2.16
2.00
5.4 5.2 5.0
f1 (ppm)
3.810
3.794
3.786
3.770
1.99
3.87 3.78
f1 (ppm)
3.281
3.256
3.232
1.99
3.33 3.24
f1 (ppm)
2.485
2.435
2.422
2.387
5.36
2.52 2.43
f1 (ppm)
1.506
18.37
1.53 1.44
f1 (ppm)

^{13}C NMR (101 MHz, $CDCl_3$)

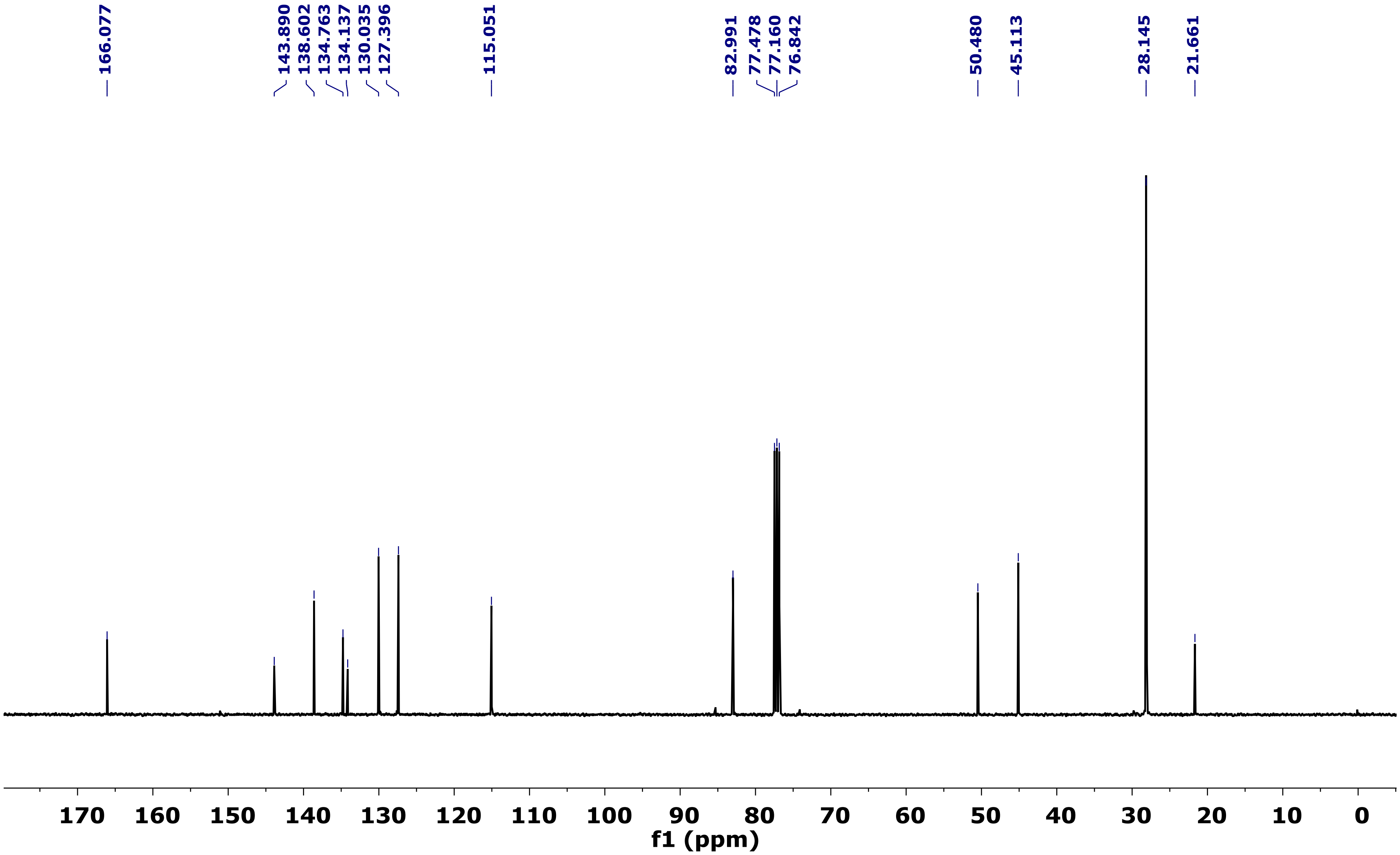

Product 3d

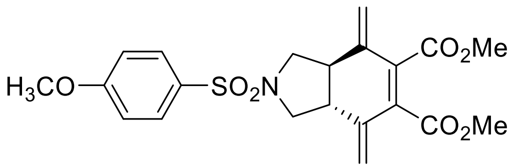

^{1}H NMR (400 MHz, $CDCl_3$)

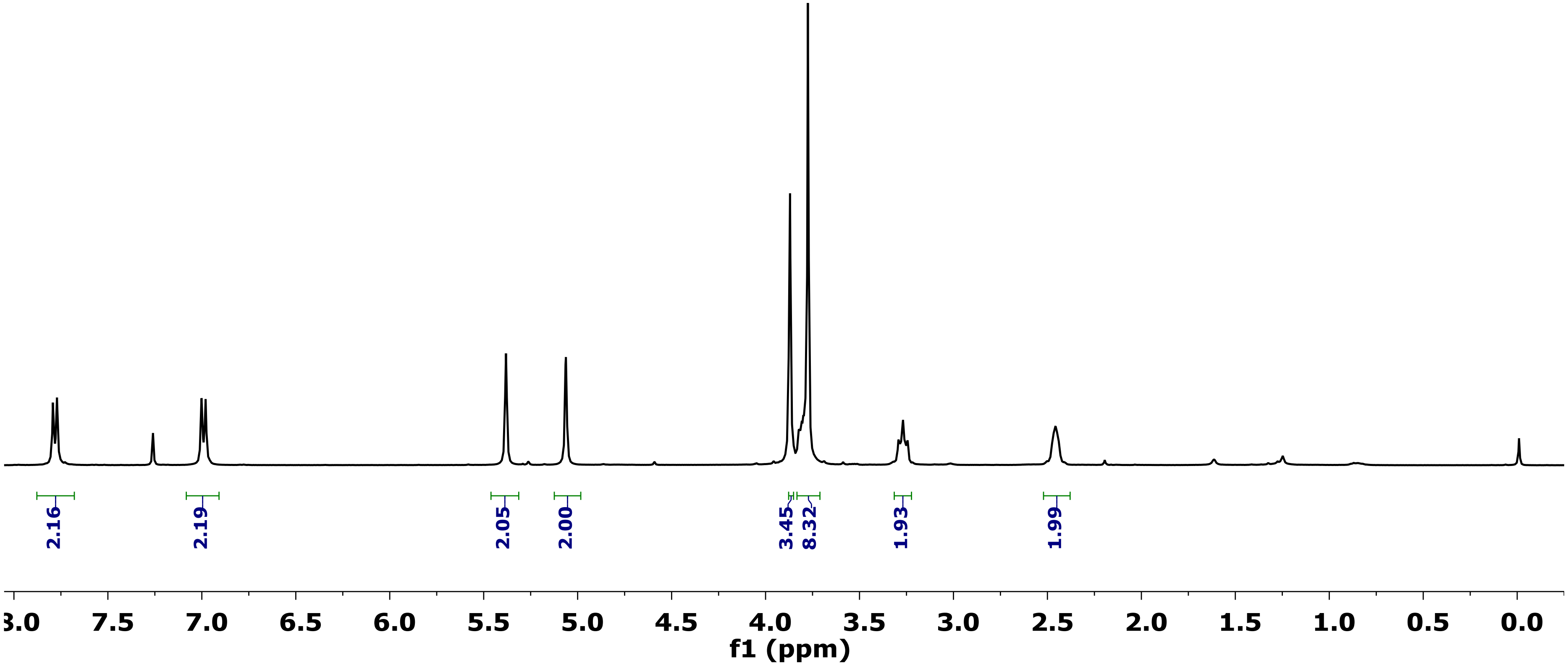

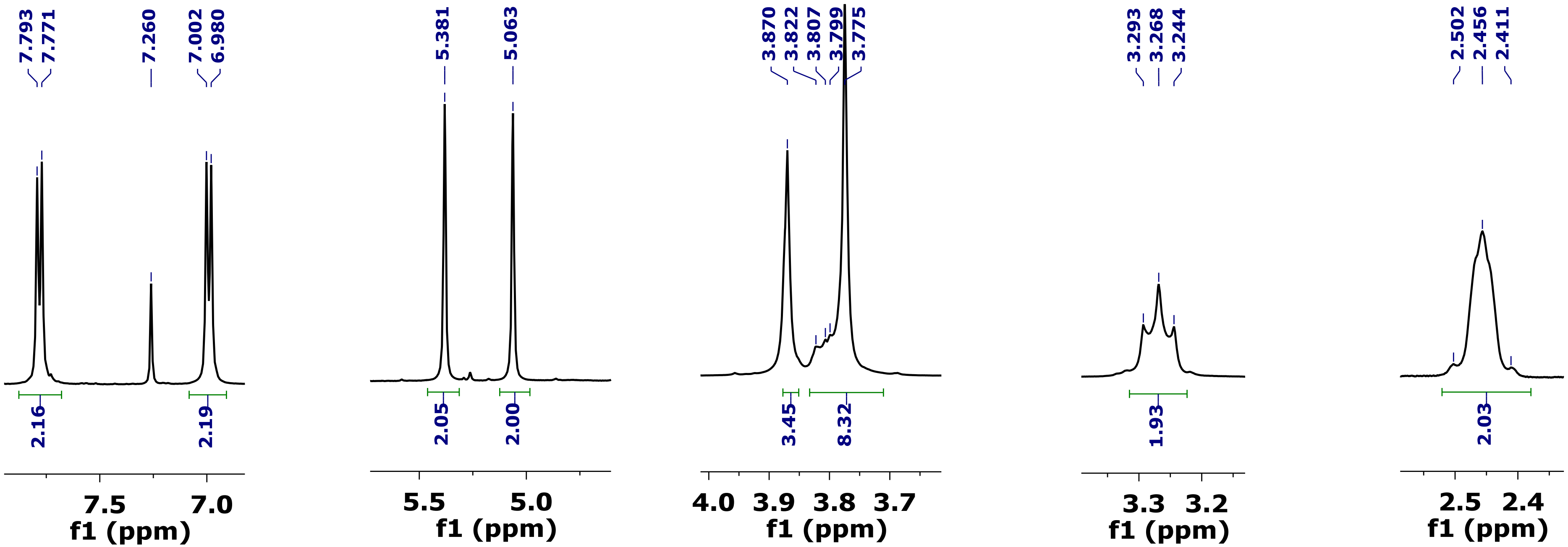
7.793
7.771
7.260
7.002
6.980
2.16
2.19
7.5
7.0
f1 (ppm)
5.381
5.063
2.05
2.00
5.5
5.0
f1 (ppm)
3.870
3.822
3.807
3.799
3.775
3.45
8.32
4.0
3.9
3.8
3.7
f1 (ppm)
3.293
3.268
3.244
1.93
3.3
3.2
f1 (ppm)
2.502
2.456
2.411
2.03
2.5
2.4
f1 (ppm)

^{13}C NMR (101 MHz, $CDCl_3$)

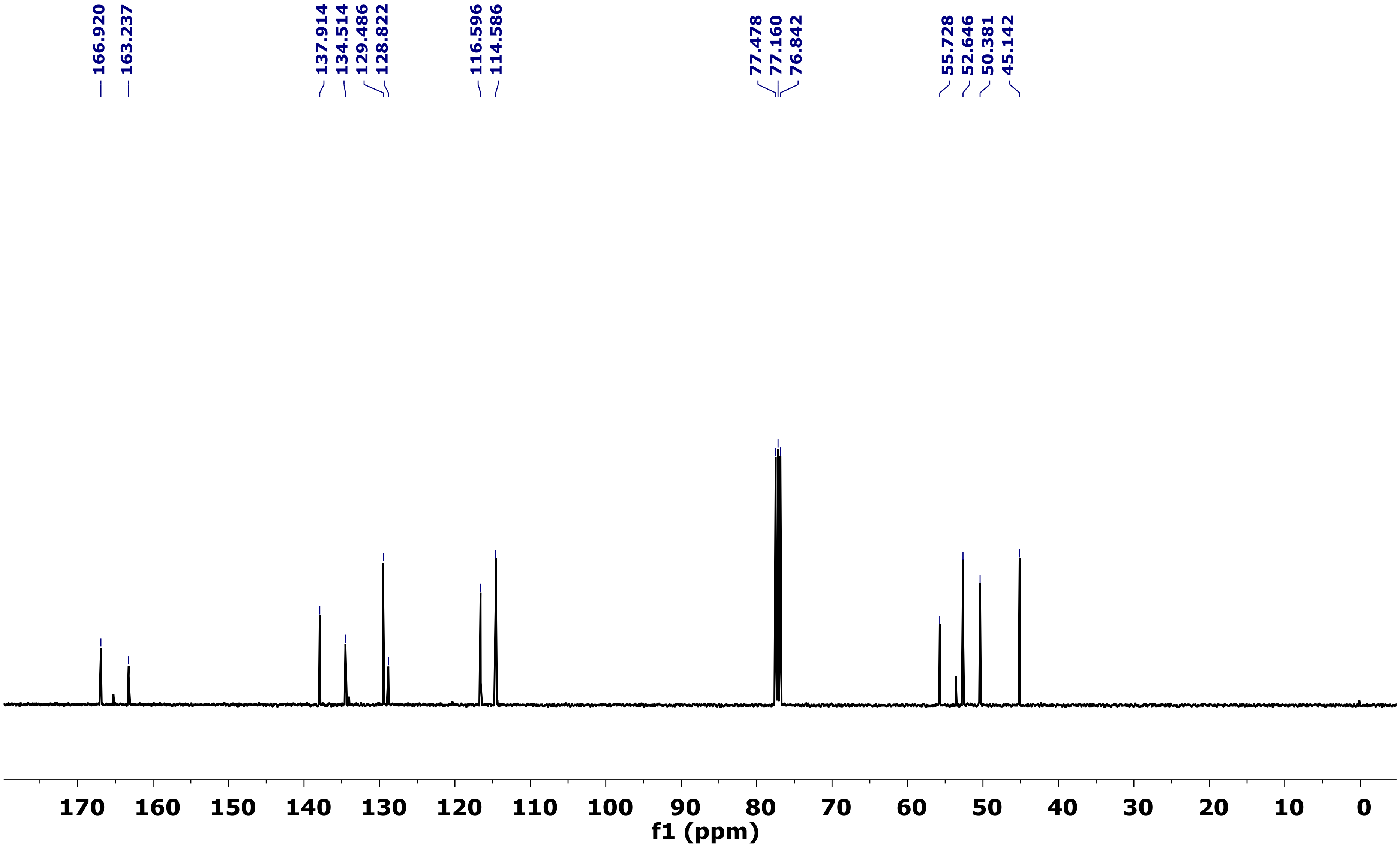

Product 3e

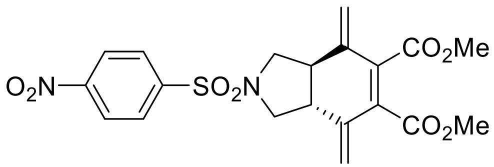

^1H NMR (400 MHz, $CDCl_3$)

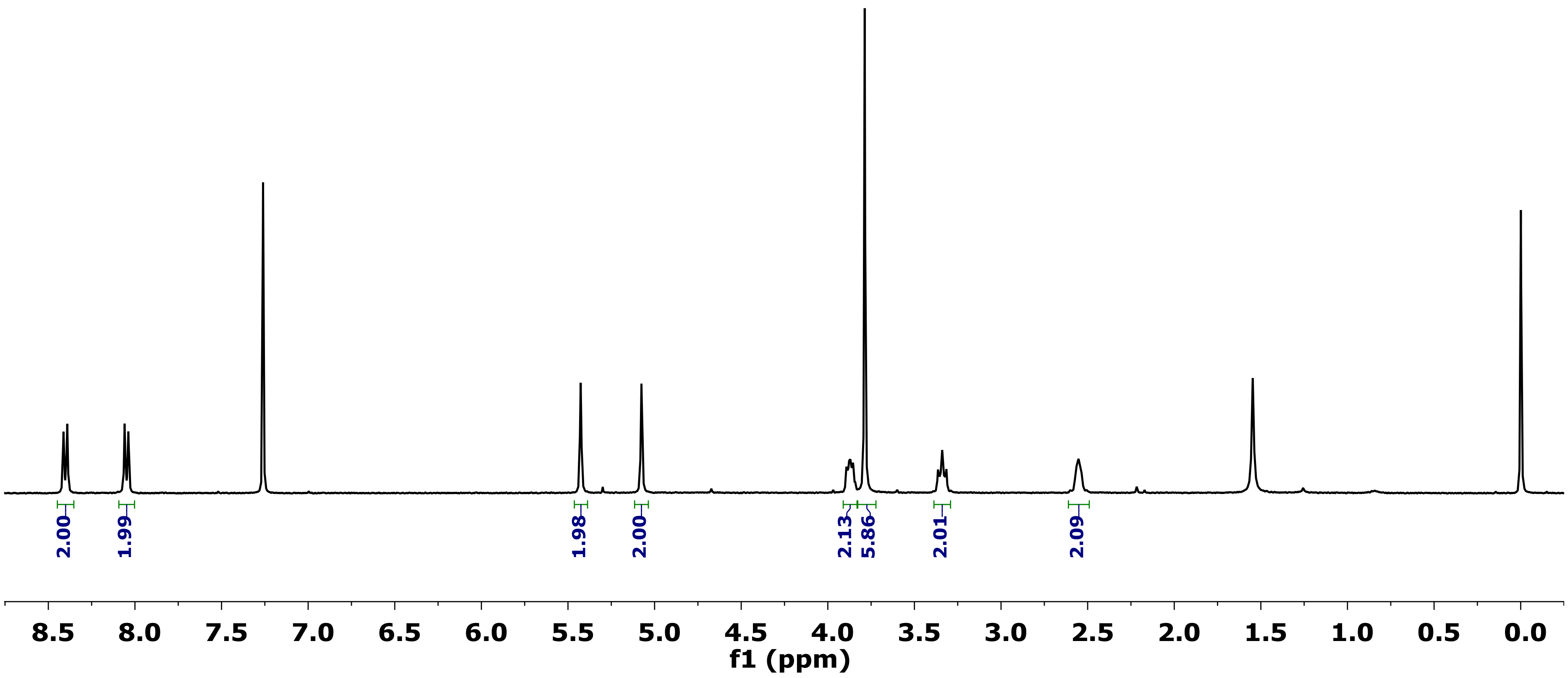

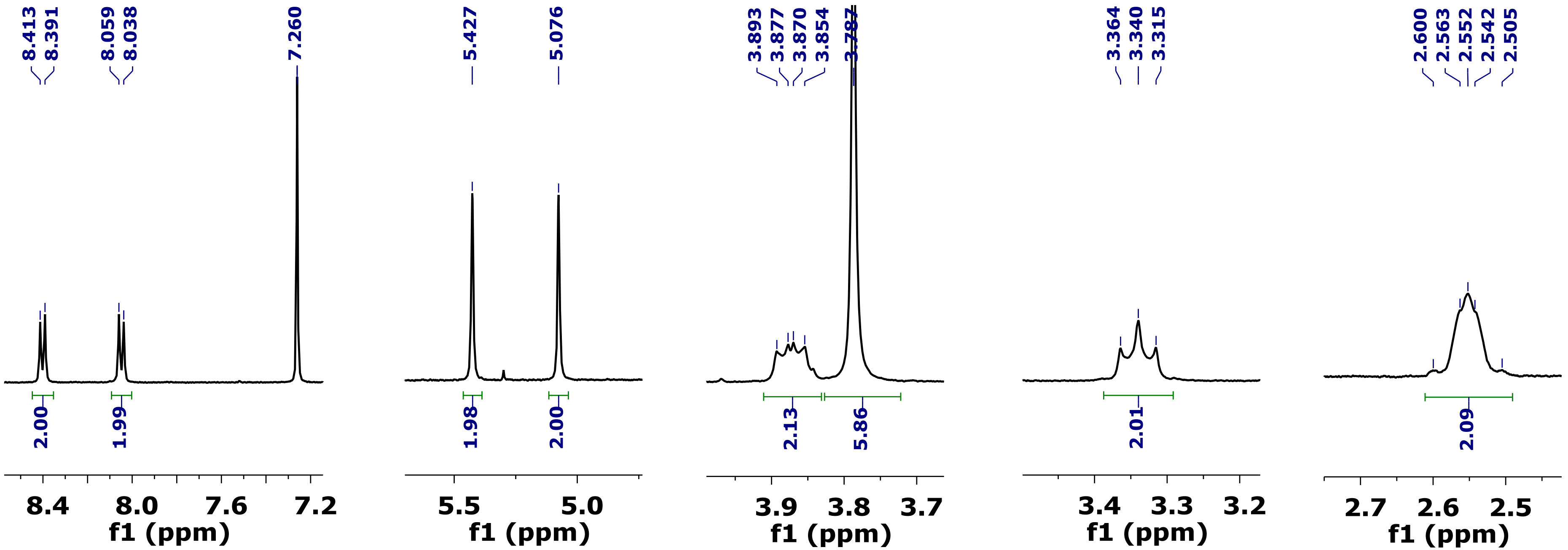
8.413
8.391
8.059
8.038
7.260
5.427
5.076
3.893
3.877
3.870
3.854
3.787
3.364
3.340
3.315
2.600
2.563
2.552
2.542
2.505
2.00
1.99
1.98
2.00
2.13
5.86
2.01
2.09
8.4
8.0
7.6
7.2
5.5
5.0
3.9
3.8
3.7
3.4
3.3
3.2
2.7
2.6
2.5
f1 (ppm)

^{13}C NMR (101 MHz, $CDCl_3$)

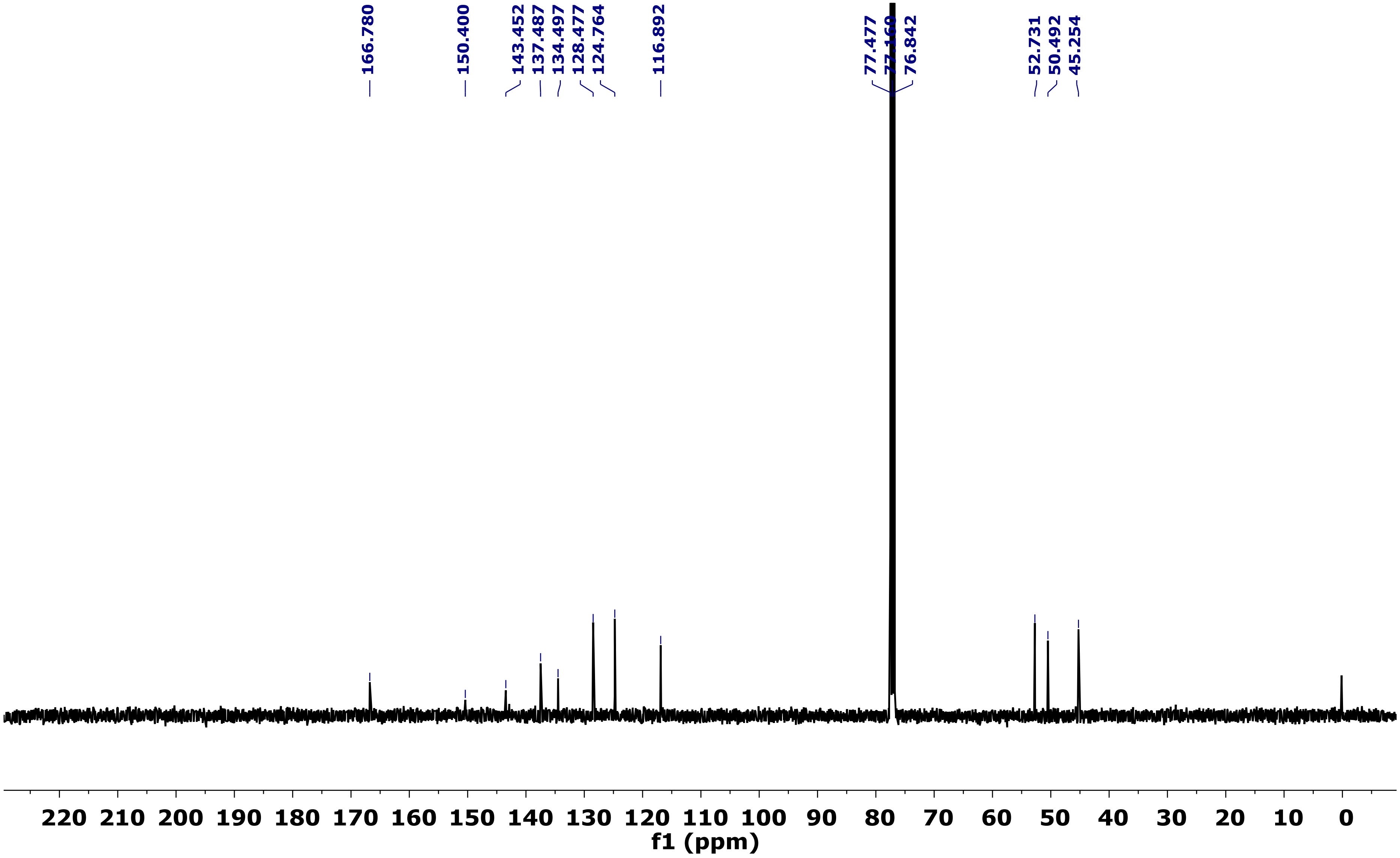

Product 3f

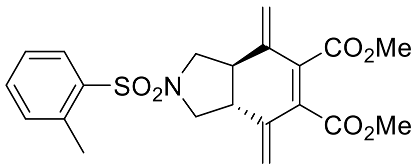

^1H NMR (400 MHz, $CDCl_3$)

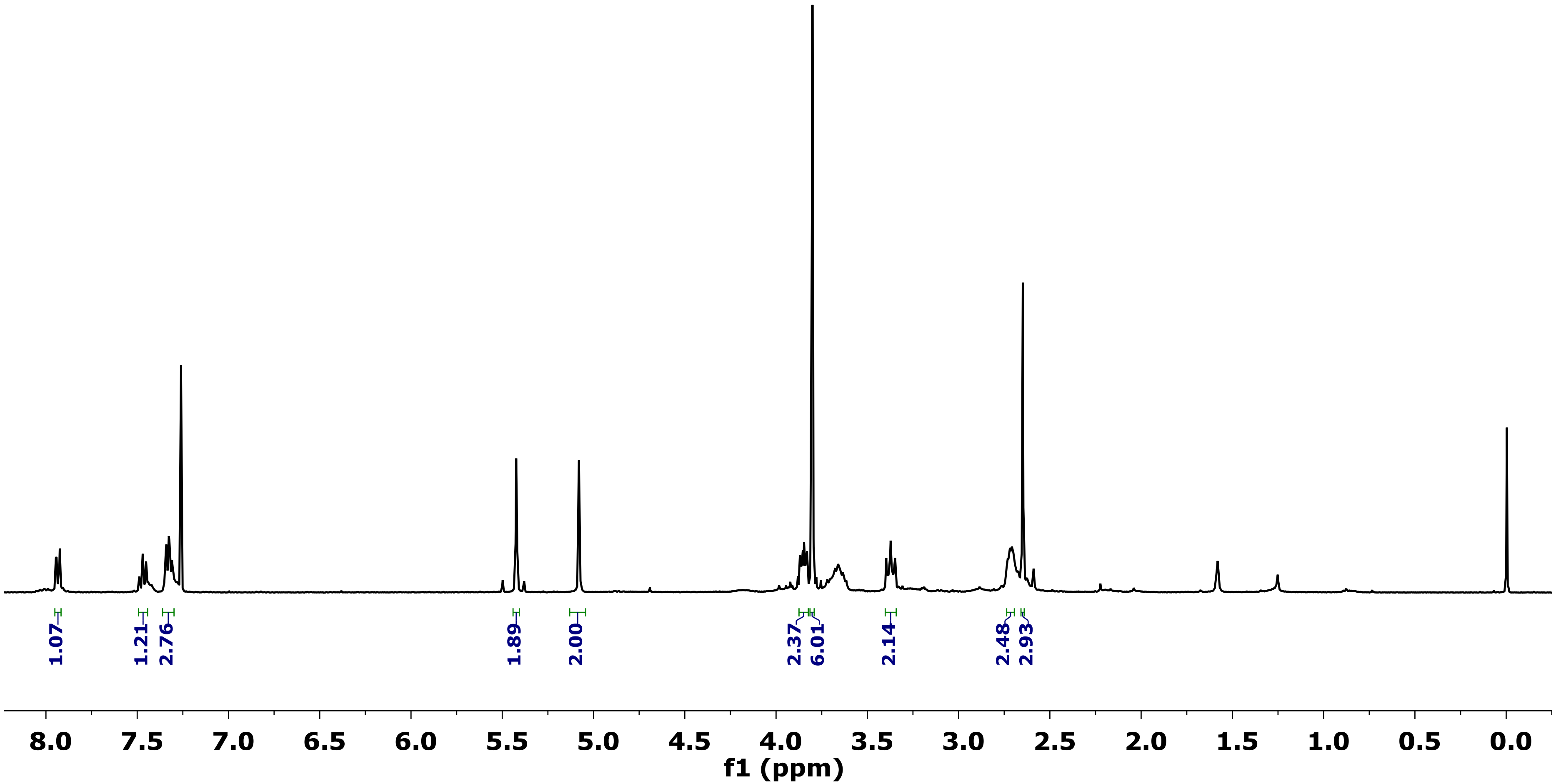

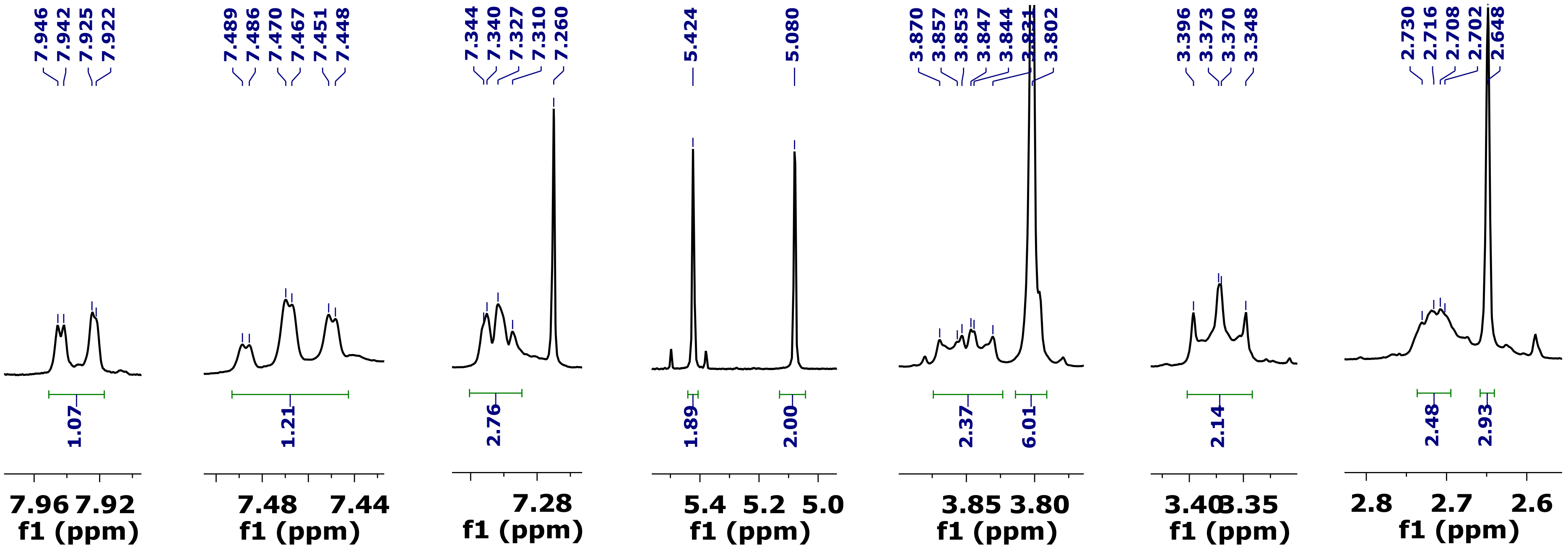
7.946
7.942
7.925
7.922
1.07
7.96 7.92
f1 (ppm)
7.489
7.486
7.470
7.467
7.451
7.448
1.21
7.48 7.44
f1 (ppm)
7.344
7.340
7.327
7.310
7.260
2.76
7.28
f1 (ppm)
5.424
5.080
1.89
2.00
5.4 5.2 5.0
f1 (ppm)
3.870
3.857
3.853
3.847
3.844
3.802
2.37
6.01
3.85 3.80
f1 (ppm)
3.396
3.373
3.370
3.348
2.14
3.40 3.35
f1 (ppm)
2.730
2.716
2.708
2.702
2.648
2.48
2.93
2.8 2.7 2.6
f1 (ppm)

^{13}C NMR (101 MHz, $CDCl_3$)

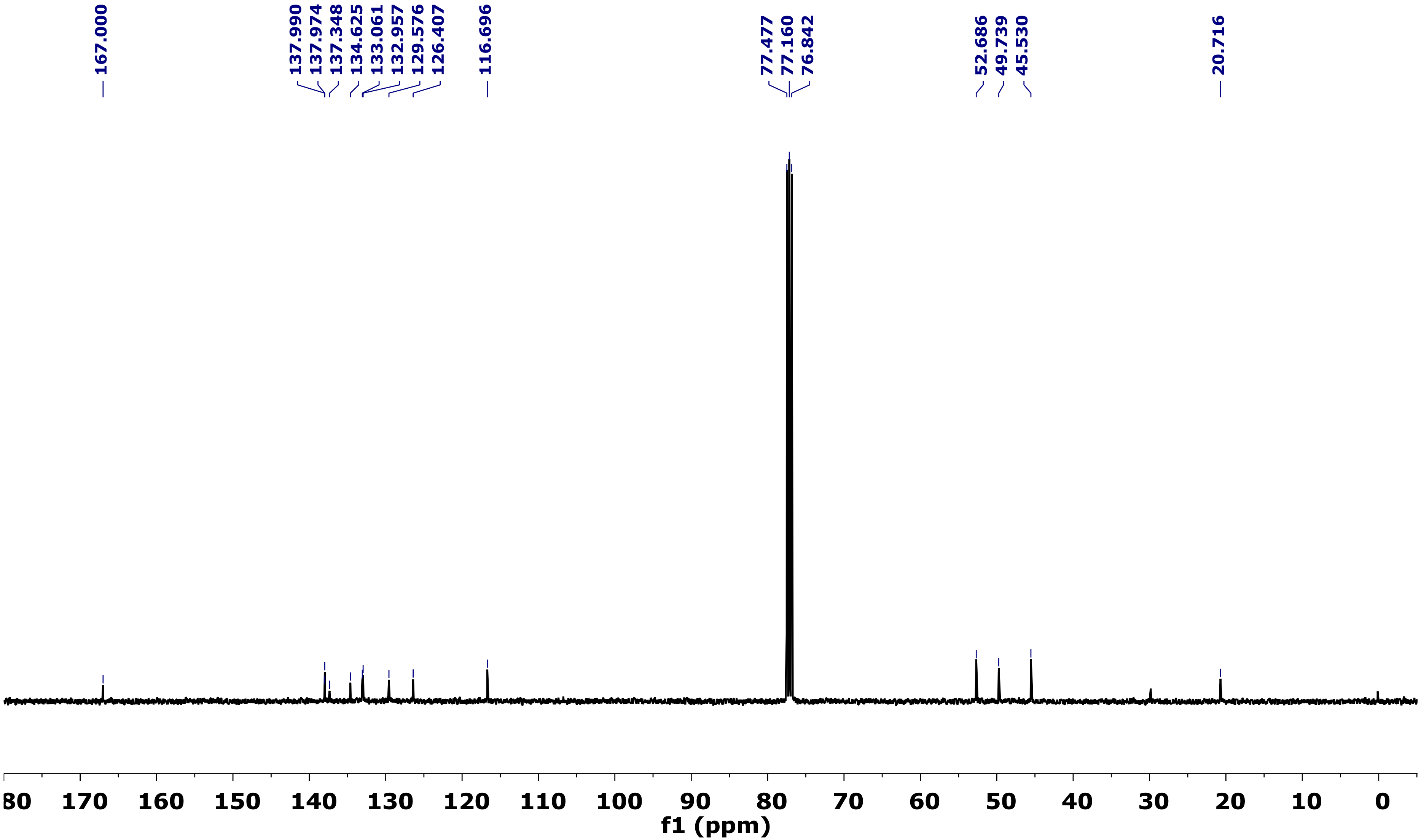

Product 3g

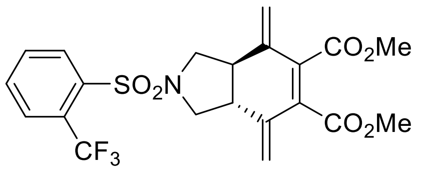

^{1}H NMR (400 MHz, $CDCl_3$)

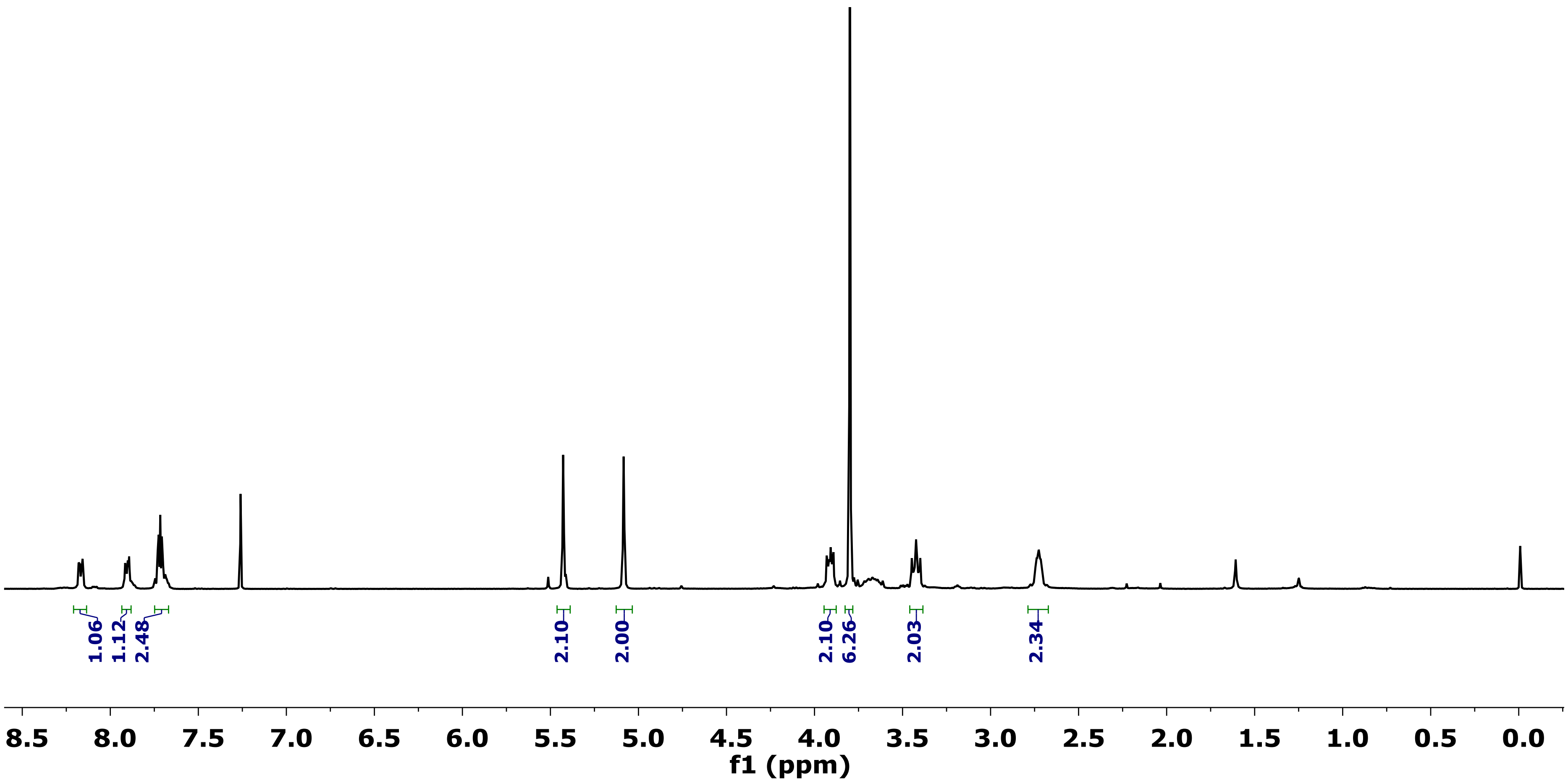

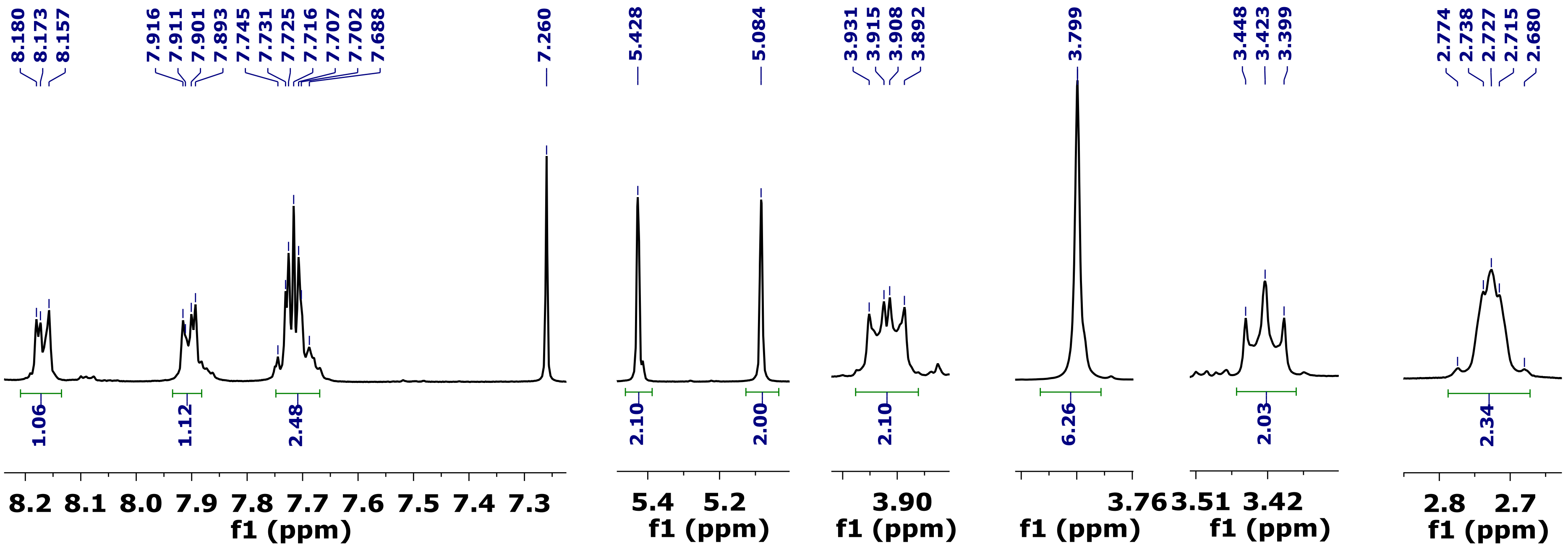
8.180
8.173
8.157
7.916
7.911
7.901
7.893
7.745
7.731
7.725
7.716
7.707
7.702
7.688
7.260
5.428
5.084
3.931
3.915
3.908
3.892
3.799
3.448
3.423
3.399
2.774
2.738
2.727
2.715
2.680
1.06
1.12
2.48
2.10
2.00
2.10
6.26
2.03
2.34
8.2 8.1 8.0 7.9 7.8 7.7 7.6 7.5 7.4 7.3
f1 (ppm)
5.4 5.2
f1 (ppm)
3.90
f1 (ppm)
3.76
f1 (ppm)
3.51 3.42
f1 (ppm)
2.8 2.7
f1 (ppm)

^{13}C NMR (101 MHz, $CDCl_3$)

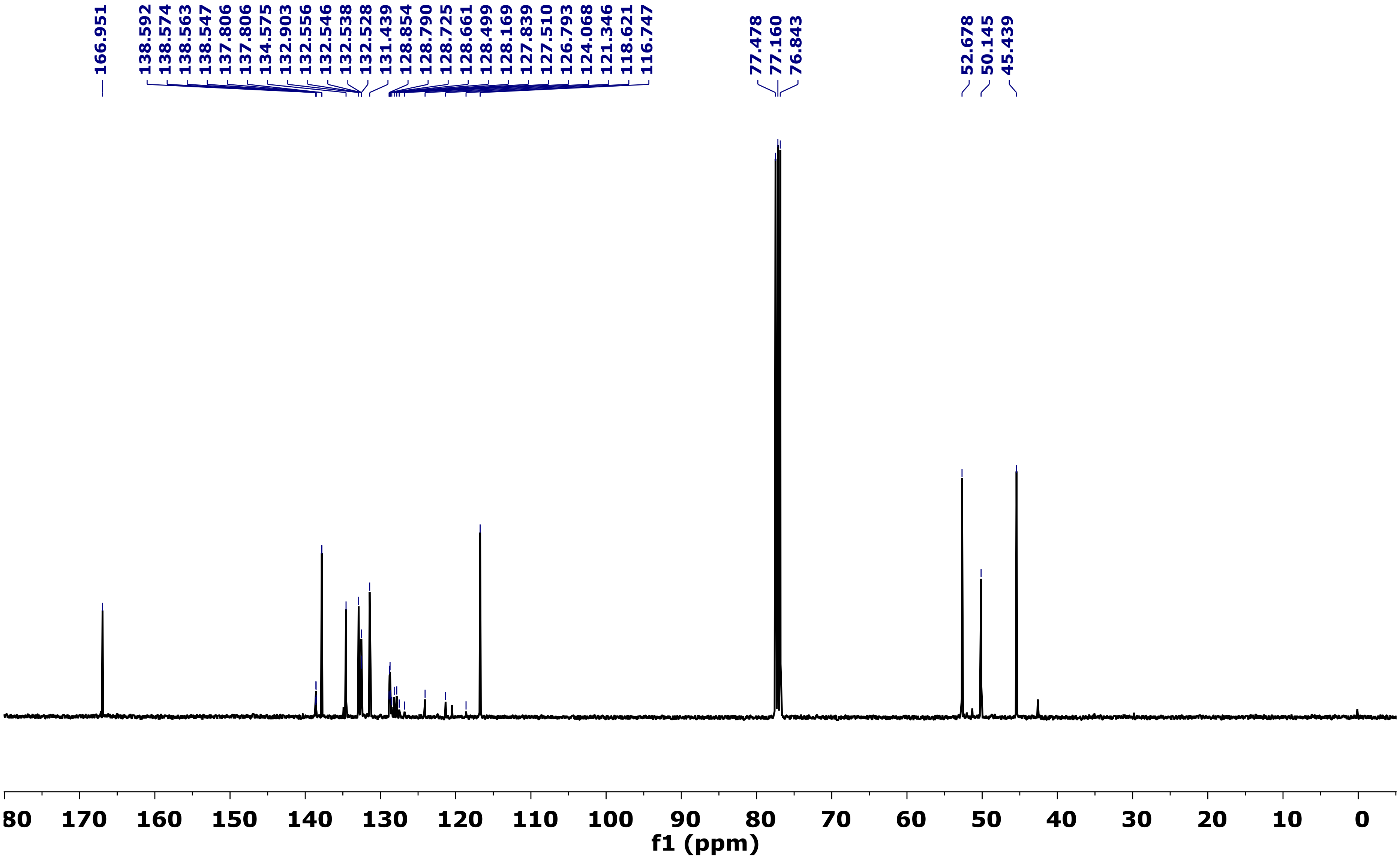

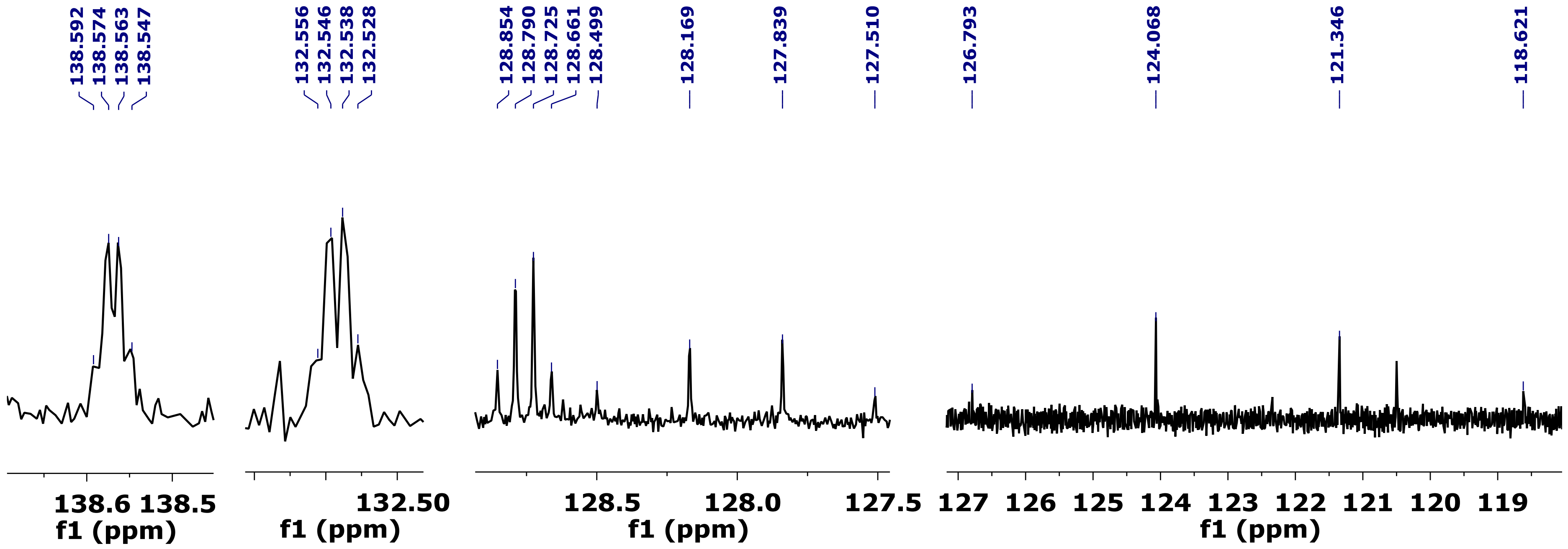
138.592
138.574
138.563
138.547
132.556
132.546
132.538
132.528
128.854
128.790
128.725
128.661
128.499
128.169
127.839
127.510
126.793
124.068
121.346
118.621
138.6 138.5
f1 (ppm)
132.50
f1 (ppm)
128.5
128.0
127.5
f1 (ppm)
127 126 125 124 123 122 121 120 119
f1 (ppm)

Product 3h

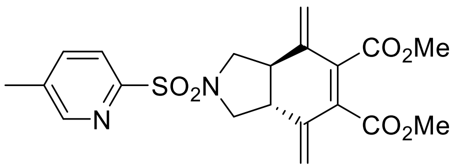

^{1}H NMR (400 MHz, $CDCl_3$)

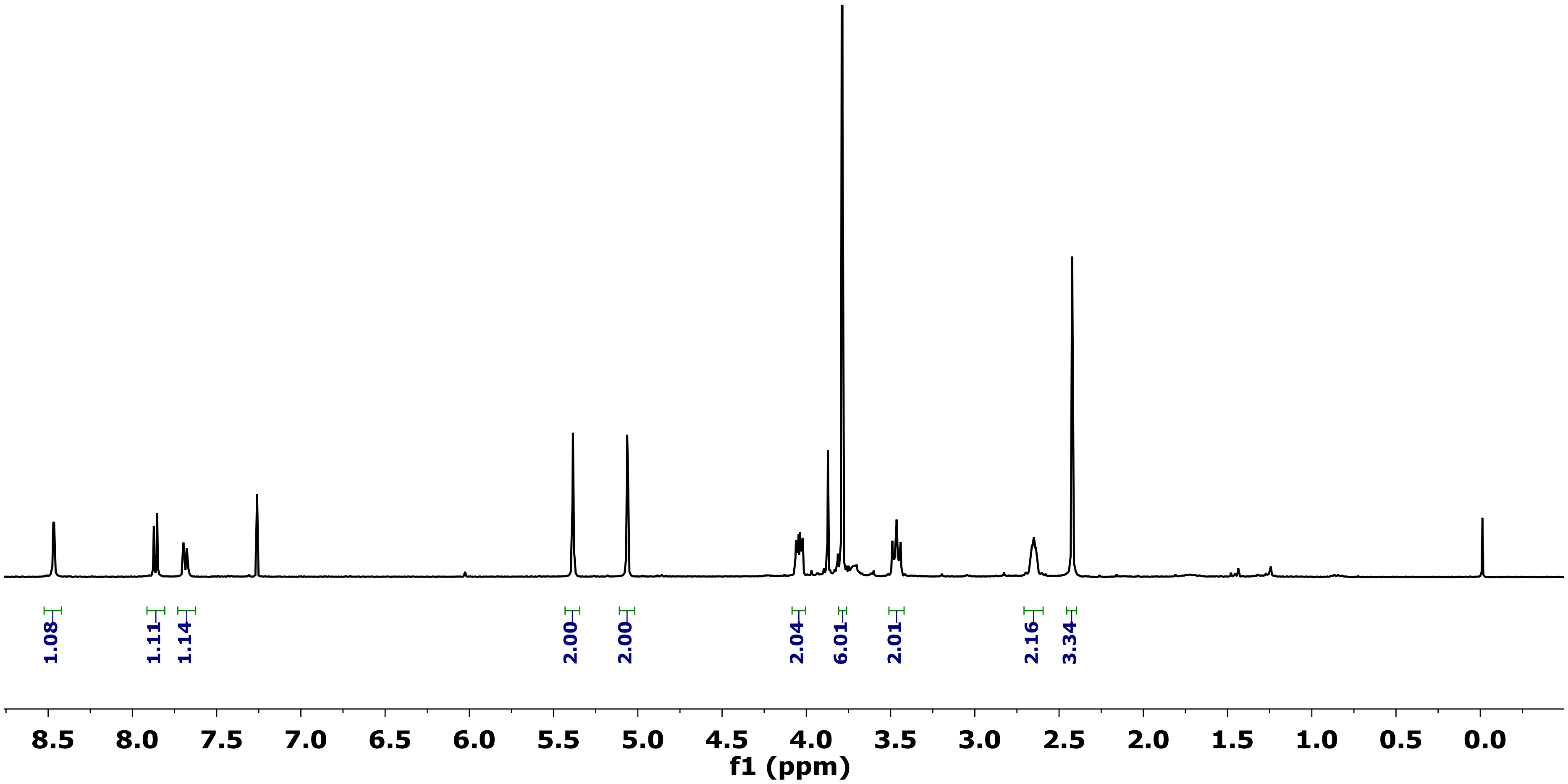

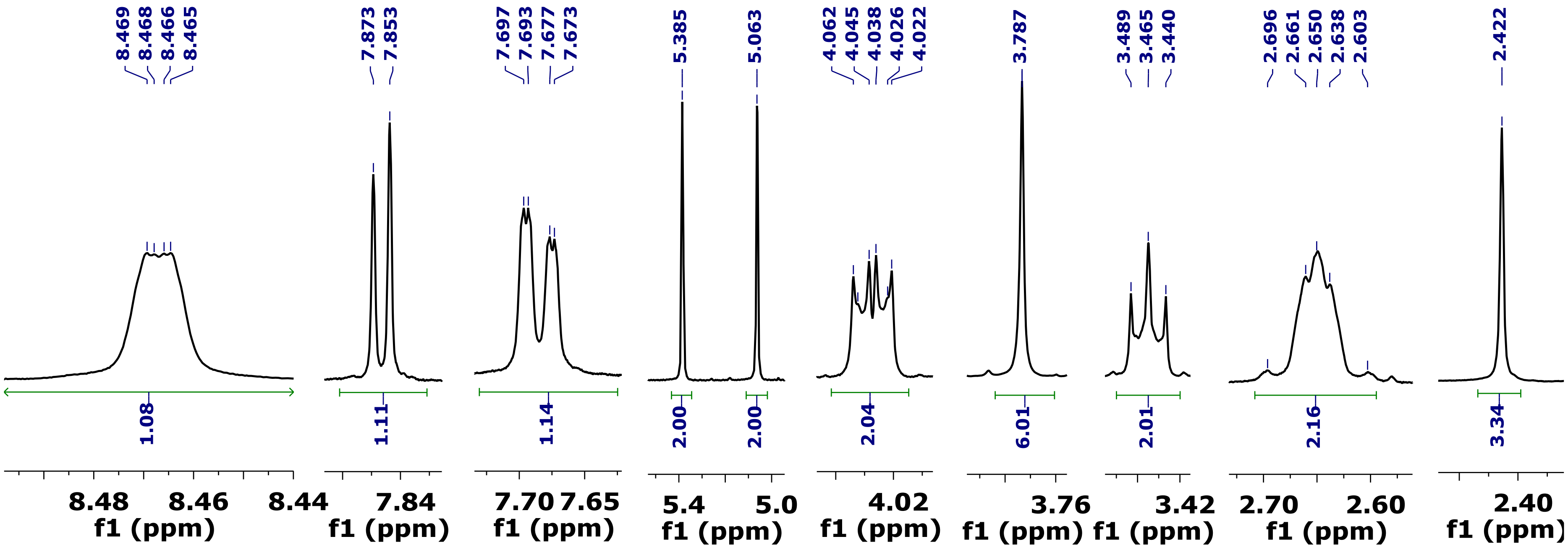
8.469
8.468
8.466
8.465
7.873
7.853
7.697
7.693
7.677
7.673
5.385
5.063
4.062
4.045
4.038
4.026
4.022
3.787
3.489
3.465
3.440
2.696
2.661
2.650
2.638
2.603
2.422
1.08
1.11
1.14
2.00
2.00
2.04
6.01
2.01
2.16
3.34
8.48 8.46 8.44
7.84
7.70 7.65
5.4 5.0
4.02
3.76
3.42
2.70 2.60
2.40
f1 (ppm)

^{13}C NMR (101 MHz, $CDCl_3$)

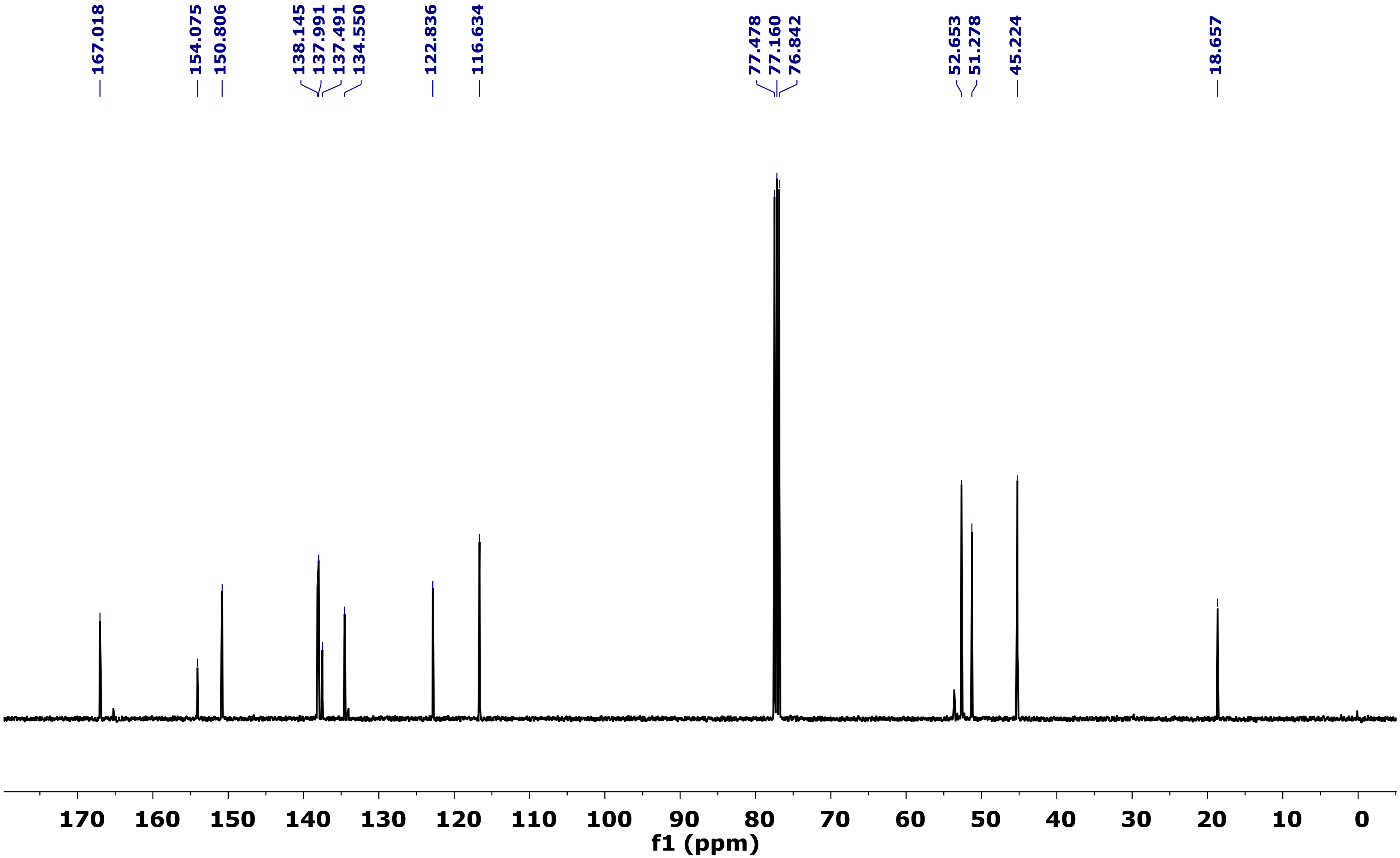

Product 3i

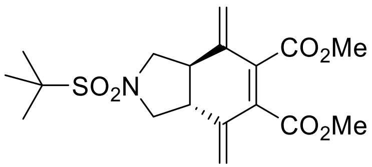

^1H NMR (400 MHz, $CDCl_3$)

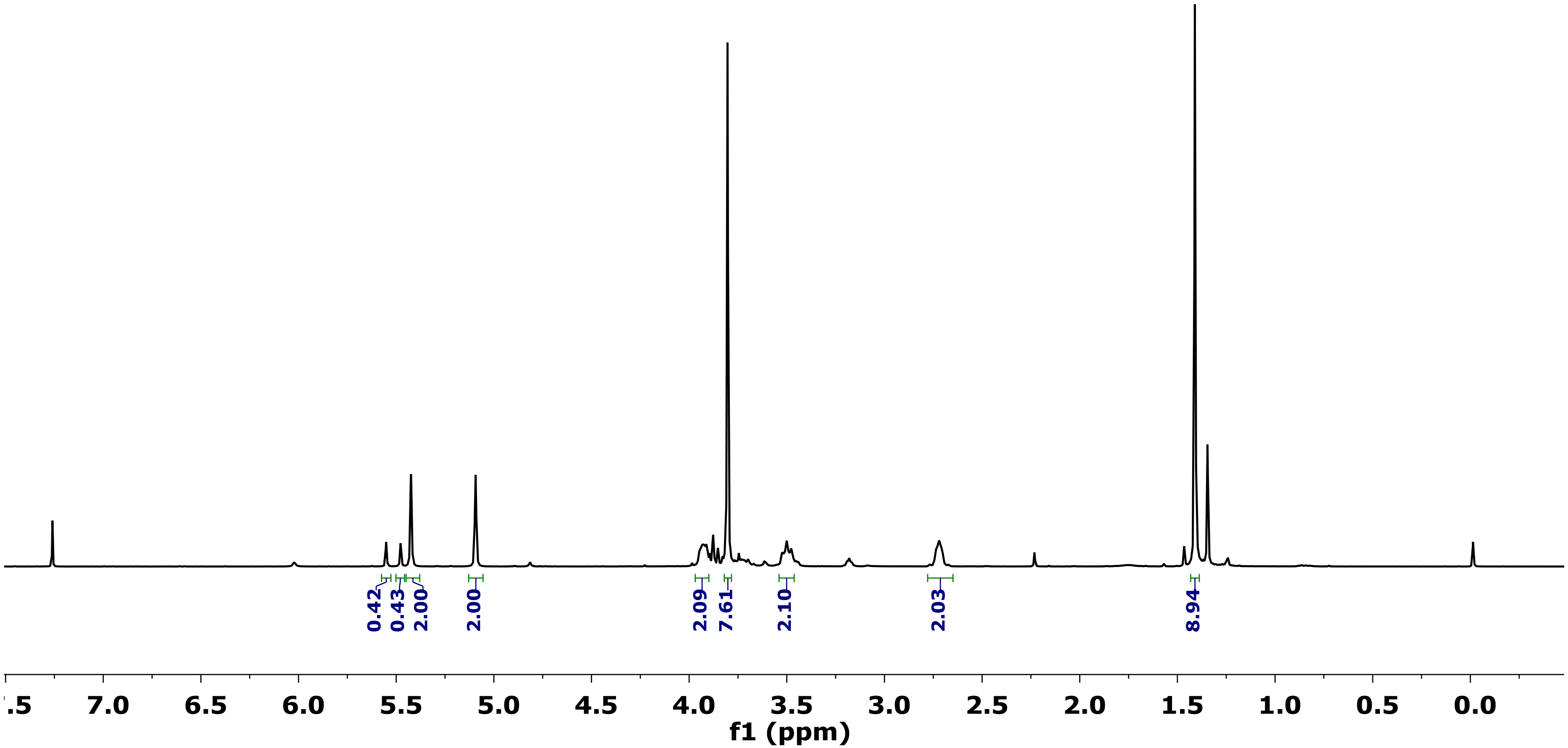

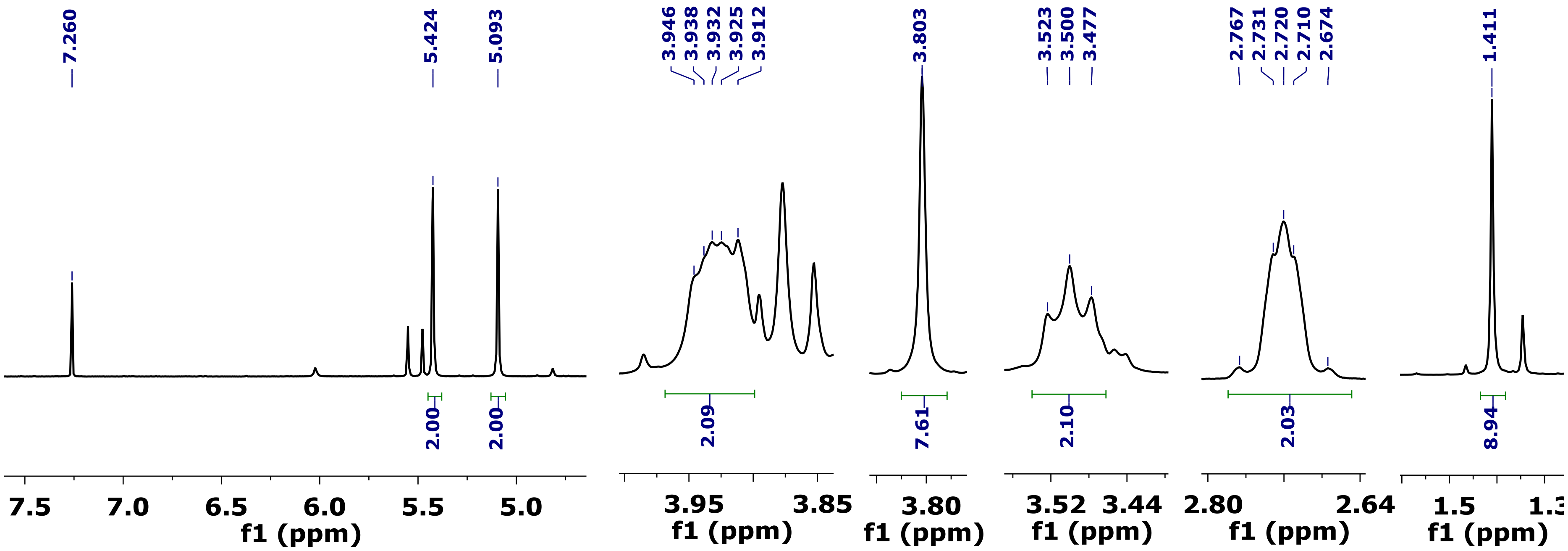
7.260
5.424
5.093
3.946
3.938
3.932
3.925
3.912
3.803
3.523
3.500
3.477
2.767
2.731
2.720
2.710
2.674
1.411
2.00
2.00
2.09
7.61
2.10
2.03
8.94
7.5 7.0 6.5 6.0 5.5 5.0
f1 (ppm)
3.95 3.85
f1 (ppm)
3.80
f1 (ppm)
3.52 3.44
f1 (ppm)
2.80 2.64
f1 (ppm)
1.5
f1 (ppm)

^{13}C NMR (101 MHz, $CDCl_3$)

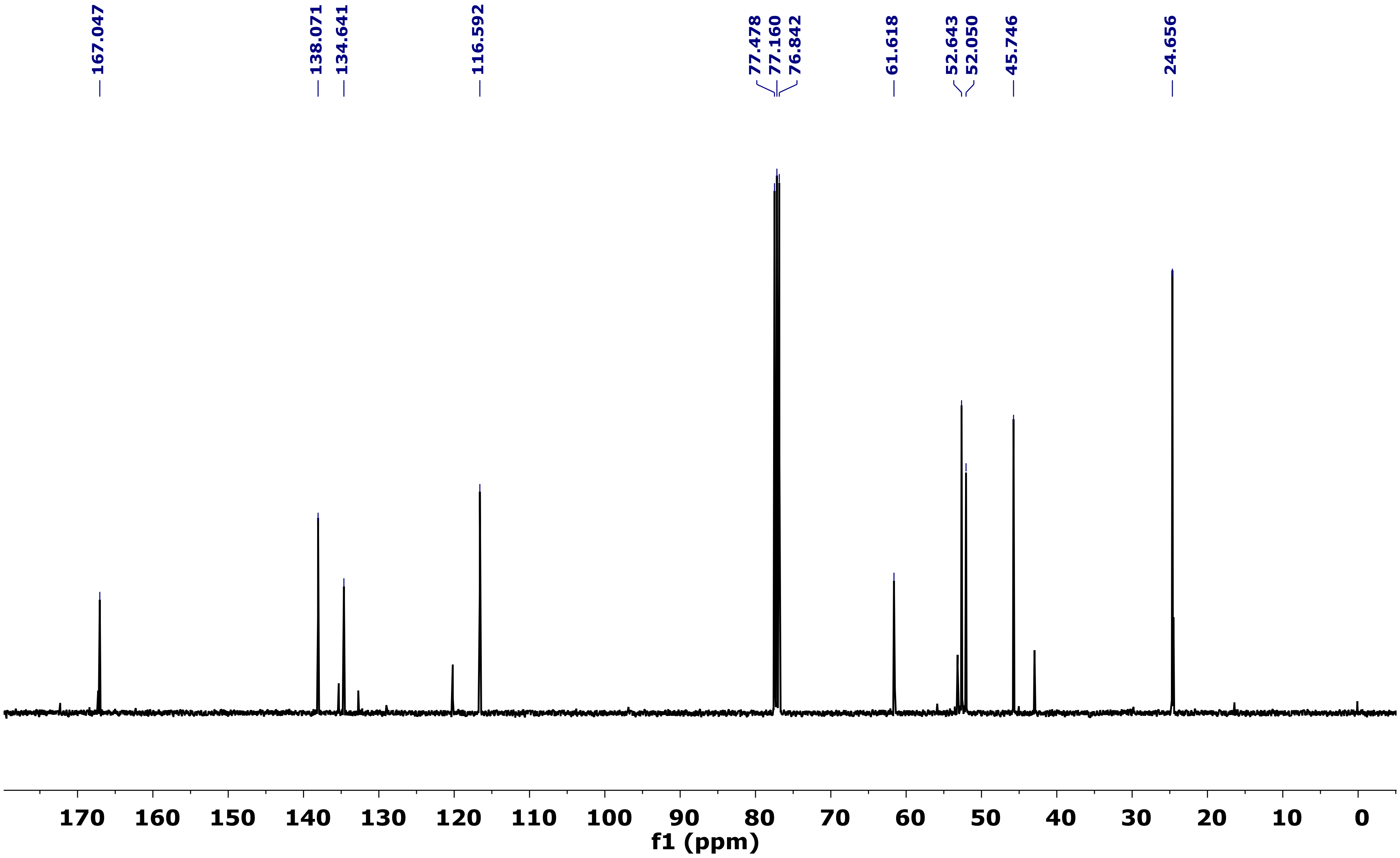

Product 3j

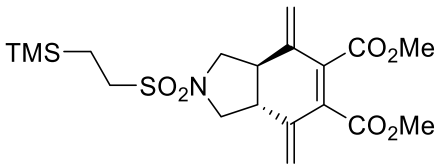

^{1}H NMR (400 MHz, $CDCl_3$)

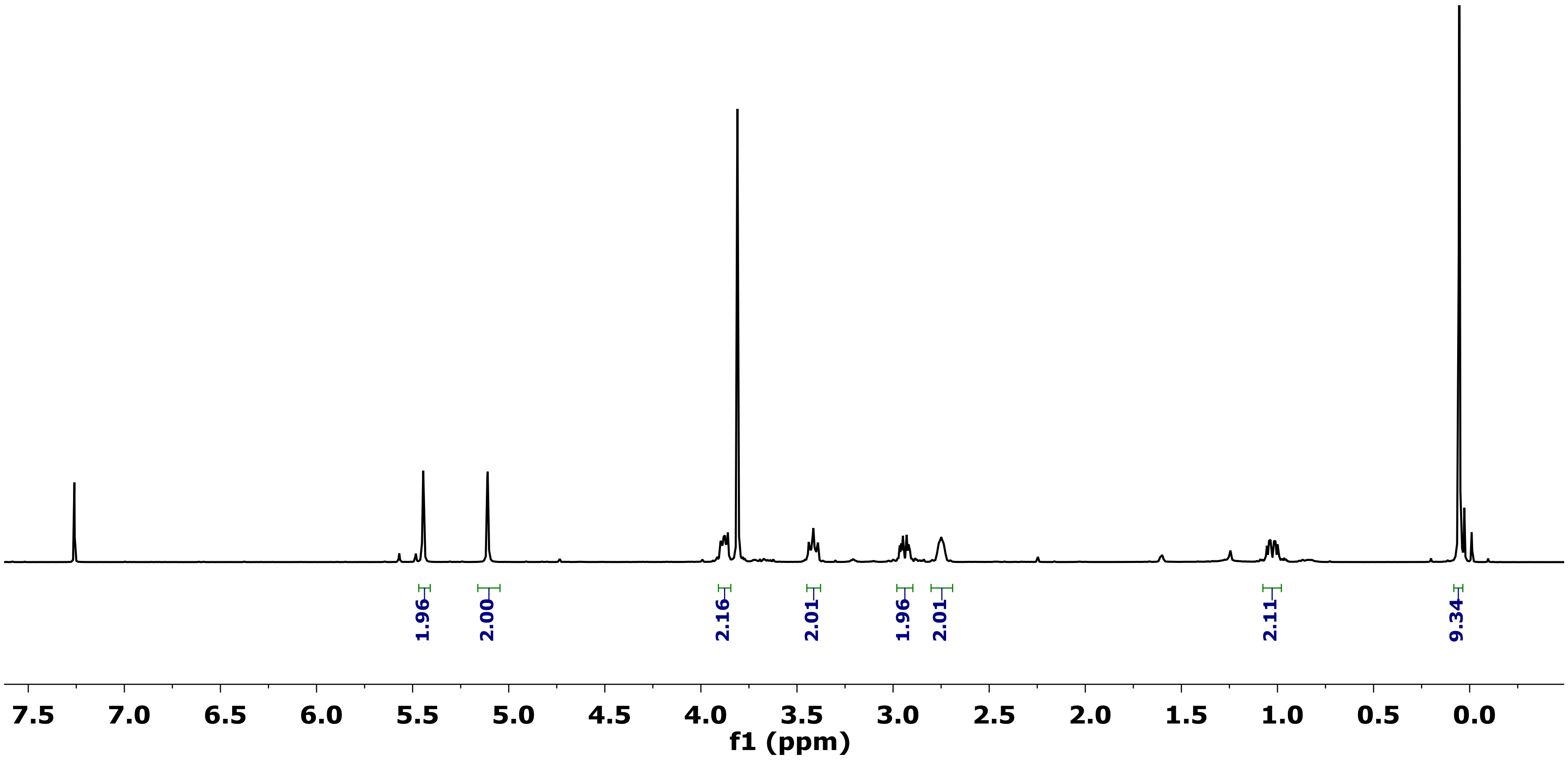

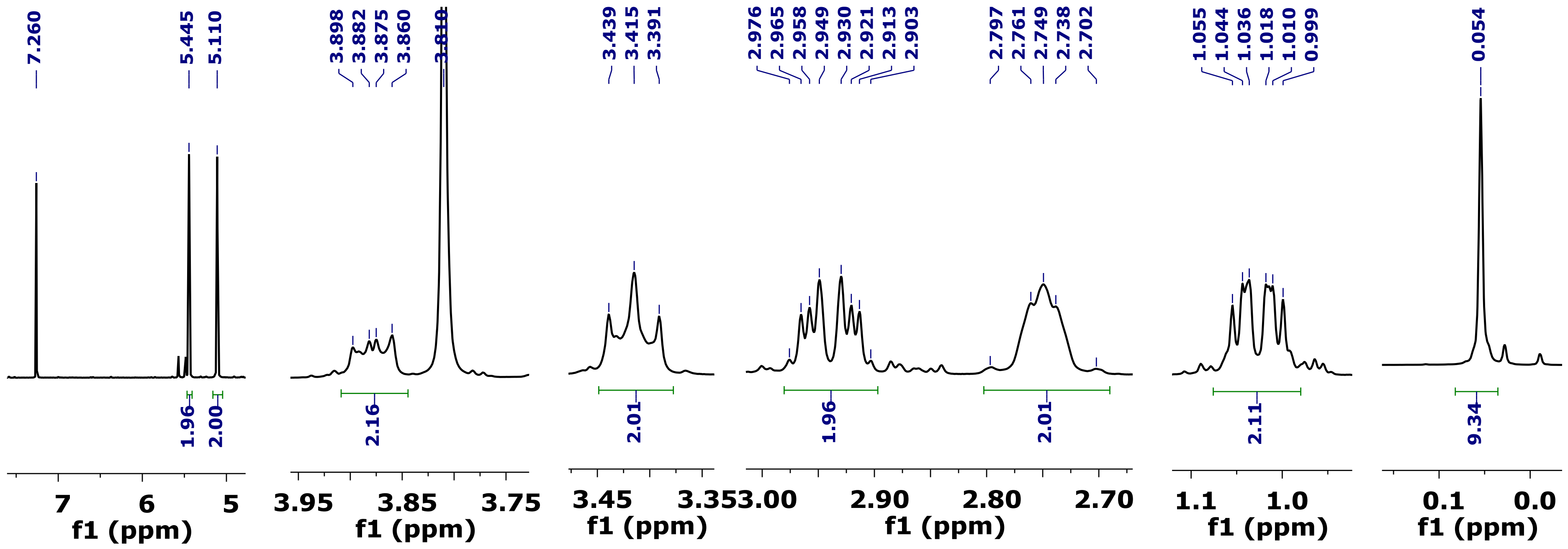
7.260
5.445
5.110
1.96
2.00
7
6
5
f1 (ppm)
3.898
3.882
3.875
3.860
3.810
2.16
3.95
3.85
3.75
f1 (ppm)
3.439
3.415
3.391
2.01
3.45
3.35
f1 (ppm)
2.976
2.965
2.958
2.949
2.930
2.921
2.913
2.903
2.797
2.761
2.749
2.738
2.702
1.96
2.01
3.00
2.90
2.80
2.70
f1 (ppm)
1.055
1.044
1.036
1.018
1.010
0.999
2.11
1.1
1.0
f1 (ppm)
0.054
9.34
0.1
0.0
f1 (ppm)

^{13}C NMR (101 MHz, $CDCl_3$)

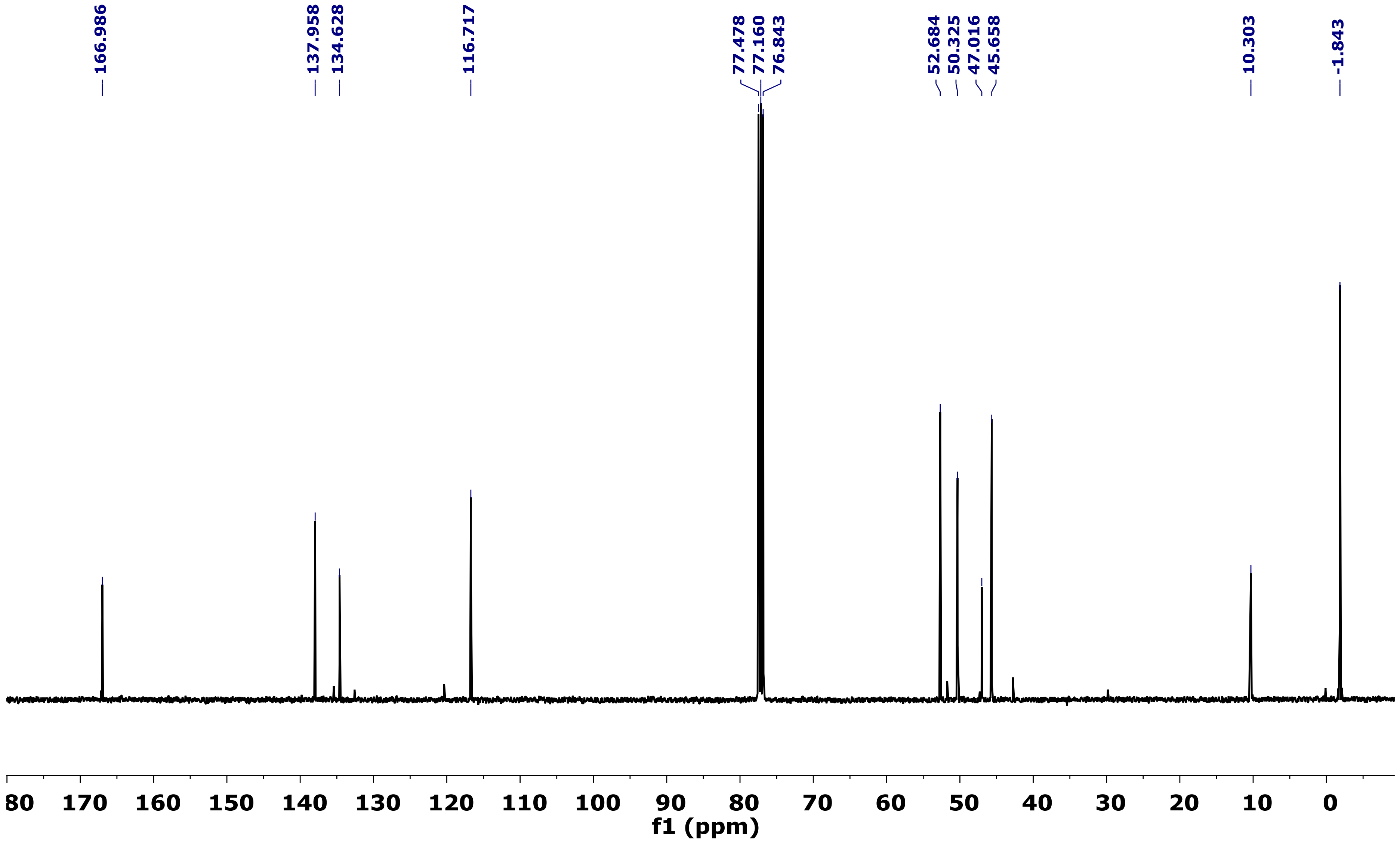

Product 3k

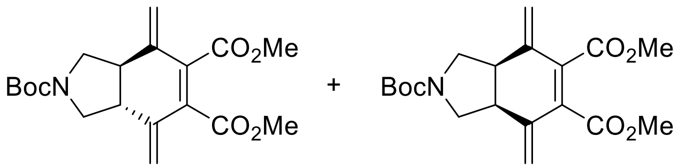

^{1}H NMR (400 MHz, $CDCl_3$)

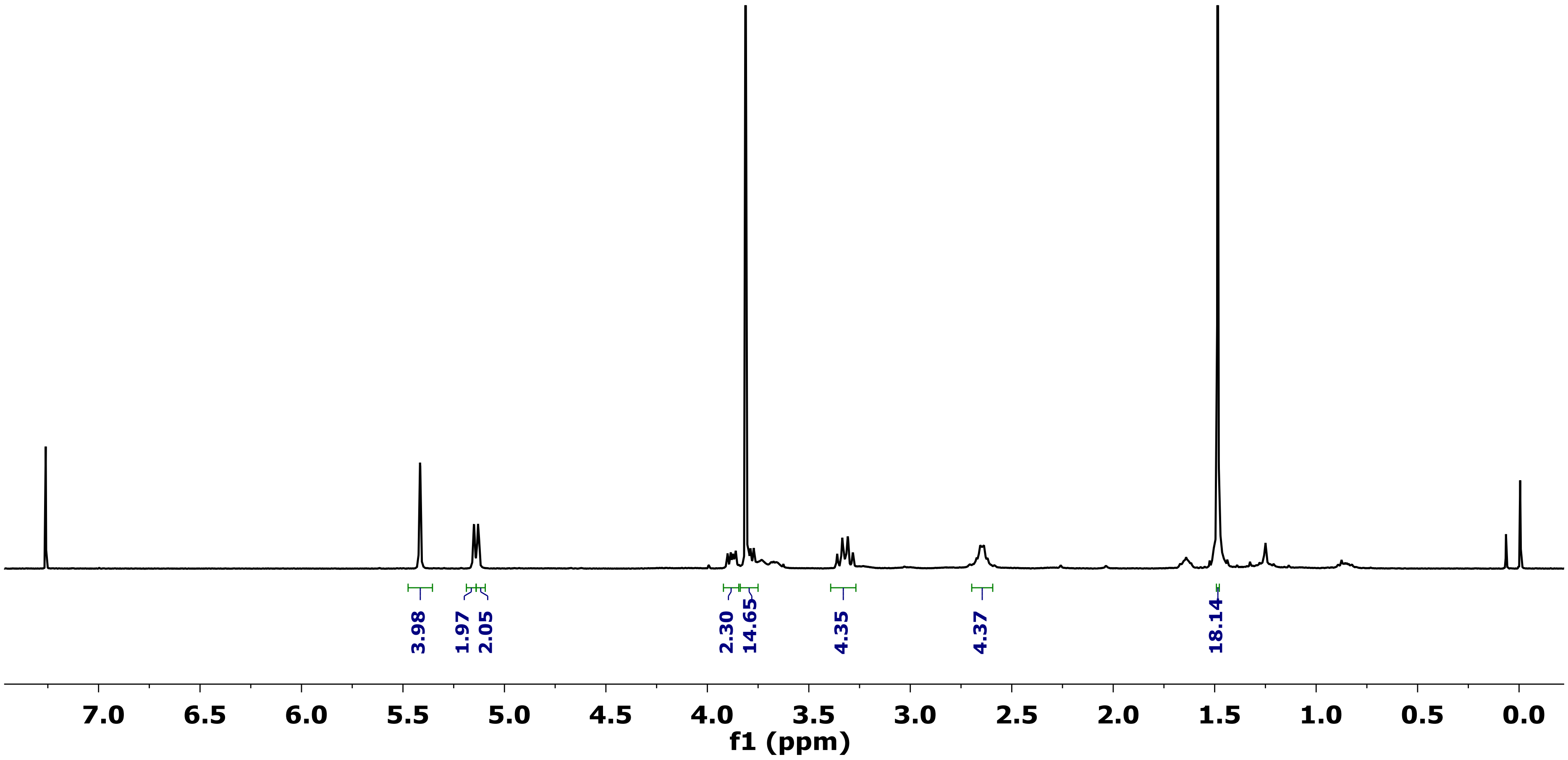

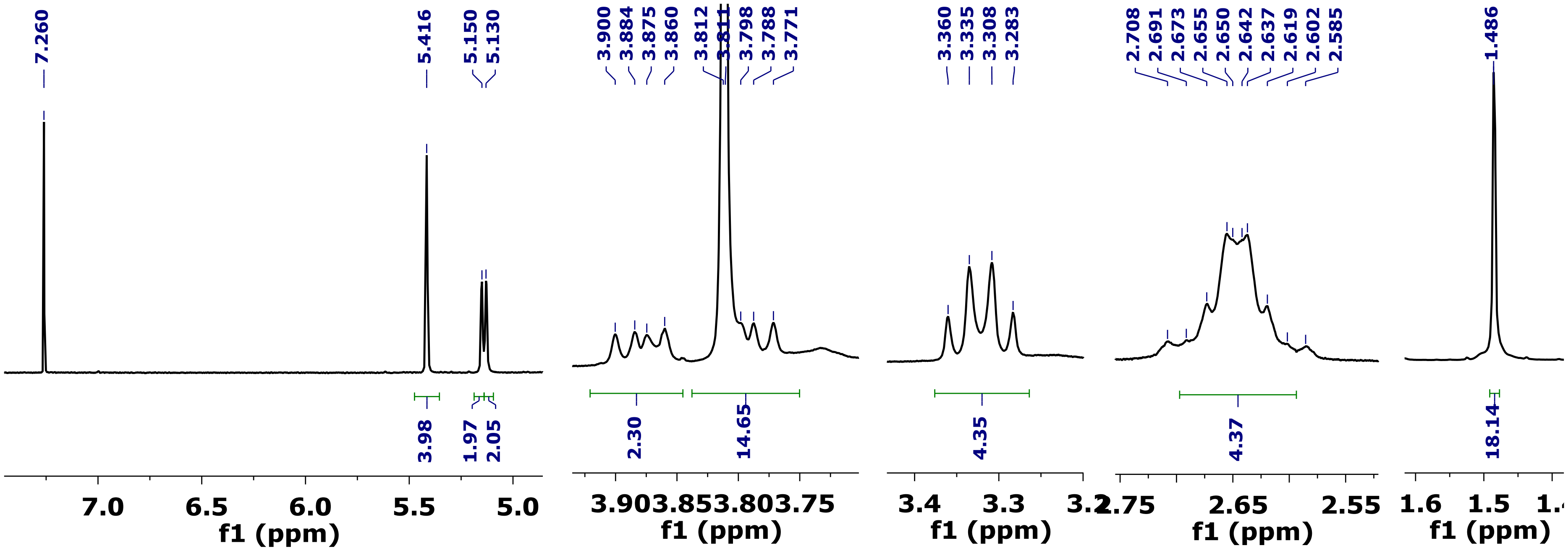
7.260
5.416
5.150
5.130
3.98
1.97
2.05
7.0
6.5
6.0
5.5
5.0
f1 (ppm)
3.900
3.884
3.875
3.860
3.812
3.811
3.798
3.788
3.771
2.30
14.65
3.90
3.85
3.80
3.75
f1 (ppm)
3.360
3.335
3.308
3.283
4.35
3.4
3.3
f1 (ppm)
2.708
2.691
2.673
2.655
2.650
2.642
2.637
2.619
2.602
2.585
4.37
2.75
2.65
2.55
f1 (ppm)
1.486
18.14
1.6
1.5
f1 (ppm)

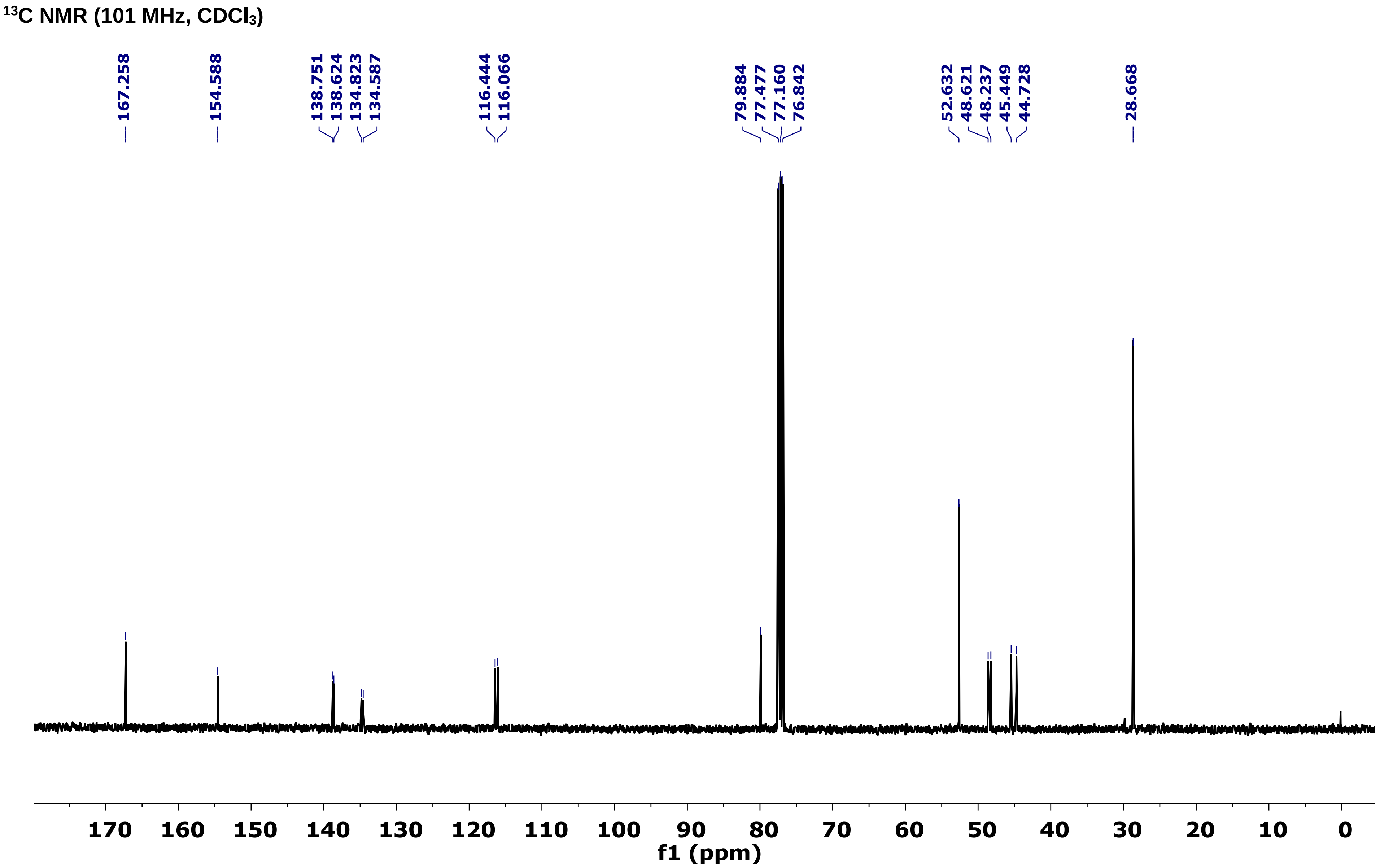

13C NMR (101 MHz, CDCl3)
167.258
154.588
138.751
138.624
134.823
134.587
116.444
116.066
79.884
77.477
77.160
76.842
52.632
48.621
48.237
45.449
44.728
28.668
170 160 150 140 130 120 110 100 90 80 70 60 50 40 30 20 10 0
f1 (ppm)

2D NMR HSQC

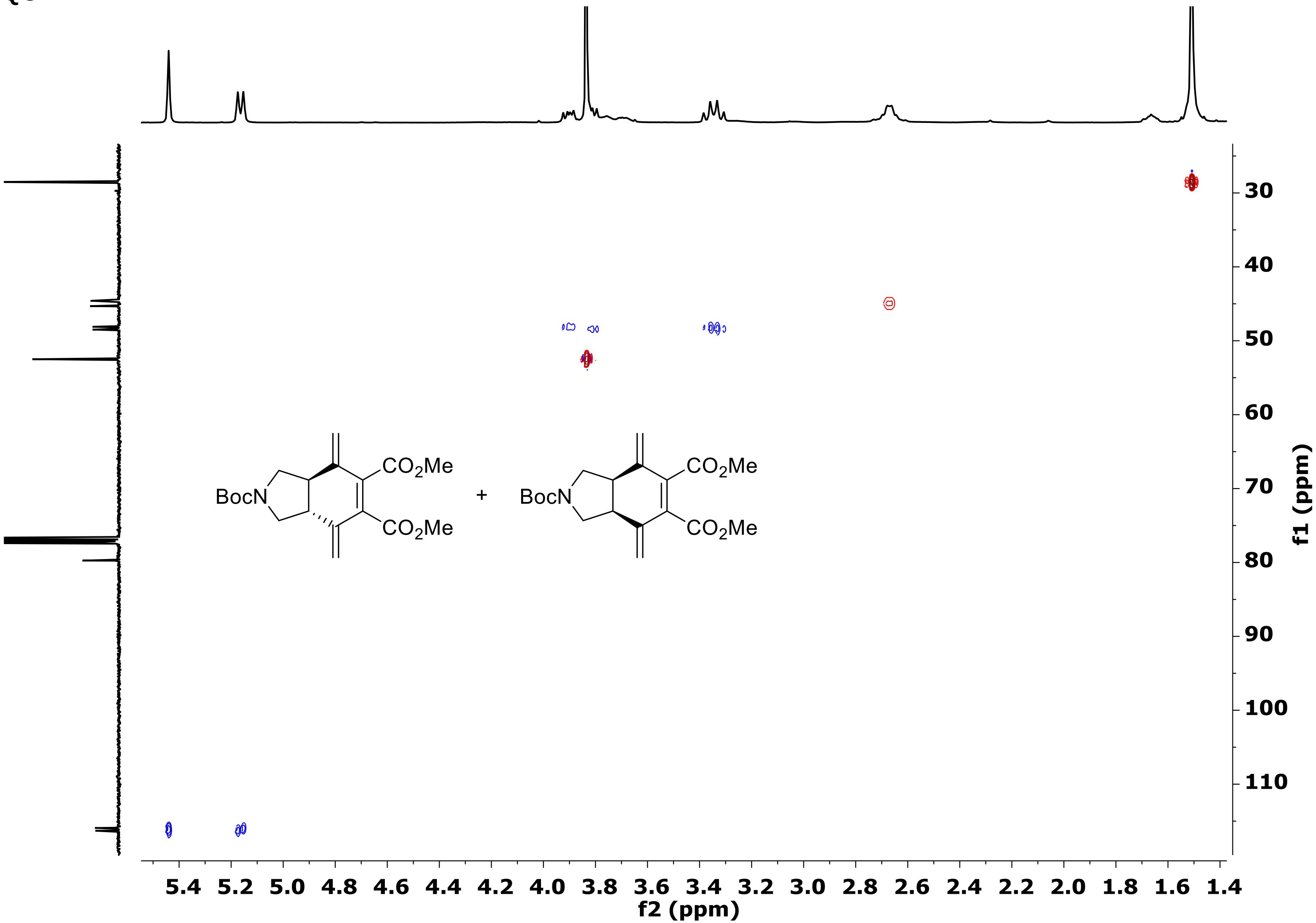

BocN
CO2Me
CO2Me
+
BocN
CO2Me
CO2Me
5.4 5.2 5.0 4.8 4.6 4.4 4.2 4.0 3.8 3.6 3.4 3.2 3.0 2.8 2.6 2.4 2.2 2.0 1.8 1.6 1.4
f2 (ppm)
30
40
50
60
70
80
90
100
110
f1 (ppm)

2D NMR COSY

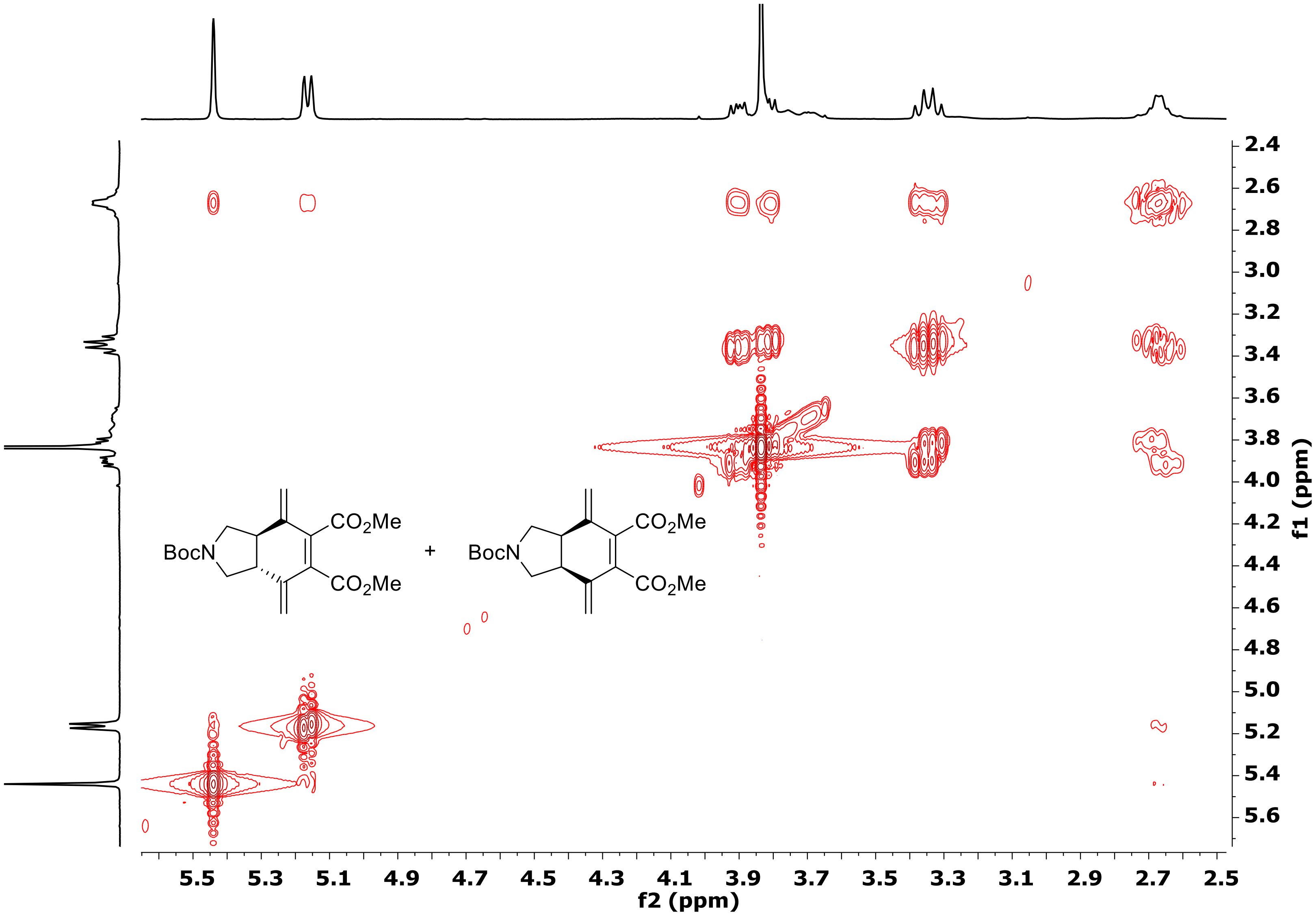

BocN
CO2Me
CO2Me
+
BocN
CO2Me
CO2Me
2.4
2.6
2.8
3.0
3.2
3.4
3.6
3.8
4.0
4.2
4.4
4.6
4.8
5.0
5.2
5.4
5.6
f1 (ppm)
5.5
5.3
5.1
4.9
4.7
4.5
4.3
4.1
3.9
3.7
3.5
3.3
3.1
2.9
2.7
2.5
f2 (ppm)

Product 3l

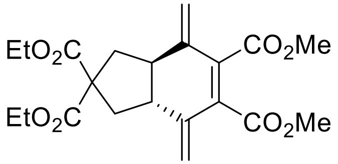

^{1}H NMR (400 MHz, $CDCl_3$)

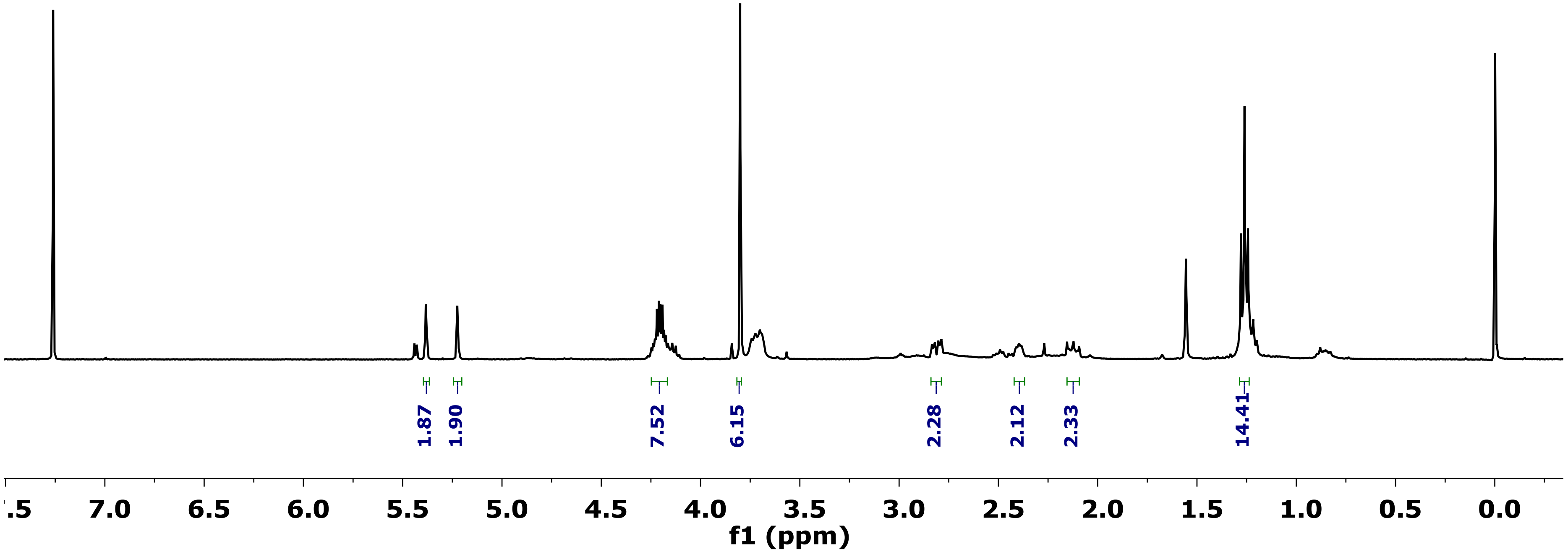

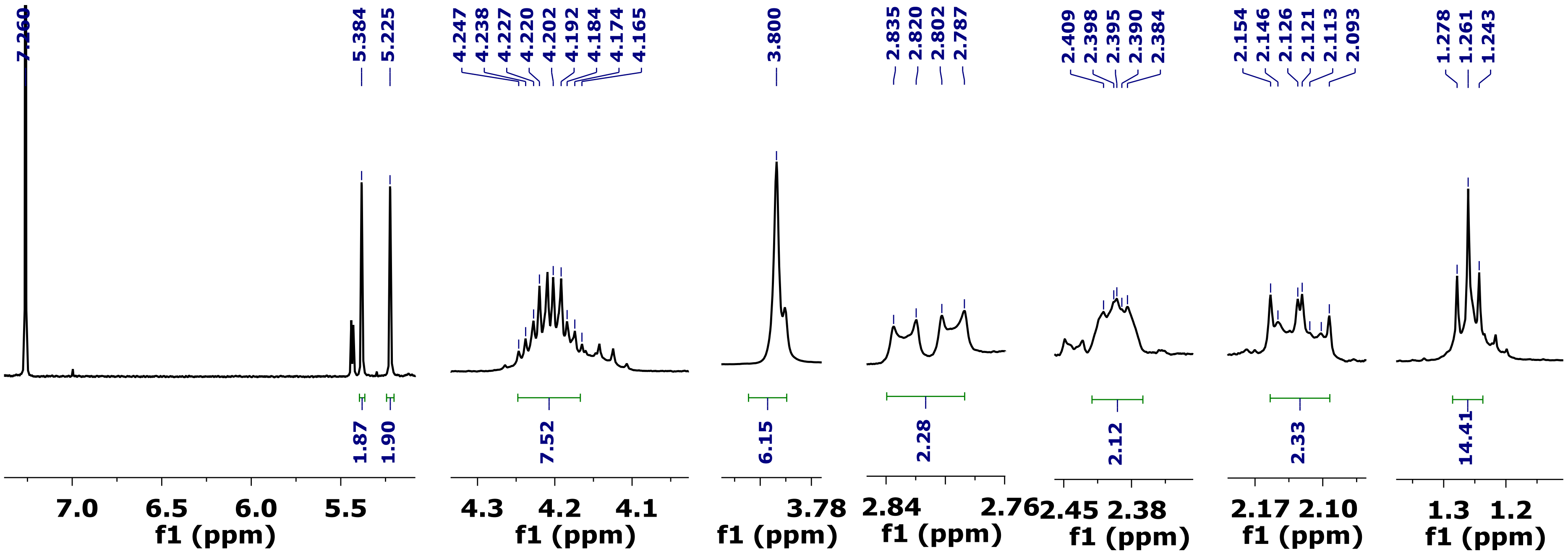
7.260
5.384
5.225
4.247
4.238
4.227
4.220
4.202
4.192
4.184
4.174
4.165
3.800
2.835
2.820
2.802
2.787
2.409
2.398
2.395
2.390
2.384
2.154
2.146
2.126
2.121
2.113
2.093
1.278
1.261
1.243
1.87
1.90
7.52
6.15
2.28
2.12
2.33
14.41
7.0 6.5 6.0 5.5
4.3 4.2 4.1
3.78
2.84 2.76
2.45 2.38
2.17 2.10
1.3 1.2
f1 (ppm)

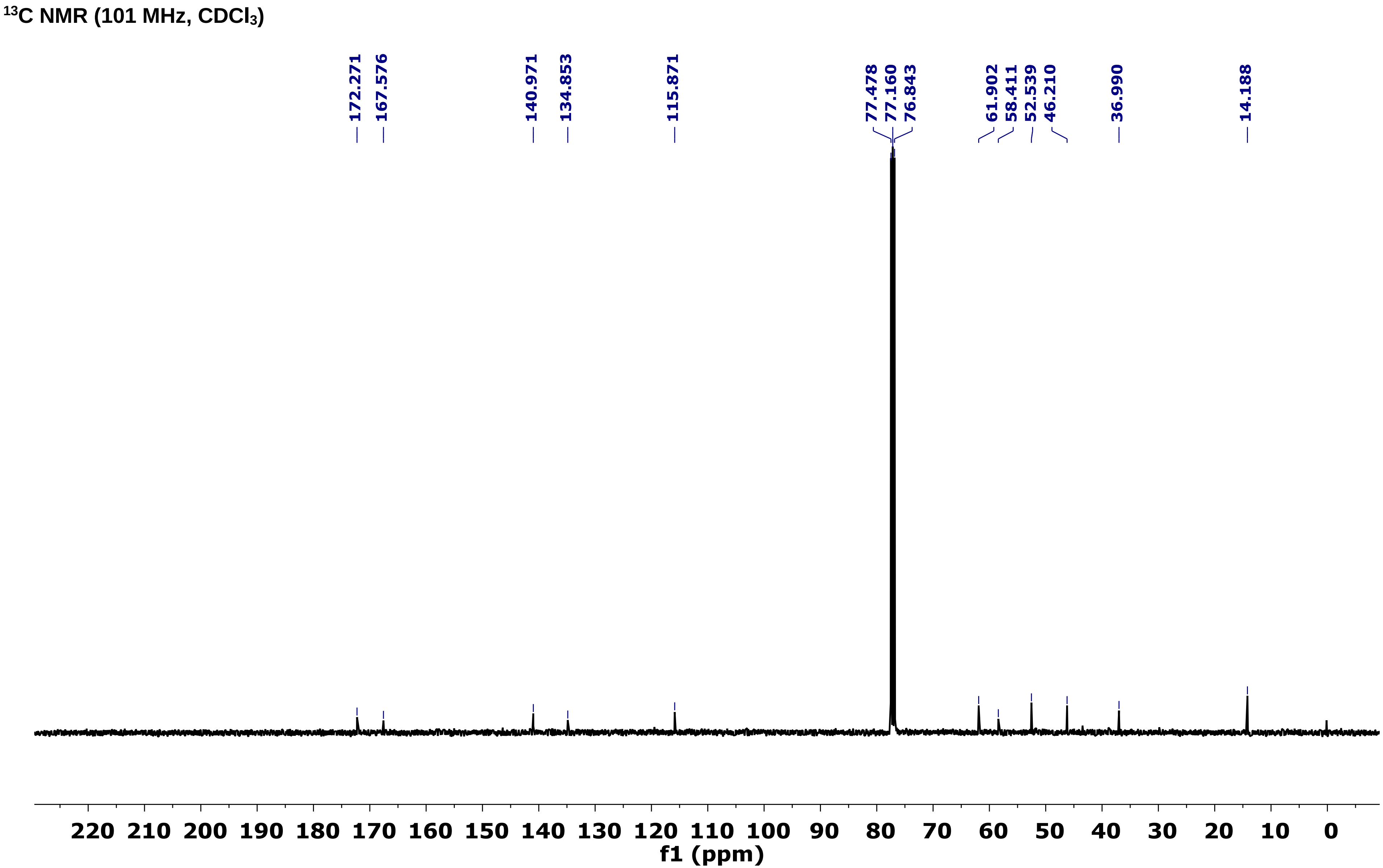
13C NMR (101 MHz, CDCl3)
172.271
167.576
140.971
134.853
115.871
77.478
77.160
76.843
61.902
58.411
52.539
46.210
36.990
14.188
220 210 200 190 180 170 160 150 140 130 120 110 100 90 80 70 60 50 40 30 20 10 0
f1 (ppm)

2D NMR HSQC

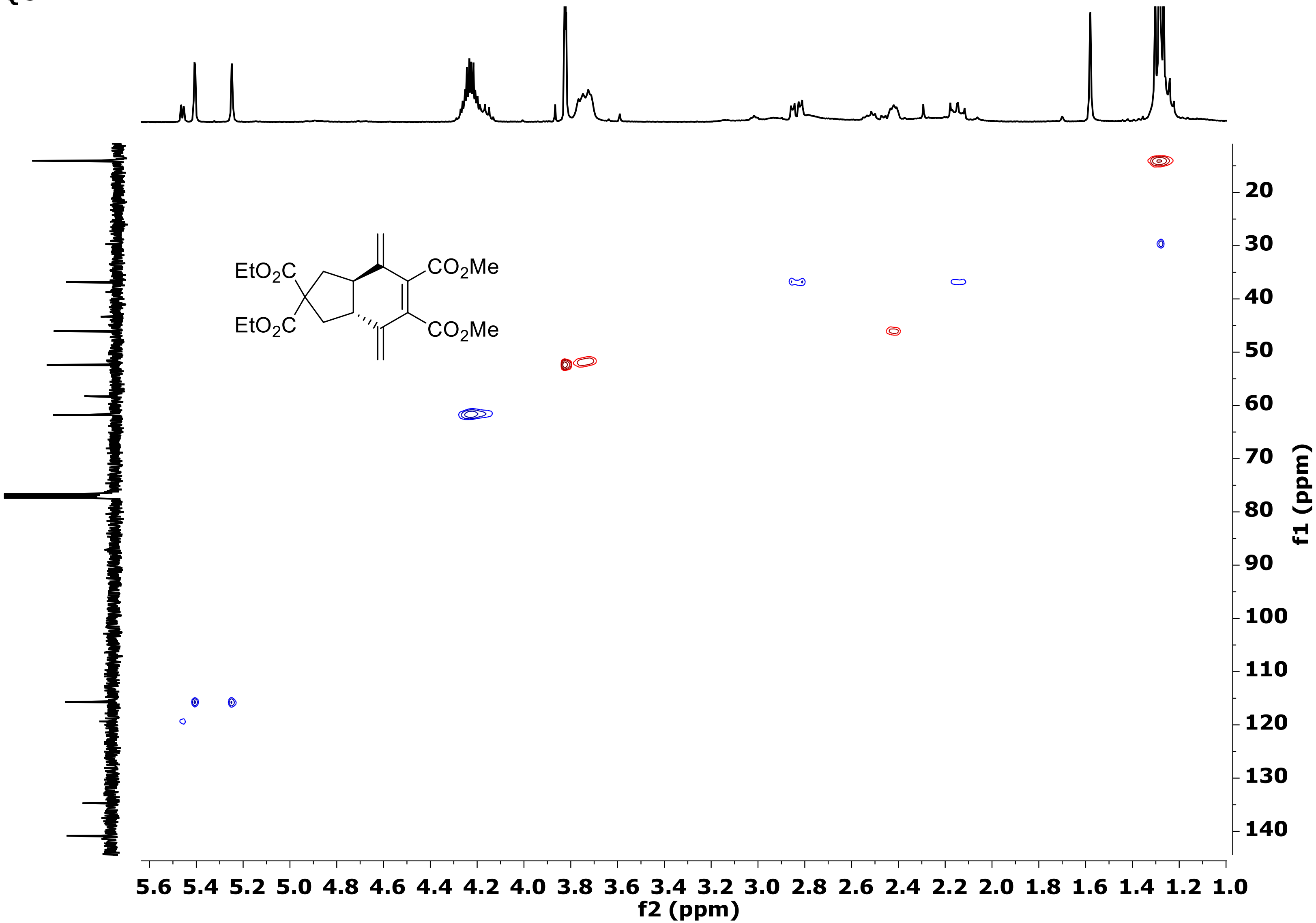
EtO2C
EtO2C
CO2Me
CO2Me
5.6 5.4 5.2 5.0 4.8 4.6 4.4 4.2 4.0 3.8 3.6 3.4 3.2 3.0 2.8 2.6 2.4 2.2 2.0 1.8 1.6 1.4 1.2 1.0
f2 (ppm)
20 30 40 50 60 70 80 90 100 110 120 130 140
f1 (ppm)

2D NMR COSY

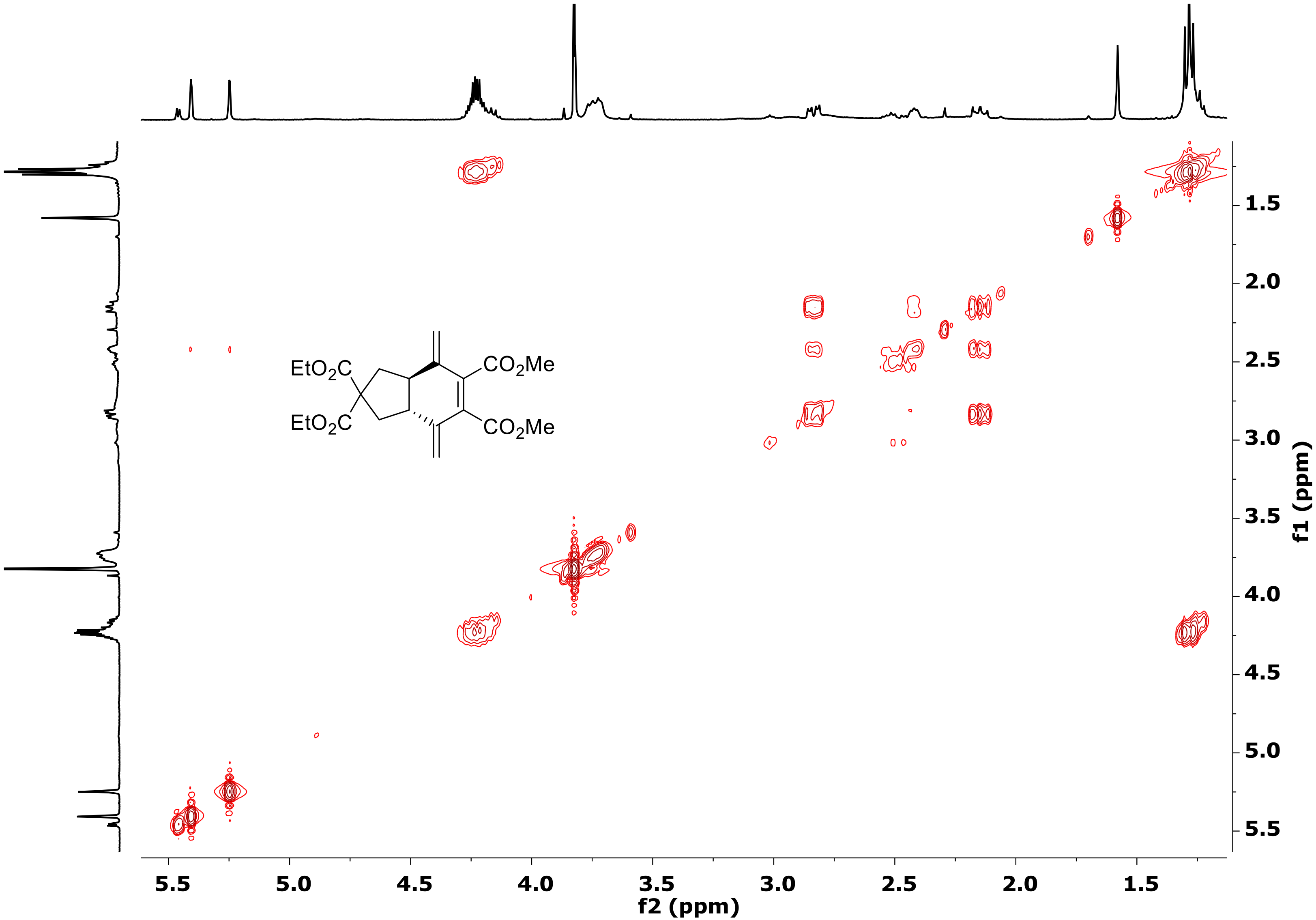

Product 3m

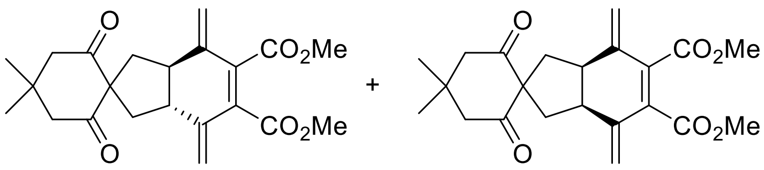

^{1}H NMR (400 MHz, $CDCl_3$)

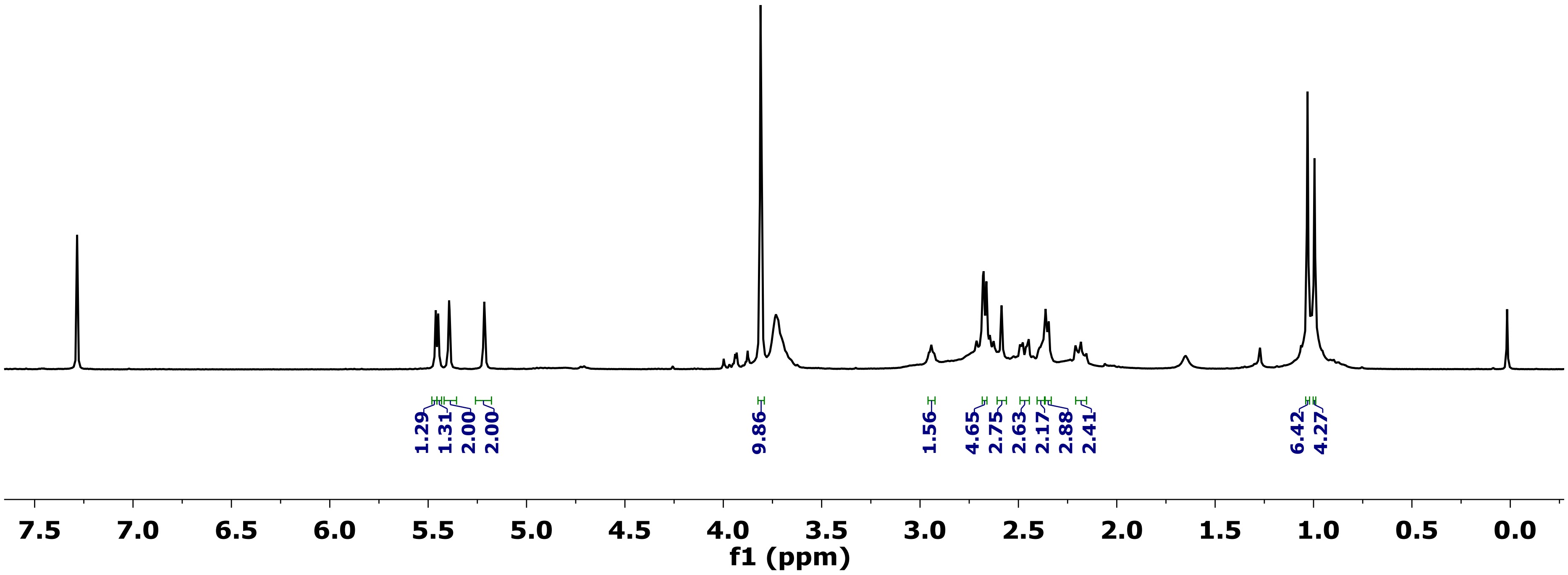

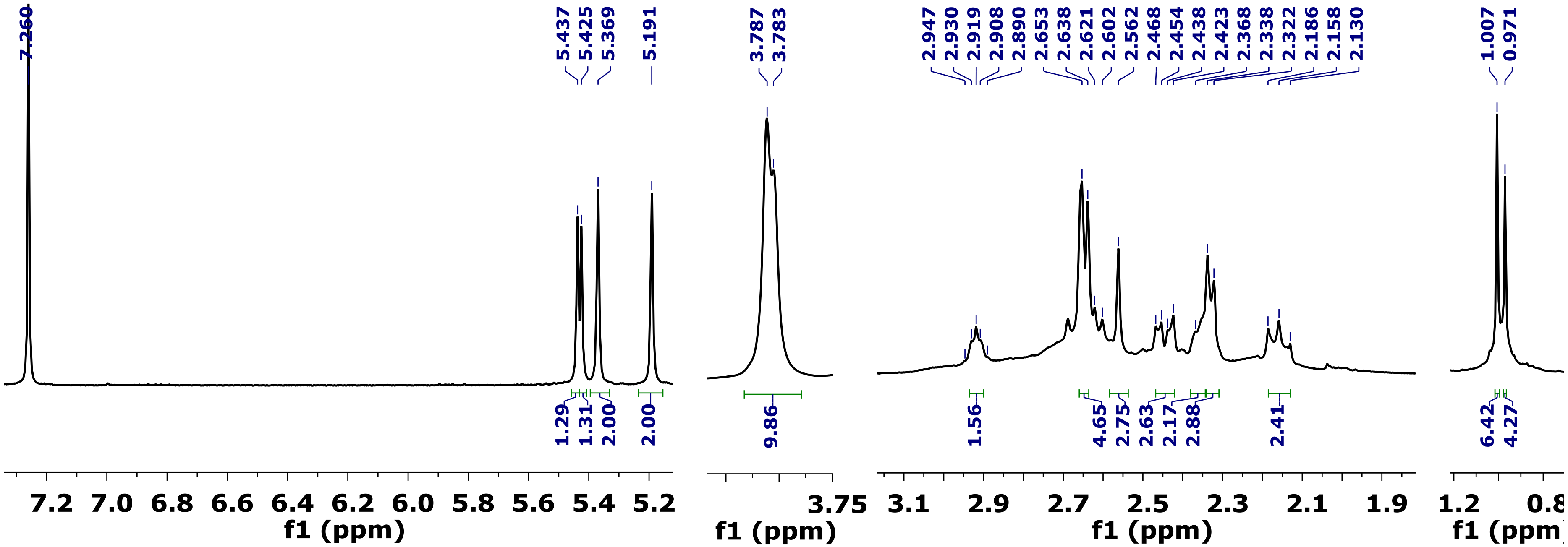
7.260
5.437
5.425
5.369
5.191
3.787
3.783
2.947
2.930
2.919
2.908
2.890
2.653
2.638
2.621
2.602
2.562
2.468
2.454
2.438
2.423
2.368
2.338
2.322
2.186
2.158
2.130
1.007
0.971
1.29
1.31
2.00
2.00
9.86
1.56
4.65
2.75
2.63
2.17
2.88
2.41
6.42
4.27
7.2 7.0 6.8 6.6 6.4 6.2 6.0 5.8 5.6 5.4 5.2
f1 (ppm)
3.75
f1 (ppm)
3.1 2.9 2.7 2.5 2.3 2.1 1.9
f1 (ppm)
1.2 0.8
f1 (ppm)

^{13}C NMR (101 MHz, $CDCl_3$)

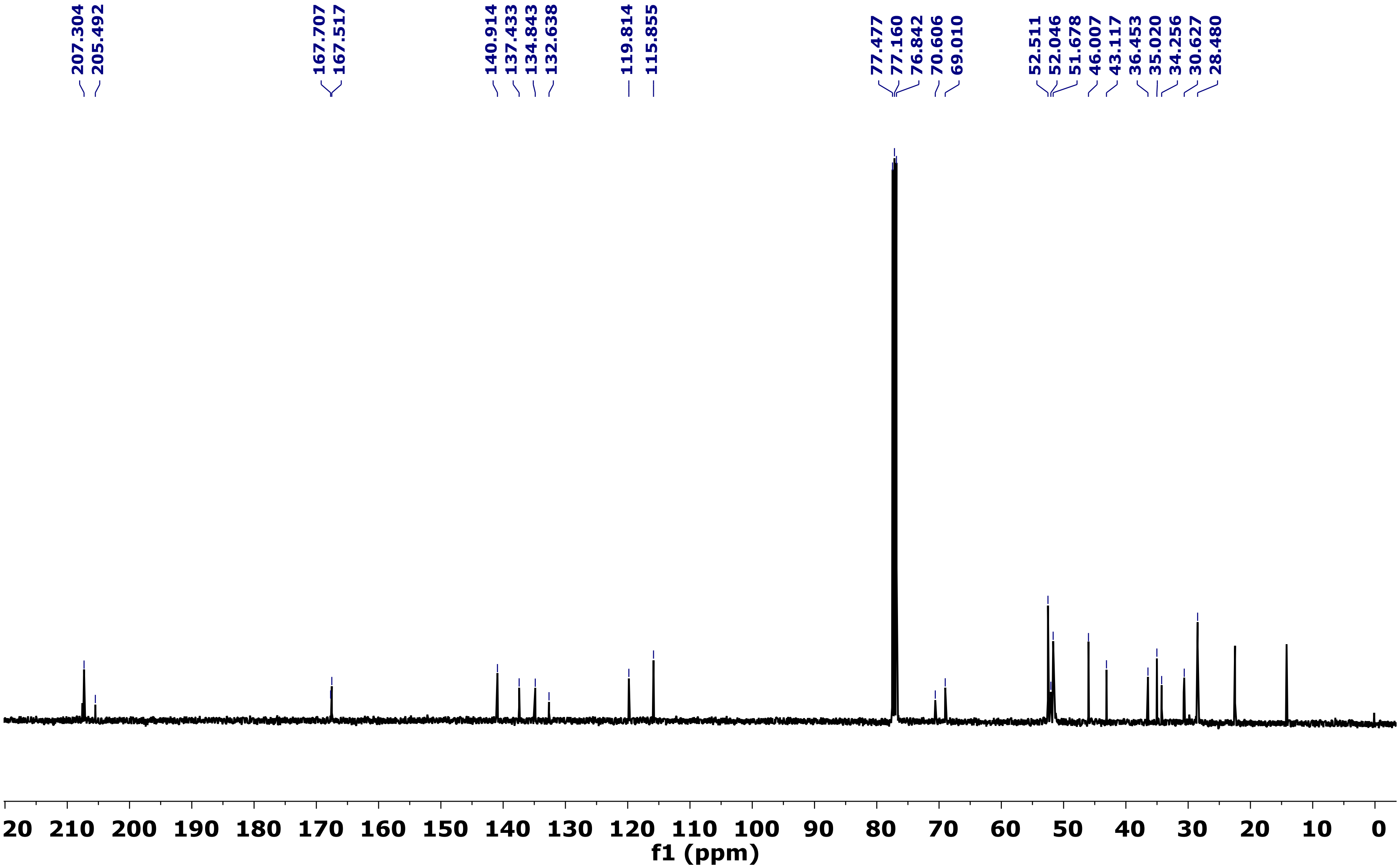

2D NMR HSQC

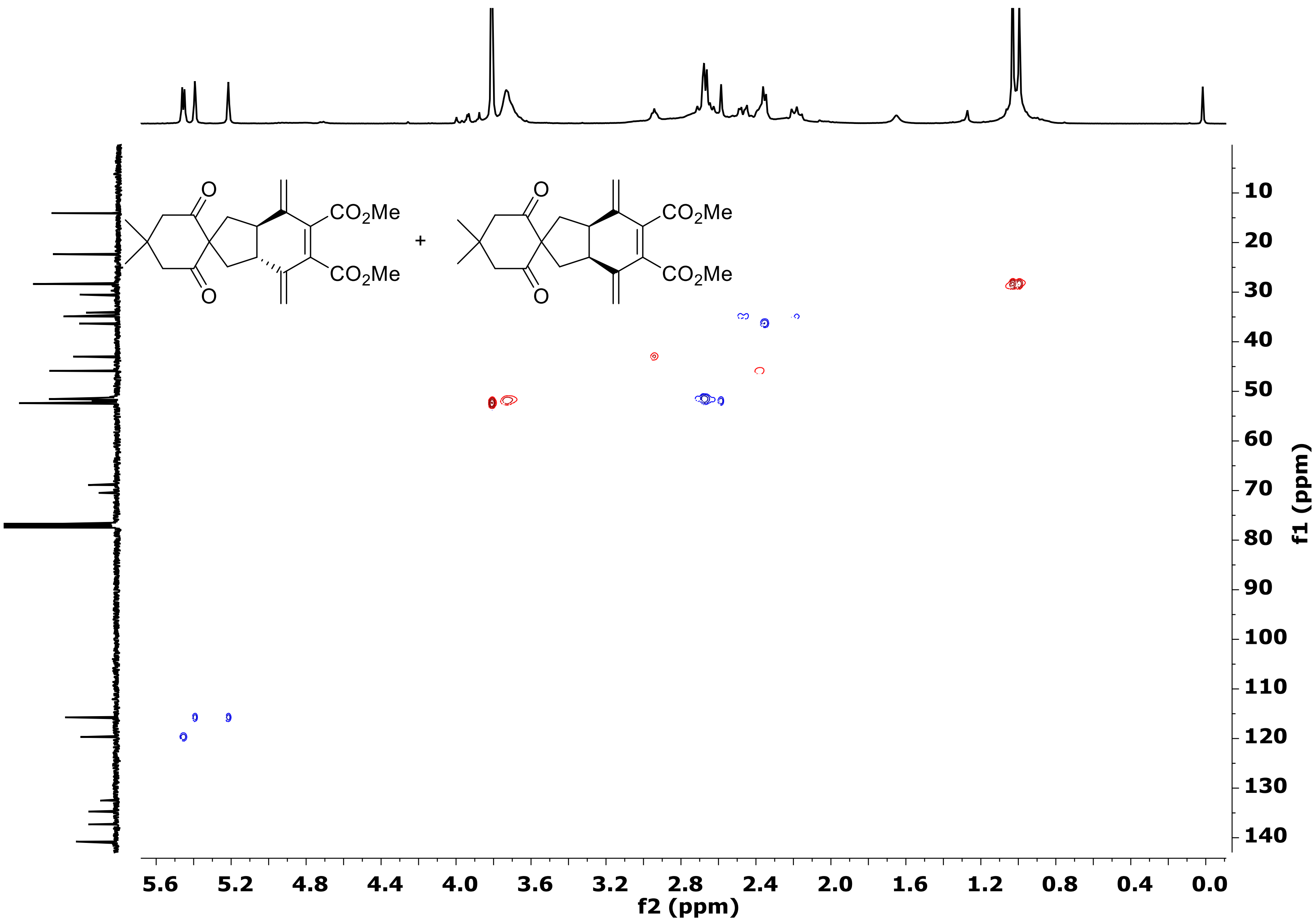

O
CO2Me
CO2Me
O
+
f1 (ppm)
f2 (ppm)

2D NMR COSY

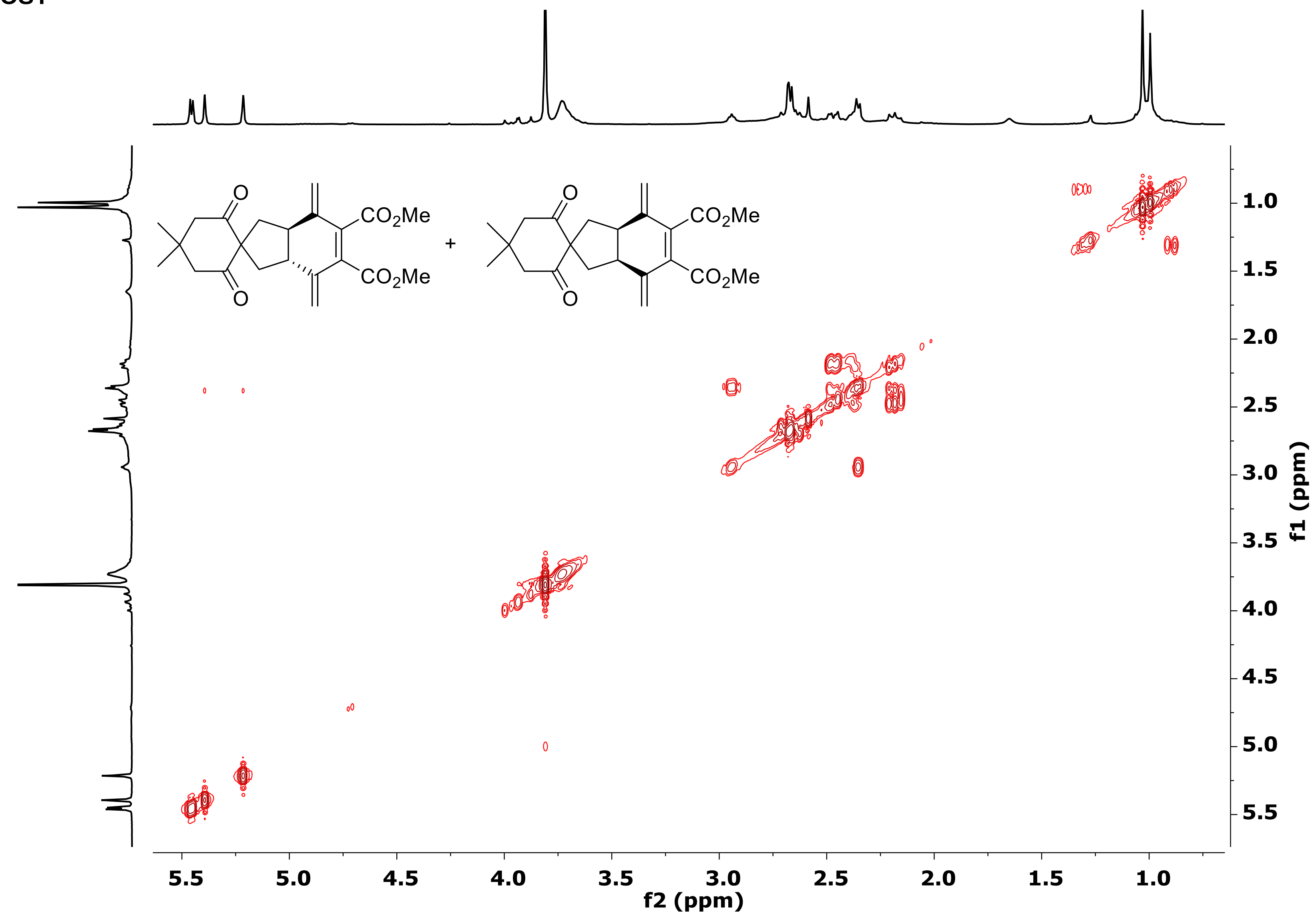

CO2Me
CO2Me
+
CO2Me
CO2Me
O
O
O
O
f1 (ppm)
f2 (ppm)
5.5
5.0
4.5
4.0
3.5
3.0
2.5
2.0
1.5
1.0

Product 3n

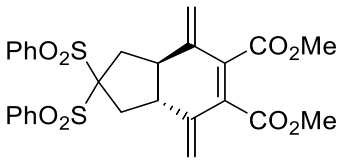

^{1}H NMR (400 MHz, CDCl3)

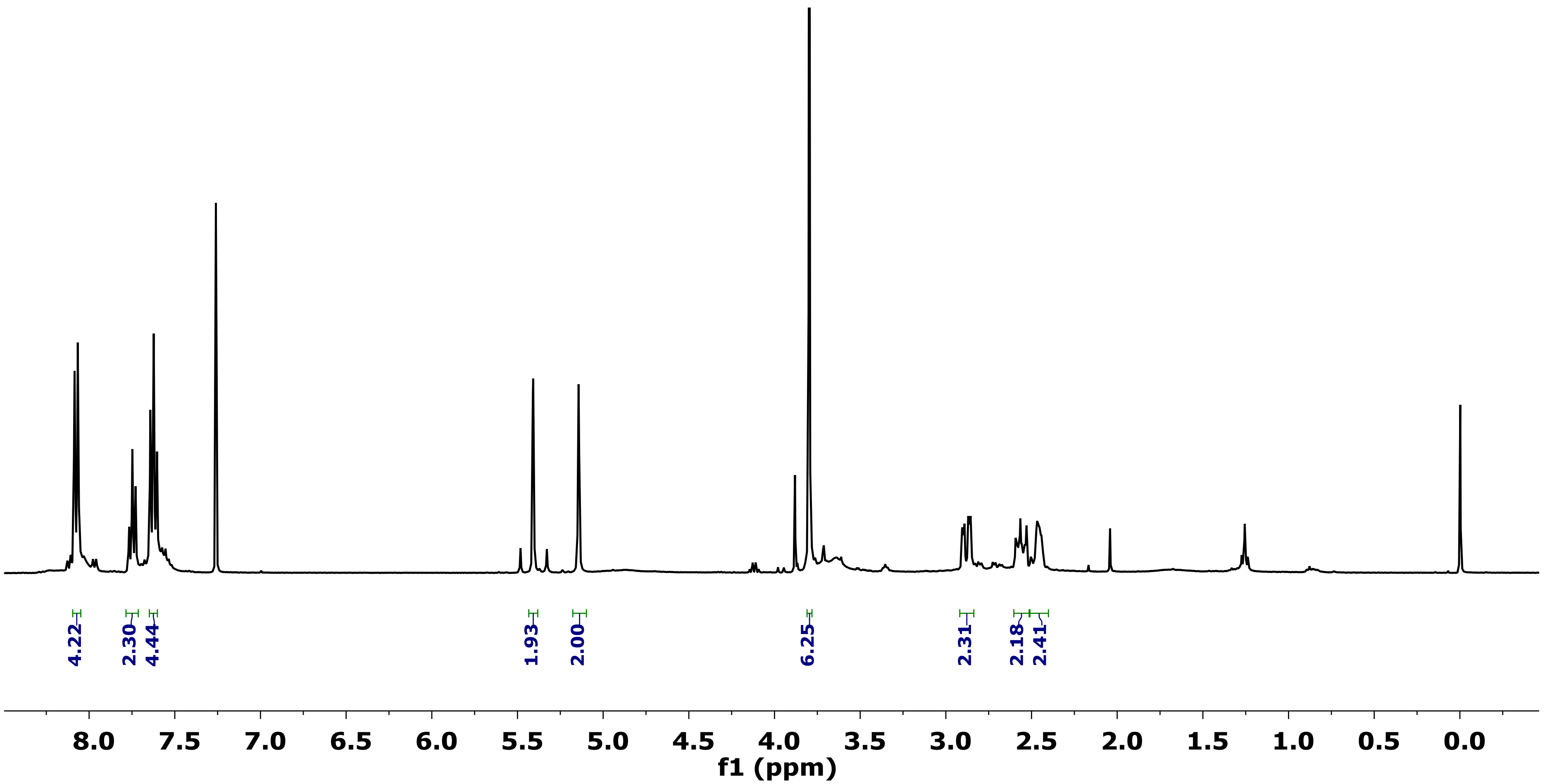

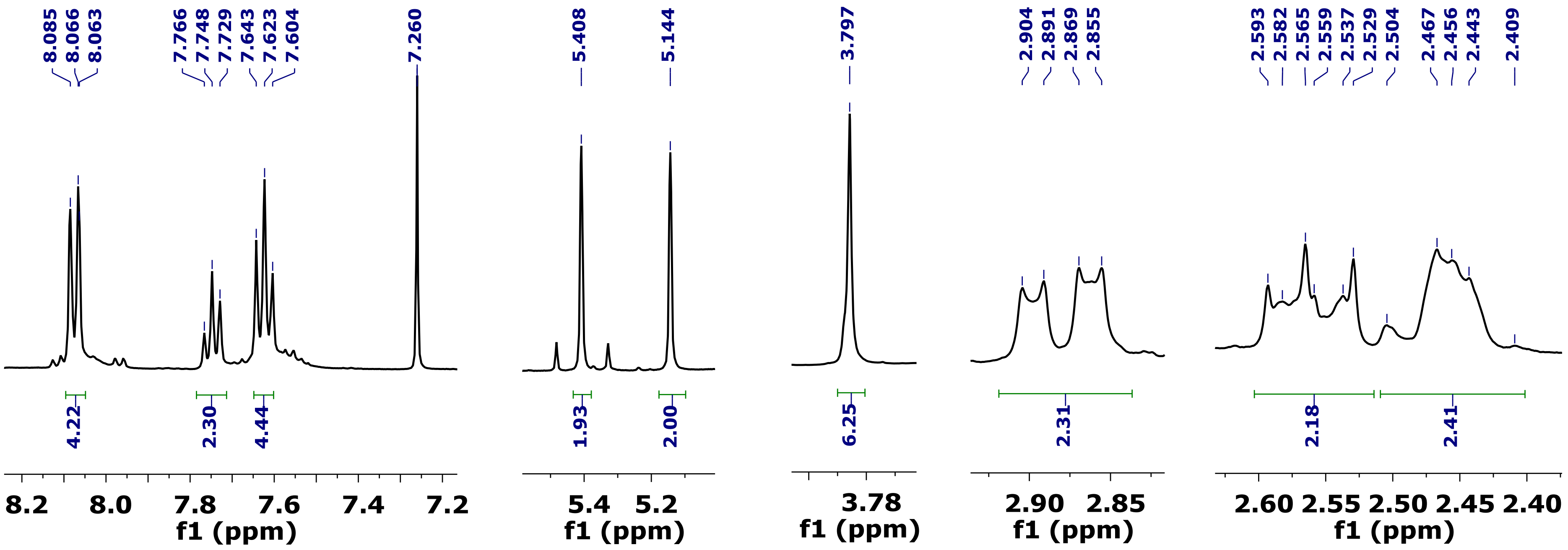

8.085
8.066
8.063
7.766
7.748
7.729
7.643
7.623
7.604
7.260
5.408
5.144
3.797
2.904
2.891
2.869
2.855
2.593
2.582
2.565
2.559
2.537
2.529
2.504
2.467
2.456
2.443
2.409
4.22
2.30
4.44
1.93
2.00
6.25
2.31
2.18
2.41
8.2 8.0 7.8 7.6 7.4 7.2
f1 (ppm)
5.4 5.2
f1 (ppm)
3.78
f1 (ppm)
2.90 2.85
f1 (ppm)
2.60 2.55 2.50 2.45 2.40
f1 (ppm)

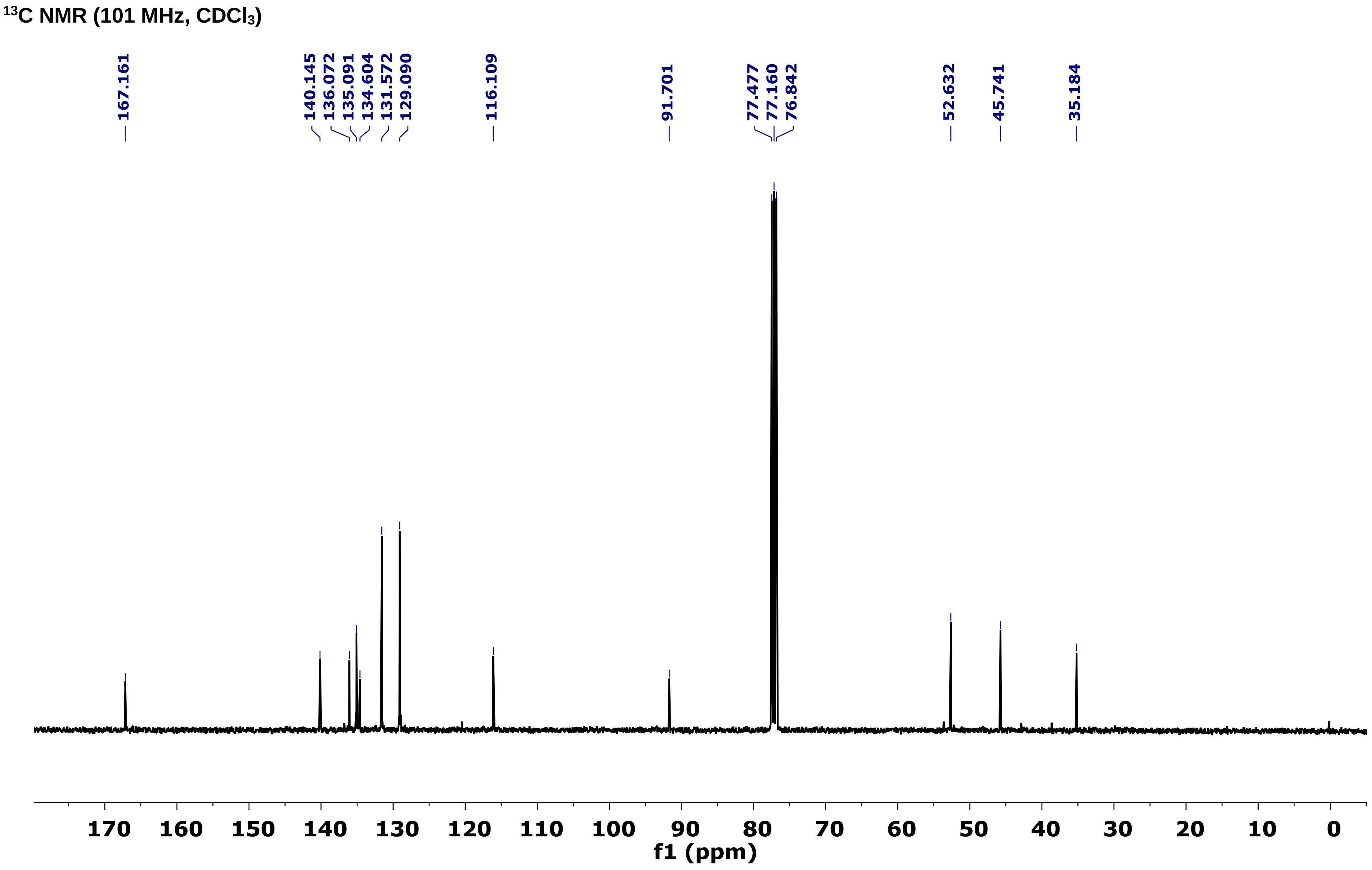

^{13}C NMR (101 MHz, CDCl$_3$)
167.161
140.145
136.072
135.091
134.604
131.572
129.090
116.109
91.701
77.477
77.160
76.842
52.632
45.741
35.184
170 160 150 140 130 120 110 100 90 80 70 60 50 40 30 20 10 0
f1 (ppm)

Product 3o

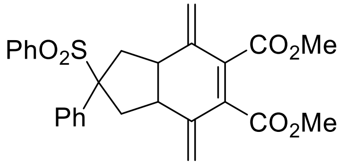

mix of diastereoisomers

^{1}H NMR (400 MHz, $CDCl_3$)

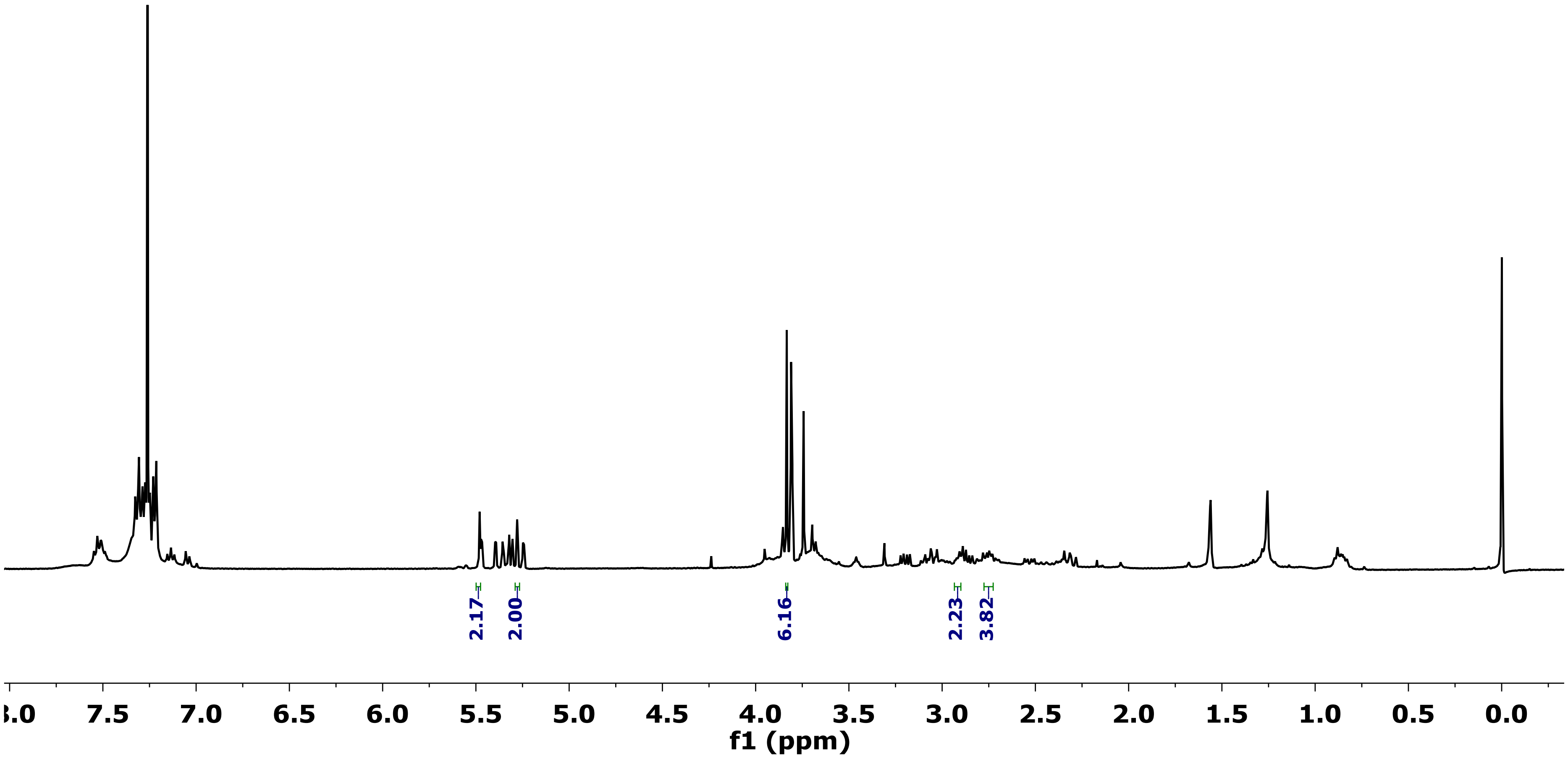

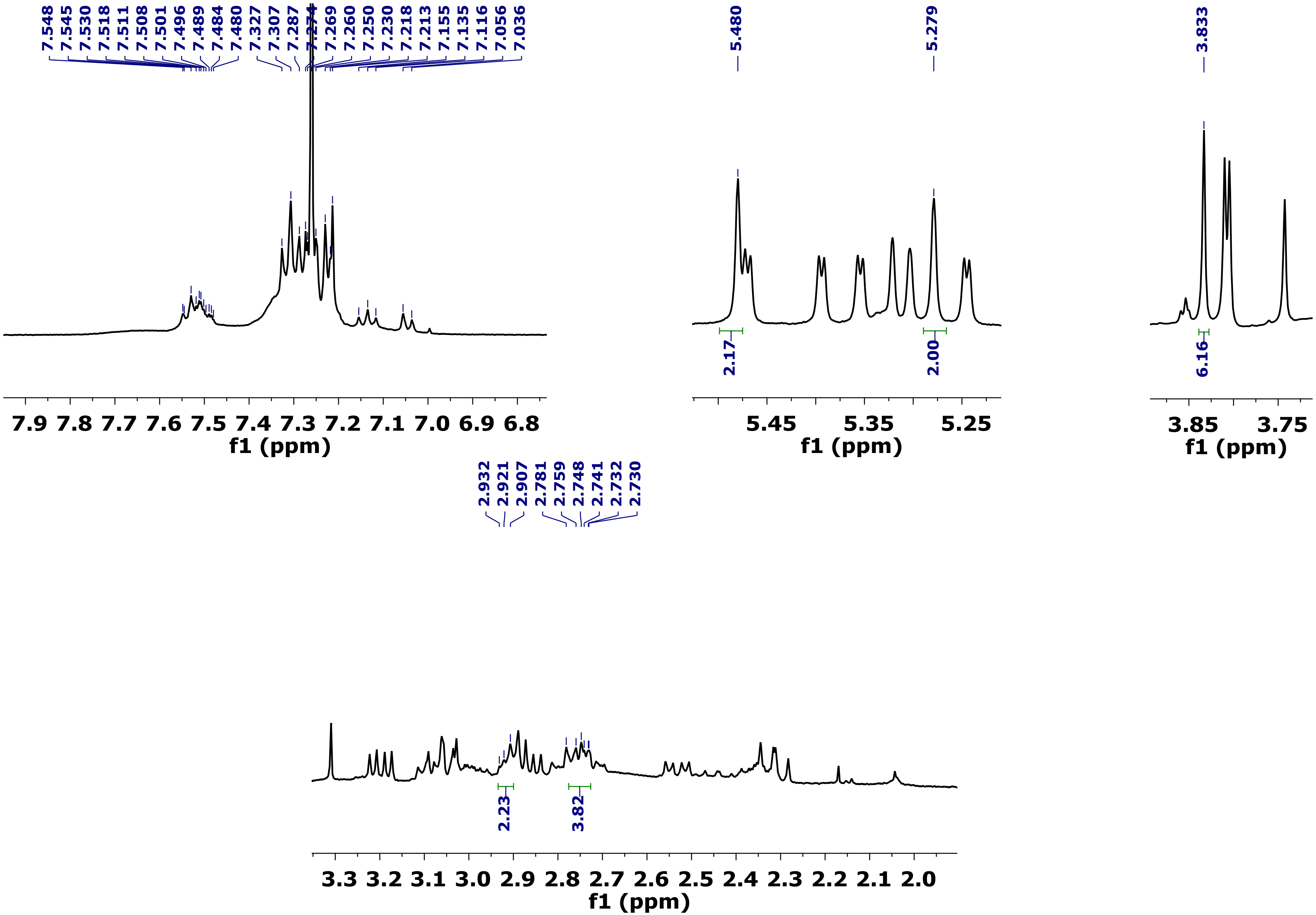
7.548
7.545
7.530
7.518
7.511
7.508
7.501
7.496
7.489
7.484
7.480
7.327
7.307
7.287
7.274
7.269
7.260
7.250
7.230
7.218
7.213
7.155
7.135
7.116
7.056
7.036
7.9 7.8 7.7 7.6 7.5 7.4 7.3 7.2 7.1 7.0 6.9 6.8
f1 (ppm)
5.480
5.279
2.17
2.00
5.45 5.35 5.25
f1 (ppm)
3.833
6.16
3.85 3.75
f1 (ppm)
2.932
2.921
2.907
2.781
2.759
2.748
2.741
2.732
2.730
2.23
3.82
3.3 3.2 3.1 3.0 2.9 2.8 2.7 2.6 2.5 2.4 2.3 2.2 2.1 2.0
f1 (ppm)

^{13}C NMR (101 MHz, $CDCl_3$)

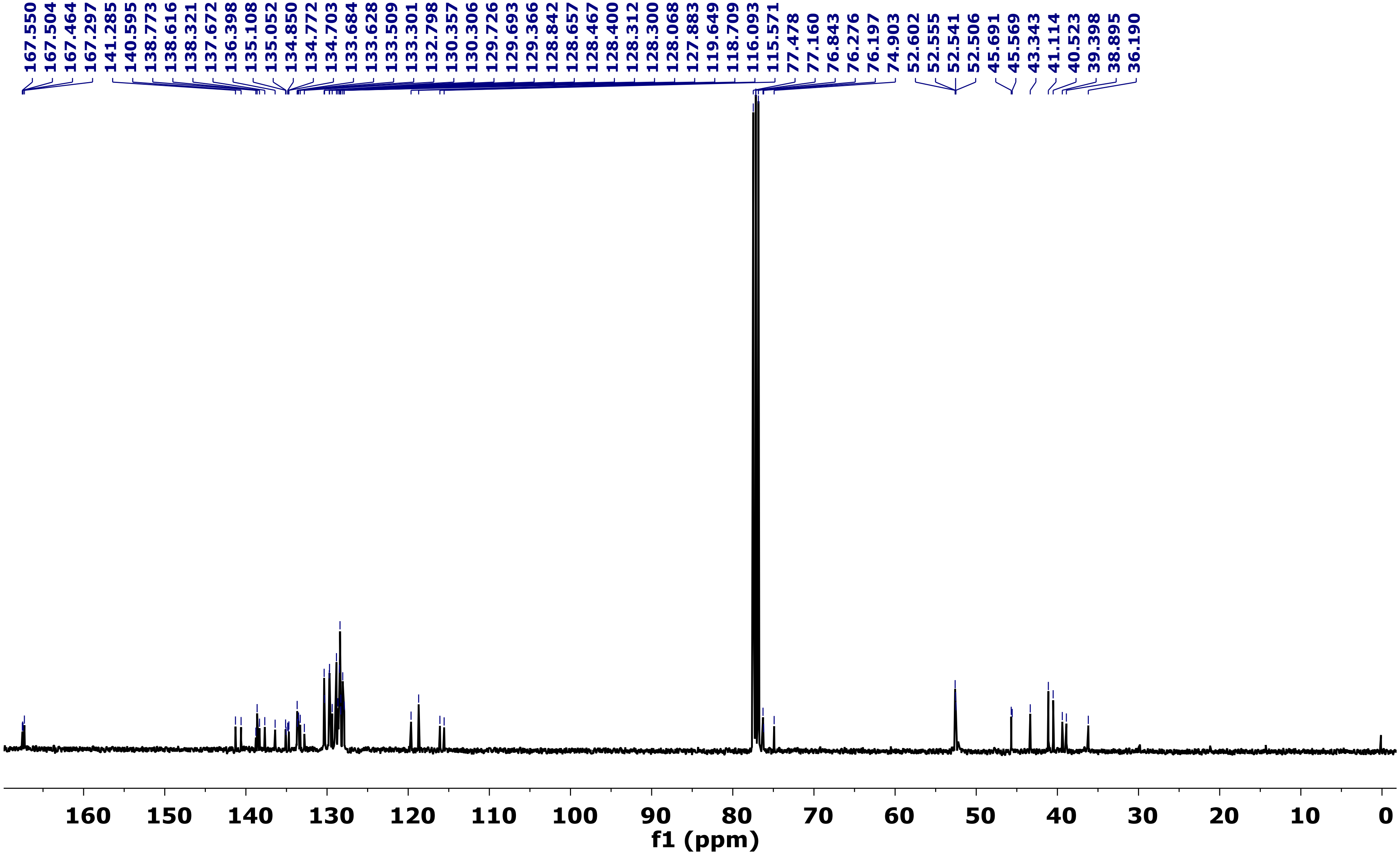

2D NMR HSQC

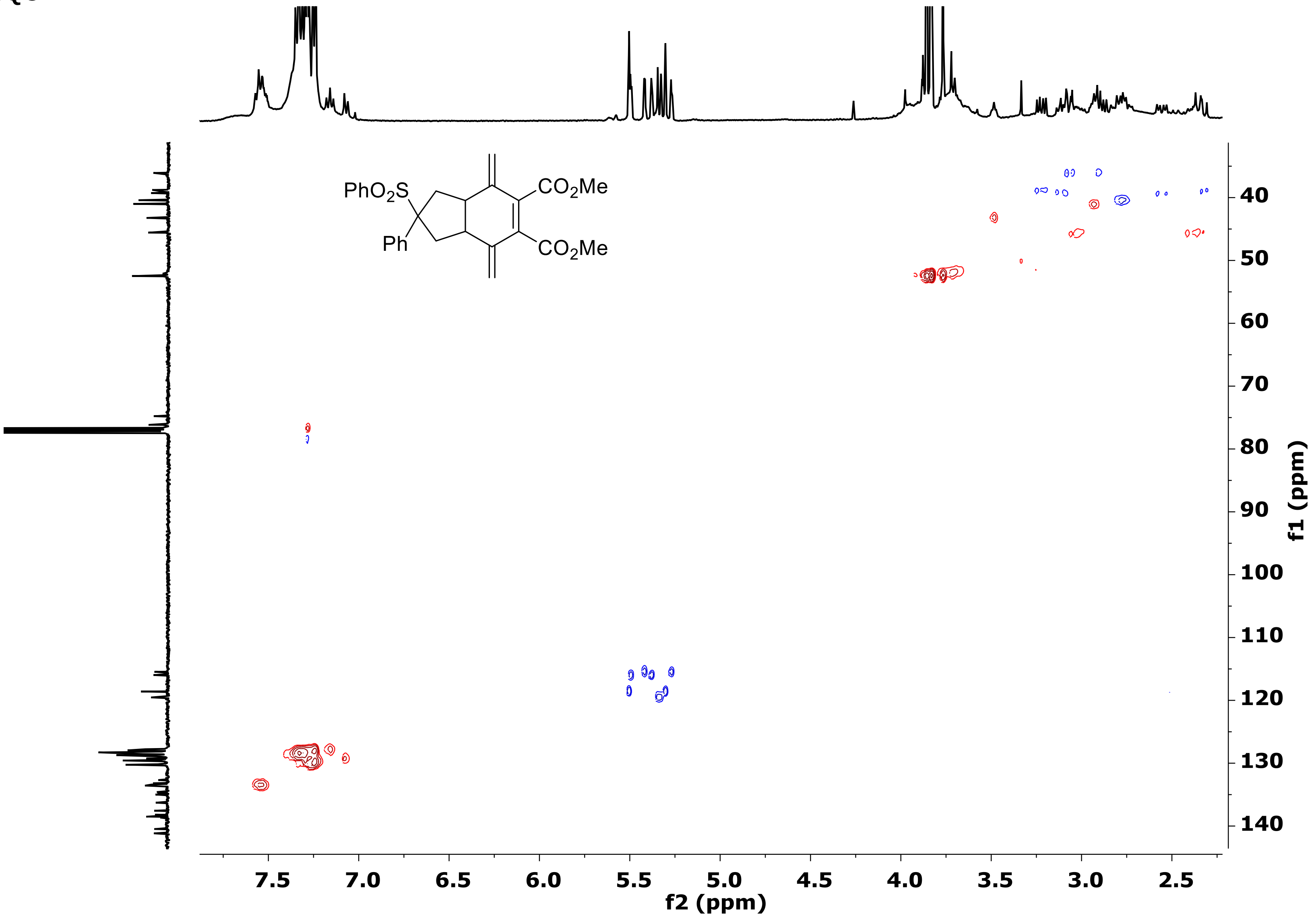
PhO2S
Ph
CO2Me
CO2Me
7.5 7.0 6.5 6.0 5.5 5.0 4.5 4.0 3.5 3.0 2.5
f2 (ppm)
40 50 60 70 80 90 100 110 120 130 140
f1 (ppm)

2D NMR COSY

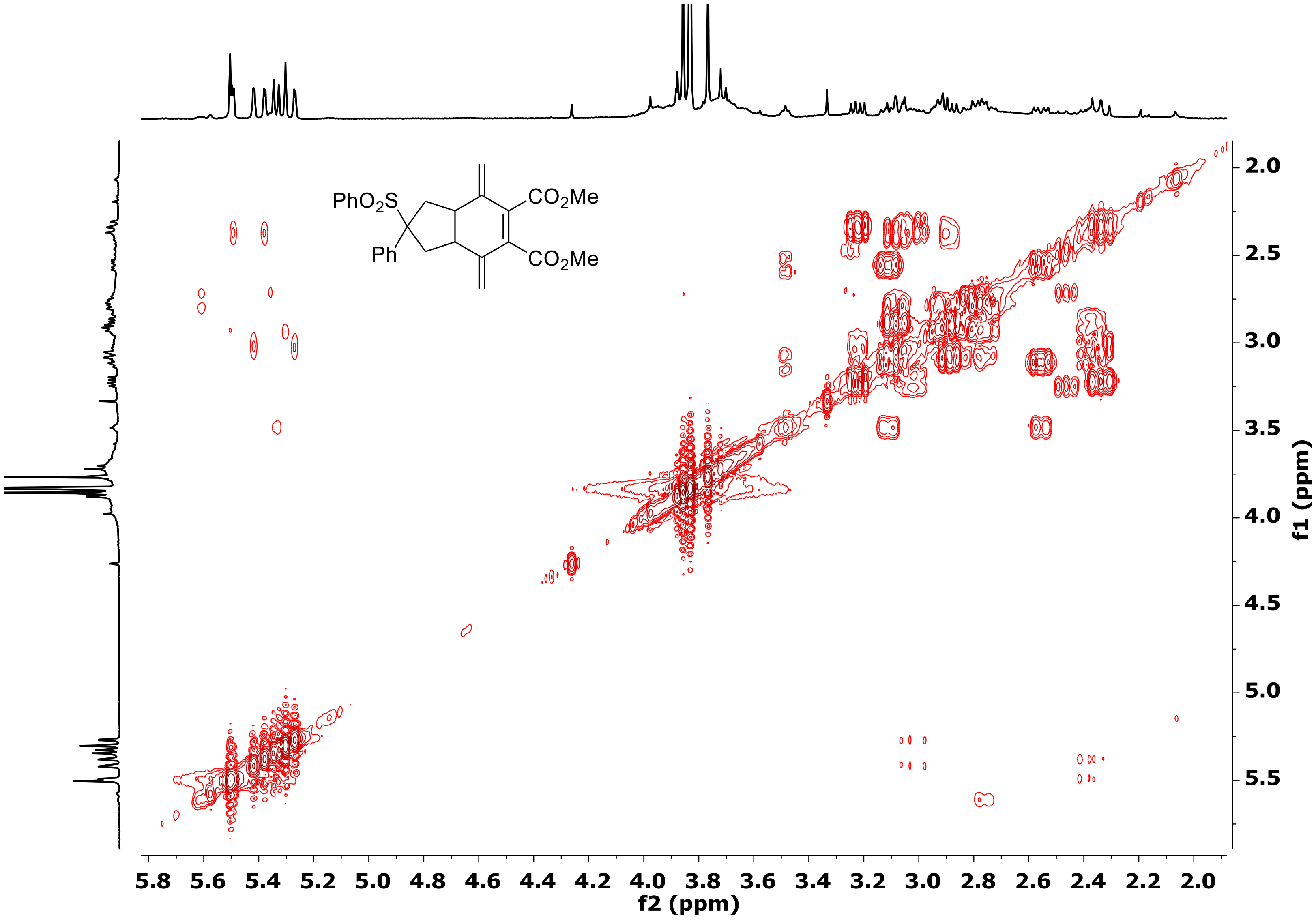

PhO2S
Ph
CO2Me
CO2Me
f2 (ppm)
f1 (ppm)
5.8 5.6 5.4 5.2 5.0 4.8 4.6 4.4 4.2 4.0 3.8 3.6 3.4 3.2 3.0 2.8 2.6 2.4 2.2 2.0
2.0 2.5 3.0 3.5 4.0 4.5 5.0 5.5

2D NMR COSY

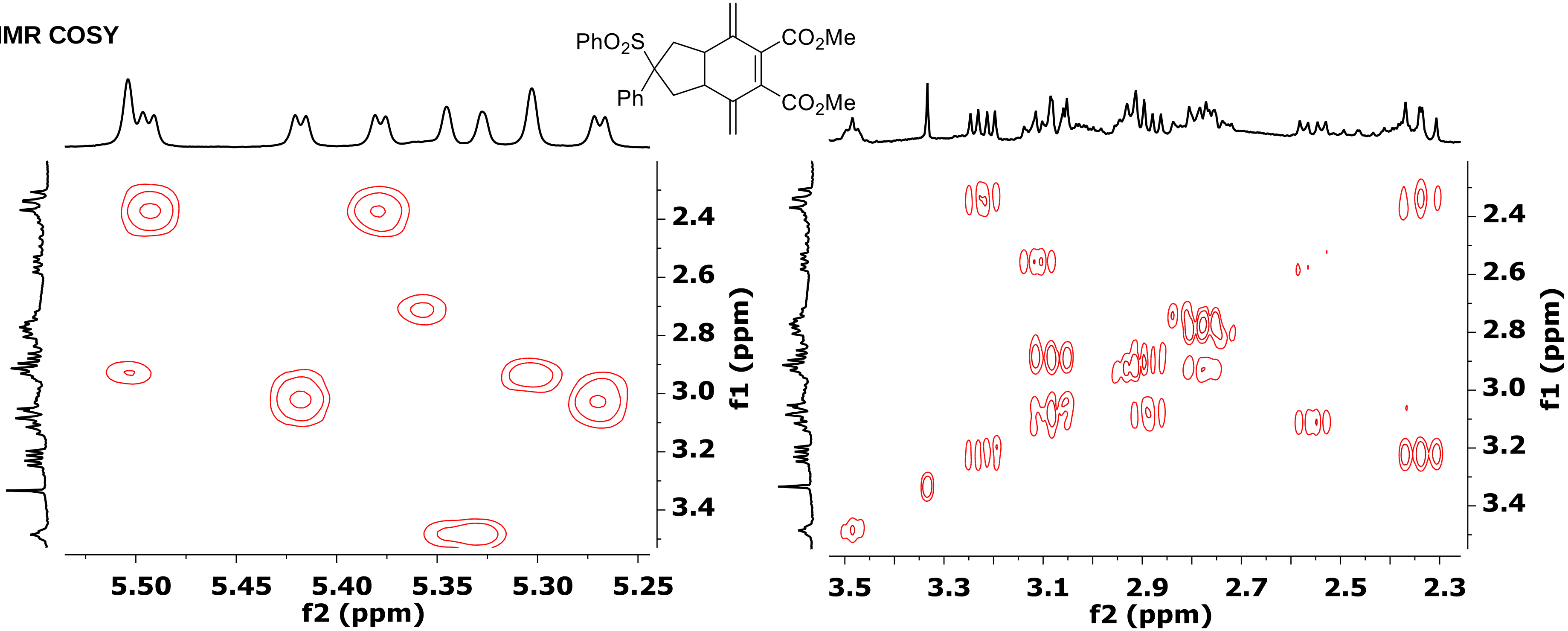
PhO2S
Ph
CO2Me
CO2Me
5.50 5.45 5.40 5.35 5.30 5.25
f2 (ppm)
2.4 2.6 2.8 3.0 3.2 3.4
f1 (ppm)
3.5 3.3 3.1 2.9 2.7 2.5 2.3
f2 (ppm)
2.4 2.6 2.8 3.0 3.2 3.4
f1 (ppm)

Product 3p

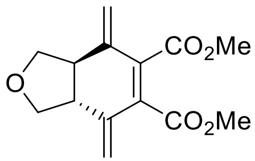

^{1}H NMR (400 MHz, $CDCl_3$)

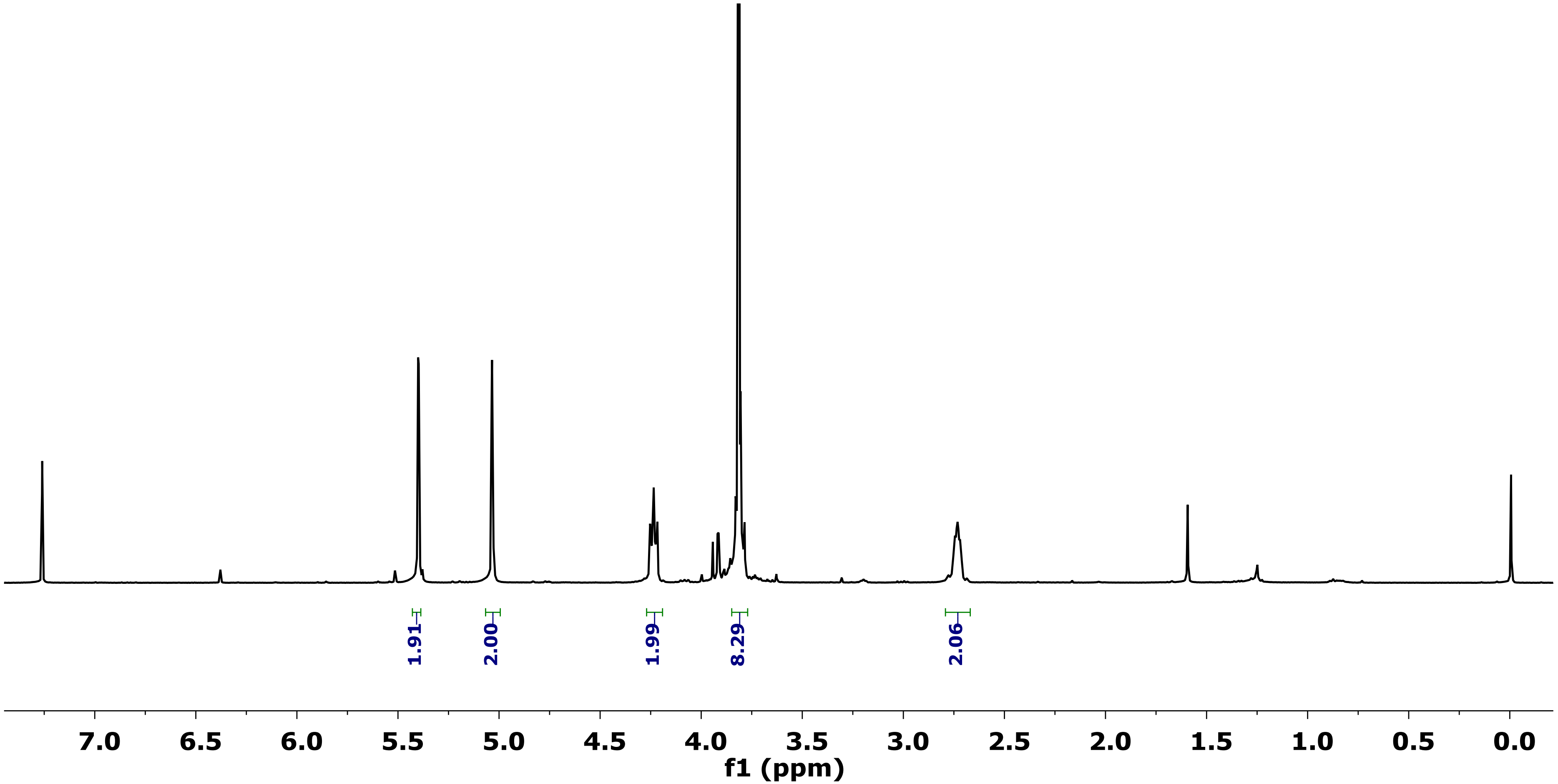

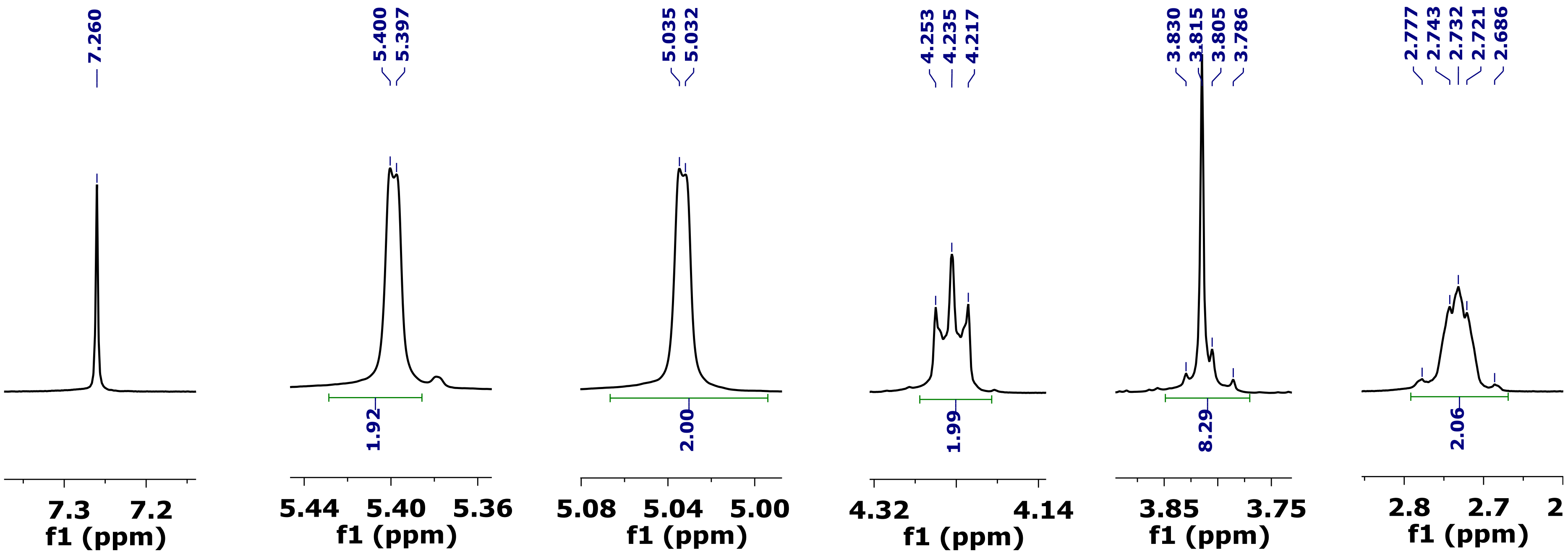
7.260
1.92
5.400
5.397
2.00
5.035
5.032
1.99
4.253
4.235
4.217
8.29
3.830
3.815
3.805
3.786
2.06
2.777
2.743
2.732
2.721
2.686
7.3 7.2
5.44 5.40 5.36
5.08 5.04 5.00
4.32 4.14
3.85 3.75
2.8 2.7 2
f1 (ppm)

^{13}C NMR (101 MHz, $CDCl_3$)

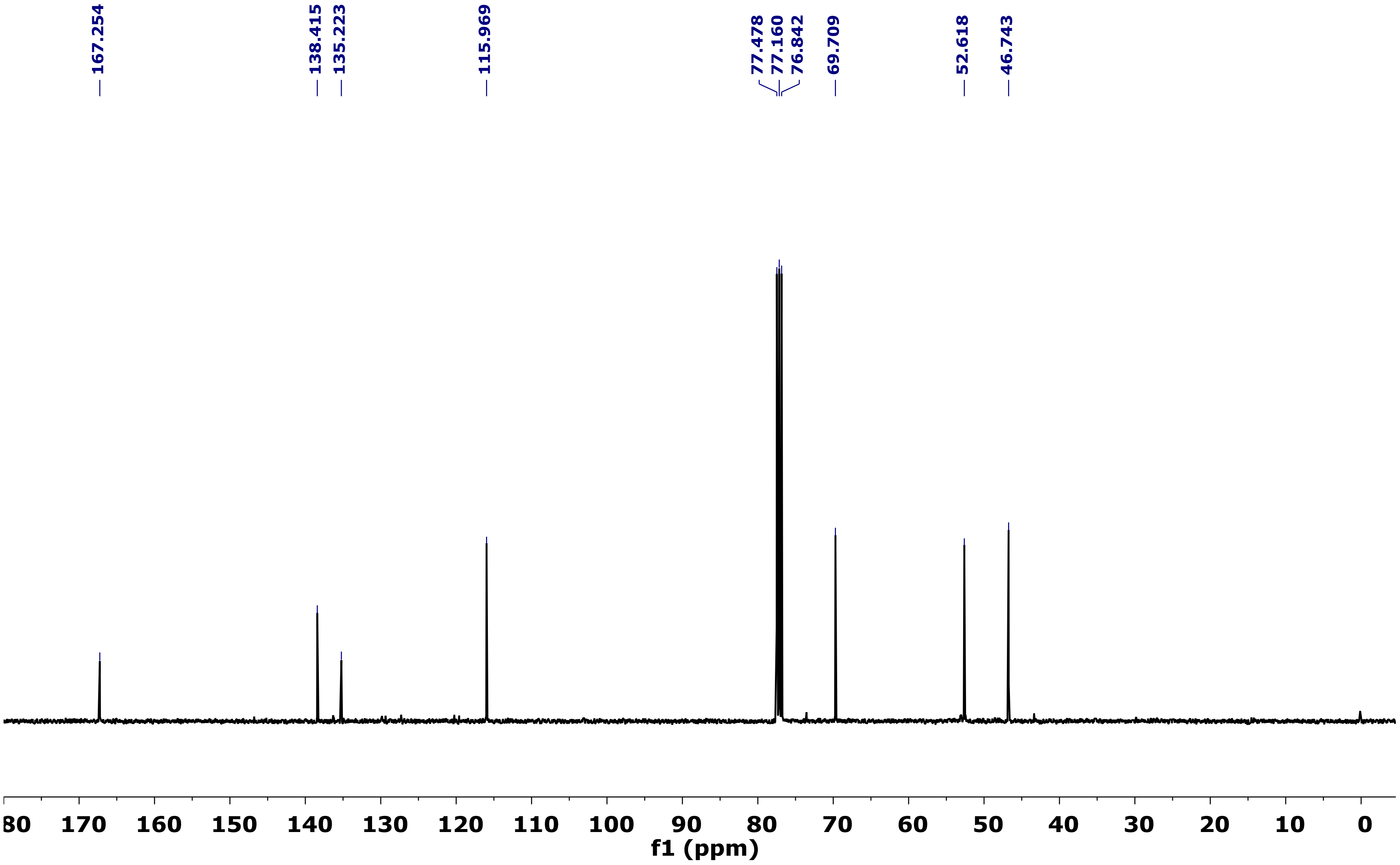

Crystal structure of compound 3a-*trans*

Colourless needle-like crystals of **3a-*trans*** were grown at room temperature via evaporation. Compound **3a-*trans*** was dissolved in acetone and contained within a long straight tube.

A total of 495 frames were collected. The total exposure time was 2.75 hours. The frames were integrated with the Bruker SAINT software package using a narrow-frame algorithm. The integration of the data using a monoclinic unit cell yielded a total of 16805 reflections to a maximum θ angle of 28.30° (0.75 Å resolution), of which 4929 were independent (average redundancy 3.409, completeness = 99.6%, R_{int} = 2.82%, R_{sig} = 3.10%) and 4671 (94.77%) were greater than 2σ(F^2). The final cell constants of a = 5.870(6) Å, b = 23.41(2) Å, c = 7.254(8) Å, β = 91.59(2)°, volume = 996.4(18) $Å^3$, are based upon the refinement of the XYZ-centroids of 8596 reflections above 20 σ(I) with 5.618° < 2θ < 56.42°. Data were corrected for absorption effects using the Multi-Scan method (SADABS). The ratio of minimum to maximum apparent transmission was 0.903. The calculated minimum and maximum transmission coefficients (based on crystal size) are 0.6733 and 0.7457.

The structure was solved and refined using the Bruker SHELXTL Software Package, using the space group P 1 21 1, with Z = 2 for the formula unit, $C_{21}H_{23}NO_6S$. The final anisotropic full-matrix least-squares refinement on F^2 with 284 variables converged at R1 = 3.99%, for the observed data and wR2 = 9.04% for all data. The goodness-of-fit was 1.077. The largest peak in the final difference electron density synthesis was 0.303 $e^-/Å^3$ and the largest hole was -0.238 $e^-/Å^3$ with an RMS deviation of 0.045 $e^-/Å^3$. On the basis of the final model, the calculated density was 1.392 g/cm^3 and F(000), 440 e^-.

Figure S7. ORTEP representation of **3a-*trans*** with probability level of 50%

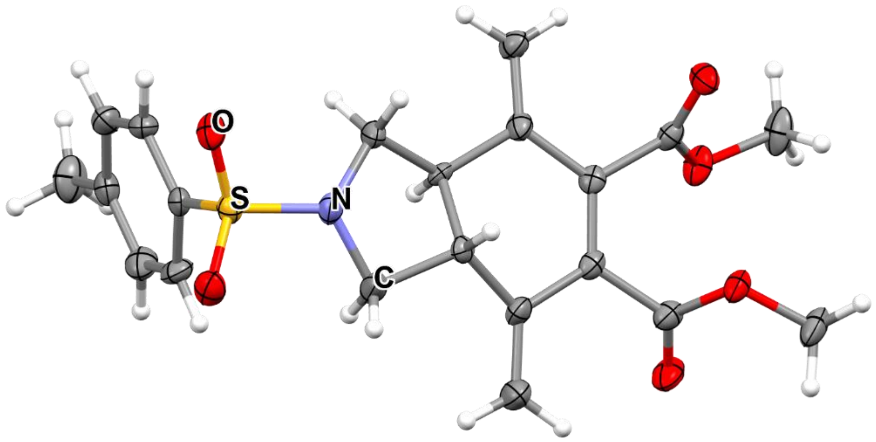

Table S2. Sample and crystal data for **3a-*trans*.**

**Identification code**	9257	
**Chemical formula**	$C_{21}H_{23}NO_6S$	
**Formula weight**	417.46 g/mol	
**Temperature**	100(2) K	
**Wavelength**	0.71076 Å	
**Crystal size**	0.070 x 0.110 x 0.200 mm	
**Crystal habit**	colorless block	
**Crystal system**	monoclinic	
**Space group**	P 1 21 1	
**Unit cell dimensions**	a = 5.870(6) Å	α = 90°
	b = 23.41(2) Å	β = 91.59(2)°
	c = 7.254(8) Å	γ = 90°
**Volume**	996.4(18) $Å^3$	
**Z**	2	
**Density (calculated)**	1.392 g/cm^3	
**Absorption coefficient**	0.201 mm^{-1}	
**F(000)**	440	

Table S3. Data collection and structure refinement for **3a-*trans***.

**Diffractometer**	D8 QUEST ECO three-circle diffractometer	
**Radiation source**	Ceramic x-ray tube (Mo Kα, λ = 0.71076 Å)	
**Theta range for data collection**	2.94 to 28.30°	
**Index ranges**	-7<=h<=7, -31<=k<=30, -9<=l<=9	
**Reflections collected**	16805	
**Independent reflections**	4929 [R(int) = 0.0282]	
**Coverage of independent reflections**	99.6%	
**Absorption correction**	Multi-Scan	
**Max. and min. transmission**	0.7457 and 0.6733	
**Structure solution technique**	direct methods	
**Structure solution program**	SHELXT 2014/5 (Sheldrick, 2014)	
**Refinement method**	Full-matrix least-squares on F^2	
**Refinement program**	SHELXL-2017/1 (Sheldrick, 2017)	
**Function minimized**	$\Sigma\ w(F_o^2 - F_c^2)^2$	
**Data / restraints / parameters**	4929 / 1 / 284	
**Goodness-of-fit on F^2**	1.077	
**Final R indices**	4671 data; I>2σ(I)	R1 = 0.0399, wR2 = 0.0890
	all data	R1 = 0.0428, wR2 = 0.0904
**Weighting scheme**	$w=1/[\sigma^2(F_o^2)+(0.0400P)^2+0.3564P]$<br>where $P=(F_o^2+2F_c^2)/3$	
**Absolute structure parameter**	0.05(3)	
**Largest diff. peak and hole**	0.303 and -0.238 $eÅ^{-3}$	
**R.M.S. deviation from mean**	0.045 $eÅ^{-3}$	

Table S4. Atomic coordinates and equivalent isotropic atomic displacement parameters (Å2) for **3a-*trans***.

U(eq) is defined as one third of the trace of the orthogonalized U_{ij} tensor.

	**x/a**	**y/b**	**z/c**	**U(eq)**
S19	0.92648(11)	0.40683(3)	0.38099(9)	0.02199(15)
O11	0.6313(3)	0.70616(9)	0.4608(3)	0.0275(5)
O12	0.9576(3)	0.65915(10)	0.5162(3)	0.0275(5)
O15	0.9862(4)	0.57467(10)	0.8994(3)	0.0338(5)
O16	0.7769(3)	0.65502(9)	0.8791(3)	0.0254(4)
C18	0.5857(6)	0.49342(15)	0.8918(5)	0.0363(8)
O20	0.8789(4)	0.37189(10)	0.5382(3)	0.0319(5)
O21	0.7447(3)	0.42789(9)	0.2640(3)	0.0278(5)
C29	0.4282(5)	0.60845(13)	0.2232(4)	0.0249(6)
N6	0.0645(4)	0.46214(11)	0.4596(3)	0.0256(5)
C1	0.6159(4)	0.60618(12)	0.5337(4)	0.0185(5)
C2	0.6594(4)	0.57735(12)	0.6938(4)	0.0196(5)
C3	0.5445(5)	0.52330(13)	0.7384(4)	0.0246(6)
C4	0.3193(13)	0.5188(3)	0.6201(9)	0.0185(17)
C4'	0.4187(11)	0.4973(3)	0.5800(7)	0.0210(15)
C5	0.2212(6)	0.45639(15)	0.6224(4)	0.0359(8)
C7	0.1403(5)	0.50778(12)	0.3336(4)	0.0240(6)
C8	0.3740(12)	0.5257(3)	0.4168(9)	0.0153(17)
C8'	0.2836(10)	0.5429(2)	0.4751(8)	0.0152(13)
C9	0.4542(5)	0.58548(12)	0.3896(4)	0.0220(6)
C10	0.7303(4)	0.66255(13)	0.5000(4)	0.0205(5)
C13	0.0746(5)	0.71409(16)	0.5054(5)	0.0361(8)
C14	0.8273(5)	0.60071(13)	0.8347(4)	0.0243(6)
C17	0.9445(6)	0.68190(15)	0.0012(5)	0.0360(8)
C22	0.1106(5)	0.36818(11)	0.2390(4)	0.0192(5)
C23	0.2884(5)	0.33629(14)	0.3165(4)	0.0269(6)
C24	0.4396(5)	0.30928(14)	0.2025(5)	0.0306(7)
C25	0.4144(5)	0.31235(13)	0.0118(4)	0.0255(6)
C26	0.2296(6)	0.34212(13)	0.9366(4)	0.0271(6)
C27	0.0795(5)	0.37091(12)	0.0488(4)	0.0236(6)
C28	0.5839(6)	0.28401(17)	0.8873(6)	0.0403(8)

Crystal structure of compound RhL4

The crystals were placed in oil, and a single crystal was selected, mounted on a glass fibre and placed in a low-temperature N_2 stream.

X-ray diffraction data collection was carried out on a Bruker PHOTON III DUO CPAD diffractometer equipped with an Oxford Cryosystem liquid N_2 device, using Mo-Kα radiation (λ = 0.71073 Å). The crystal-detector distance was 37mm. The cell parameters were determined (APEX3 software) [16] from reflections taken from 1 set of 180 frames at 1s exposure. The structure was solved using the program SHELXT-2014 [17]. The refinement and all further calculations were carried out using SHELXL-2018 [18]. The H-atoms were included in calculated positions and treated as riding atoms using SHELXL default parameters. The non-H atoms were refined anisotropically, using weighted full-matrix least-squares on F^2. A semi-empirical absorption correction was applied using SADABS in APEX3 [1]; transmission factors: T_{min}/T_{max} = 0.6583/0.7456.

A total of 1233 frames were collected. The total exposure time was 3 hours. The frames were integrated with the Bruker SAINT software package using a narrow-frame algorithm. The integration of the data using a monoclinic unit cell yielded a total of 48851 reflections to a maximum θ angle of 27.92° (0.76 Å resolution), of which 5621 were independent (average redundancy 5, completeness = 99.6%, R_{int} = 5.88%, R_{sig} = 11.58%) and 4175(74.27%) were greater than $2\sigma(F^2)$. The final cell constants of a = 34.0009(11) Å, b = 6.9081(2) Å, c = 22.8106(8) Å, β = 118.467(2)°, volume = 4710.0(3) $Å^3$, are based upon the refinement of the XYZ-centroids of 6996v reflections above 20 σ(I) with 5.02° < 2θ < 54.24°. Data were corrected for absorption effects using the Multi-Scan method (SADABS). The ratio of minimum to maximum apparent transmission was 0.883. The calculated minimum and maximum transmission coefficients (based on crystal size) are 0.6583 and 0.7456.

The structure was solved and refined using the Bruker SHELXTL Software Package, using the space group C 1 2/c 1, with Z = 8 for the formula unit, $C_{24}H_{36}BrN_2RhS$. The final anisotropic full-matrix least-squares refinement on F^2 with 263 variables converged at R1 = 4.20%, for the observed data and wR2 = 8.70% for all data. The goodness-of-fit was 1.004. The largest peak in the final difference electron density synthesis was 0.805 $e^-/Å^3$ and the largest hole was -0.952 $e^-/Å^3$ with an RMS deviation of 0.136 $e^-/Å^3$. On the basis of the final model, the calculated density was 1.600 g/cm^3 and F(000), 2320 e^-.

Figure S8. ORTEP representation of **RhL4** with probability level of 50%, Hydrogen are omitted for clarity.

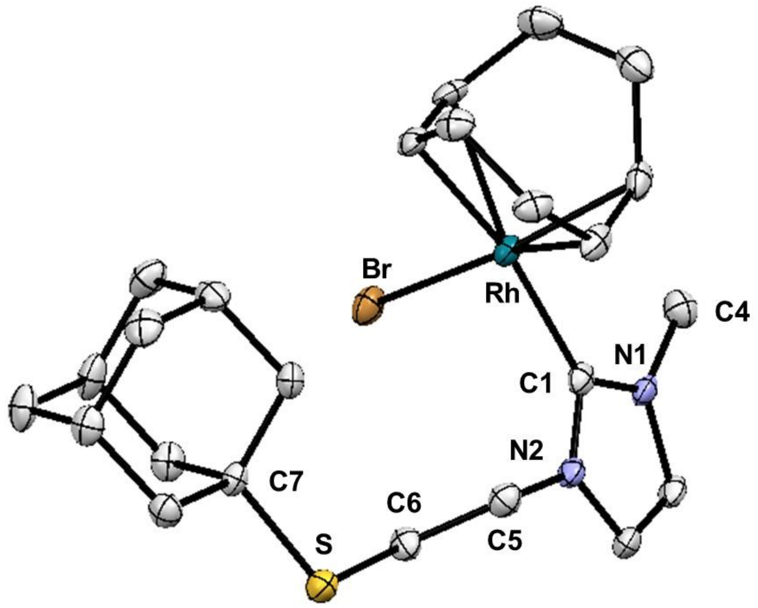

Table S5. Sample and crystal data for **RhL4.**

**Identification code**	esbta_a	
**Chemical formula**	$C_{24}H_{36}BrN_2RhS$	
**Formula weight**	567.43 g/mol	
**Temperature**	120(2) K	
**Wavelength**	0.71073 Å	
**Crystal size**	0.070 x 0.110 x 0.200 mm	
**Crystal habit**	yellow needle	
**Crystal system**	monoclinic	
**Space group**	C 2/c	
**Unit cell dimensions**	a = 34.0009(11) Å	α = 90°
	b = 6.9081(2) Å	β = 118.467(2)°
	c = 22.8106(8) Å	γ = 90°
**Volume**	4710.0(3) $Å^3$	
**Z**	8	
**Density (calculated)**	1.600 Mg/cm^3	
**Absorption coefficient**	2.524 mm^{-1}	
**F(000)**	2320	

Table S6. Data collection and structure refinement for **RhL4**.

**Diffractometer**	CCD Bruker Kappa APEX II DUO four-circle diffractometer
**Radiation source**	Ceramic x-ray tube (Mo Kα, λ = 0.71076 Å)
**Theta range for data collection**	2.505 to 27.928°

**Index ranges**	-44<=h<=44, -9<=k<=9, -29<=l<=30	
**Reflections collected**	48851	
**Independent reflections**	5621 [R(int) = 0.1158]	
**Coverage of independent reflections**	99.9%	
**Absorption correction**	Semi-empirical from equivalents	
**Max. and min. transmission**	0.7456 and 0.6583	
**Structure solution technique**	direct methods	
**Structure solution program**	SHELXL-2014	
**Refinement method**	Full-matrix least-squares on F^2	
**Refinement program**	SHELXL-2018	
**Function minimized**	$\Sigma w(F_o^2 - F_c^2)^2$	
**Data / restraints / parameters**	5621 / 0 / 263	
**Goodness-of-fit on F^2**	1.004	
**Final R indices**	4671 data; I>2σ(I)	R1 = 0.0420, wR2 = 0.0870
	all data	R1 = 0.0719, wR2 = 0.1013
**Weighting scheme**	$w=1/[\sigma^2(F_o^2)+(0.0459P)^2+10.3374P]$<br>where $P=(F_o^2+2F_c^2)/3$	
**Absolute structure parameter**		
**Largest diff. peak and hole**	0.805 and -0.952 eÅ^{-3}	
**R.M.S. deviation from mean**	0.136 eÅ^{-3}	

Table S7. Atomic coordinates (x 10^4) and equivalent isotropic atomic displacement parameters (Å2 x 10^3) for **RhL4**.

U(eq) is defined as one third of the trace of the orthogonalized U_{ij} tensor.

	**x/a**	**y/b**	**z/c**	**U(eq)**
Rh1	3746(1)	8292(1)	5251(1)	16(1)
Br1	3334(1)	5293(1)	4664(1)	24(1)
C1	3141(1)	9632(6)	4819(2)	17(1)
N1	2846(1)	9762(5)	5060(2)	17(1)
C2	2453(1)	10622(6)	4594(2)	20(1)
C3	2504(1)	11050(6)	4061(2)	20(1)
N2	2929(1)	10437(5)	4207(2)	17(1)
C4	2931(2)	9122(7)	5718(2)	24(1)
C5	3103(1)	10525(6)	3730(2)	21(1)
C6	2911(1)	8893(7)	3226(2)	21(1)
S1	3088(1)	8988(2)	2589(1)	23(1)
C7	3561(1)	7297(6)	2894(2)	17(1)
C8	3940(1)	7863(6)	3586(2)	18(1)

C9	4337(1)	6472(7)	3789(2)	23(1)
C10	4506(1)	6599(7)	3275(2)	26(1)
C11	4133(1)	6027(6)	2587(2)	22(1)
C12	3734(1)	7417(6)	2383(2)	21(1)
C13	3413(1)	5205(6)	2911(2)	22(1)
C14	3810(2)	3828(6)	3122(2)	26(1)
C15	4186(2)	4404(7)	3811(2)	26(1)
C16	3981(2)	3949(7)	2614(2)	26(1)
C17	4072(1)	10973(6)	5394(2)	22(1)
C18	4059(1)	10456(6)	5980(2)	23(1)
C19	4462(2)	9745(7)	6618(2)	28(1)
C20	4520(2)	7543(8)	6627(2)	29(1)
C21	4333(1)	6595(6)	5954(2)	20(1)
C22	4417(1)	7228(6)	5449(2)	19(1)
C23	4728(1)	8902(7)	5532(2)	23(1)
C24	4471(1)	10807(6)	5280(2)	23(1)

Crystal structure of cationic compound [RhL4][PF_6]

The crystals were placed in oil, and a single crystal was selected, mounted on a glass fibre and placed in a low-temperature N_2 stream.

X-ray diffraction data collection was carried out on a Bruker PHOTON III DUO CPAD diffractometer equipped with an Oxford Cryosystem liquid N_2 device, using Mo-Kα radiation (λ = 0.71073 Å). The crystal-detector distance was 37mm. The cell parameters were determined (APEX3 software)[16] from reflections taken from 1 set of 180 frames at 1s exposure. The structure was solved using the program SHELXT-2014[17]. The refinement and all further calculations were carried out using SHELXL-2014[18]. The H-atoms were included in calculated positions and treated as riding atoms using SHELXL default parameters. The non-H atoms were refined anisotropically, using weighted full-matrix least-squares on F^2. A semi-empirical absorption correction was applied using SADABS in APEX3[16]; transmission factors: T_{min}/T_{max} = 0.5741/0.7456.

A total of 3180 frames were collected. The total exposure time was 4 hours. The frames were integrated with the Bruker SAINT software package using a narrow-frame algorithm. The

integration of the data using a triclinic unit cell yielded a total of 190277 reflections to a maximum θ angle of 29.047° (0.71 Å resolution), of which 14766 were independent (average redundancy 6, completeness = 99.9%, R_{int} = 4.55%, R_{sig} = 1,87%) and 12941(87,64%) were greater than 2σ(F^2). The final cell constants of a = 11.7520(7) Å, b = 16.6122(11) Å, c = 16.8574(11) Å, β = 82.247(2)°, volume = 2769.5(3) $Å^3$, are based upon the refinement of the XYZ-centroids of 9177 reflections above 20 σ(I) with 4.90° < 2θ < 58.06°. Data were corrected for absorption effects using the Multi-Scan method (SADABS). The ratio of minimum to maximum apparent transmission was 0.953. The calculated minimum and maximum transmission coefficients (based on crystal size) are 0.7107 and 0.7458.

The structure was solved and refined using the Bruker SHELXTL Software Package, using the space group P -1, with Z = 4 for the formula unit, $C_{24}H_{36}F_6N_2PRhS$. The final anisotropic full-matrix least-squares refinement on F^2 with 627 variables converged at R1 = 5.10%, for the observed data and wR2 = 12.70% for all data. The goodness-of-fit was 1.048. The largest peak in the final difference electron density synthesis was 3.736 $e^-/Å^3$ and the largest hole was -1.495 $e^-/Å^3$ with an RMS deviation of 0.118 $e^-/Å^3$. On the basis of the final model, the calculated density was 1.517 g/cm^3 and F(000), 1296 e^-.

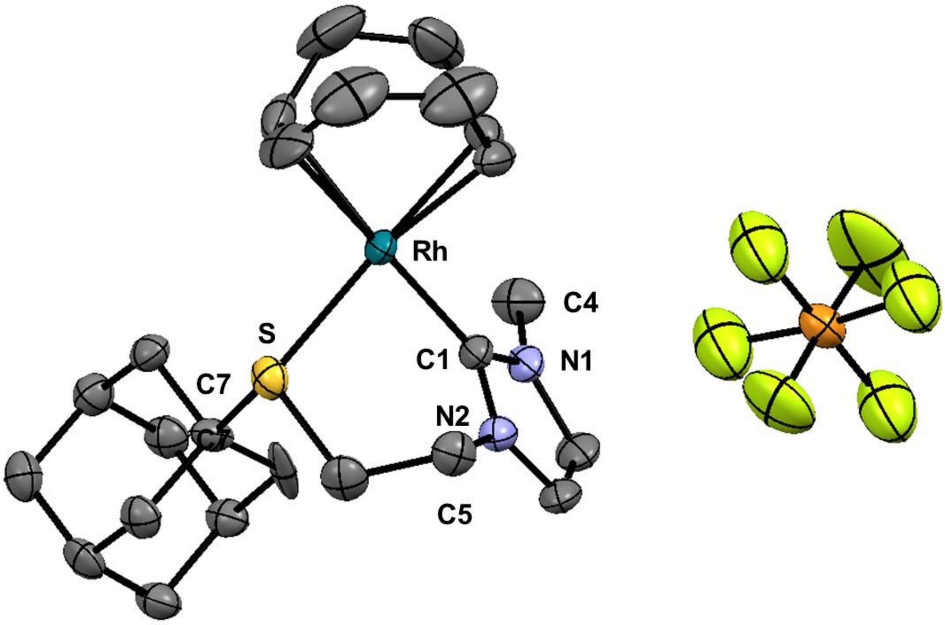

Figure S9. ORTEP representation of **[RhL4][PF$_6$]** with probability level of 50%, Hydrogen are omitted for clarity.

Table S8. Sample and crystal data for **[RhL4][PF_6].**

**Identification code**	sblta211216	
**Chemical formula**	$C_{24}H_{36}F_6N_2PRhS$	
**Formula weight**	632.49 g/mol	
**Temperature**	120(2) K	
**Wavelength**	0.71073 Å	
**Crystal size**	0.160 x 0.140 x 0.100 mm	
**Crystal habit**	yellow needle	
**Crystal system**	Triclinic,	
**Space group**	*P* -1	
**Unit cell dimensions**	a = 11.7520(7) Å	α = 61.611(2)°
	b = 16.6122(11) Å	β = 82.247(2)°
	c = 16.8574(11) Å	γ = 73.055(2)°
**Volume**	2769.5(3) $Å^3$	
**Z**	4	
**Density (calculated)**	1.517 Mg/cm^3	
**Absorption coefficient**	0.806 mm^{-1}	
**F(000)**	1296	

Table S9. Data collection and structure refinement for **[RhL4][PF_6]**.

**Diffractometer**	CCD Bruker Kappa APEX II DUO four-circle diffractometer	
**Radiation source**	Ceramic x-ray tube (Mo Kα, λ = 0.71076 Å)	
**Theta range for data collection**	2.000 to 29.047°	
**Index ranges**	-16<=h<=16, -22<=k<=22, -23<=l<=23	
**Reflections collected**	190277	
**Independent reflections**	14766 [R(int) = 0.0455]	
**Coverage of independent reflections**	99.9%	
**Absorption correction**	Semi-empirical from equivalents	
**Max. and min. transmission**	0.7458 and 0.7107	
**Structure solution technique**	direct methods	
**Structure solution program**	SHELXL-2018	
**Refinement method**	Full-matrix least-squares on F^2	
**Refinement program**	SHELXL-2018	
**Function minimized**	$\Sigma w(F_o^2 - F_c^2)^2$	
**Data / restraints / parameters**	14766 / 2 / 627	
**Goodness-of-fit on F^2**	1.048	
**Final R indices**	4671 data; I>2σ(I)	R1 = 0.0510, wR2 = 0.1270
	all data	R1 = 0.0588, wR2 = 0.1340
**Largest diff. peak and hole**	3.736 and -1.495 $eÅ^{-3}$	

Table S10. Atomic coordinates (x 10^4) and equivalent isotropic atomic displacement parameters (Å2 x 10^3) for **[RhL4][PF_6]**.

U(eq) is defined as one third of the trace of the orthogonalized U_{ij} tensor.

	**x/a**	**y/b**	**z/c**	**U(eq)**
C(1	8628(3)	7089(2)	6813(2)	23(1)
C(2)	10193(3)	6158(2)	7769(3)	32(1)
C(3)	9202(4	6108(2)	8267(2)	32(1)
C(4)	10667(3)	7012(3)	6135(3)	35(1)
C(5)	7003(3)	6870(3)	7931(2)	31(1)
C(6)	6523(3)	7856(3)	7862(2)	30(1)
C(7)	7602(3)	9424(2)	6769(2)	24(1)
C(8)	8795(3)	8795(2)	7227(2)	28(1)
C(9)	9566(3)	9419(3)	7190(3)	33(1)
C(10)	8935(4)	10007(3)	7671(3)	35(1)
C(11)	7734(4)	10642(3)	7204(3)	37(1)
C(12)	7943(5)	11297(3)	6211(3)	44(1)
C(13)	8576(4)	10707(3)	5735(3)	40(1)
C(14)	7804(4)	10086(2)	5780(2)	30(1)
C(15)	9779(4)	10075(3)	6199(3)	42(1)
C(16)	6958(3)	10016(3)	7250(2)	30(1)
C(17)	8270(3)	7737(3)	4706(2)	32(1)
C(18)	7721(3)	7032(2)	5282(2)	32(1)
C(19)	6517(4)	6964(3)	5138(3)	40(1)
C(20)	5580(4)	7926(3)	4738(3)	36(1)
C(21)	5714(3)	8580(2)	5087(2)	27(1)
C(22)	6334(3)	9256(2)	4660(2)	26(1)
C(23)	7042(4)	9434(3)	3807(2)	34(1)
C(24)	7798(4)	8526(3)	3785(2)	37(1)
N(1)	9834(2)	6754(2)	6878(2)	26(1)
N(2)	8248(3)	6677(2)	7677(2)	26(1)
F(1)	7621(7)	2083(5)	2162(6)	93(1)
F(2)	7075(6)	3466(5)	2388(5)	93(1)
F(3)	5513(6)	2972(5)	3117(5)	93(1)
F(4))	6100(8)	1664(6)	2999(7)	93(1)

F(5)	5896(4)	3144(3)	1795(3)	88(1)
F(6)	7517(6)	1878(4)	3518(3)	136(2)
P(1)	6722(1)	2526(1)	2670(1)	40(1)
S(1))	6576(1)	8789(1)	6714(1)	23(1)
Rh(1)	7437(1)	8032(1)	5798(1)	20(1)

References

(1) Artigas, A.; Castanyer, C.; Roig, N.; Lledó, A.; Solà, M.; Pla-Quintana, A.; Roglans, A. Synthesis of fused dihydroazepine derivatives of fullerenes by a Rh-catalyzed cascade process. *Adv. Synth. Catal.* **2021**, *363*, 3835–3844.

(2) Vila, J.; Vinardell, R.; Solà, M.; Pla-Quintana, A.; Roglans, A. A Rh(I)-catalyzed cascade cyclization of 1,5-bisallenes and alkynes for the formation of *cis*-3,4-arylvinyl pyrrolidines and cyclopentanes. *Adv. Synth. Catal.* **2022**, *364*, 206–217.

(3) Artigas, A.; Vila, J.; Lledó, A.; Solà, M.; Pla-Quintana, A.; Roglans, A. A Rh-catalyzed cycloisomerization/Diels-Alder cascade reaction of 1,5-bisallenes for the Synthesis of polycyclic heterocycles. *Org. Lett.* **2019**, *21*, 6608–6613.

(4) Yuan, D.; Tang, H.Y.; Xiao, L.F.; Huynh, H. V. CSC-pincer versus pseudo-pincer complexes of palladium(II): a comparative study on complexation and catalytic activities of NHC complexes. *Dalton Trans.* **2011**, *40*, 8788–8795.

(5) Field, L.D.; Messerle, B.A.; Vuong, K Q.; Turner, P. Intramolecular hydroamination with Rhodium(I) and Iridium(I) complexes containing a phosphine−N-heterocyclic carbene ligand. *Organometallics*, **2005**, *24*, 4241–4250.

(6) Fliedel, C.; Schnee, G.; Braunstein, P. Versatile coordination modes of novel hemilabile S-NHC ligands. *Dalton Trans.* **2009**, 2474–2476.

(7) Chen, W.; Egly, J.; Pobtador-Bahamonde, A.I.; Maisse-Francois, A.; Bellemin-Laponnaz, S.; Achard T. Synthesis, characterization, catalytic and biological application of half-sandwich ruthenium complexes bearing hemilabile (κ2–C,S)-thioether-functionalised NHC ligands. *Dalton Trans.* **2020**, *49*, 3243- 3252.

(8) Gaussian 16, Revision A.03, Frisch, M.J.; Trucks, G.W.; Schlegel, H.B.; Scuseria, G.E.; Robb, M.A.; Cheeseman, J.R.; Scalmani, G.; Barone, V.; Petersson, G.A.; Nakatsuji, H.; Li, X.; Caricato, M.; Marenich, A.V.; Bloino, J.; Janesko, B.G.; Gomperts, R.; Mennucci, B.; Hratchian, H.P.; Ortiz, J.V.; Izmaylov, A.F.; Sonnenberg, J.L.; Williams-Young, D.; Ding, F.; Lipparini, F.; Egidi, F.; Goings, J.; Peng, B.; Petrone, A.; Henderson, T.; Ranasinghe, D.; Zakrzewski, V.G.; Gao, J.; Rega, N.; Zheng, G.; Liang, W.; Hada, M.; Ehara, M.; Toyota, K.; Fukuda, R.; Hasegawa, J.; Ishida, M.; Nakajima, T.; Honda, Y.; Kitao, O.; Nakai, H.; Vreven, T.; Throssell, K.; Montgomery, J.A., Jr.; Peralta, J.E.; Ogliaro, F.; Bearpark, M.J.; Heyd, J.J.; Brothers, E.N.; Kudin, K.N.; Staroverov, V.N.; Keith, T.A.; Kobayashi, R.; Normand, J.; Raghavachari, K.; Rendell, A.P.; Burant, J.C.; Iyengar, S.S.; Tomasi, J.; Cossi, M.; Millam, J.M.; Klene, M.; Adamo, C.; Cammi, R.; Ochterski, J.W.; Martin, R.L.; Morokuma, K.; Farkas, O.; Foresman, J. B.; Fox, D.J. Gaussian, Inc., Wallingford CT, 2016.

(9) (a) Stephens, P.J.; Devlin, F.J.; Chabalowski, C.F.; Frisch, M.J. Ab Initio calculation of vibrational absorption and circular dichroism spectra using Density Functional Force

Fields. *J. Phys. Chem.* **1994**, *98*, 11623–11627. (b) Becke, A.D. Density-functional thermochemistry. III. The Role of exact exchange. *J. Chem. Phys.* **1993**, *98*, 5648–5652. (c) Lee, C.; Yang, W.; Parr, R.G. Development of the Colle-Salvetti correlation-energy formula into a Functional of the Electron Density. *Phys. Rev. B* **1988**, *37*, 785–789.

(10) (a) Dunning, T.H. Gaussian basis sets for use in correlated molecular calculations. I. The atoms Boron through Neon and Hydrogen. *J. Chem. Phys.* **1989**, *90*, 1007–1023. (b) Woon, D.E.; Dunning, T.H. Gaussian basis sets for use in correlated molecular calculations. III. The atoms Aluminum through Argon. *J. Chem. Phys.* **1993**, *98*, 1358–1371.

(11) Peterson, K.A.; Figgen, D.; Dolg, M.; Stoll, H. Energy-consistent relativist pseudopotentials and correlation consistent basis sets for the 4d elements Y-Pd. *J. Chem. Phys.* **2007**, *126*, 124101.

(12) Chai, J.-D.; Head-Gordon, M. Long-range corrected hybrid density functional with damped atom–atom dispersion corrections. *Phys. Chem. Chem. Phys.*, **2008**,*10*, 6615–6620.

(13) Marenich, A.V.; Cramer, C.J.; Truhlar, D. G. Universal solvation model based on solute electron density and on a continuum model of the solvent defined by the bulk dielectric constant and atomic surface tensions. *J. Phys. Chem. B* **2009**, *113*, 6378–6396.

(14) Grimme, S.; Antony, J.; Ehrlich, S.; Krieg, H. A consistent and accurate Ab Initio parametrization of density functional dispersion correction (DFT-D) for the 94 elements H-Pu. *J. Chem. Phys.* **2010**, *132*, 154104.

(15) Atkins, P.; De Paula, J.; Keeler, J. (2017). *Atkins' physical chemistry* (11th ed.). Oxford University Press.

(16) "M86-EXX229V1 APEX3 User Manual", Bruker AXS Inc., Madison, USA, 2016.

(17) Sheldrick, G.M. SHELXT – Integrated space-group and crystal-structure determination. *Acta Cryst.* **2015***,* A71, 3-8.

(18) Sheldrick, G.M. Crystal structure refinement with SHELXL. *Acta Cryst.* **2015***,* C71, 3-8.
